# Selection for Unequal Densities of σ^70^ Promoter-Like Signals in Different Regions of Large Bacterial Genomes

**DOI:** 10.1371/journal.pgen.0020185

**Published:** 2006-11-10

**Authors:** Araceli M Huerta, M. Pilar Francino, Enrique Morett, Julio Collado-Vides

**Affiliations:** 1 Centro de Ciencias Genómicas, Universidad Nacional Autónoma de México, Cuernavaca, México; 2 Evolutionary Genomics Program, Department of Energy Joint Genome Institute, and Genomics Division, Lawrence Berkeley National Laboratory, Walnut Creek, California, United States of America; 3 Departamento de Ingeniería Celular y Biocatálisis, Instituto de Biotecnología, Universidad Nacional Autónoma de México, Cuernavaca, México; INSERM U571, France

## Abstract

The evolutionary processes operating in the DNA regions that participate in the regulation of gene expression are poorly understood. In *Escherichia coli,* we have established a sequence pattern that distinguishes regulatory from nonregulatory regions. The density of promoter-like sequences, that could be recognizable by RNA polymerase and may function as potential promoters, is high within regulatory regions, in contrast to coding regions and regions located between convergently transcribed genes. Moreover, functional promoter sites identified experimentally are often found in the subregions of highest density of promoter-like signals, even when individual sites with higher binding affinity for RNA polymerase exist elsewhere within the regulatory region. In order to see the generality of this pattern, we have analyzed 43 additional genomes belonging to most established bacterial phyla. Differential densities between regulatory and nonregulatory regions are detectable in most of the analyzed genomes, with the exception of those that have evolved toward extreme genome reduction. Thus, presence of this pattern follows that of genes and other genomic features that require weak selection to be effective in order to persist. On this basis, we suggest that the loss of differential densities in the reduced genomes of host-restricted pathogens and symbionts is an outcome of the process of genome degradation resulting from the decreased efficiency of purifying selection in highly structured small populations. This implies that the differential distribution of promoter-like signals between regulatory and nonregulatory regions detected in large bacterial genomes confers a significant, although small, fitness advantage. This study paves the way for further identification of the specific types of selective constraints that affect the organization of regulatory regions and the overall distribution of promoter-like signals through more detailed comparative analyses among closely related bacterial genomes.

## Synopsis

The most important step in the regulation of genetic expression is the initiation of transcription. This process is accomplished by the association or specific binding of RNA polymerase to particular sequence segments present in the DNA, the promoters. Promoters are located in the upstream regions of the transcribed genes. The evolutionary processes operating in the DNA regions that participate in the regulation of gene expression are poorly understood. For a long time, the canonical picture of a σ^70^ promoter has been a 60 base pair region defined by the transcription start-point (+1) and two conserved hexanucleotide sequences centered 10 and 35 base pairs upstream from the +1. The authors have shown that in *Escherichia coli,* promoters exist in clusters, as a series of overlapping potentially competing RNAP interaction sites. The E. coli regulatory regions contain high densities of these promoter-like signals, in contrast to coding regions and regions located between convergently transcribed genes. They report that the differential densities between regulatory and nonregulatory regions are detectable in most eubacterial genomes, with the exception of those that have experienced severe genome degradation and size reduction. This suggests that the presence of this pattern in large bacterial genomes confers a significant, although small, fitness advantage.

## Introduction

For both prokaryotes and eukaryotes, understanding of the organizational structure and mode of evolution of regulatory DNA sequences is incomplete. Although in the Bacteria gene regulation involves fewer proteins and *cis*-regulatory DNA sites, and less complex interactions among them, recent findings suggest that the classic description of bacterial promoter regions may have been significantly oversimplified [1]. In *Escherichia coli,* RNA polymerase (RNAP) is composed of a core complex of α, β, β′, and ω subunits and one of a variety of σ factors, the primary one being σ^70^, which is essential for general transcription in exponentially growing cells. The canonical model of the σ^70^ promoter is defined as a simple pair of hexamers, positioned at −35 and −10 base pairs (bp) from the transcription start (+1), with respective consensus sequences TTGACA and TATAAT, and separated by a spacer of 15 to 21 bp [2]. RNAP-σ^70^ can recognize and bind −35 and −10 motifs that differ substantially from their consensus sequences, although mutations that bring these motifs closer to the consensi generally increase promoter strength [3]. On average, E. coli promoters preserve only eight of the 12 canonical bases of the −35 and −10 hexamers [4,5]. It has been recently shown that most of the regulatory regions in E. coli do not contain a single promoter sequence [1] but rather display high densities of potential RNAP-σ^70^ binding sites, forming clusters of overlapping promoter-like signals. In contrast, such signal densities are not detected in coding regions and regions located between convergently transcribed genes. Moreover, functional promoter sites identified experimentally are often found within the subregions of highest density of overlapping signals, even when individual sites with higher binding affinity for RNAP exist elsewhere within the region [1].

Even though the degeneracy of the σ^70^-binding promoter motifs ensures that new sites can evolve (i.e., appear and become fixed in populations) via local point mutation on short timescales [6], random fixation of mutations by neutral drift could not explain the different promoter-like signal densities between regulatory and nonregulatory regions of E. coli reported in [1]. Moreover, it has been shown that natural selection acts to remove spurious occurrences of the two consensus words of the σ^70^ promoter (TTGACA and TATAAT) from both coding and noncoding regions in several eubacterial genomes, implying that it is disadvantageous to maintain misplaced sites which can strongly bind RNAP-σ^70^ and interfere with proper gene expression [7]. This suggests that the observed excess of promoter-like signals in regulatory regions is likely to be the result of natural selection for some past or present function.

Here we report that differential densities of promoter-like signals between regulatory and nonregulatory regions are detectable in most bacterial genomes, with the exception of those that have experienced severe size reduction. We argue that the phylogenetic distribution of this differential density pattern implies that this genomic feature is maintained by weak natural selection, and we discuss possible functional roles for the high redundancy of promoter-like signals in the regulatory regions of large bacterial genomes.

## Results/Discussion

**Figure 1:**
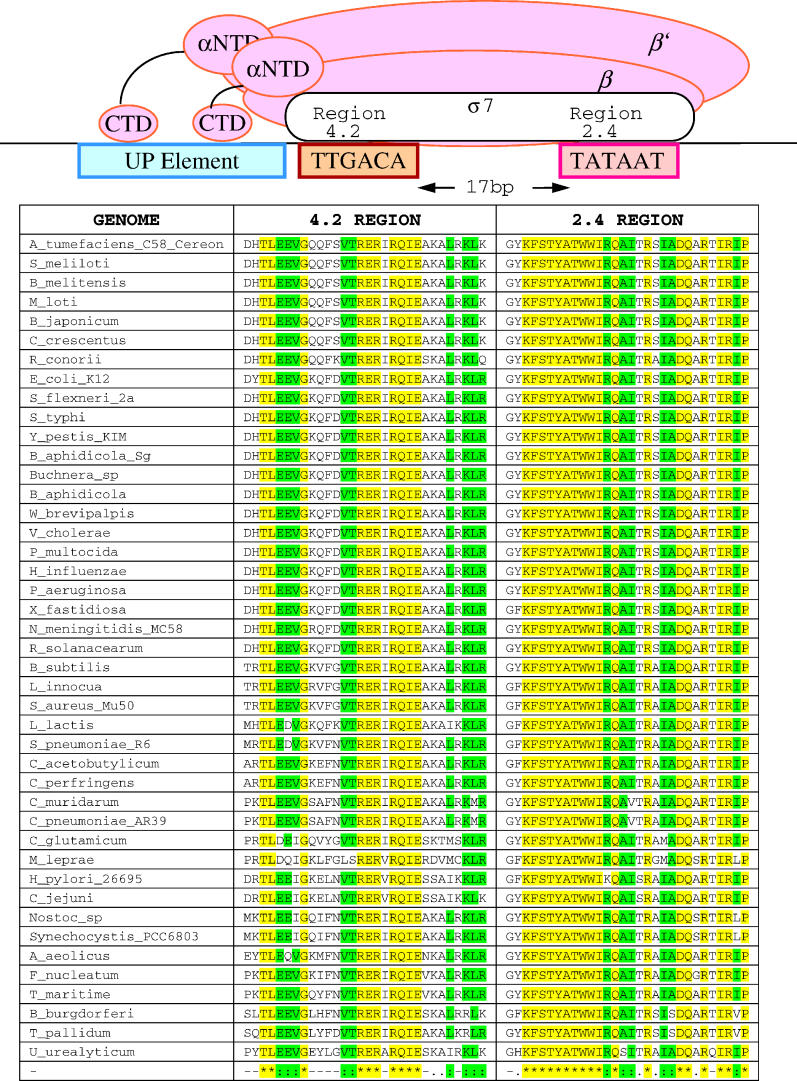
Schematic Representation of the Main Interactions of RNAP with Promoter DNA and Alignment of the σ^70^ Motifs for Recognition and Binding of −10 (2.4 Region) and −35 Promoter Sequences (4.2 Region) for Representative Eubacteria Sequence alignments of several σ^70^ factors from different bacteria reveal four conserved regions that can be further divided into subregions [39]. Only regions 2 and 4 are well conserved in all members of the σ^70^ family [40–43] and include subregions involved in binding to the core RNAP complex, promoter melting, and recognition of the −10 and −35 promoter sequences (regions 2.4 and 4.2, respectively) [10,40,44]. CLUSTALW was used to generate the alignment with default parameters (http://www.ebi.ac.uk/clustalw) [45].

**Figure 2:**
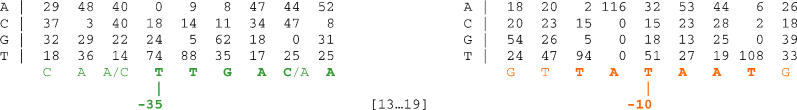
Frequency Matrices for the −10 and −35 Motifs of σ^70^ Promoters in E. coli This matrix pair (Matrix_18_15_13_2_1.5) was selected for searching across bacterial genomes from a collection of optimized matrices defined for E. coli in [1]. Note that in order to compare these matrices with the canonical patterns (TTGACA and TATAAT), the spacers of 13 bp to 19 bp between the two boxes correspond to the 15 bp to 21 bp reported in the literature, as the TGT triplet is considered as part of the −10 box. Before searching for promoter-like signals, these matrices were calibrated using the noncoding base composition of each target genome.

In order to explore whether the differential promoter signal density pattern discovered in E. coli is common to other bacterial species, we conducted similar analyses for a representative set containing 43 additional genomes belonging to different genera across all major bacterial phyla. This comparison is valid given that RNAP is evolutionarily conserved across Bacteria. Moreover, there seems to be only one housekeeping σ^70^ factor present in any given species [8,9], and all eubacterial σ^70^ protein sequences can be clearly aligned and contain highly similar motifs for the recognition and binding of −10 and −35 promoter sequences [10] (Figure 1). This implies that the DNA sequences of promoter motifs in these organisms must also be similar to those found in E. coli. Therefore, we used in our searches position weight matrices (PWMs) describing the −10 and −35 sequences determined in E. coli [1] and specifically calibrated to the base composition of strictly noncoding regions of each analyzed genome (Figure 2).

Table 1 reports for every genome the consensus and average score of the detected −10 and −35 motifs. The consensi were obtained after applying the COVER Function [1] on the set of promoter-like signals found by the PWM search in the functional regulatory regions of each genome (see Materials and Methods). From Table 1, it can be seen that all the additional 43 species analyzed have consensus sequences for both motifs highly similar to those of *E. coli,* as well as average scores of comparable magnitudes. In fact, large GC-rich genomes (*Pseudomonas, Ralstonia,* and most of the alpha-proteobacteria) display motif scores above those of *E. coli,* probably due to the greater compositional difference between the AT-rich motifs and the background genomic sequence. In contrast, small GC-poor genomes have lower motif scores, with the insect endosymbiont *Wigglesworthia* displaying the lowest.

**Table 1:**
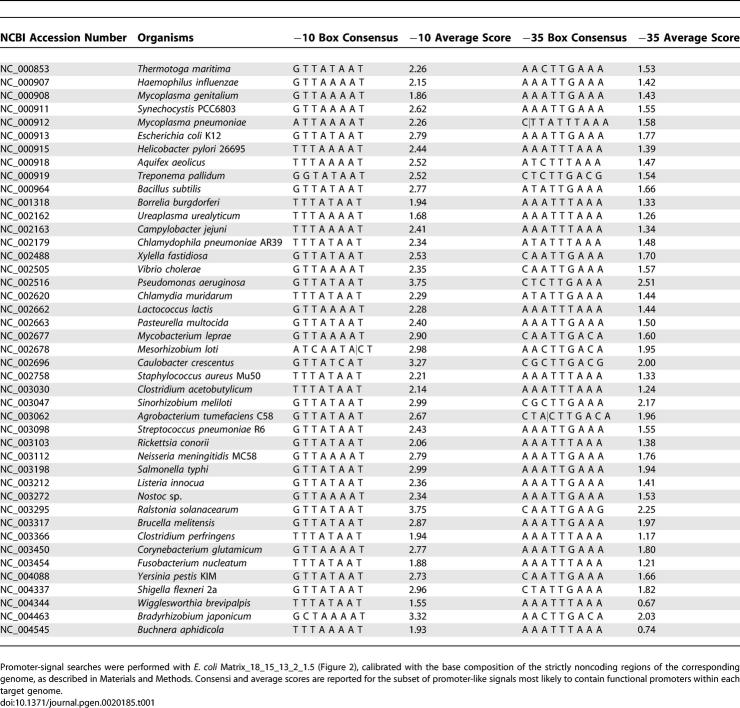
−10 and −35 Consensi and Corresponding Average Scores for σ^70^ Promoter-Like Signals in Representative Bacterial Genomes

**Figure 3:**
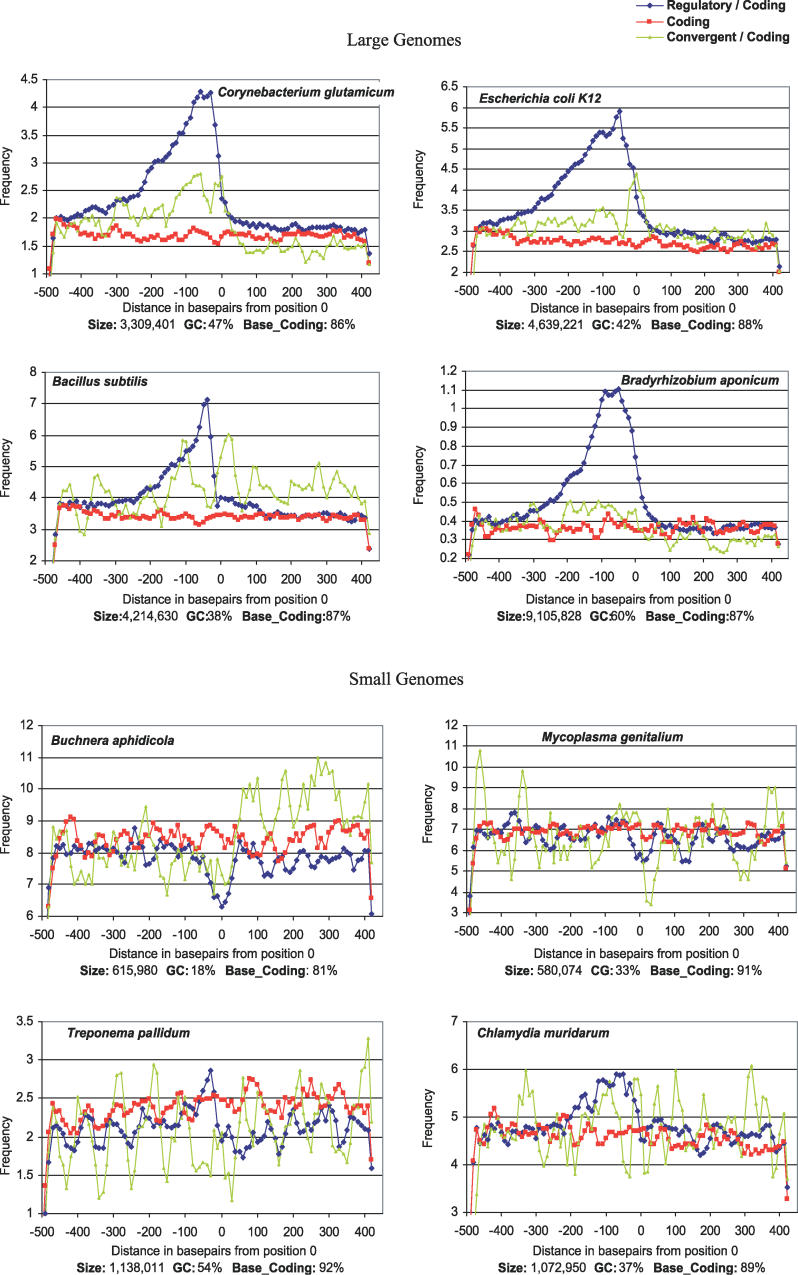
Signal Density in Regulatory versus Nonregulatory Regions of Large and Small Eubacterial Genomes Regulatory regions correspond to the strictly noncoding regions located upstream of a gene start. For the set of genomes selected in this study, the average size of the strictly noncoding upstream regions is 182 bp. For the sake of the graph, the regions were extended to 500 bases upstream and 500 bases downstream of the start of the gene (position 0). Nonregulatory regions include the coding regions and the noncoding regions between convergently transcribed genes. For coding regions, the middle point of a gene was taken as the position 0 and 500 bases upstream and 500 bases downstream of this position were included. For the set of genomes analyzed here, the average size of the convergent regions is 194.5 bp. For the sake of the graph, the end of the 3′ gene was taken as position 0 and 500 bases upstream and 500 bases downstream of this position were included. The number of signals was averaged within intervals of 10 bp.

Table 2 shows the promoter-like signal density patterns detected for all the genomes analyzed. Two main alternative profiles were obtained, as illustrated in Figure 3 (profiles for all species analyzed are available in Figure S1 and at http://www.ccg.unam.mx/Computational_Genomics/PromotorTools/PLoS06/Figure_S1.hmtl). Regulatory and nonregulatory regions contain differential densities of promoter-like signals in 24 genomes, including genera belonging to phyla distantly related to E. coli, such as the *Firmicutes, Actinobacteria, Cyanobacteria,* and *Thermotogae*. Clearly, the presence of the pattern is highly dependent on genome size (Pearson's correlation = 0.59, *p* < 0.001). All genomes above 4 Mb display marked differences in promoter-like signals between regulatory and nonregulatory regions, whereas none of the genomes under 1.5 Mb do so. Among the genomes of intermediate size, the pattern is detectable in 65% of the cases. There is also a notable effect of GC content (Pearson's correlation = 0.58, *p* < 0.001). Although the differential signal densities can be seen in genomes of very different GC contents (from 30% GC Lactococcus lactis to 62% GC Ralstonia solanacearum and Caulobacter crescentus), overall, 75% of GC-rich genomes display the differential pattern, in contrast to 53% of those under 50% GC. Multiple regression analysis reveals that up to 0.43 of the variance in pattern overrepresentation might be related to these two variables, genome size and GC content (for a correlation of about 0.66).

**Table 2:**
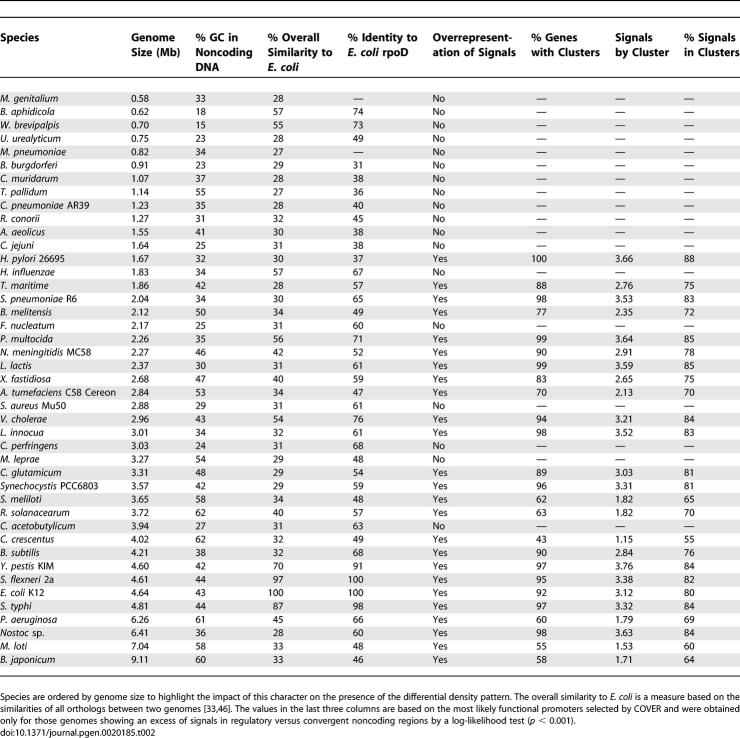
Density Patterns of σ^70^ Promoter-Like Signals in Eubacterial Genomes

To test if the accumulation of promoter-like signals observed in regulatory regions is due to AT-richness of these sequences, we generated randomized regulatory sequences (see Materials and Methods) and we searched for promoter-like signals. As expected, the randomized regulatory sequences of large genomes show flattened distributions with significantly less promoter-like signals than real regulatory regions (*p* < 0.001), meaning that observed densities in regulatory regions are not due to an AT bias. Results for random regulatory regions are available in Figure S2 and at http://www.ccg.unam.mx/Computational_Genomics_PromotorTools/PLoS06/Figure_S2.html.

The observations here reported strongly suggest that the differential density of promoter-like signals in regulatory and nonregulatory regions of large bacterial genomes is maintained by natural selection. First, the fact that this pattern is observed in phylogenetically distant genomes argues against mutational biases being its main source, since mutational biases are known to vary among genomes, particularly when there are large differences in GC content. Second, differential signal density is highly dependent on large genome size, and is completely absent from most of the small genomes of animal parasites and symbionts with an intracellular or predominantly host-restricted lifestyle *(Mycoplasma, Ureaplasma, Treponema, Borrelia, Campylobacter, Rickettsia, Buchnera,* and *Wigglesworthia)*. And third, analysis on conservation of promoter-like signals in a group of 14 related species shows 79% signal conservation (see Materials and Methods).

### Degradation of Regulatory Functions in the Small Genomes of Host-Restricted Bacteria

Acquisition of such obligate dependence on a host is known to have many deleterious consequences, collectively known as genome degradation or genome reduction [11]. Reduced genomes have independently evolved many times within several bacterial phyla, repeatedly undergoing rapid sequence evolution and acquiring extremely low GC content, often with clearly maladaptive consequences, including accumulation of deleterious amino acid substitutions and loss of adaptive codon biases [12,13]. Typical changes also include a large increase in the frequency of mobile elements in the early phases of genome degradation, chromosomal rearrangements mediated by recombination among these elements, pseudogene formation, and deletions of varying size. There is recent evidence that genome degradation also affects gene regulation, due to losses of certain promoter sequences, specialized σ factors, and regulatory proteins [14–16]. These common changes likely reflect a diminished capacity of reduced genomes to respond to natural selection, conducive to the accumulation of all types of moderately deleterious mutations that would normally be purged from the genomes of free-living bacteria. This lowered effectiveness of purifying selection is due to the subdivided population structure imposed by confinement within a host, which limits the effective size of the bacterial population by subjecting it to recurrent bottlenecks and by thwarting opportunities for recombination with close relatives [17]. In addition, different types of molecular evolutionary analyses indicate that the rate of generation of point mutations is accelerated in reduced genomes [18,19].

We argue that a decrease in the efficiency of purifying selection in host-restricted bacteria allows promoter-like signals to rapidly accumulate all along the genomic sequence of these organisms, causing the loss of differential signal density patterns. This interpretation is based on the following evidence: (i) simulation results demonstrating that transcription factor binding sites can rapidly appear as a consequence of local point mutations on short time scales without invoking selection [6]; (ii) the observation that natural selection can act to remove spurious transcription factor binding sites from nonregulatory regions in many bacterial genomes, with a weak strength similar to that of selection on adaptive codon bias [7]; (iii) empirical and theoretical results demonstrating that the effectiveness of selection is diminished in the small, highly structured populations of host-restricted bacteria [11–17]; (iv) the finding that promoter-like sequences in these organisms show a certain degree of degradation reflected in their decreased scores for the −10 and −35 motifs (Table 2), suggesting an overall reduction of the efficiency of selection on regulatory function; and (v) the fact that the nonregulatory regions of host-restricted bacteria present a frequency of promoter-like signals similar to the highest frequency peak found inside the regulatory regions of their commensal or free-living relatives (Figure 3). Figure 3 shows the frequency of promoter-like signals in *Buchnera aphidicola, Mycoplasma genitalium,* and their relatives E. coli and Bacillus subtilis. The increased frequency of promoter-like signals could have regulatory implications by making it more difficult for RNAP to correctly identify regulatory regions and the functional promoters within them. In that case, one could expect a slow response to changes in cellular conditions and/or a decreased level of gene expression.

**Figure 4:**
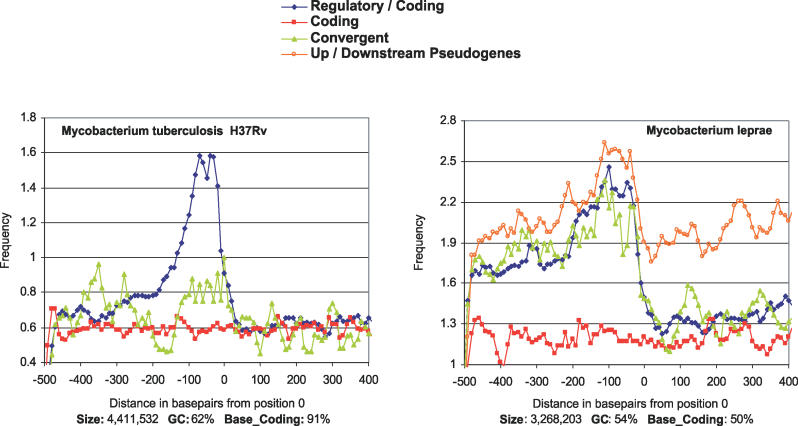
Signal Density in Regulatory versus Nonregulatory Regions of M. tuberculosis and M. leprae M. leprae shows an increase of promoter-like signals in the upstream and downstream regions of pseudogenes relative to the regulatory regions. M. leprae contains over 1,115 recognizable pseudogenes [20]. Both 500 bp upstream and downstream of the start of pseudogenes (position 0) were analyzed for searches of promoter-like signals. All other region definitions and methodology are as in Figure 3.

To further test whether genome degradation produces loss of differential signal densities, we decided to compare signal density patterns between a genome that has recently entered a process of degradation and close relatives evolving under stronger purifying selection. Our initial analyses pinpointed *Mycobacterium leprae,* the obligate intracellular pathogen that causes leprosy, as one of the rare genomes above 3 Mb for which differential promoter signal densities are not detected. However, although the genome of M. leprae remains relatively large (3.27 Mb) and GC-rich (58% overall), it is clearly decreasing in size and GC content relative to its closest relative, M. tuberculosis (4.41 Mb and 65.6% overall GC). Genome comparisons between different mycobacterial species indicate that M. leprae has also undergone massive gene decay by pseudogenization and deletion, as well as numerous genomic rearrangements likely due to the proliferation of IS elements [20]. As we expected, analyses of promoter-like signals in two strains of M. tuberculosis do reveal the differential signal densities characteristic of large genomes (Figure 4 and Table 3). Both M. tuberculosis strains display higher average scores than M. leprae for the −35 and −10 promoter motifs (Table 3), supporting the idea that the leprosy bacillus is undergoing a general degradation of its regulatory sequences. Preliminary comparative analyses between the upstream regions of pseudogenes in M. leprae and the regulatory regions of M. leprae and M. tuberculosis reveal an evident increase of promoter-like signals in the upstream regions of pseudogenes (Figure 4). Studies about mechanisms that prevent the unnecessary expression of degenerated genes suggest that the accumulation of mutations in ribosome-binding sites and promoter regions provoke a translational and transcriptional silencing of pseudogenes [21]. Overall, the promoter-like signals upstream of M. leprae pseudogenes still retain the conserved −35 and −10 conseni of σ^70^ promoters (“TTGACA” and “TATAAT”, respectively), indicating that promoter degeneration is not yet pervasive across the pseudogenes of this species. However, the average scores of the −35 and −10 motifs of promoters in pseudogenes are below the average scores of the promoters found in the regulatory regions, especially for the −35 motif (Table 3), implying a certain level of degradation of these signals. It is known that the −35 box is dispensable for transcription initiation as long as there is a regulatory mechanism to anchor the polymerase in the promoter, which can be achieved by regulatory proteins or extended −10 boxes. We suggest that excessive density of lightly degraded promoter-like signals may be silencing pseudogenes in two ways: (i) by attracting the polymerase to sites that could be competing with the “real” promoter and/or (ii) by presenting polymerase binding sites where the −35 degraded box is not sufficient to support open complex formation. Table S1 in Supporting Information shows selected comparisons between M. leprae pseudogenes and their corresponding functional equivalents in M. tuberculosis.

**Table 3:**
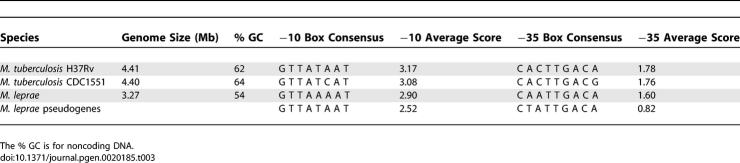
−10 and −35 Consensi and Average Scores for the Degrading Genome of M. leprae and Its Close Relative M. tuberculosis

### Potential Functions of the Promoter-Like Signals

In the course of our analyses, we identify two types of promoter-like signal densities: a global density and a local density (Figures 4 and 7 in [1]). The global density is obtained during the search for the −10 and −35 motifs using PWMs. In *E. coli,* this search produces an average of 38 promoter-like signals per 250-bp regulatory region, which may be distributed evenly across the sequence or present different levels of overlap. The second type of density, the local density, results from applying the COVER function [1], which establishes a competition on the set of promoter-like signals in terms of −10 and −35 score and position of the signal regarding the gene start, selecting the more probable candidates to be functional promoters (see Material and Methods section for details). In *E. coli,* COVER produces 4.7 signals in average per 250-bp regulatory region. Most of these signals tend to exist as a series of overlapping potentially competing RNAP binding sites. The average size of these clusters of overlapped signals is 42 bp. Seventy-four percent of the E. coli experimentally mapped promoters are embedded in this kind of clusters [1]. We suggest that each of these types of densities may be maintained by natural selection for very different reasons.

#### Global density of promoter-like signals in regulatory regions.

It is most likely that the global density is largely built by promoter-like signals that do not substitute the function of the primary promoter which has to respond to a given cellular condition; this would be the case if the majority of detected signals could bind RNAP and form a closed complex but were not able to proceed with the subsequent steps required to initiate transcription. However, a subset of these promoter-like signals could be just a single point mutation away from being able to operate as active transcription initiation sites. These could be called cryptic promoters, in analogy to cryptic genes that can be activated by single mutations. Some cryptic promoters could be relics of ancient promoters, indicating the high frequency of changes in regulatory regions. This high frequency correlates with the observed high flexibility in the evolution of transcriptional regulators in bacteria [22].

The permanence of cryptic promoters in the regulatory regions of bacteria could be facilitated by different kinds of evolutionary processes. First, they could constitute a collection of “back up” promoter sequences, maintained by selection for robustness. Bacteria having redundant signals capable of acquiring functionality with a single base change would increase in frequency in the population, because a fraction of their descendent cells would carry such activating mutations and would be able to survive in the advent of a deleterious mutation that destroyed the main promoter. In other words, the existence of multiple potential promoters would minimize the deleterious effects of genetic mutations on gene expression. In addition, for bacterial species prone to encounter a variety of environments, the existence of cryptic promoters of different strength could also allow for rapid evolution of changes in gene expression allowing adaptation to environmental changes. Taking into account that for σ^70^ transcription, the precise positioning of the regulatory proteins strongly determines their positive or negative role [23], this large availability of promoter-like sequences provides a fertile ground for quick changes in the role of regulatory proteins. Those changes have been proposed to depend on the demand of gene expression in a model where selection governs changes in gene regulation [24].

It is also possible that some of the signals detected in regulatory regions are not cryptic but rather fully functional promoters that are utilized only in special conditions. Although, in general, the site of transcription initiation is known to be rather precise, 25% of the transcription units in E. coli are known to be transcribed by more than one single promoter. The average number of mapped promoters coexisting in multiple-promoter regulatory regions is 3 [1,25]. The simultaneous availability of alternative promoters for a given gene would provide plasticity of gene expression in response to different conditions regularly encountered by the bacterial cell.

The regulatory region of the *E. coli lac* operon exhibits signals of both types. Six promoter sequences have been experimentally detected in close proximity to the primary *lac* promoter *(lacP1)*. Four signals were created via single base pair mutations in the wild sequence, and two were detected in vivo as weak promoters that function when the primary promoter is impaired and its activator protein is absent [26,27].

Finally, the global density of promoter-like motifs in the regulatory regions could be bringing the polymerase near the promoter during the random DNA search prior to forming closed complexes, by attracting the enzyme to the general vicinity of the functional promoter. However, kinetic studies suggest that this might only result in a minor increase in the rate of formation of closed complexes (Jay D. Gralla, personal communication).

#### Local density of overlapping promoter-like signals.

Overlapping promoter-like signals could play a regulatory role through functional interaction with the true promoter sequence, and their effect on regulation could be negative or positive.

Overlapping signals could negatively affect regulation by different means. The overlapping promoter-like signals might play a negative role if their interaction with RNAP were competitive. When two or more promoters are in close proximity, the potential exists for competition between them for the binding of RNAP [28]. Regulatory proteins play an important role in helping RNAP to choose the functional promoter sequence according to specific conditions. The regulation of the *gal* gene is an example of the effect of regulatory proteins on the positioning of RNAP between two competing promoters [29]. Also, the promoter-like signals could also induce pauses in the early steps of elongation when σ^70^ is still bound to core RNAP. The strongest evidence indicating that σ^70^ can play a functional role during elongation comes from studies of the bacteriophage λ PR′ promoter and the lacUV5 promoter. Biochemical experiments have shown that σ^70^-dependent pause occurs during early elongation in these promoters after RNAP has escaped from the promoter and synthesized a 16 or 17 nucleotide transcript. This pause is mediated by protein–DNA interaction between σ^70^ and a DNA sequence element in the initially transcribed region that resembles a promoter −10 element [30,31].

On the other hand, overlapping signals could also affect regulation positively. Some of these sites could be noncompetitive weak promoters that, in the absence of activation of the primary promoter, produce basal transcription of downstream genes. For example, transcription units that encode their own regulator would require constitutive levels of basal transcription. The overlapping promoter-like signals might also play a positive role by collecting RNAP molecules which could then be channeled to the primary promoter sequence [32].

### Conclusion

Clearly, the distribution of the differential pattern of promoter-like signal densities among bacterial genomes mirrors that of genes and other genomic features that require weak selection to be effective in order to persist. This implies that the differential density of promoter-like signals between regulatory and nonregulatory regions confers some small but significant fitness advantage. Therefore, the outcome of gene regulation could be affected by factors much beyond the sequence of a single pair of RNAP binding sites in a given regulatory region, including the general abundance and organization of promoter-like signals in the region, as well as the presence of signals in the non-regulatory portions of the genome. Identification of the specific types of selective constraints that shape the number, position and arrangement of promoter-like signals across the different genomic regions of large bacterial genomes will require further comparative analyses among closely-related bacteria.

## Materials and Methods

### Sequence data.

To evaluate the genomic frequency of promoter-like signals, three kinds of regions were defined in each genome according to National Center for Biotechnology Information (NCBI) annotations: coding, convergent, and strictly noncoding (excluding the convergent regions).

Coding regions contain genes with sizes above 1 kb. Convergent regions are noncoding regions located between the ends of two genes convergently transcribed. Convergent regions are analyzed separately because they are not expected to contain any functional promoters. In contrast, the strictly noncoding regions are located upstream of a gene start and are likely to contain regulatory regions.

To estimate the most likely functional promoters in each genome, we predicted the transcriptional units (single genes and operons) with the method of Moreno-Hagelsieb and Collado-Vides [33], which relies on the distribution of distances between genes in a given genome. The set of 250-bp sequences upstream of the first gene of every transcription unit is likely to constitute the smallest set of regulatory regions required for the expression of all genes in a genome and should contain the highest proportion of true functional promoters.

We used random sequences to evaluate if the overrepresentation of promoter-like signals in the regulatory regions is an artifact of AT-richness. We generated the random sequences according to a Markov chain model by using the RANDOM-SEQ program from RSAtools sofware [34]. For each genome, we obtained 1,000 random sequences, 1,000 bp sized, having the same nucleotide composition observed in regulatory regions using a Markov chain of order 0.

### Promoter consensus matrices.

The promoter model we adopted for our searches was Matrix_18_15_13_2_1.5 (Figure 2), defined through a thorough evaluation of more than 200 E. coli matrix pairs that optimized different criteria [1]. Matrices were obtained from a representative set of 116 promoters which have the description of the precise +1 nucleotide of transcription initiation, as well as the information on their regulation. The method used was the following: (i) The 116 sequences were aligned with respect to the position of the transcription initiation (+1). The first 18 bp upstream from the +1 were used as input for WCONSENSUS program [35] to identify the motif corresponding to the −10 conserved region. This size was selected considering that the distance from the −10 hexamer to the +1 varies from 4 to 12 bp. (ii) In order to identify the −35 box, we performed a realignment of the promoter sequences anchoring the −10 boxes identified by the program. New sequences of various lengths, initiating at 13 bp upstream of the −10, were selected. (iii) WCONSENSUS was run with this new set of sequences. Thus, for each one of the −10 alignments, several alternative sequence sizes were analyzed. The best pair, Matrix_18_15_13_2_1.5, was selected on the basis that it contains the canonical consensus sequences for both the −10 and −35 motifs and that it outperformed all others according to measures of sensitivity, specificity, precision, and accuracy.

### Calibration of weight matrices.

The elements of the frequency matrices in Figure 2 are simply the number of times that the indicated letter is observed at the indicated position in the alignment of 116 E. coli promoter sequences. Such elements must be calibrated before matrices can be used in other genomes by normalizing the frequencies with the *a priori* probability for the corresponding base in each genome. To estimate the a priori probabilities, we used the frequency observed for each nucleotide in the set of all noncoding regions for each analyzed genome. Calibrations were done by means of PATSER program [35,36]:

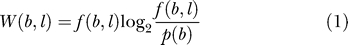
where *f(b,l)* is the relative probability of the base *b* at the position *l* of the input E. coli matrix and *p(b)* is the a priori probability of base *b* in the noncoding regions of the analyzed genome.

### Scoring a DNA promoter site.

In order to define which sequences would be considered promoter-like signals in the different target genomes, we determined minimal cutoff scores that would retain 98% of the 116 original motifs from functional σ^70^ promoters that were used in generating the E. coli frequency matrices. With this aim, PATSER program was used to rescore the original E. coli motifs with the calibrated matrices corresponding to each genome. The mean and standard deviation of the new scores were obtained. In most of the analyzed genomes, 98% of the original motifs was retained using a cutoff of μ − 2.5σ. PATSER calculates the score, *I_seq_*, of a target motif of size *L*, as:

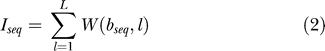
where *b_seq_* is the base found in the *l* position of the target motif. The score of a promoter is the sum of scores corresponding to the −10 and −35 motifs.

### Counting promoter-like signals.

For each genome, the calibrated matrices were used to search for promoter-like signals in the four kinds of genomic regions (random, coding, convergent, and strictly noncoding). Only those signals scoring above the respective cutoff were retained and the number of promoter-like signals found in each genomic region was calculated. The size, in base pairs, of each genomic region was obtained. To conduct the analysis of underrepresentation/overrepresentation, we chose to compare the distribution of the promoter-like signals found in the strictly noncoding regions against that found in convergent regions. Before comparison, the number of detected signals was adjusted to the size of the smallest genomic region. A log-likelihood statistic was used for the calculated proportions to determine if the genome had an excess density of promoter-like signals in potentially regulatory regions (the strictly noncoding regions) when compared against noncoding regions between convergent genes.

For genomes with significant overrepresentation of promoter-like signals in regulatory versus convergent noncoding regions (*p* < 0.001), we estimated the most likely functional promoters in the genome. To this aim, we ran searches for promoter-like signals in the 250-bp upstream regions of the operons in each genome (see section on sequence data). We applied the COVER function (described below) on the collections of promoter-like signals found in the set of regulatory regions. The resulting COVER-predicted promoters in these regulatory regions were taken as the most likely functional promoters in that genome.

### Cover function.

The COVER program [1] is used to choose the most likely functional promoters from a conglomerate of promoter-like signals identified by weight matrices. First, COVER employs a ‘‘divide and conquer'' strategy: Each promoter-like signal is assigned to a class based on the length of the spacer region between the −10 and −35 boxes. Then, the selection of the best signals within each class is based on a well-known partial order relation, the *inclusion relation* [37]. In our case, the relation uses both an “intrinsic score,” the score resulting from adding the −10 and the −35 scores, and an “external score,” based on the relative position of the predicted −10 in relation to the beginning of the gene. A promoter A is said to be included by another promoter B if, and only if, the scores of promoter B are both better than the corresponding scores of promoter A. This inclusion relation defines a “cover set”, or collection of separate subsets, for each spacer class. Within each class, the predicted functional promoter is defined as the upper border of the subset with the highest cardinality, i.e., the one which includes the highest number of promoter signals. The signals detected by COVER are mostly found grouped into clusters.

### Conservation of promoter-like signals in related bacteria.

We have obtained 2,014 groups of orthologous genes from 14 enteric genomes that showed overrepresentation of promoter-like signals in the regulatory regions. The average size of the groups of orthologs was 10.4 genes. For all the promoter-like signals found by using weight matrices, we have measured the conservation of each promoter-like signal as the number of times that the signal is present in the same relative position in each upstream region for each set of orthologs. We report 79% signal conservation. Orthologs were kindly provided by G. Moreno-Hagelsieb and consisted of BLASTP reciprocal best hits obtained as explained previously in [33].

Graphs S1 and S2 in Supplementary Material were created using the XYGRAPH program from the RSAtools software [34]. Scripts were written in Perl [38].

## Supporting Information

Figure S1 Signal Density in Regulatory versus Nonregulatory Regions for All Analyzed Eubacterial Genomes (5.4 MB PDF)

Figure S2 Signal Density in Random Regulatory versus Regulatory Regions for All Analyzed Eubacterial Genomes (493 KB PDF)

Table S1 Detailed Examples of M. leprae Pseudogenes with Orthologous Genes in M. tuberculosis
(15 KB PDF)

### Accession Numbers

The RegulonDB (http://regulondb.ccg.unam.mx/index.html) accession number for the regulatory region of the *E. coli lac* operon is ECK120014850 and that for *gal* gene is ECK120014842.

The National Center for Biotechnology Information (NCBI) (http://www.ncbi.nlm.nih.gov) accession numbers for representative bacterial genomes are given in Table 1.

## Abbreviations

bp: base pair(s)
PWM: position weight matrix
RNAP: RNA polymerase
