# Supplementary material for: Selection for Unequal Densities of σ70 Promoter-Like Signals in Different Regions of Large Bacterial Genomes
Source: PLoS Genet. 2006 Nov 10;2(11):e185. doi: 10.1371/journal.pgen.0020185 (PMC1635534; doi:10.1371/journal.pgen.0020185)
Supplement: Supplementary file 1 [file pgen.0020185.sg001.pdf]

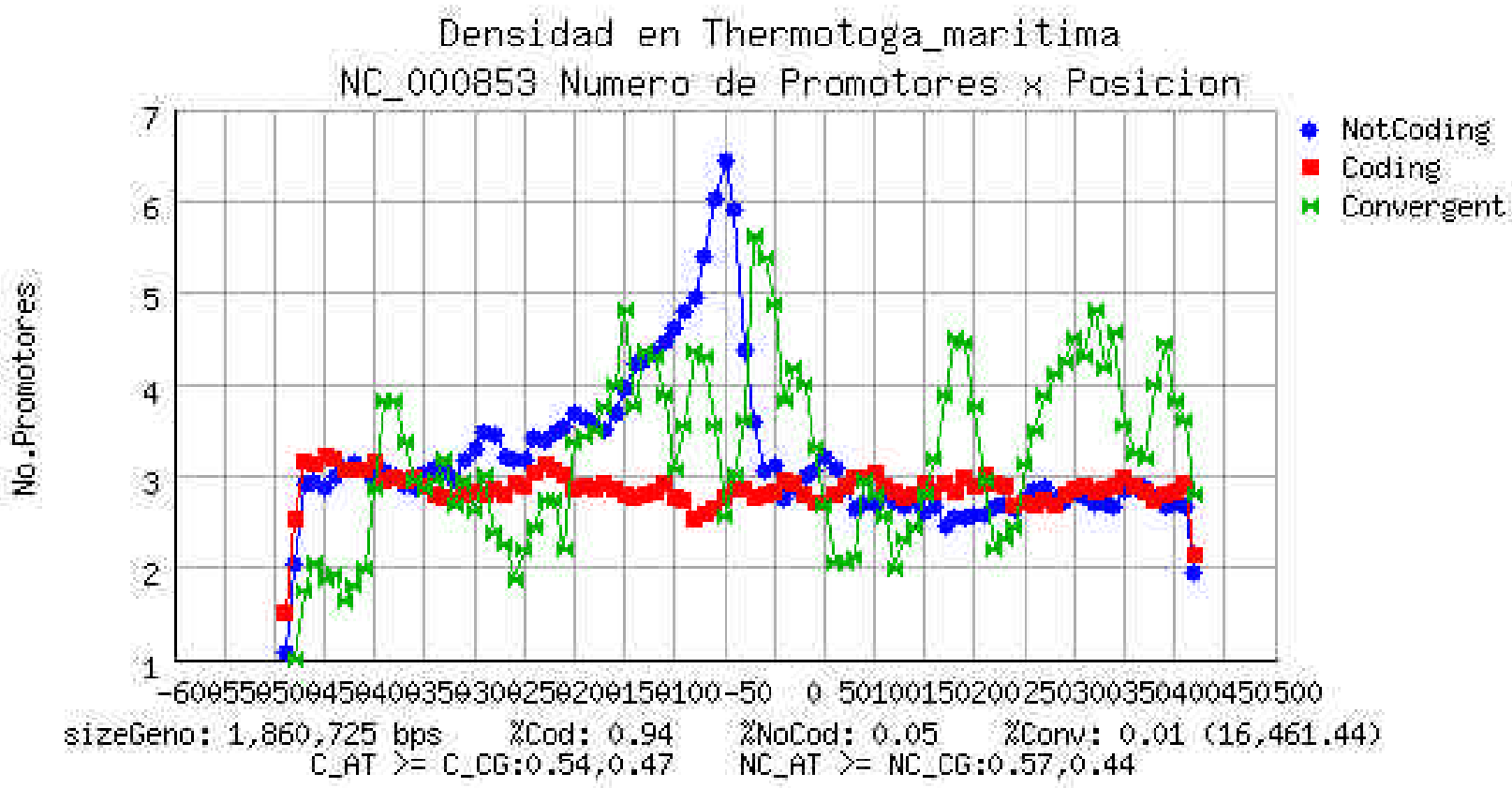
Densidad en Thermotoga_maritima
NC_000853 Numero de Promotores x Posicion
No.Promotores
NotCoding
Coding
Convergent
sizeGeno: 1,860,725 bps %Cod: 0.94 %NoCod: 0.05 %Conv: 0.01 (16,461.44)
C_AT >= C_CG:0.54,0.47 NC_AT >= NC_CG:0.57,0.44

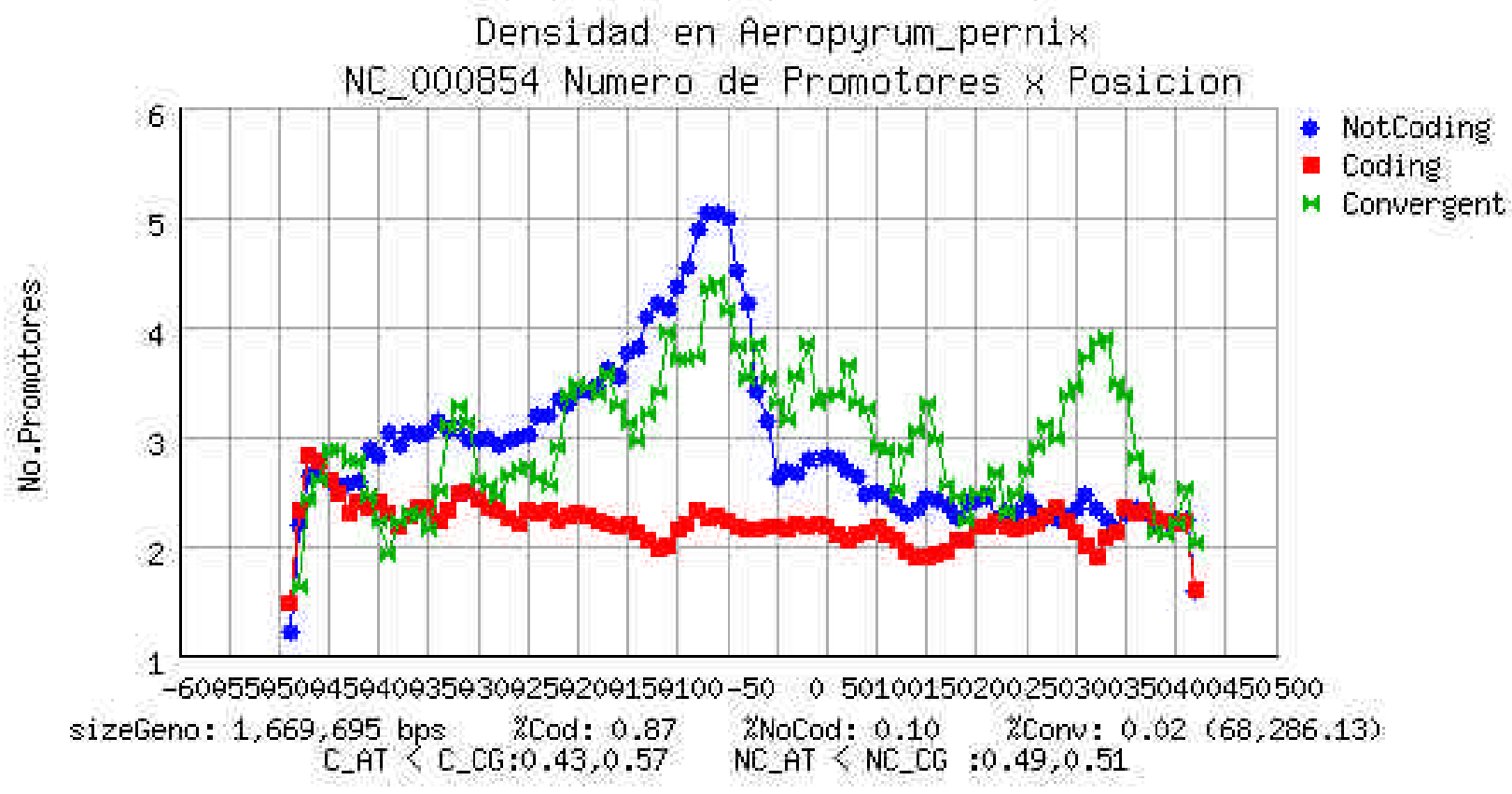
Densidad en Aeropyrum_pernix
NC_000854 Numero de Promotores x Posicion
No.Promotores
NotCoding
Coding
Convergent
sizeGeno: 1,669,695 bps %Cod: 0.87 %NoCod: 0.10 %Conv: 0.02 (68,286.13)
C_AT < C_CG:0.43,0.57 NC_AT < NC_CG :0.49,0.51

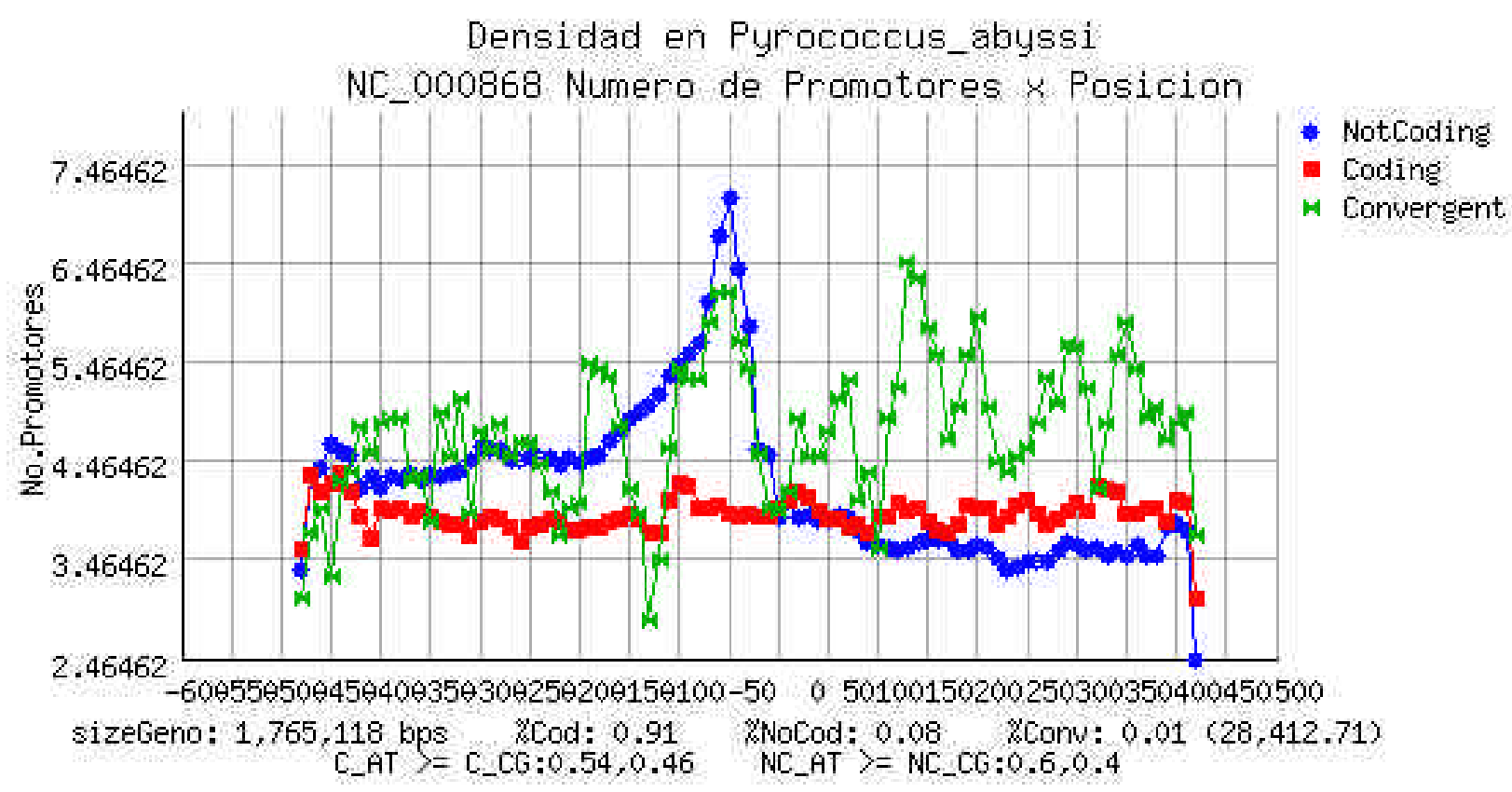
Densidad en Pyrococcus_abyssi
NC_000868 Numero de Promotores x Posicion
No.Promotores
NotCoding
Coding
Convergent
sizeGeno: 1,765,118 bps %Cod: 0.91 %NoCod: 0.08 %Conv: 0.01 (28,412.71)
C_AT >= C_CG:0.54,0.46 NC_AT >= NC_CG:0.6,0.4

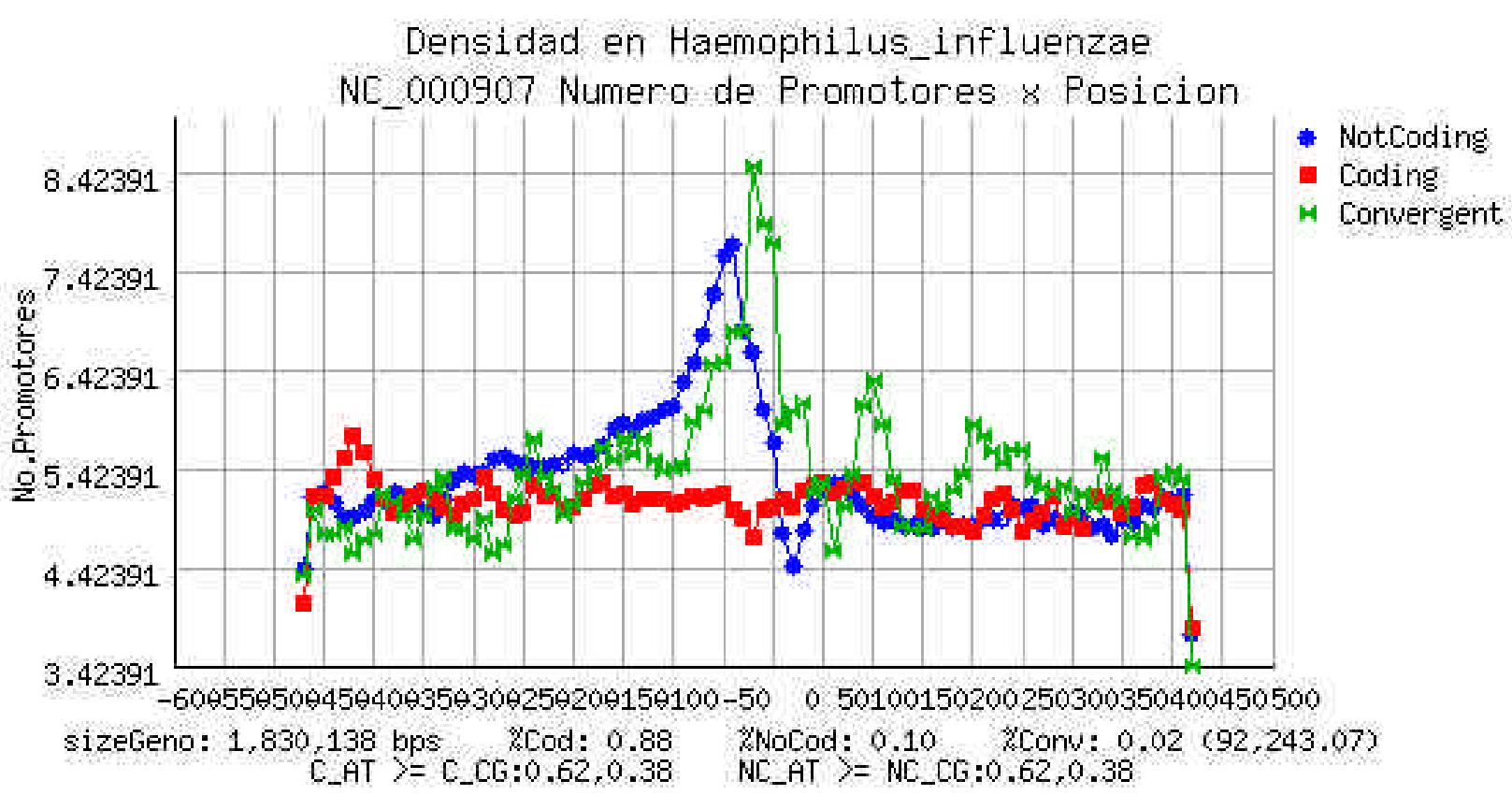
Densidad en Haemophilus_influenzae
NC_000907 Numero de Promotores x Posicion
No.Promotores
NotCoding
Coding
Convergent
sizeGeno: 1,830,138 bps %Cod: 0.88 %NoCod: 0.10 %Conv: 0.02 (92,243.07)
C_AT >= C_CG:0.62,0.38 NC_AT >= NC_CG:0.62,0.38

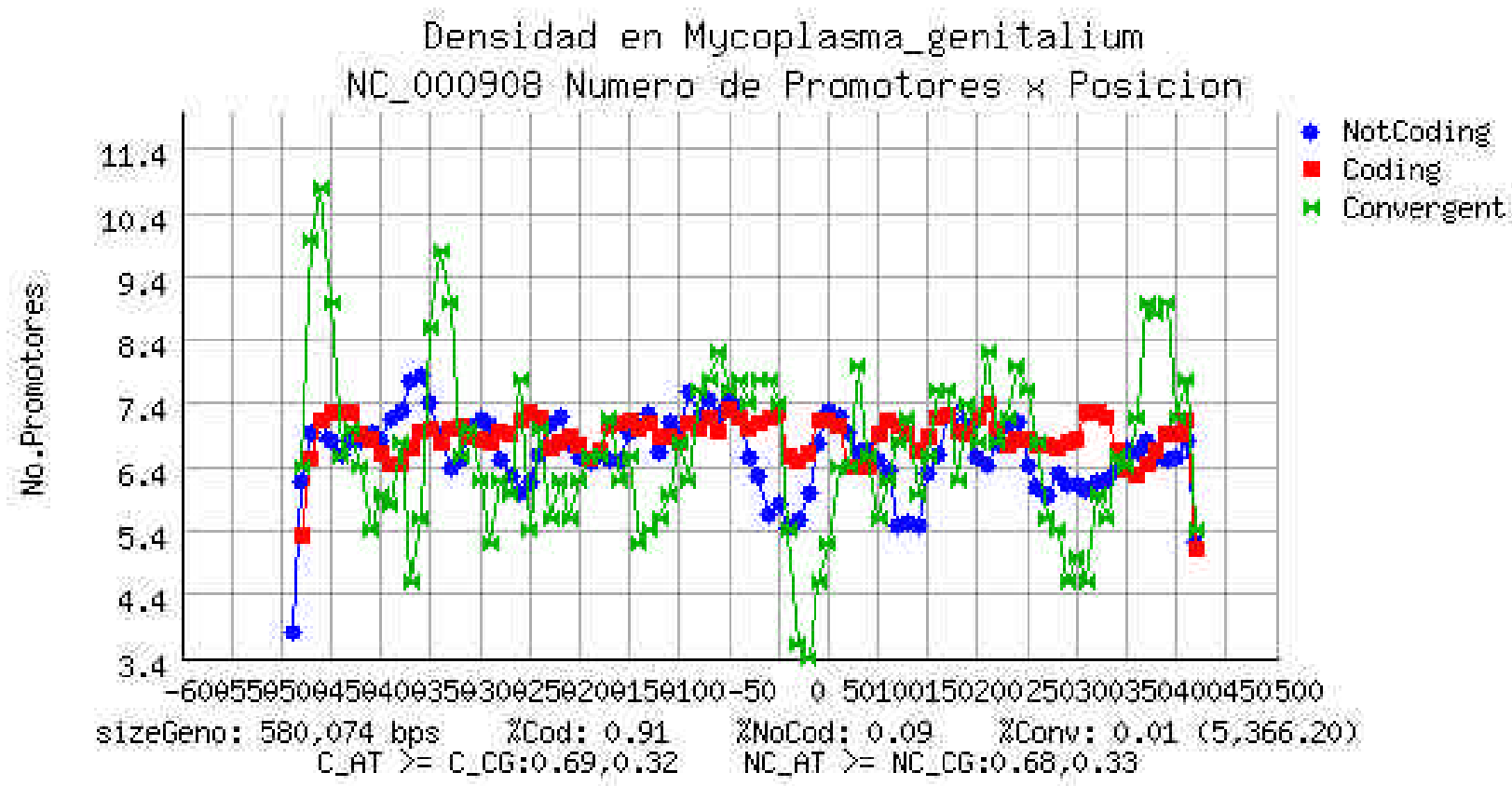
Densidad en Mycoplasma_genitalium
NC_000908 Numero de Promotores x Posicion
No.Promotores
11.4
10.4
9.4
8.4
7.4
6.4
5.4
4.4
3.4
-600550500450400350300250200150100-50 0 50100150200250300350400450500
NotCoding
Coding
Convergent
sizeGeno: 580,074 bps %Cod: 0.91 %NoCod: 0.09 %Conv: 0.01 (5,366.20)
C_AT >= C_CG:0.69,0.32 NC_AT >= NC_CG:0.68,0.33

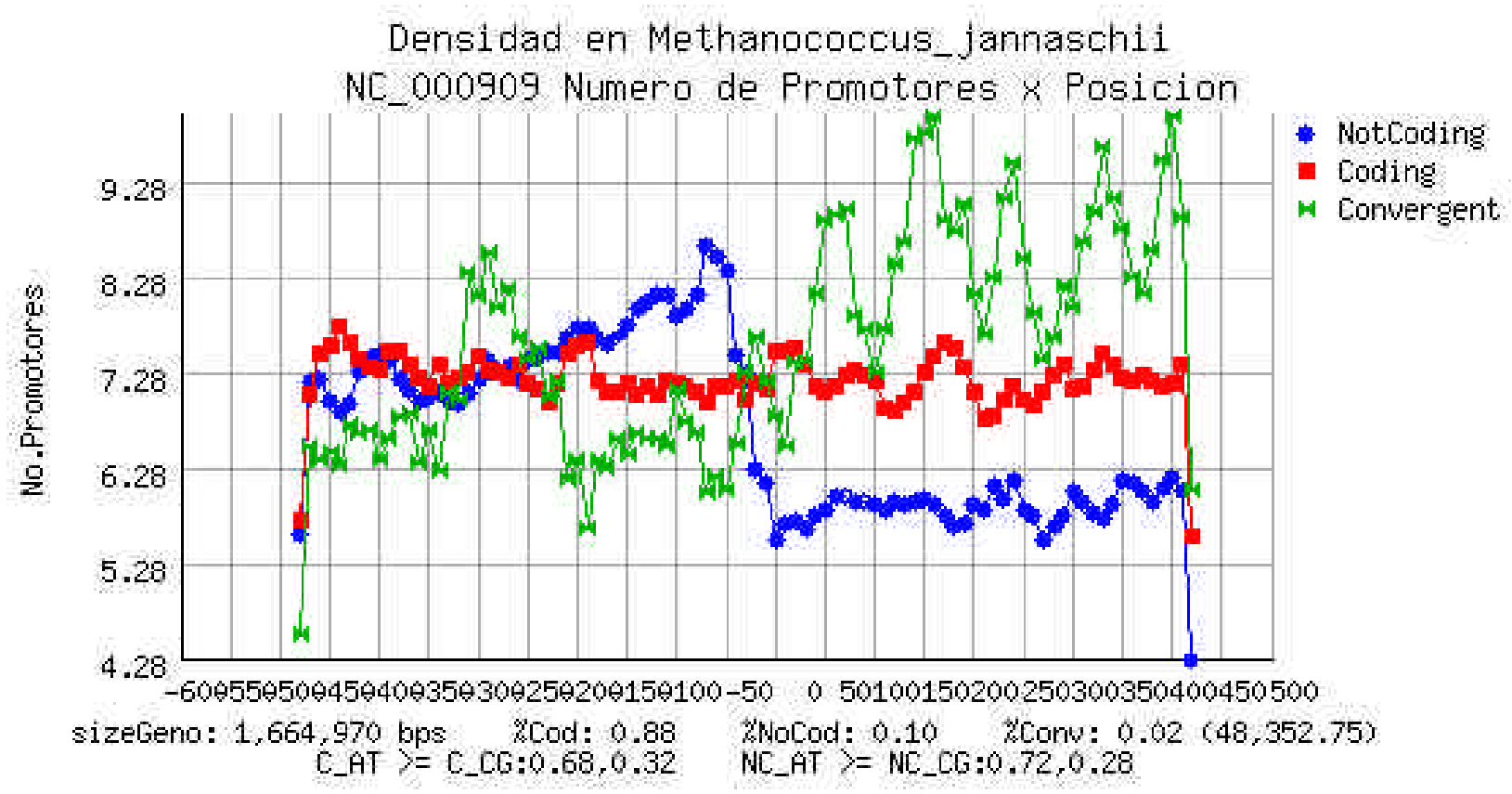
Densidad en Methanococcus_jannaschii
NC_000909 Numero de Promotores x Posicion
No.Promotores
9.28
8.28
7.28
6.28
5.28
4.28
-600550500450400350300250200150100-50 0 50100150200250300350400450500
NotCoding
Coding
Convergent
sizeGeno: 1,664,970 bps %Cod: 0.88 %NoCod: 0.10 %Conv: 0.02 (48,352.75)
C_AT >= C_CG:0.68,0.32 NC_AT >= NC_CG:0.72,0.28

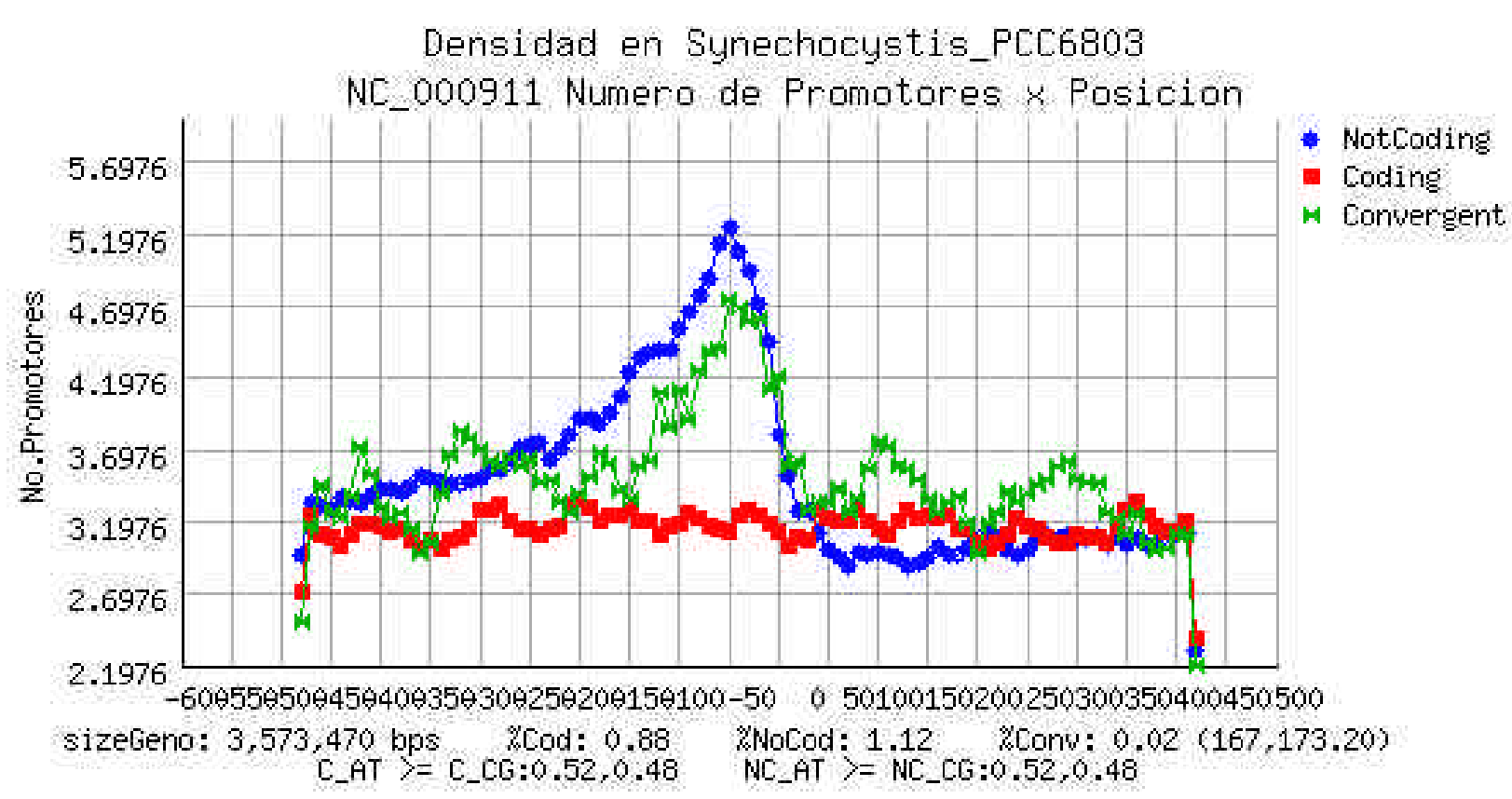
Densidad en Synechocystis_PCC6803
NC_000911 Numero de Promotores x Posicion
No.Promotores
5.6976
5.1976
4.6976
4.1976
3.6976
3.1976
2.6976
2.1976
-600550500450400350300250200150100-50 0 50100150200250300350400450500
NotCoding
Coding
Convergent
sizeGeno: 3,573,470 bps %Cod: 0.88 %NoCod: 1.12 %Conv: 0.02 (167,173.20)
C_AT >= C_CG:0.52,0.48 NC_AT >= NC_CG:0.52,0.48

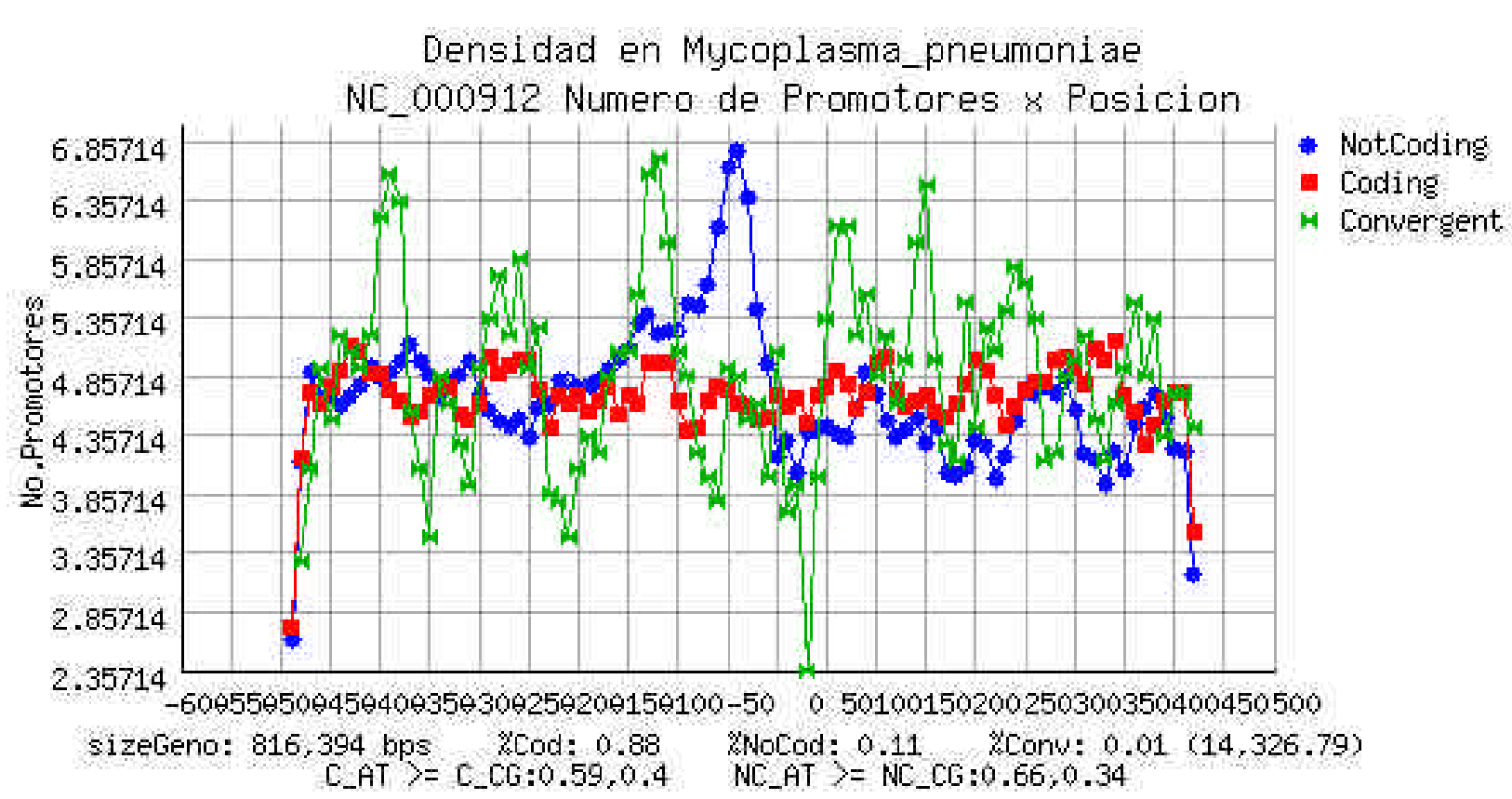
Densidad en Mycoplasma_pneumoniae
NC_000912 Numero de Promotores x Posicion
No.Promotores
6.85714
6.35714
5.85714
5.35714
4.85714
4.35714
3.85714
3.35714
2.85714
2.35714
-600550500450400350300250200150100-50 0 50100150200250300350400450500
NotCoding
Coding
Convergent
sizeGeno: 816,394 bps %Cod: 0.88 %NoCod: 0.11 %Conv: 0.01 (14,326.79)
C_AT >= C_CG:0.59,0.4 NC_AT >= NC_CG:0.66,0.34

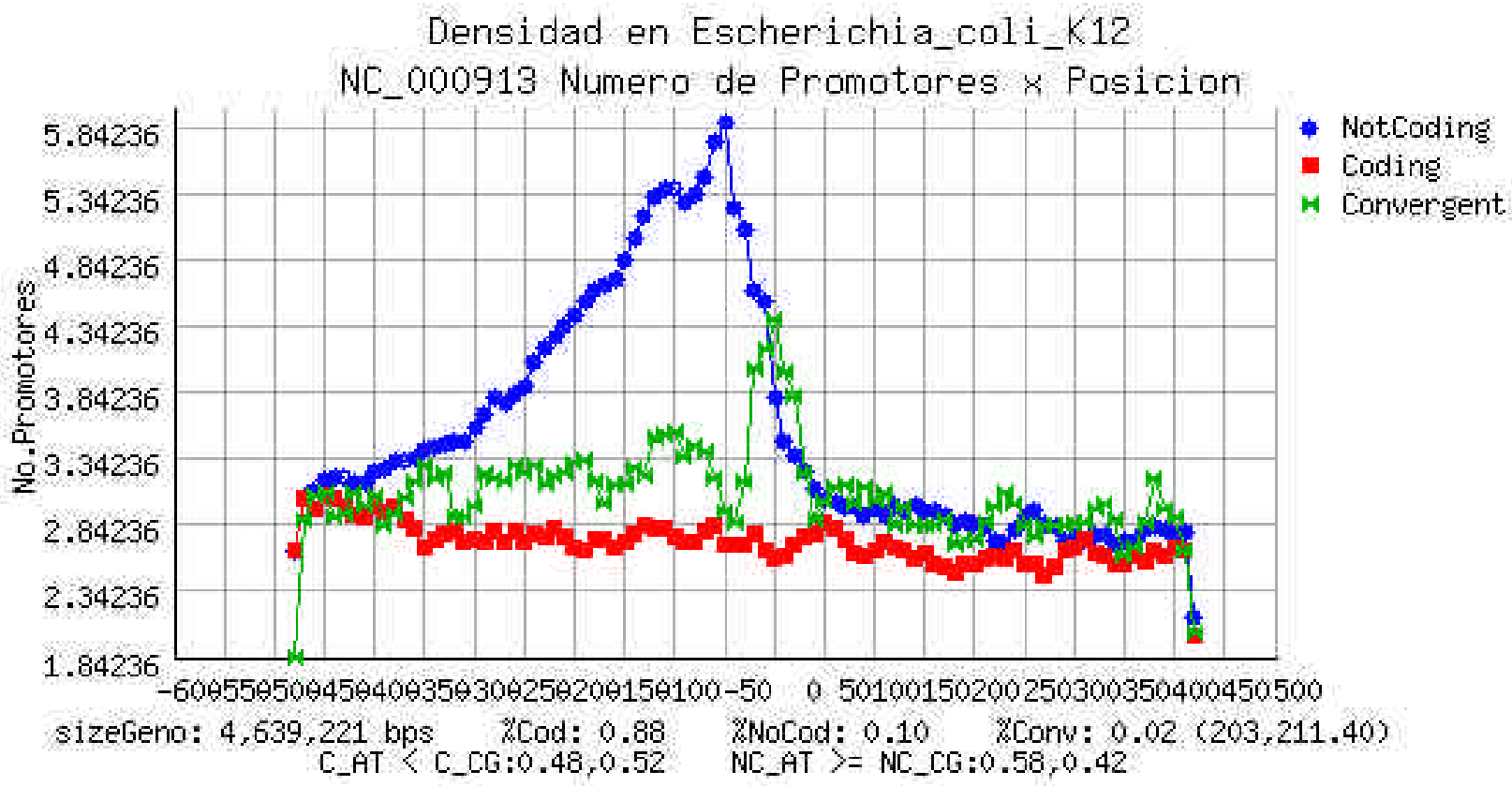
Densidad en Escherichia_coli_K12
NC_000913 Numero de Promotores x Posicion
NotCoding
Coding
Convergent
No.Promotores
5.84236
5.34236
4.84236
4.34236
3.84236
3.34236
2.84236
2.34236
1.84236
-600550500450400350300250200150100-50 0 50100150200250300350400450500
sizeGeno: 4,639,221 bps %Cod: 0.88 %NoCod: 0.10 %Conv: 0.02 (203,211.40)
C_AT < C_CG:0.48,0.52 NC_AT >= NC_CG:0.58,0.42

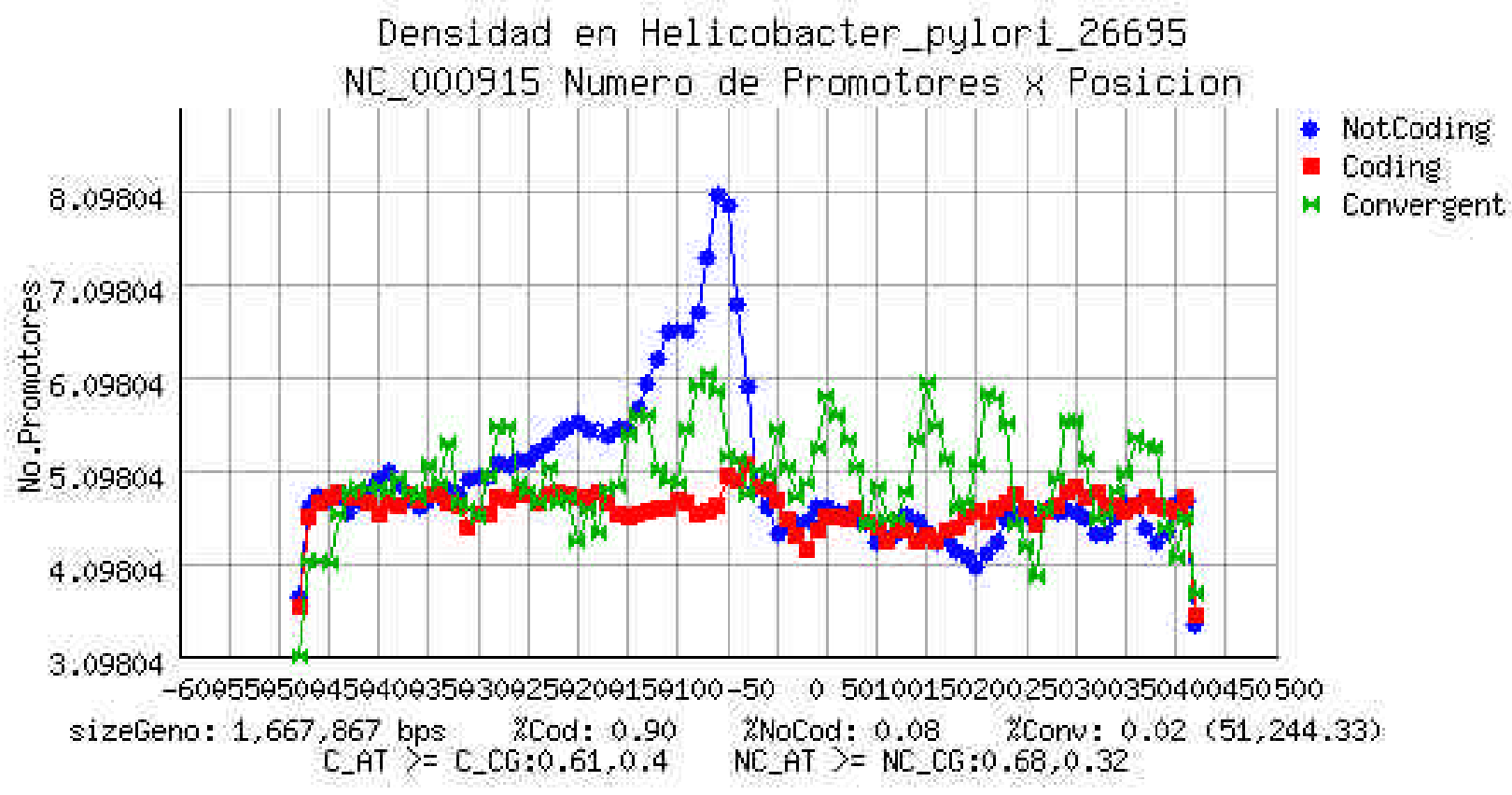
Densidad en Helicobacter_pylori_26695
NC_000915 Numero de Promotores x Posicion
NotCoding
Coding
Convergent
No.Promotores
8.09804
7.09804
6.09804
5.09804
4.09804
3.09804
-600550500450400350300250200150100-50 0 50100150200250300350400450500
sizeGeno: 1,667,867 bps %Cod: 0.90 %NoCod: 0.08 %Conv: 0.02 (51,244.33)
C_AT >= C_CG:0.61,0.4 NC_AT >= NC_CG:0.68,0.32

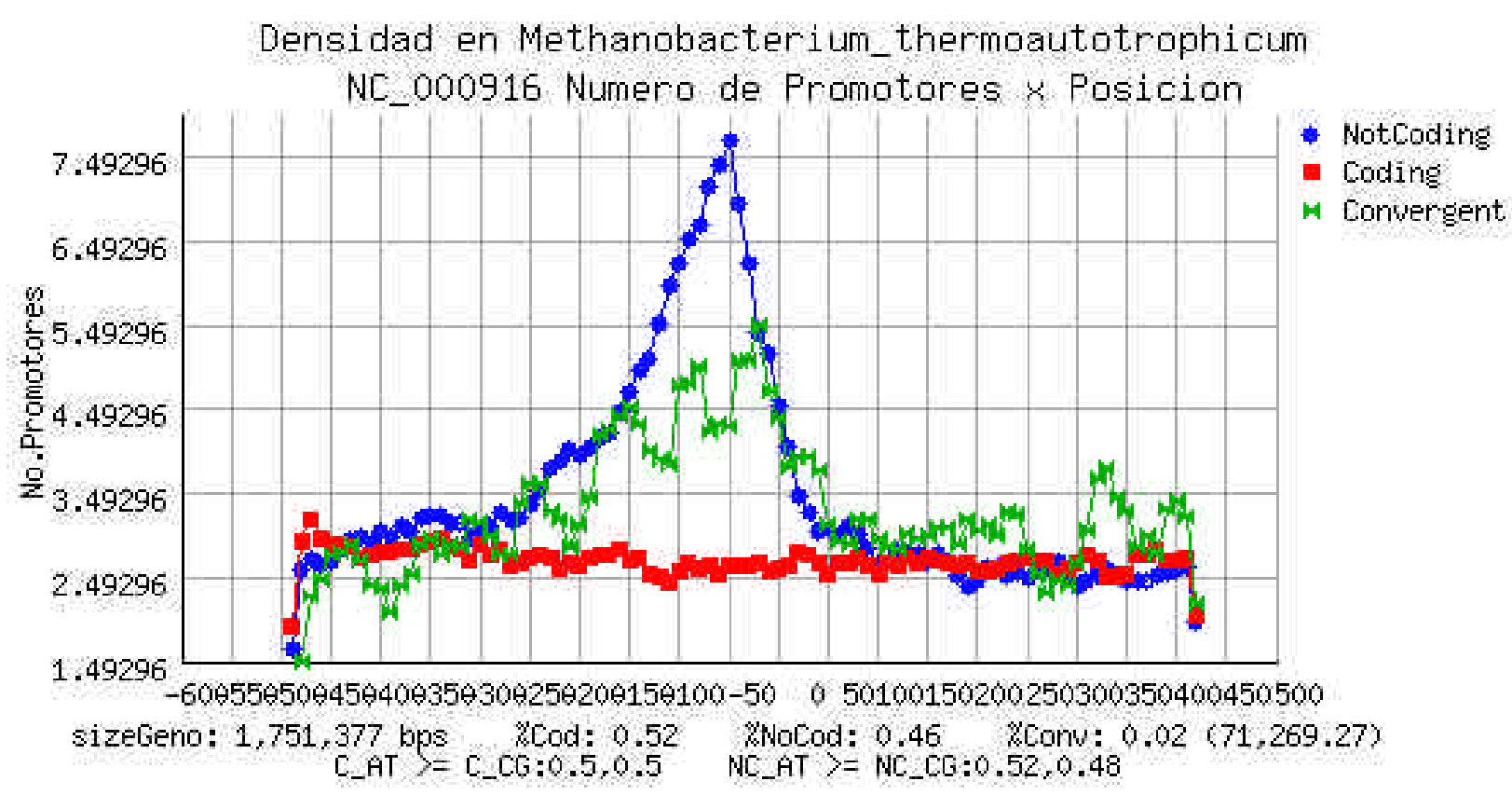
Densidad en Methanobacterium_thermoautotrophicum
NC_000916 Numero de Promotores x Posicion
NotCoding
Coding
Convergent
No.Promotores
7.49296
6.49296
5.49296
4.49296
3.49296
2.49296
1.49296
-600550500450400350300250200150100-50 0 50100150200250300350400450500
sizeGeno: 1,751,377 bps %Cod: 0.52 %NoCod: 0.46 %Conv: 0.02 (71,269.27)
C_AT >= C_CG:0.5,0.5 NC_AT >= NC_CG:0.52,0.48

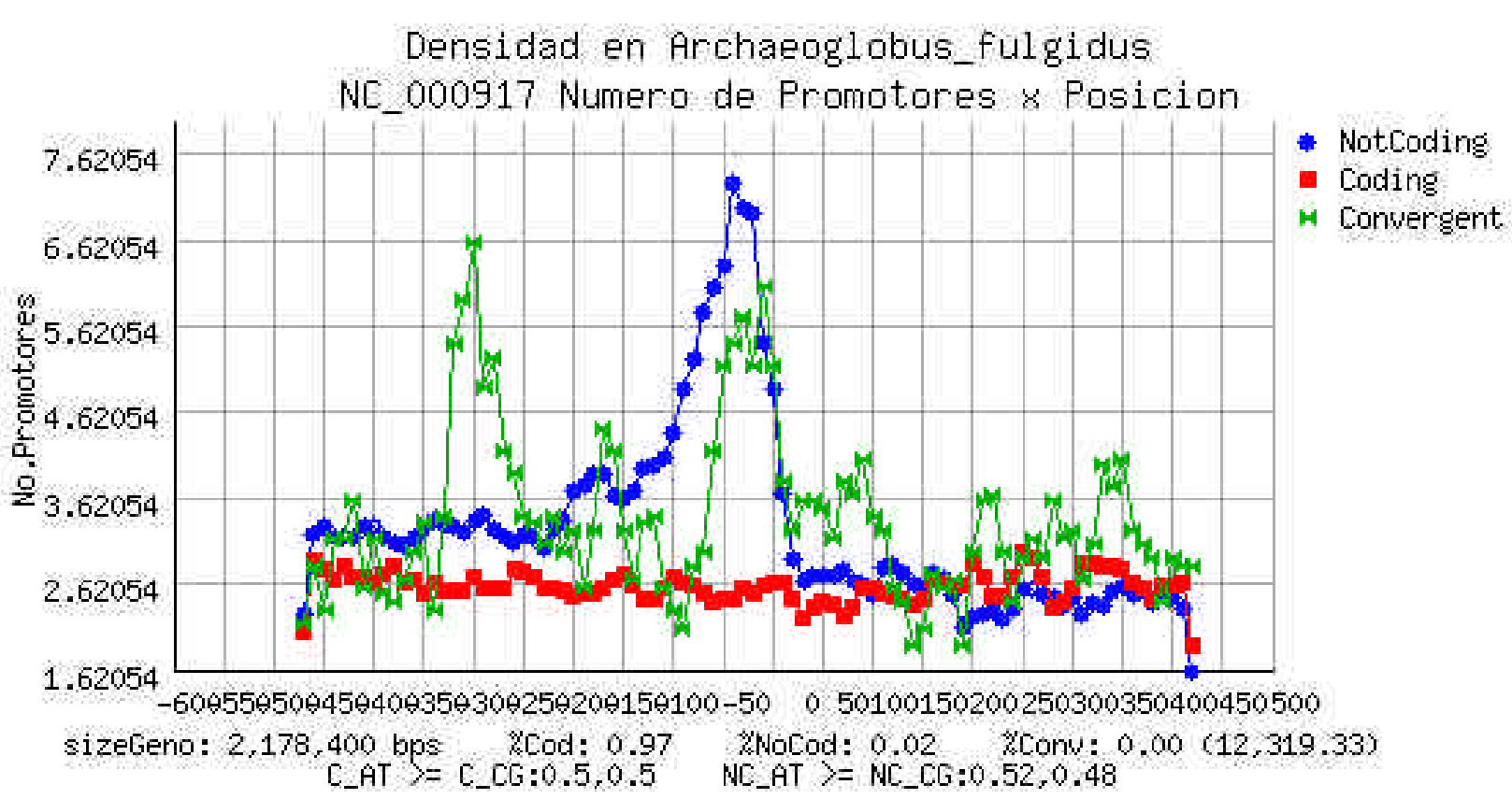
Densidad en Archaeoglobus_fulgidus
NC_000917 Numero de Promotores x Posicion
NotCoding
Coding
Convergent
No.Promotores
7.62054
6.62054
5.62054
4.62054
3.62054
2.62054
1.62054
-600550500450400350300250200150100-50 0 50100150200250300350400450500
sizeGeno: 2,178,400 bps %Cod: 0.97 %NoCod: 0.02 %Conv: 0.00 (12,319.33)
C_AT >= C_CG:0.5,0.5 NC_AT >= NC_CG:0.52,0.48

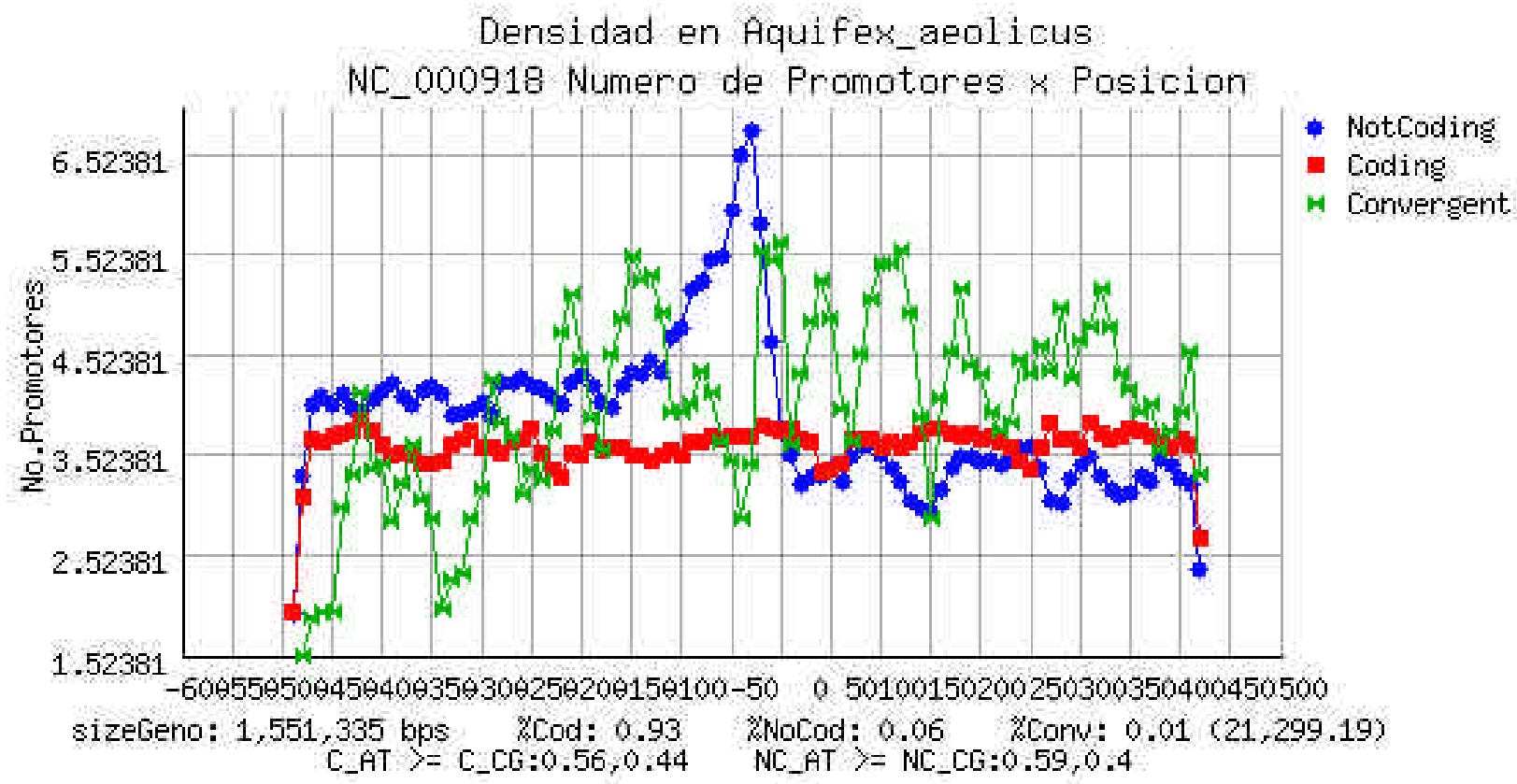
Densidad en Aquifex_aeolicus
NC_000918 Numero de Promotores x Posicion
NotCoding
Coding
Convergent
No.Promotores
sizeGeno: 1,551,335 bps %Cod: 0.93 %NoCod: 0.06 %Conv: 0.01 (21,299.19)
C_AT >= C_CG:0.56,0.44 NC_AT >= NC_CG:0.59,0.4

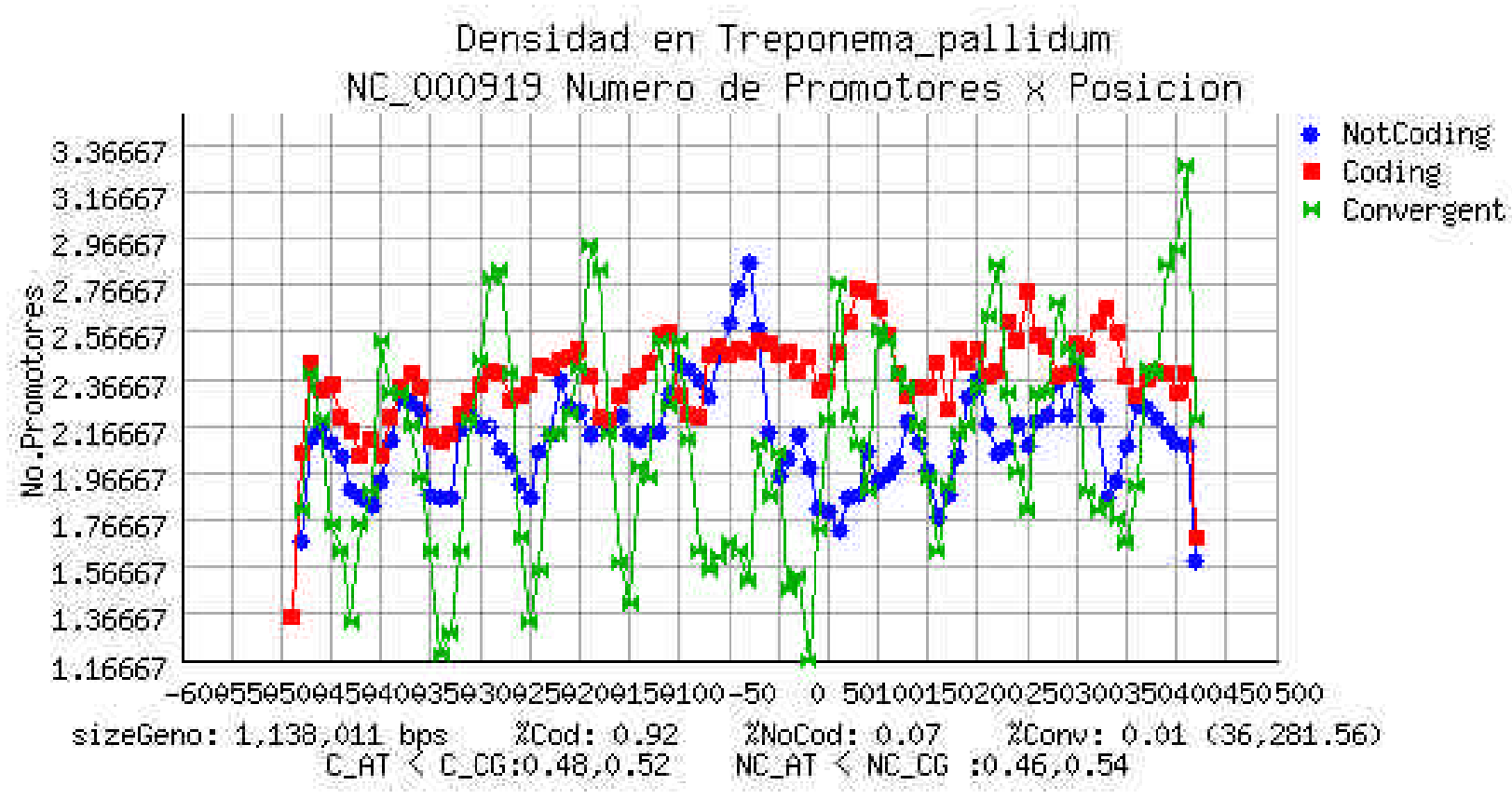
Densidad en Treponema_pallidum
NC_000919 Numero de Promotores x Posicion
NotCoding
Coding
Convergent
No.Promotores
sizeGeno: 1,138,011 bps %Cod: 0.92 %NoCod: 0.07 %Conv: 0.01 (36,281.56)
C_AT < C_CG:0.48,0.52 NC_AT < NC_CG :0.46,0.54

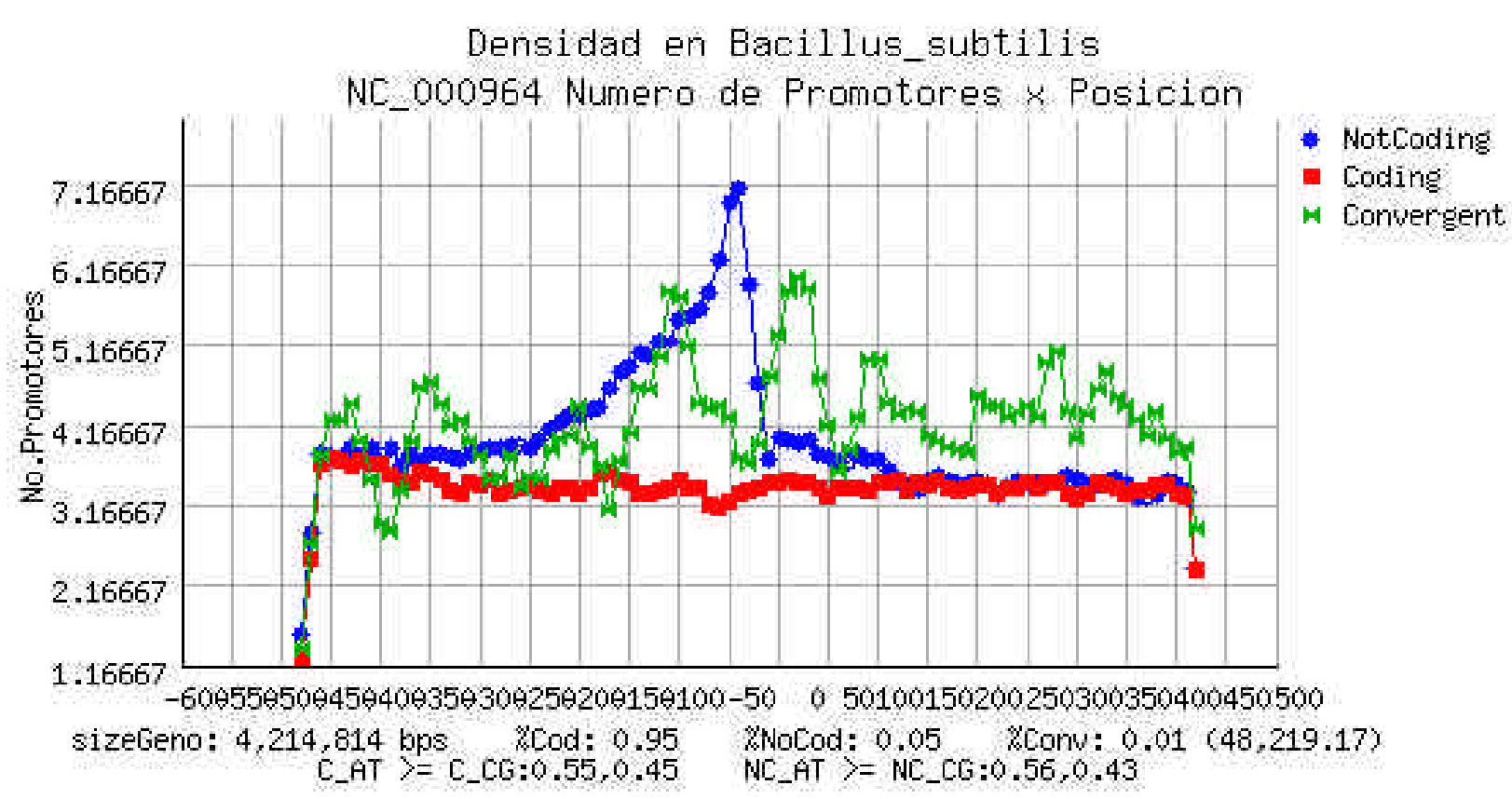
Densidad en Bacillus_subtilis
NC_000964 Numero de Promotores x Posicion
NotCoding
Coding
Convergent
No.Promotores
sizeGeno: 4,214,814 bps %Cod: 0.95 %NoCod: 0.05 %Conv: 0.01 (48,219.17)
C_AT >= C_CG:0.55,0.45 NC_AT >= NC_CG:0.56,0.43

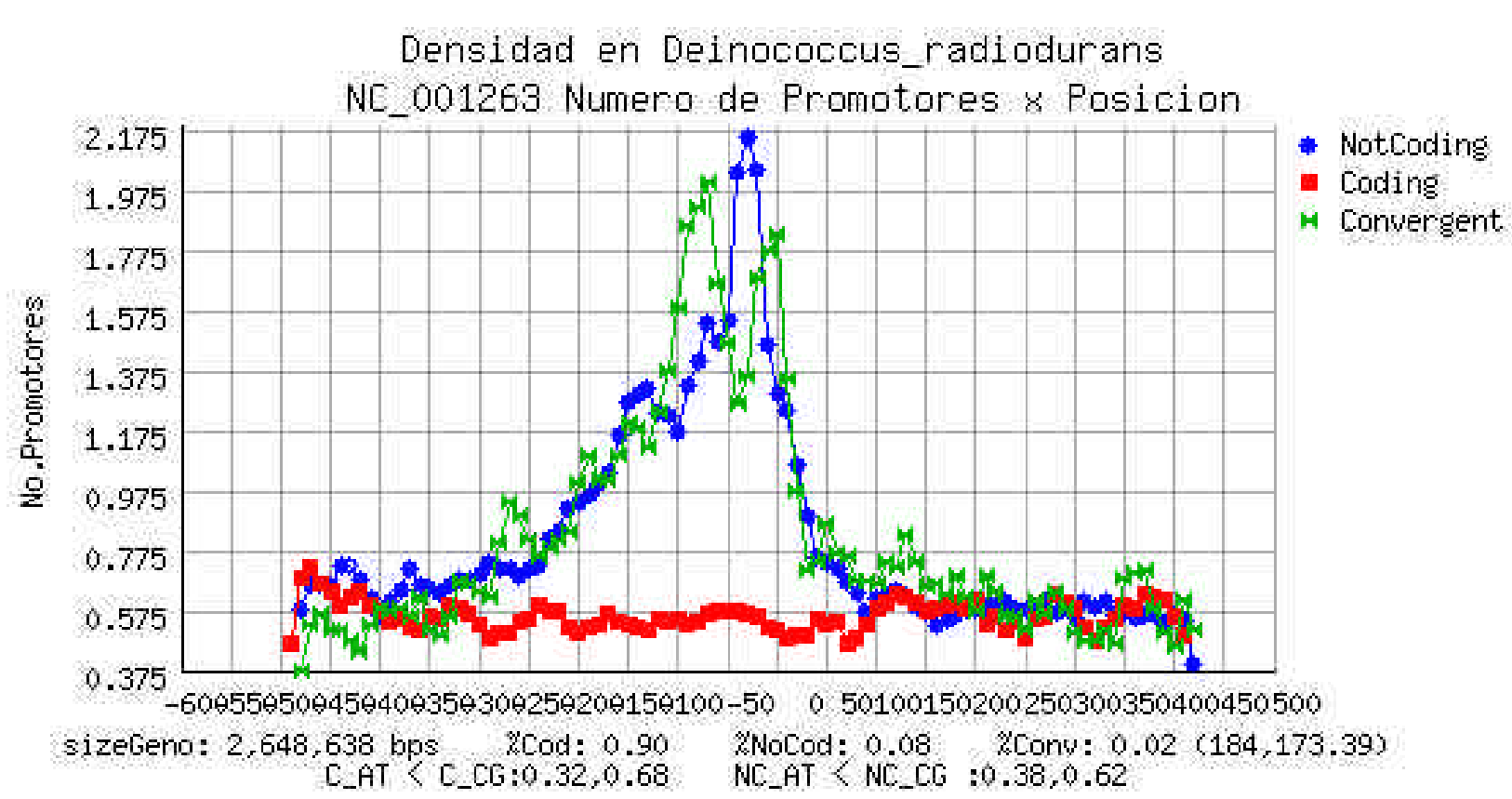
Densidad en Deinococcus_radiodurans
NC_001263 Numero de Promotores x Posicion
NotCoding
Coding
Convergent
No.Promotores
sizeGeno: 2,648,638 bps %Cod: 0.90 %NoCod: 0.08 %Conv: 0.02 (184,173.39)
C_AT < C_CG:0.32,0.68 NC_AT < NC_CG :0.38,0.62

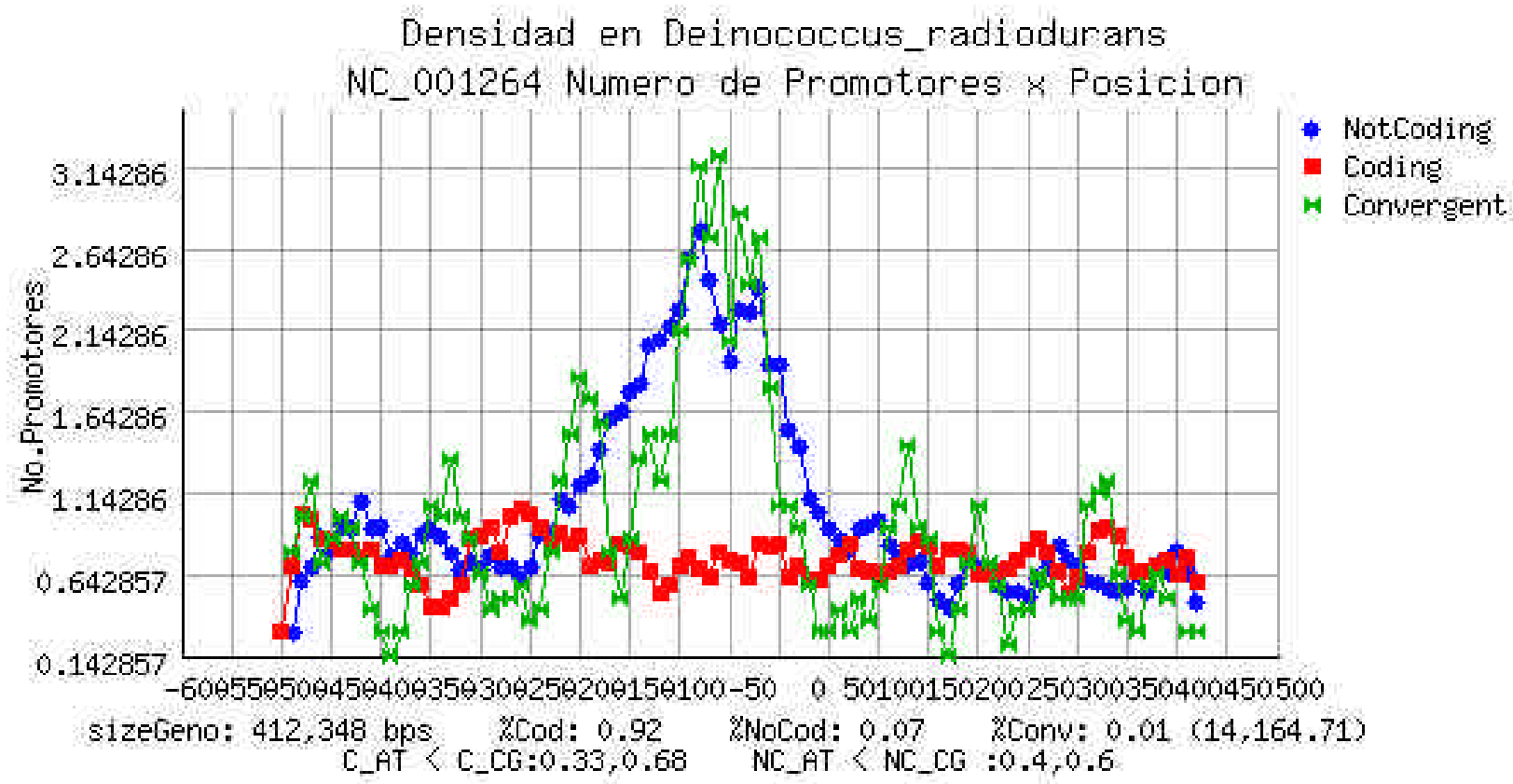
Densidad en Deinococcus_radiodurans
NC_001264 Numero de Promotores x Posicion
NotCoding
Coding
Convergent
No.Promotores
3.14286
2.64286
2.14286
1.64286
1.14286
0.642857
0.142857
-600550500450400350300250200150100-50 0 50100150200250300350400450500
sizeGeno: 412,348 bps %Cod: 0.92 %NoCod: 0.07 %Conv: 0.01 (14,164.71)
C_AT < C_CG:0.33,0.68 NC_AT < NC_CG :0.4,0.6

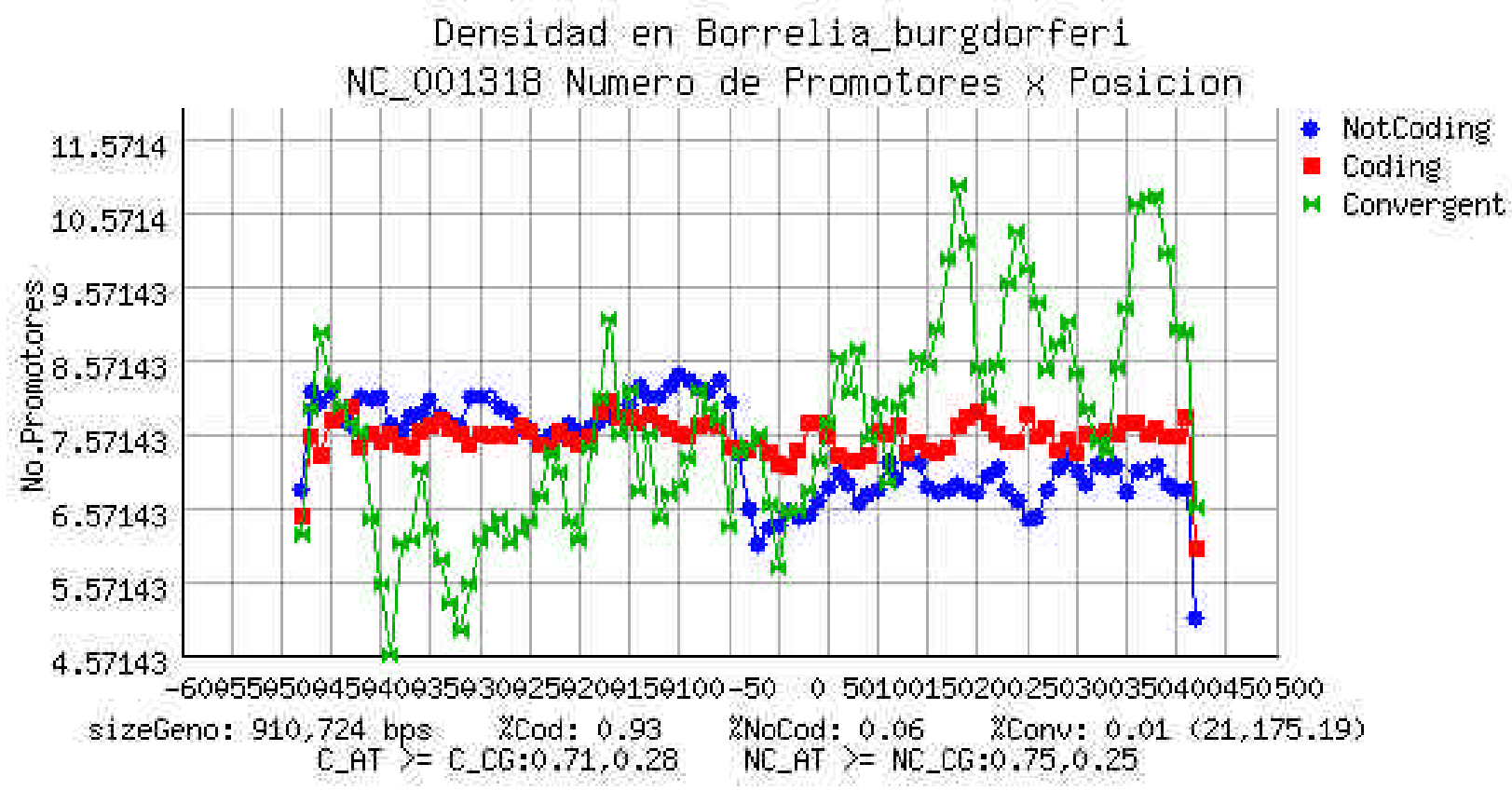
Densidad en Borrelia_burgdorferi
NC_001318 Numero de Promotores x Posicion
NotCoding
Coding
Convergent
No.Promotores
11.5714
10.5714
9.57143
8.57143
7.57143
6.57143
5.57143
4.57143
-600550500450400350300250200150100-50 0 50100150200250300350400450500
sizeGeno: 910,724 bps %Cod: 0.93 %NoCod: 0.06 %Conv: 0.01 (21,175.19)
C_AT >= C_CG:0.71,0.28 NC_AT >= NC_CG:0.75,0.25

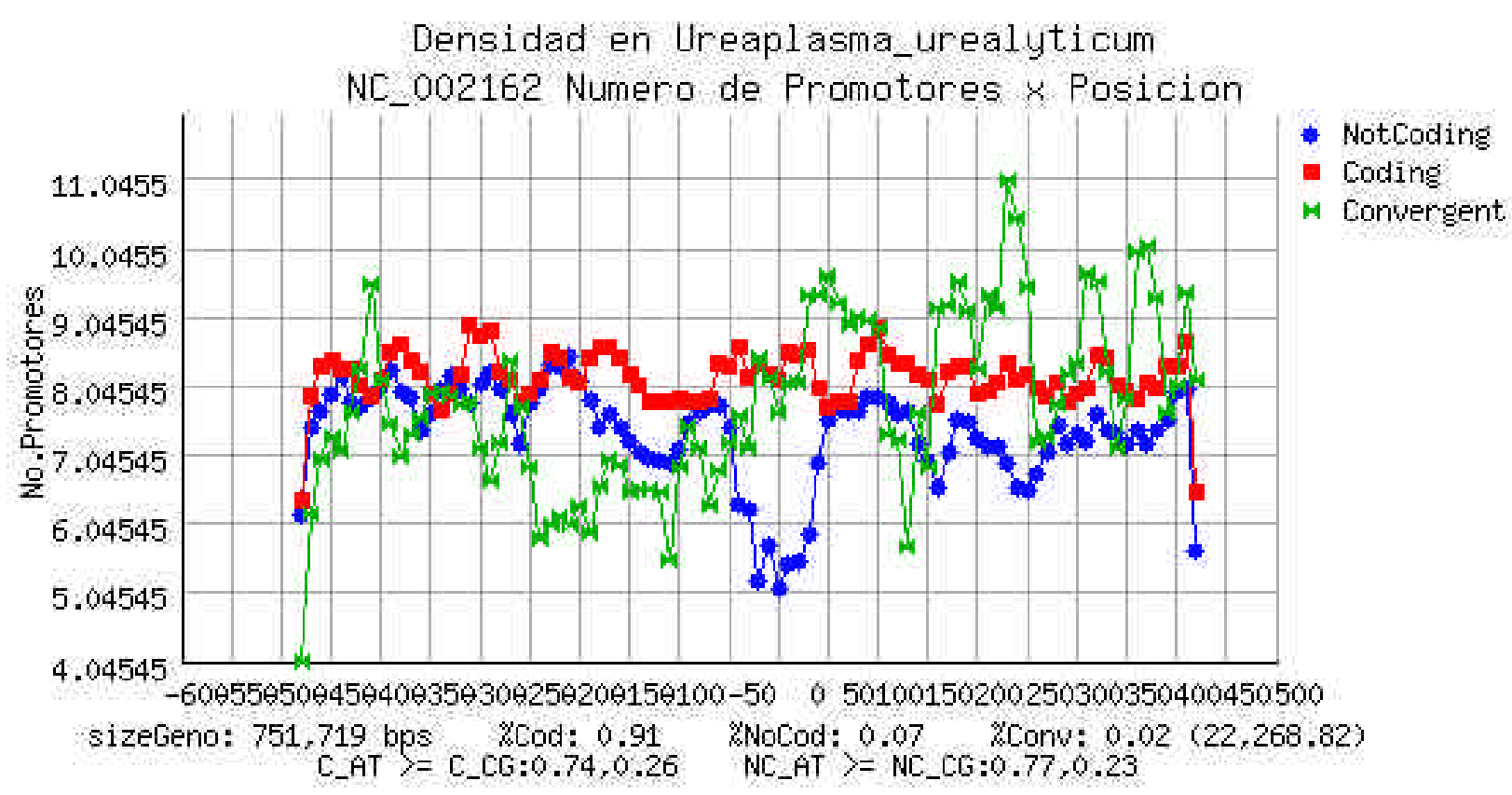
Densidad en Ureaplasma_urealyticum
NC_002162 Numero de Promotores x Posicion
NotCoding
Coding
Convergent
No.Promotores
11.0455
10.0455
9.04545
8.04545
7.04545
6.04545
5.04545
4.04545
-600550500450400350300250200150100-50 0 50100150200250300350400450500
sizeGeno: 751,719 bps %Cod: 0.91 %NoCod: 0.07 %Conv: 0.02 (22,268.82)
C_AT >= C_CG:0.74,0.26 NC_AT >= NC_CG:0.77,0.23

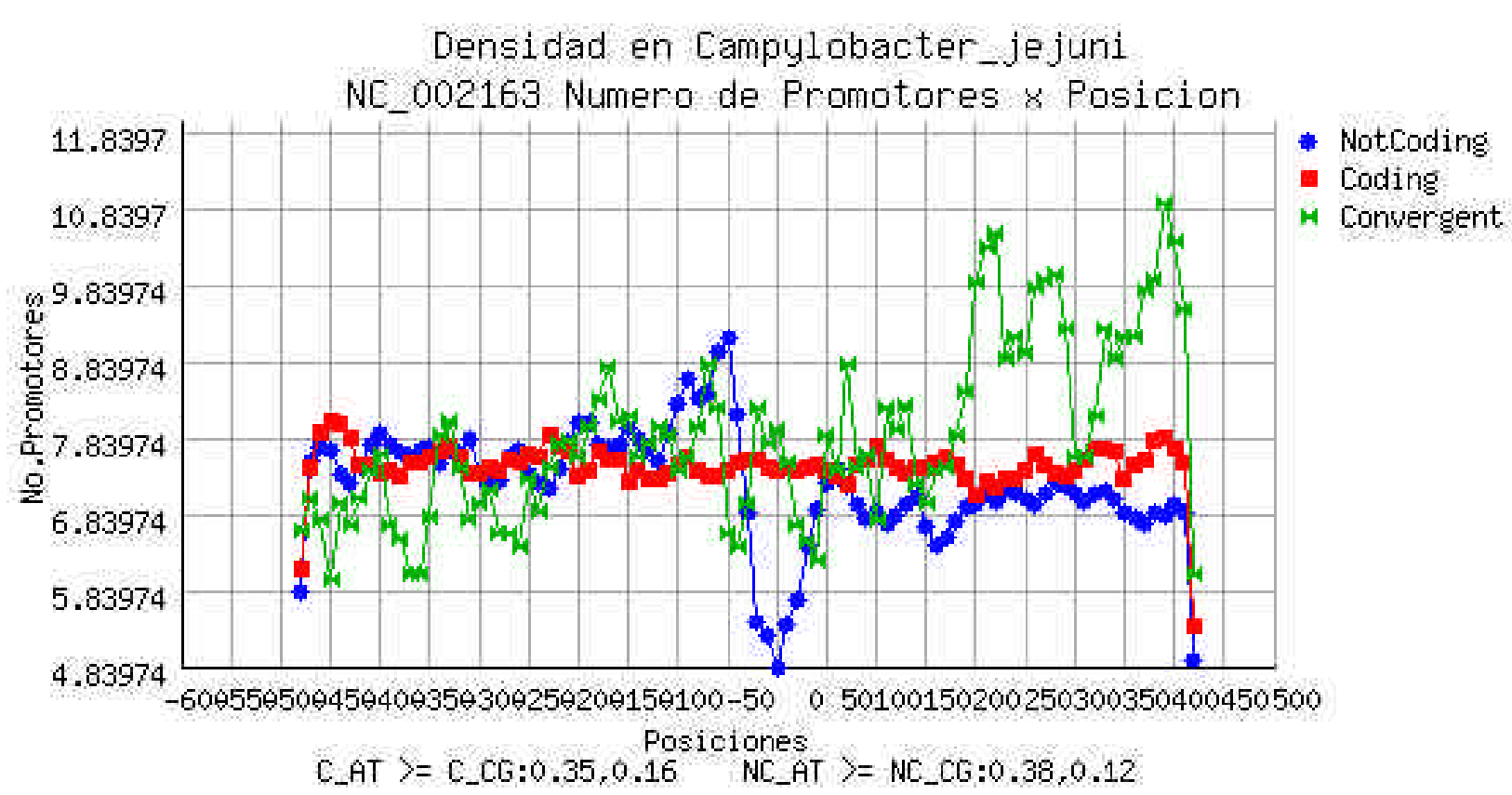
Densidad en Campylobacter_jejuni
NC_002163 Numero de Promotores x Posicion
NotCoding
Coding
Convergent
No.Promotores
11.8397
10.8397
9.83974
8.83974
7.83974
6.83974
5.83974
4.83974
-600550500450400350300250200150100-50 0 50100150200250300350400450500
Posiciones
C_AT >= C_CG:0.35,0.16 NC_AT >= NC_CG:0.38,0.12

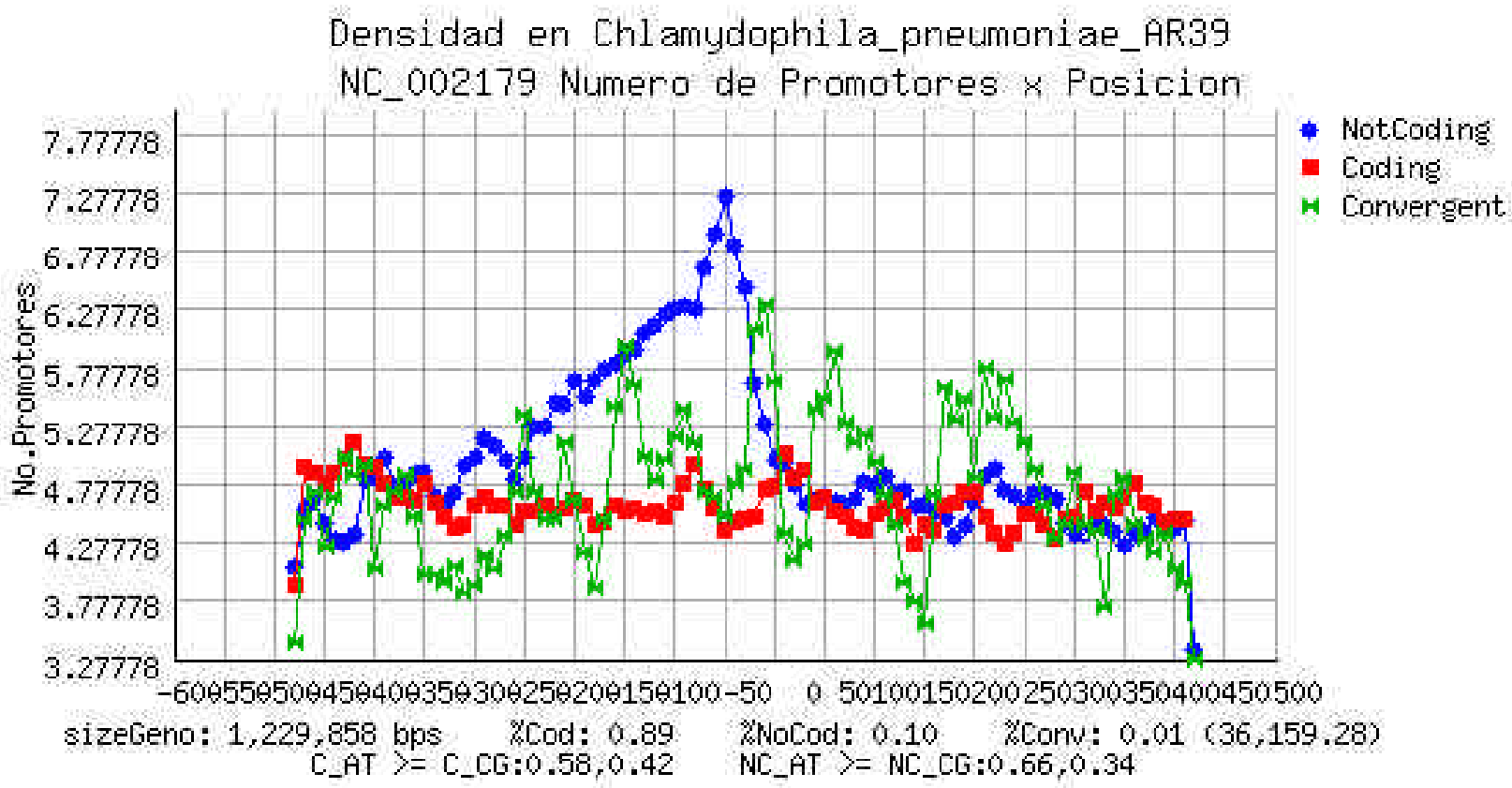
Densidad en Chlamydophila_pneumoniae_AR39
NC_002179 Numero de Promotores x Posicion
No.Promotores
7.77778
7.27778
6.77778
6.27778
5.77778
5.27778
4.77778
4.27778
3.77778
3.27778
-600550500450400350300250200150100-50 0 50100150200250300350400450500
NotCoding
Coding
Convergent
sizeGeno: 1,229,858 bps %Cod: 0.89 %NoCod: 0.10 %Conv: 0.01 (36,159.28)
C_AT >= C_CG:0.58,0.42 NC_AT >= NC_CG:0.66,0.34

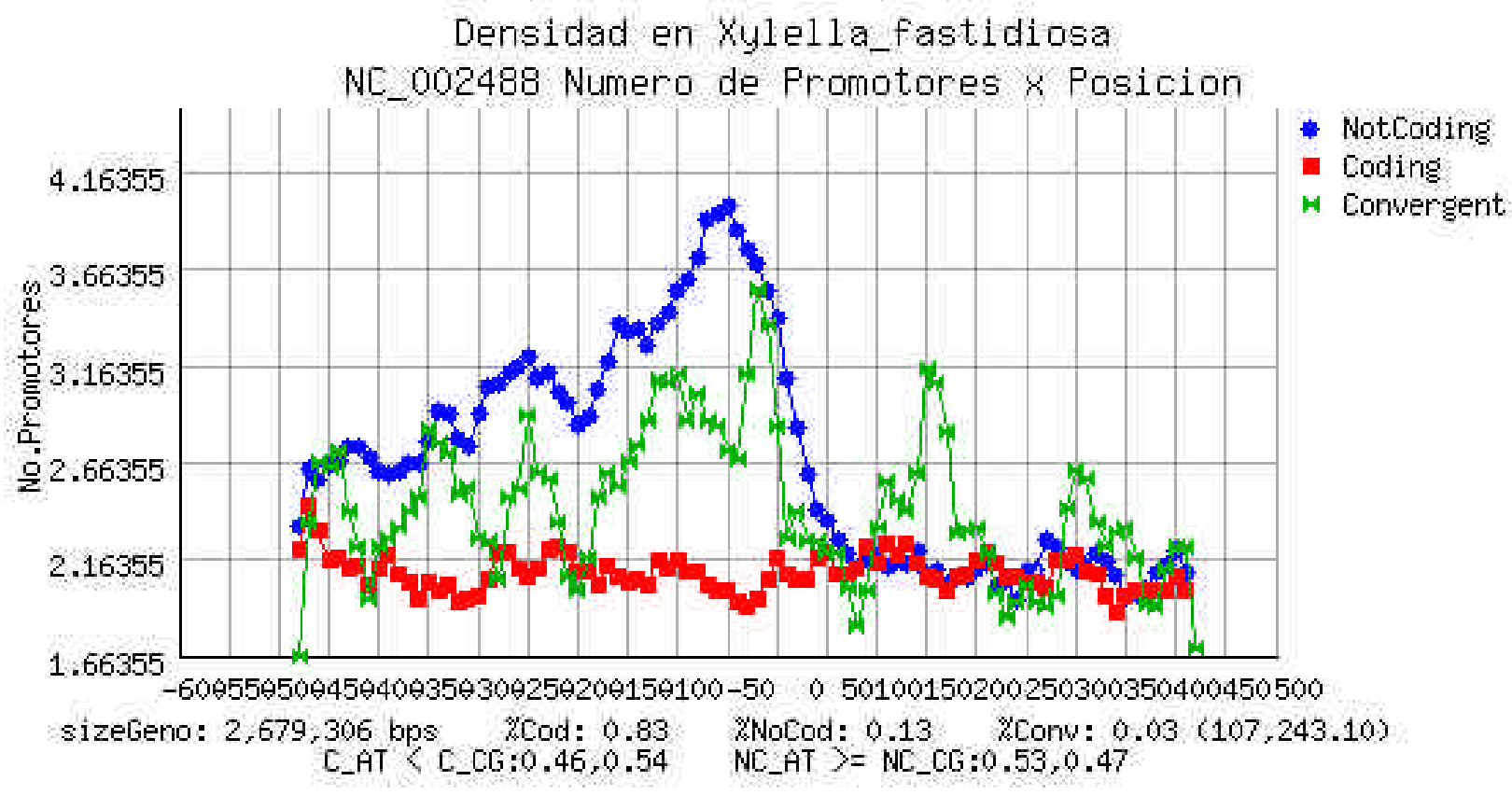
Densidad en Xylella_fastidiosa
NC_002488 Numero de Promotores x Posicion
No.Promotores
4.16355
3.66355
3.16355
2.66355
2.16355
1.66355
-600550500450400350300250200150100-50 0 50100150200250300350400450500
NotCoding
Coding
Convergent
sizeGeno: 2,679,306 bps %Cod: 0.83 %NoCod: 0.13 %Conv: 0.03 (107,243.10)
C_AT < C_CG:0.46,0.54 NC_AT >= NC_CG:0.53,0.47

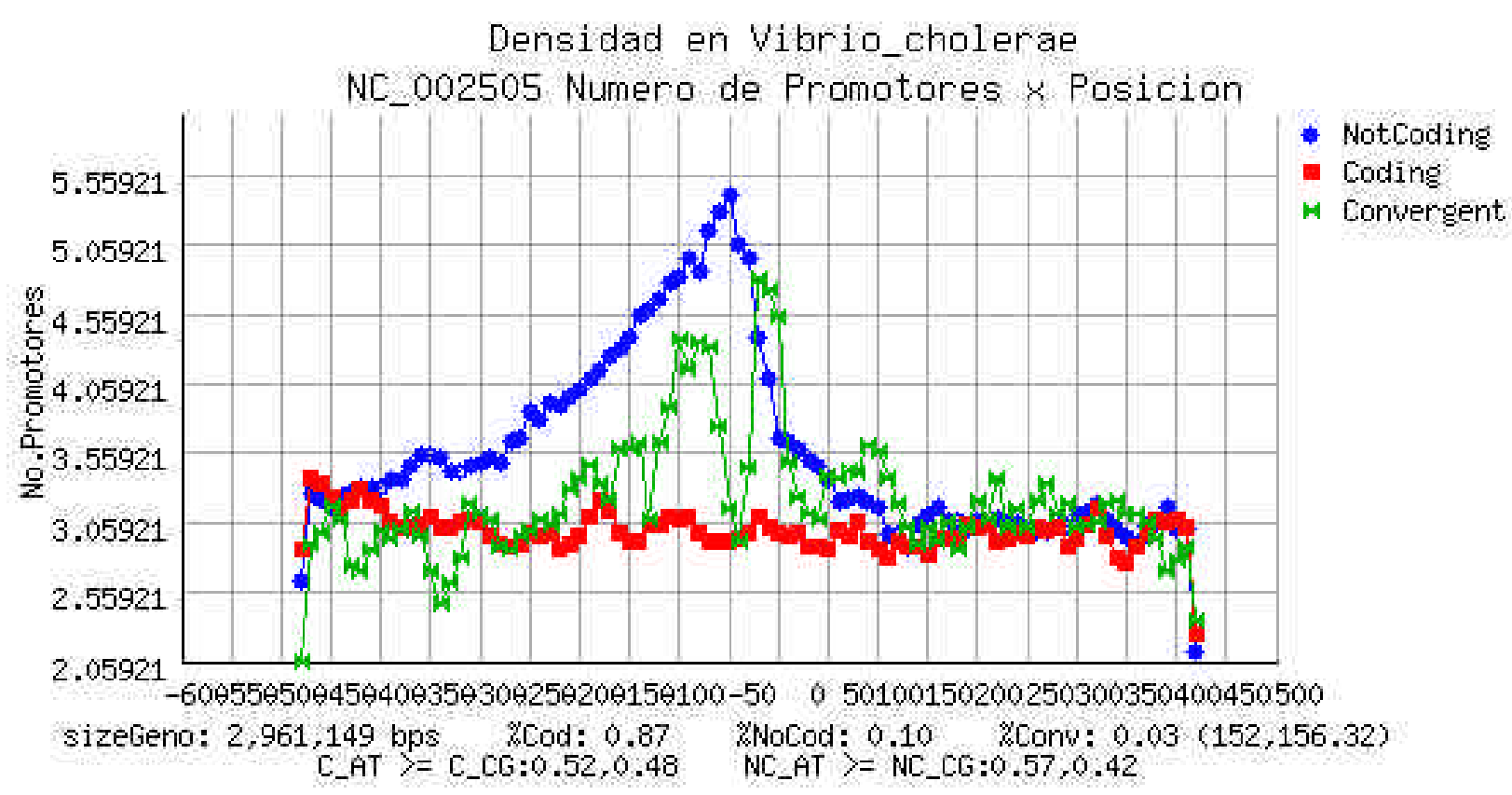
Densidad en Vibrio_cholerae
NC_002505 Numero de Promotores x Posicion
No.Promotores
5.55921
5.05921
4.55921
4.05921
3.55921
3.05921
2.55921
2.05921
-600550500450400350300250200150100-50 0 50100150200250300350400450500
NotCoding
Coding
Convergent
sizeGeno: 2,961,149 bps %Cod: 0.87 %NoCod: 0.10 %Conv: 0.03 (152,156.32)
C_AT >= C_CG:0.52,0.48 NC_AT >= NC_CG:0.57,0.42

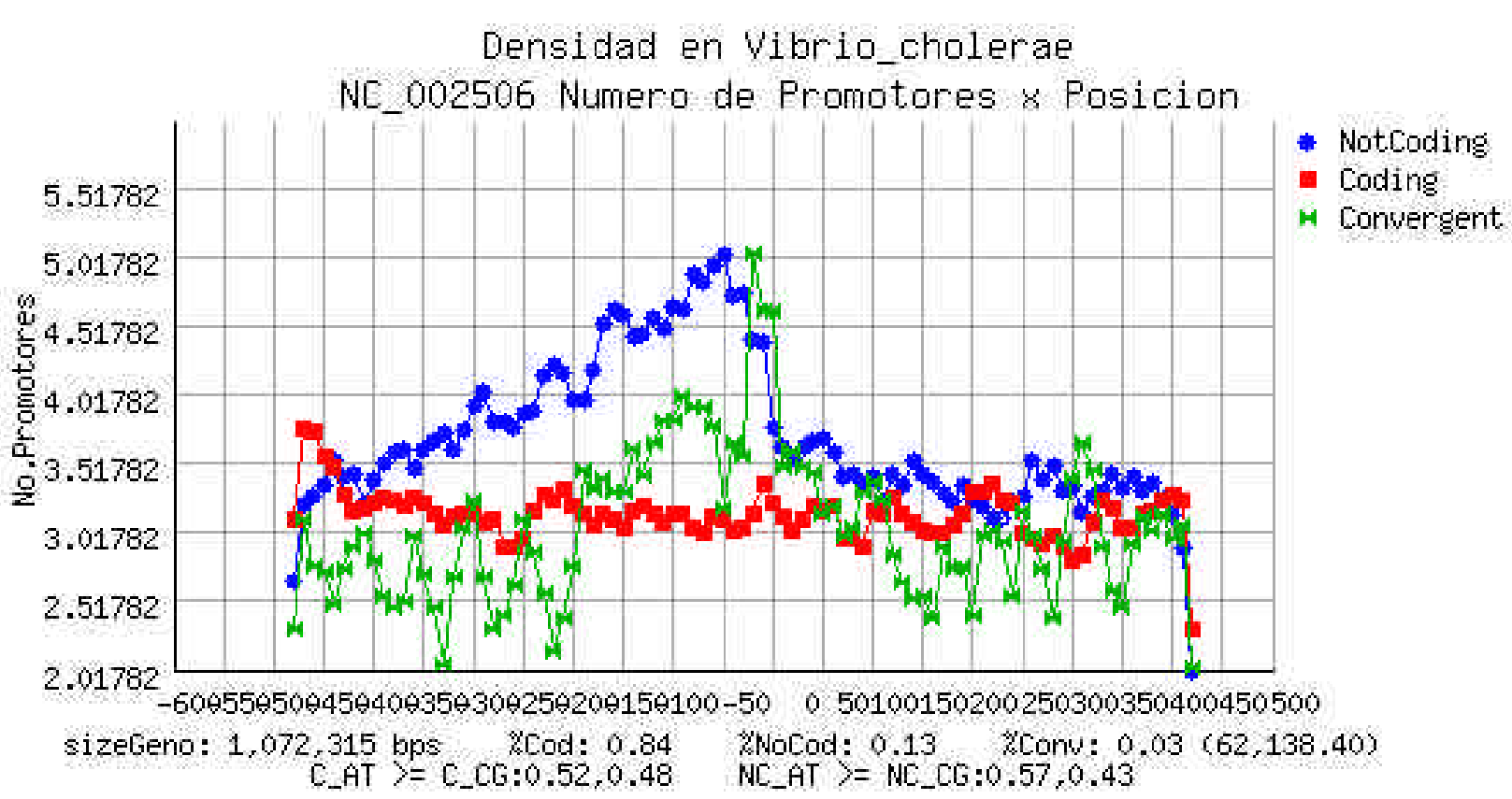
Densidad en Vibrio_cholerae
NC_002506 Numero de Promotores x Posicion
No.Promotores
5.51782
5.01782
4.51782
4.01782
3.51782
3.01782
2.51782
2.01782
-600550500450400350300250200150100-50 0 50100150200250300350400450500
NotCoding
Coding
Convergent
sizeGeno: 1,072,315 bps %Cod: 0.84 %NoCod: 0.13 %Conv: 0.03 (62,138.40)
C_AT >= C_CG:0.52,0.48 NC_AT >= NC_CG:0.57,0.43

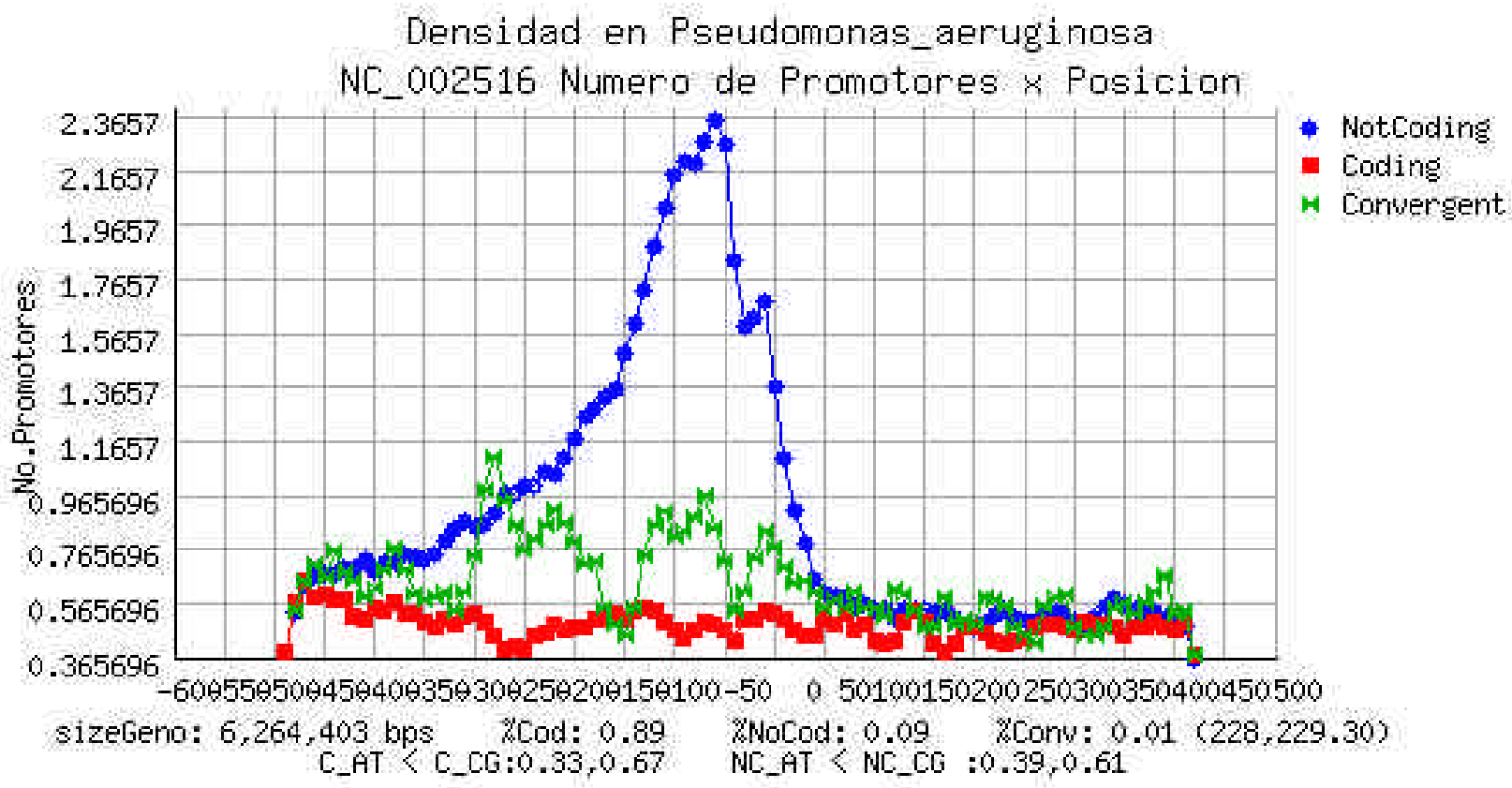
Densidad en Pseudomonas_aeruginosa
NC_002516 Numero de Promotores x Posicion
No.Promotores
NotCoding
Coding
Convergent
sizeGeno: 6,264,403 bps %Cod: 0.89 %NoCod: 0.09 %Conv: 0.01 (228,229.30)
C_AT < C_CG:0.33,0.67 NC_AT < NC_CG :0.39,0.61

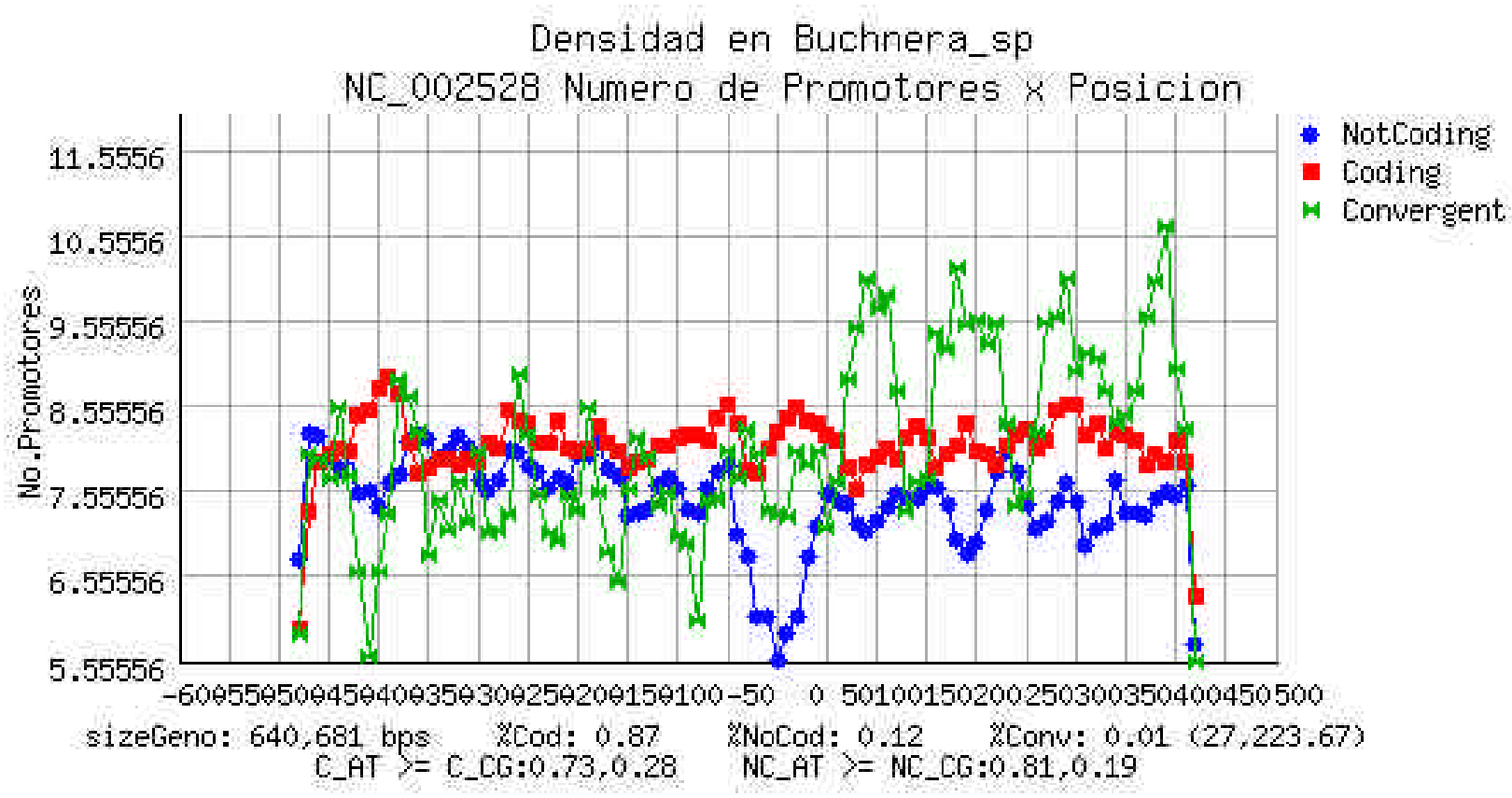
Densidad en Buchnera_sp
NC_002528 Numero de Promotores x Posicion
No.Promotores
NotCoding
Coding
Convergent
sizeGeno: 640,681 bps %Cod: 0.87 %NoCod: 0.12 %Conv: 0.01 (27,223.67)
C_AT >= C_CG:0.73,0.28 NC_AT >= NC_CG:0.81,0.19

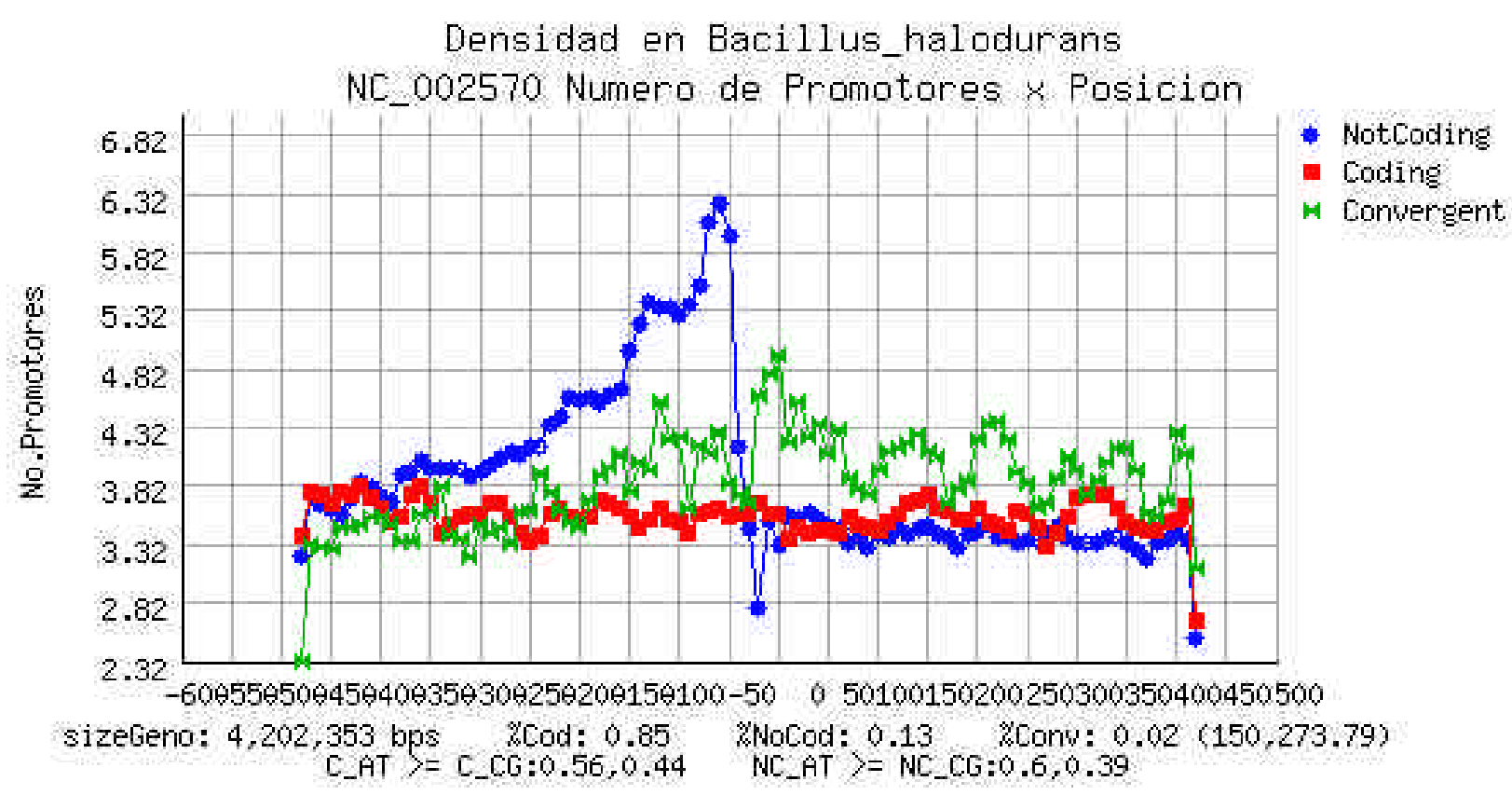
Densidad en Bacillus_halodurans
NC_002570 Numero de Promotores x Posicion
No.Promotores
NotCoding
Coding
Convergent
sizeGeno: 4,202,353 bps %Cod: 0.85 %NoCod: 0.13 %Conv: 0.02 (150,273.79)
C_AT >= C_CG:0.56,0.44 NC_AT >= NC_CG:0.6,0.39

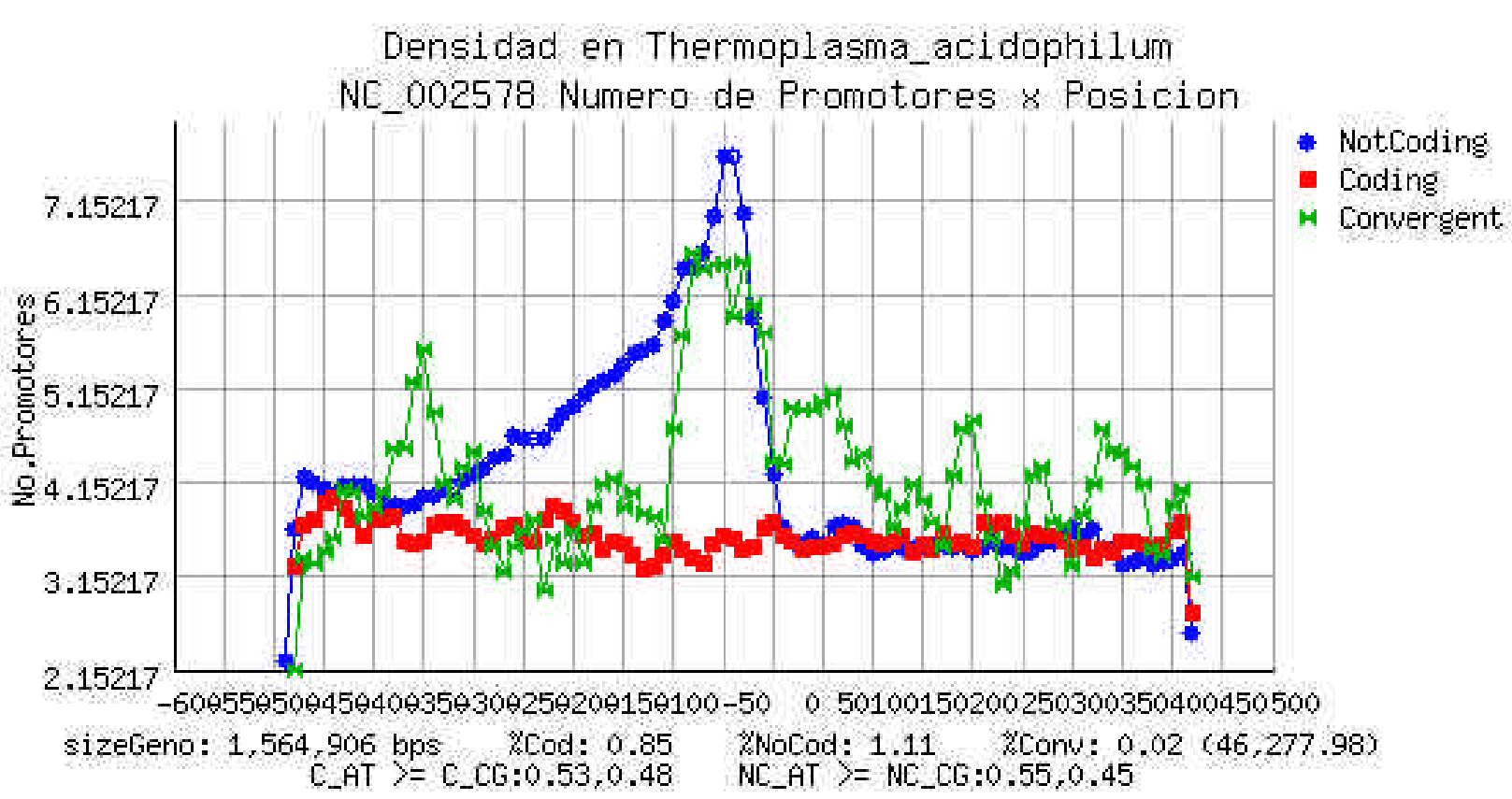
Densidad en Thermoplasma_acidophilum
NC_002578 Numero de Promotores x Posicion
No.Promotores
NotCoding
Coding
Convergent
sizeGeno: 1,564,906 bps %Cod: 0.85 %NoCod: 1.11 %Conv: 0.02 (46,277.98)
C_AT >= C_CG:0.53,0.48 NC_AT >= NC_CG:0.55,0.45

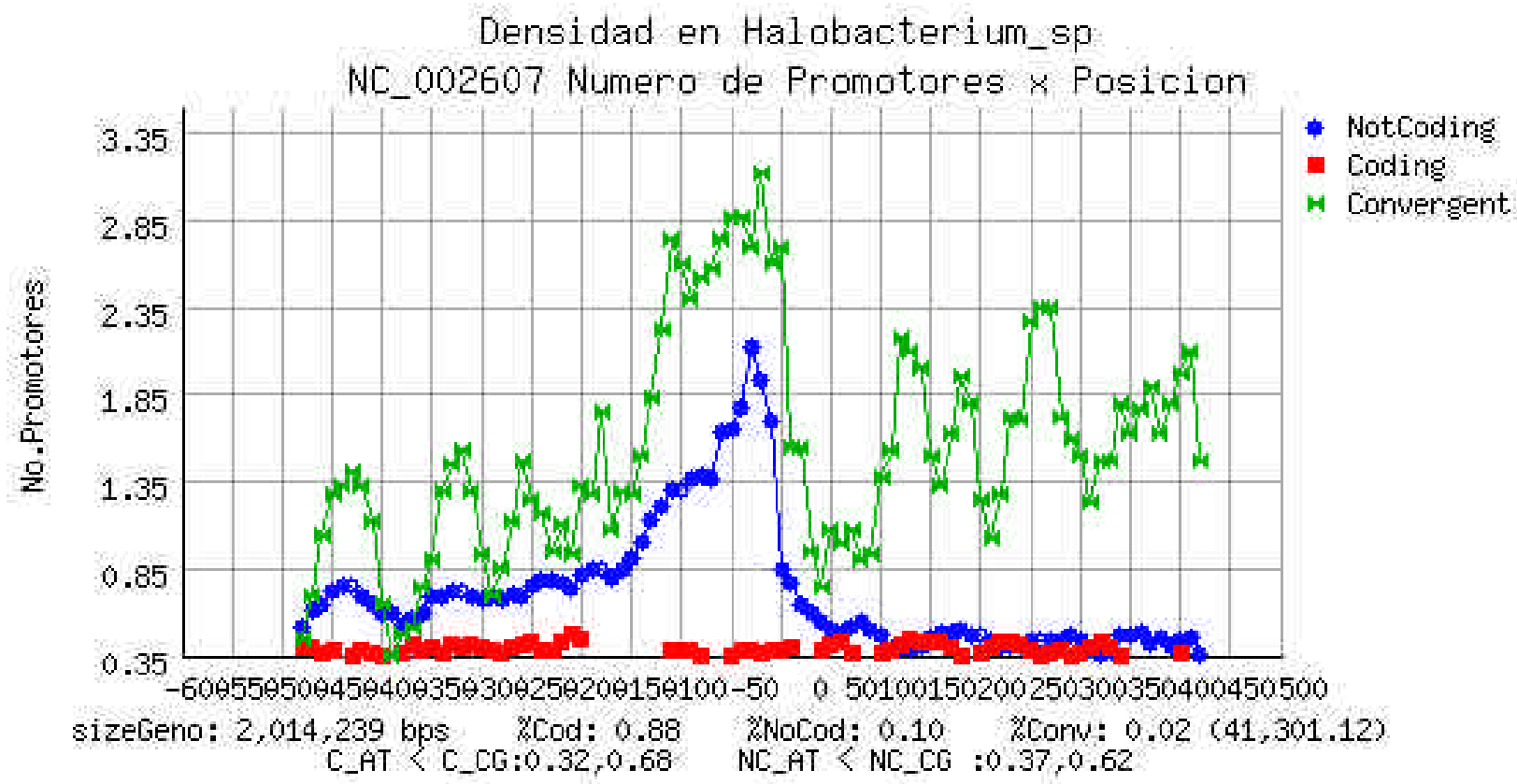
Densidad en Halobacterium_sp
NC_002607 Numero de Promotores x Posicion
NotCoding
Coding
Convergent
No.Promotores
3.35
2.85
2.35
1.85
1.35
0.85
0.35
-600550500450400350300250200150100-50 0 50100150200250300350400450500
sizeGeno: 2,014,239 bps %Cod: 0.88 %NoCod: 0.10 %Conv: 0.02 (41,301.12)
C_AT < C_CG:0.32,0.68 NC_AT < NC_CG :0.37,0.62

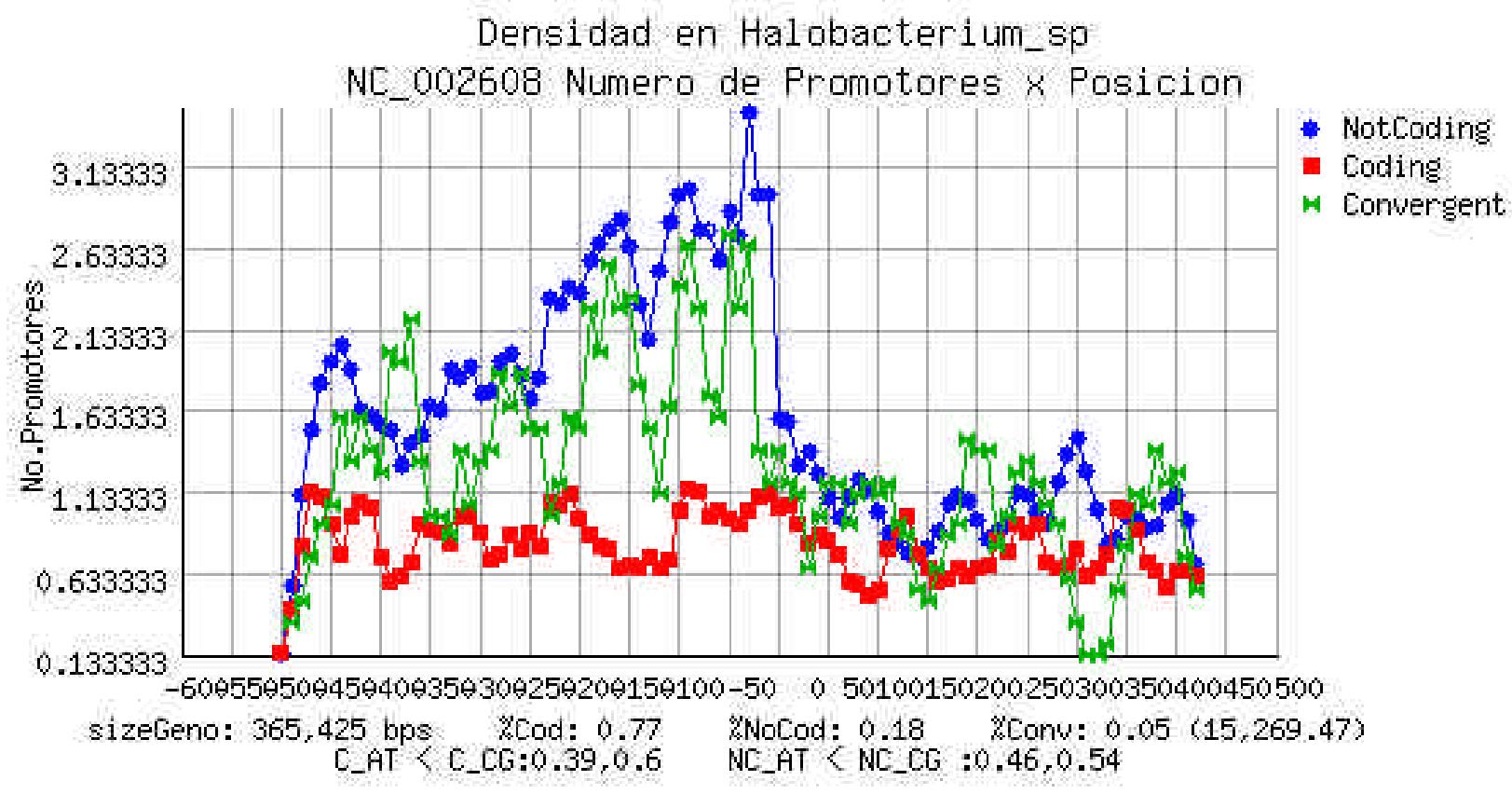
Densidad en Halobacterium_sp
NC_002608 Numero de Promotores x Posicion
NotCoding
Coding
Convergent
No.Promotores
3.13333
2.63333
2.13333
1.63333
1.13333
0.633333
0.133333
-600550500450400350300250200150100-50 0 50100150200250300350400450500
sizeGeno: 365,425 bps %Cod: 0.77 %NoCod: 0.18 %Conv: 0.05 (15,269.47)
C_AT < C_CG:0.39,0.6 NC_AT < NC_CG :0.46,0.54

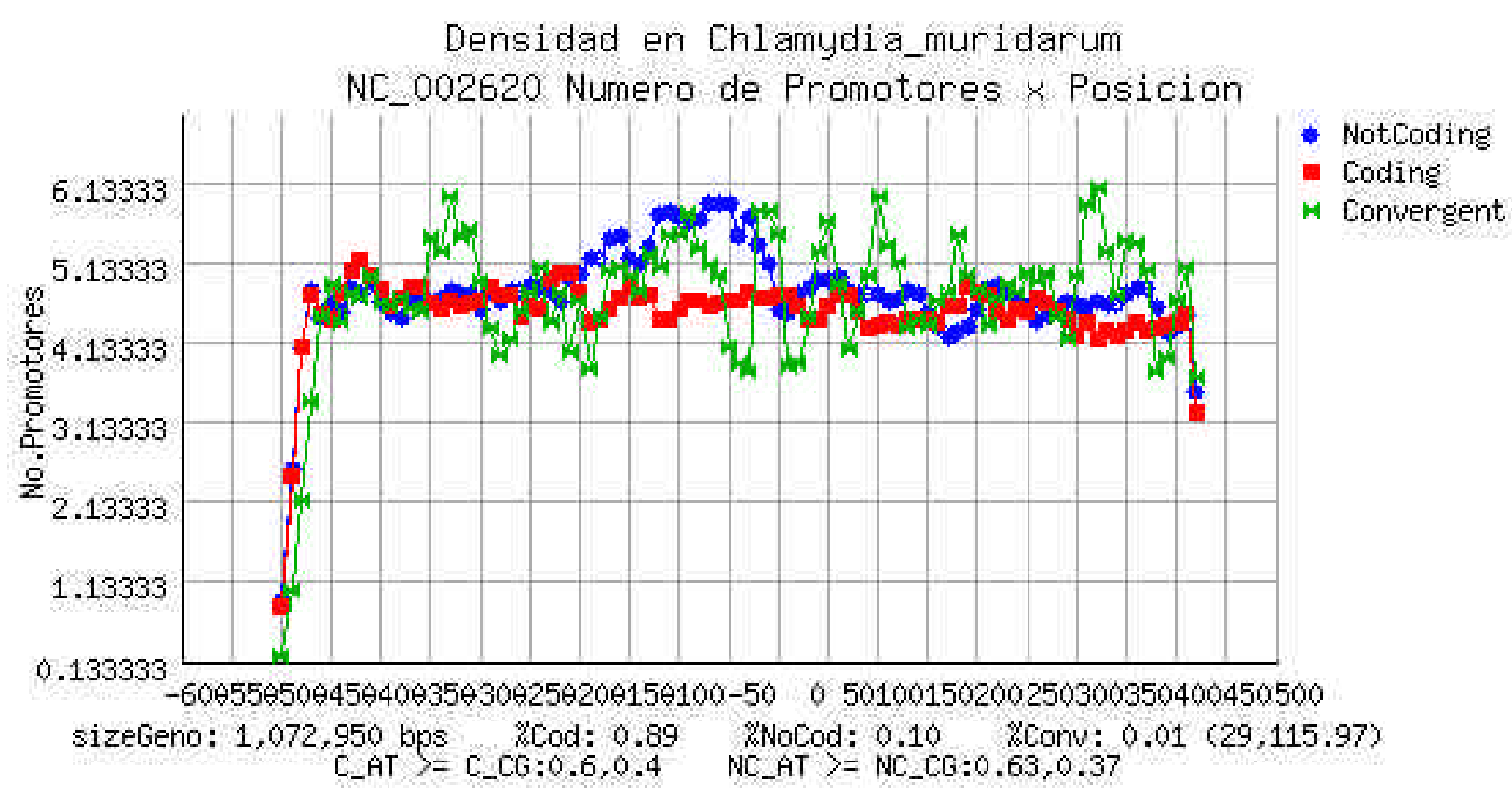
Densidad en Chlamydia_muridarum
NC_002620 Numero de Promotores x Posicion
NotCoding
Coding
Convergent
No.Promotores
6.13333
5.13333
4.13333
3.13333
2.13333
1.13333
0.133333
-600550500450400350300250200150100-50 0 50100150200250300350400450500
sizeGeno: 1,072,950 bps %Cod: 0.89 %NoCod: 0.10 %Conv: 0.01 (29,115.97)
C_AT >= C_CG:0.6,0.4 NC_AT >= NC_CG:0.63,0.37

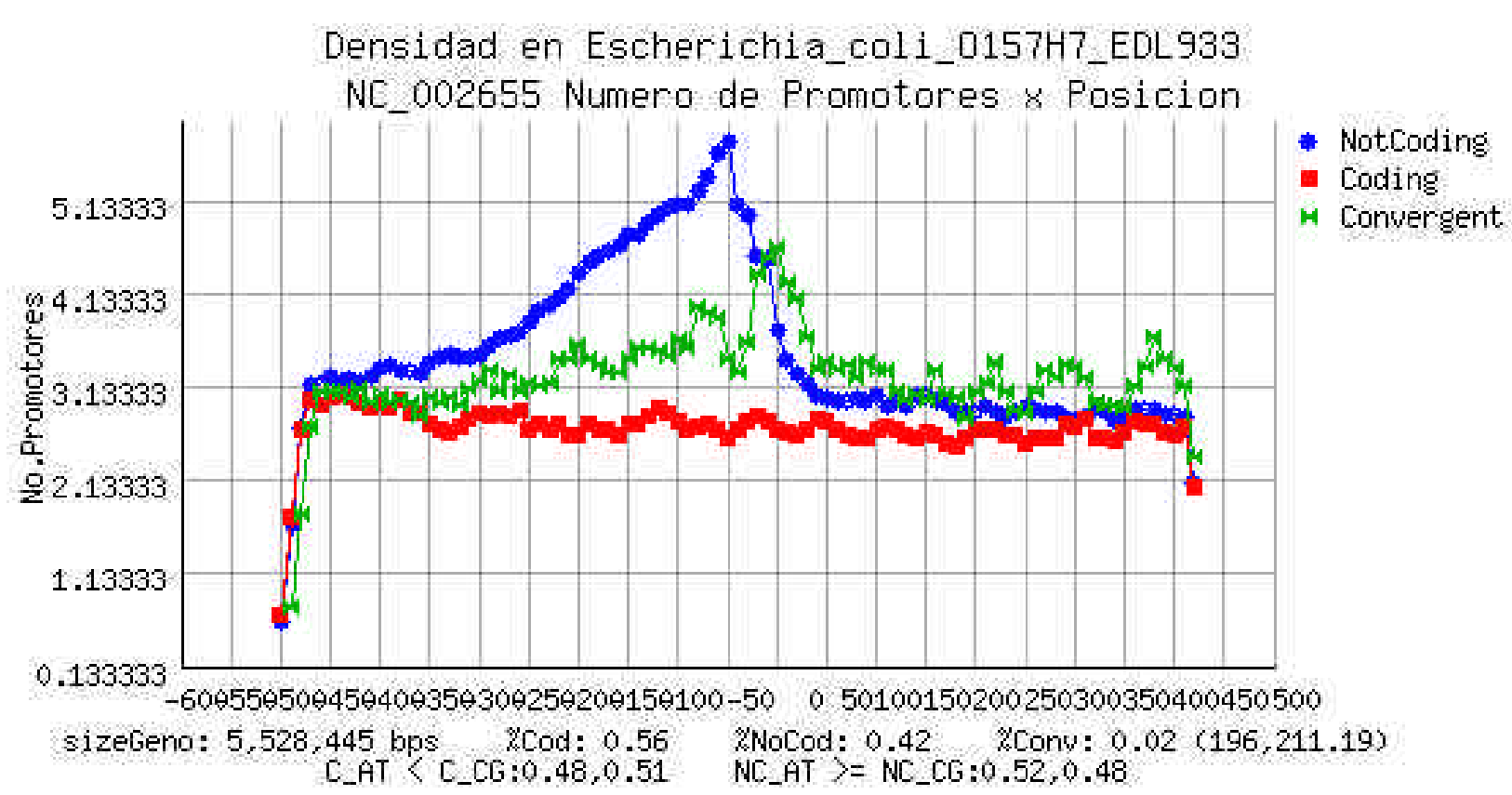
Densidad en Escherichia_coli_O157H7_EDL933
NC_002655 Numero de Promotores x Posicion
NotCoding
Coding
Convergent
No.Promotores
5.13333
4.13333
3.13333
2.13333
1.13333
0.133333
-600550500450400350300250200150100-50 0 50100150200250300350400450500
sizeGeno: 5,528,445 bps %Cod: 0.56 %NoCod: 0.42 %Conv: 0.02 (196,211.19)
C_AT < C_CG:0.48,0.51 NC_AT >= NC_CG:0.52,0.48

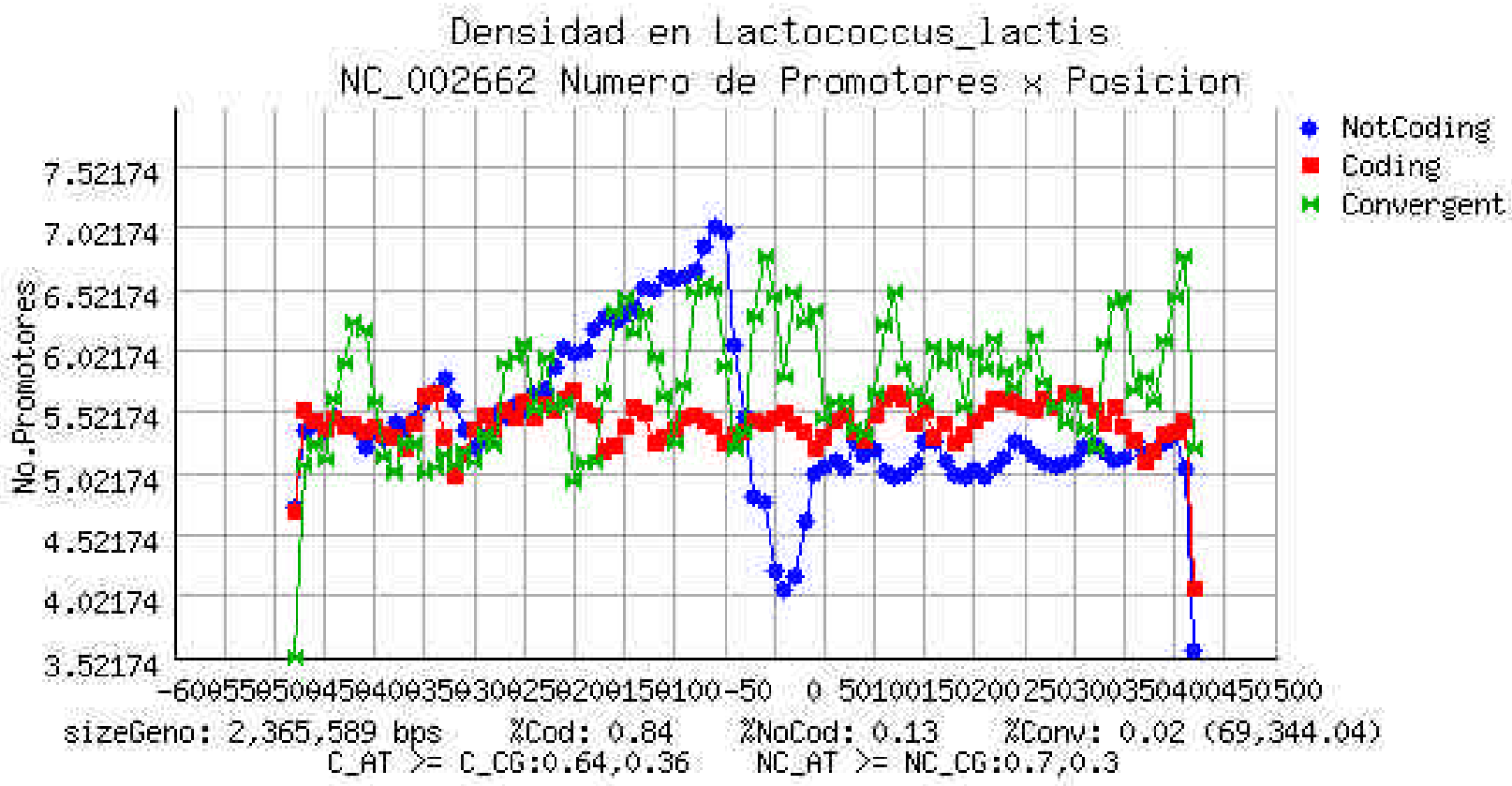
Densidad en Lactococcus_lactis
NC_002662 Numero de Promotores x Posicion
NotCoding
Coding
Convergent
No.Promotores
7.52174
7.02174
6.52174
6.02174
5.52174
5.02174
4.52174
4.02174
3.52174
-600550500450400350300250200150100-50 0 50100150200250300350400450500
sizeGeno: 2,365,589 bps %Cod: 0.84 %NoCod: 0.13 %Conv: 0.02 (69,344.04)
C_AT >= C_CG:0.64,0.36 NC_AT >= NC_CG:0.7,0.3

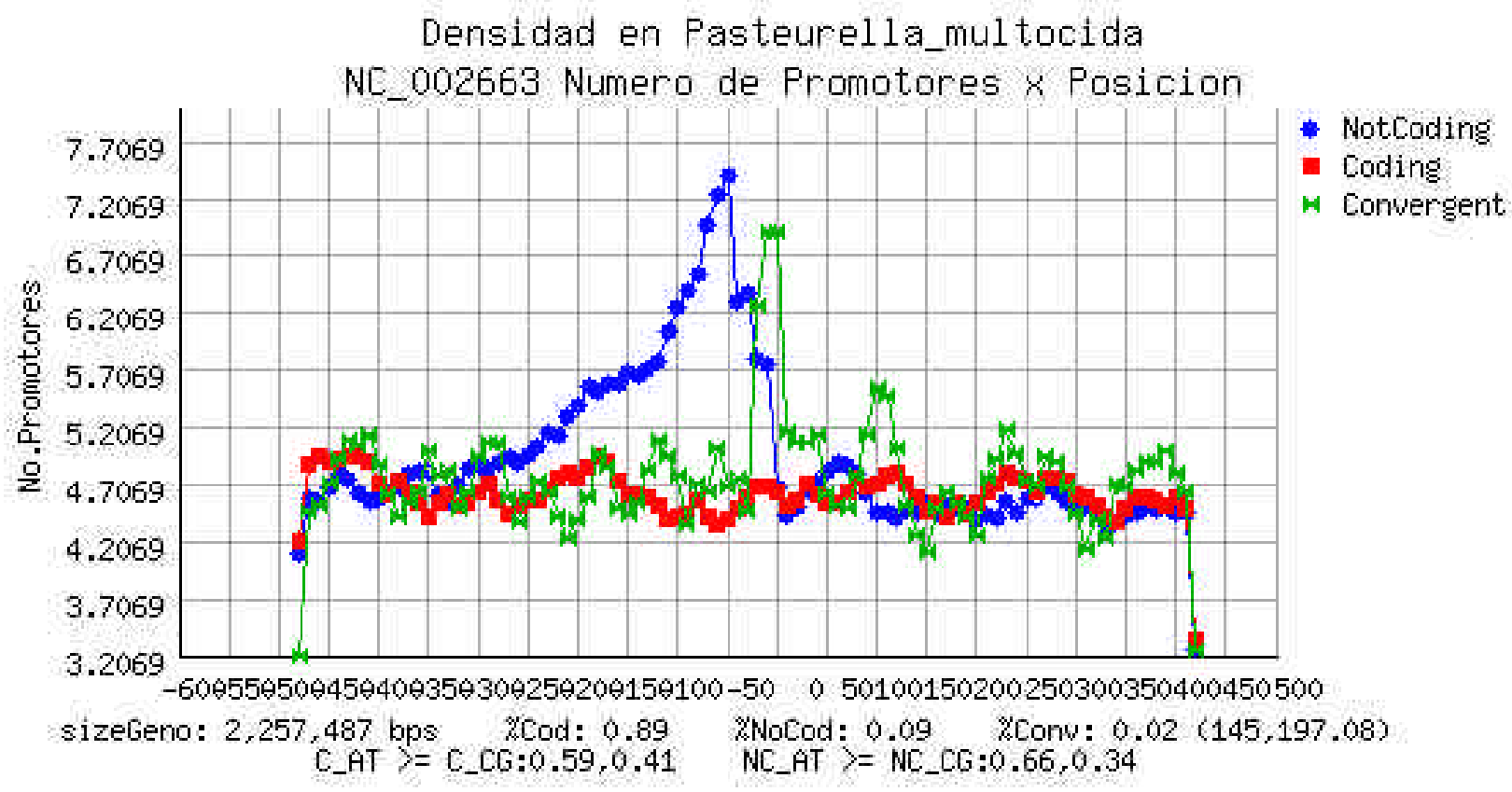
Densidad en Pasteurella_multocida
NC_002663 Numero de Promotores x Posicion
NotCoding
Coding
Convergent
No.Promotores
7.7069
7.2069
6.7069
6.2069
5.7069
5.2069
4.7069
4.2069
3.7069
3.2069
-600550500450400350300250200150100-50 0 50100150200250300350400450500
sizeGeno: 2,257,487 bps %Cod: 0.89 %NoCod: 0.09 %Conv: 0.02 (145,197.08)
C_AT >= C_CG:0.59,0.41 NC_AT >= NC_CG:0.66,0.34

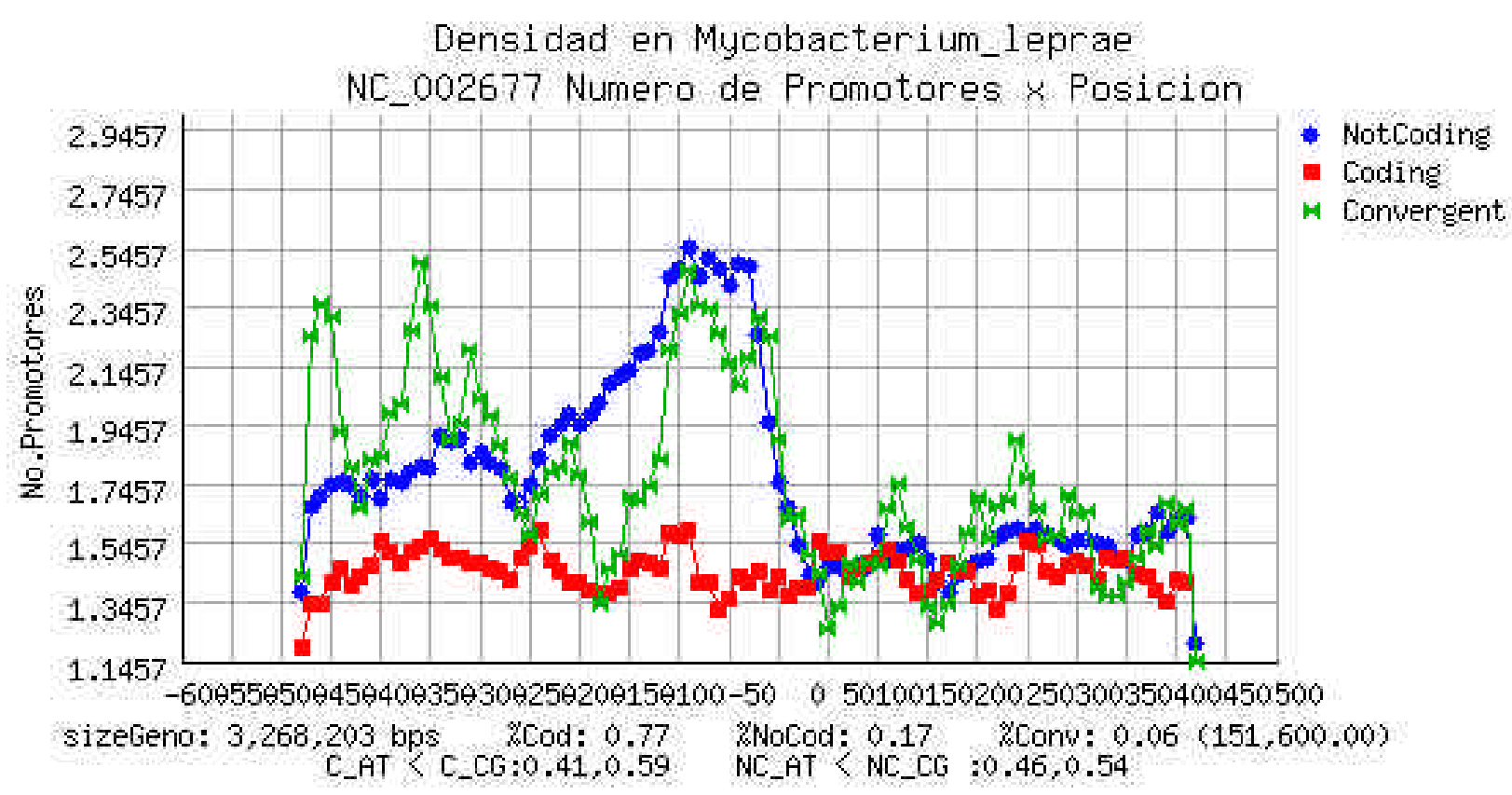
Densidad en Mycobacterium_leprae
NC_002677 Numero de Promotores x Posicion
NotCoding
Coding
Convergent
No.Promotores
2.9457
2.7457
2.5457
2.3457
2.1457
1.9457
1.7457
1.5457
1.3457
1.1457
-600550500450400350300250200150100-50 0 50100150200250300350400450500
sizeGeno: 3,268,203 bps %Cod: 0.77 %NoCod: 0.17 %Conv: 0.06 (151,600.00)
C_AT < C_CG:0.41,0.59 NC_AT < NC_CG :0.46,0.54

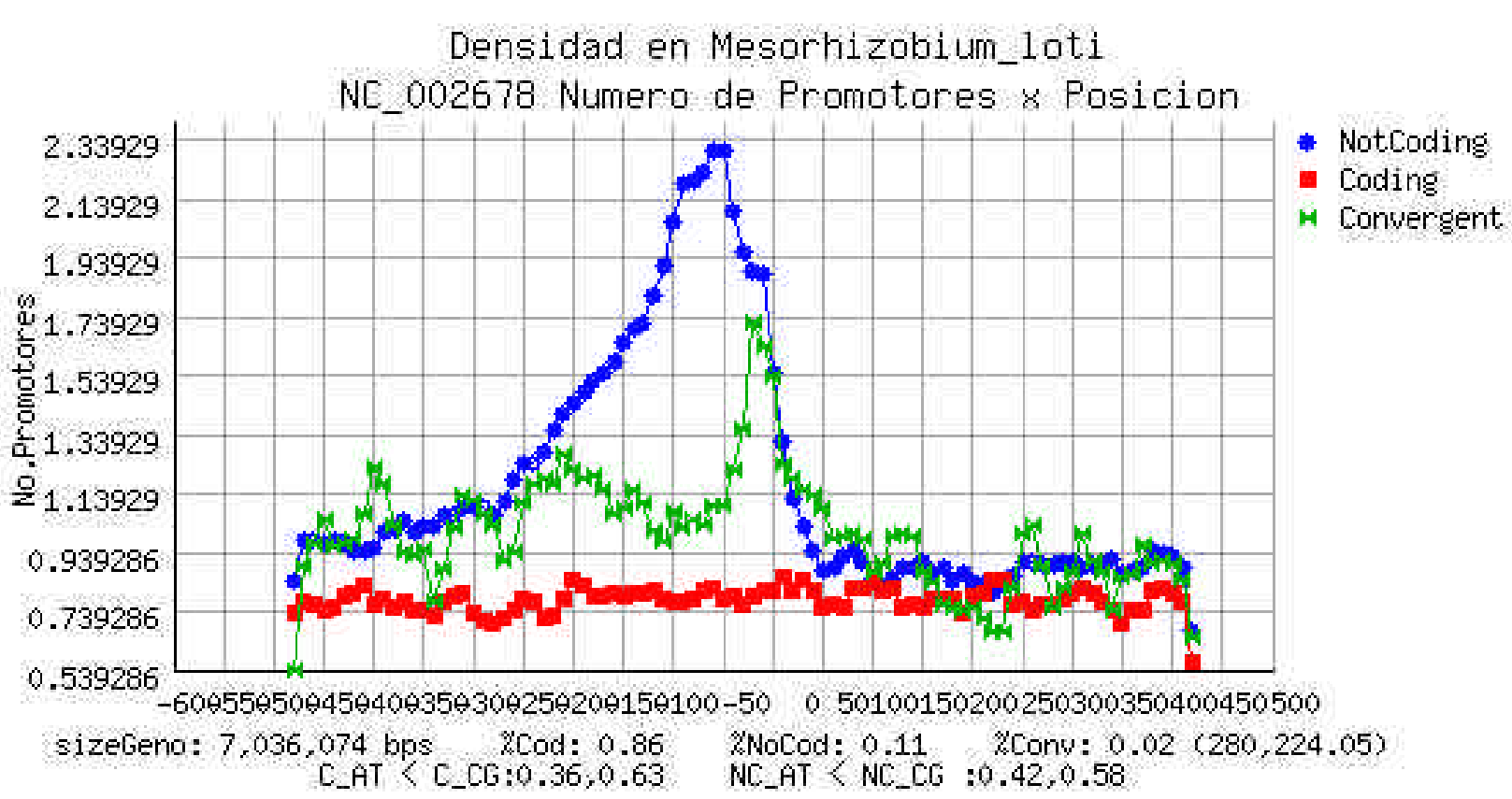
Densidad en Mesorhizobium_loti
NC_002678 Numero de Promotores x Posicion
NotCoding
Coding
Convergent
No.Promotores
2.33929
2.13929
1.93929
1.73929
1.53929
1.33929
1.13929
0.939286
0.739286
0.539286
-600550500450400350300250200150100-50 0 50100150200250300350400450500
sizeGeno: 7,036,074 bps %Cod: 0.86 %NoCod: 0.11 %Conv: 0.02 (280,224.05)
C_AT < C_CG:0.36,0.63 NC_AT < NC_CG :0.42,0.58

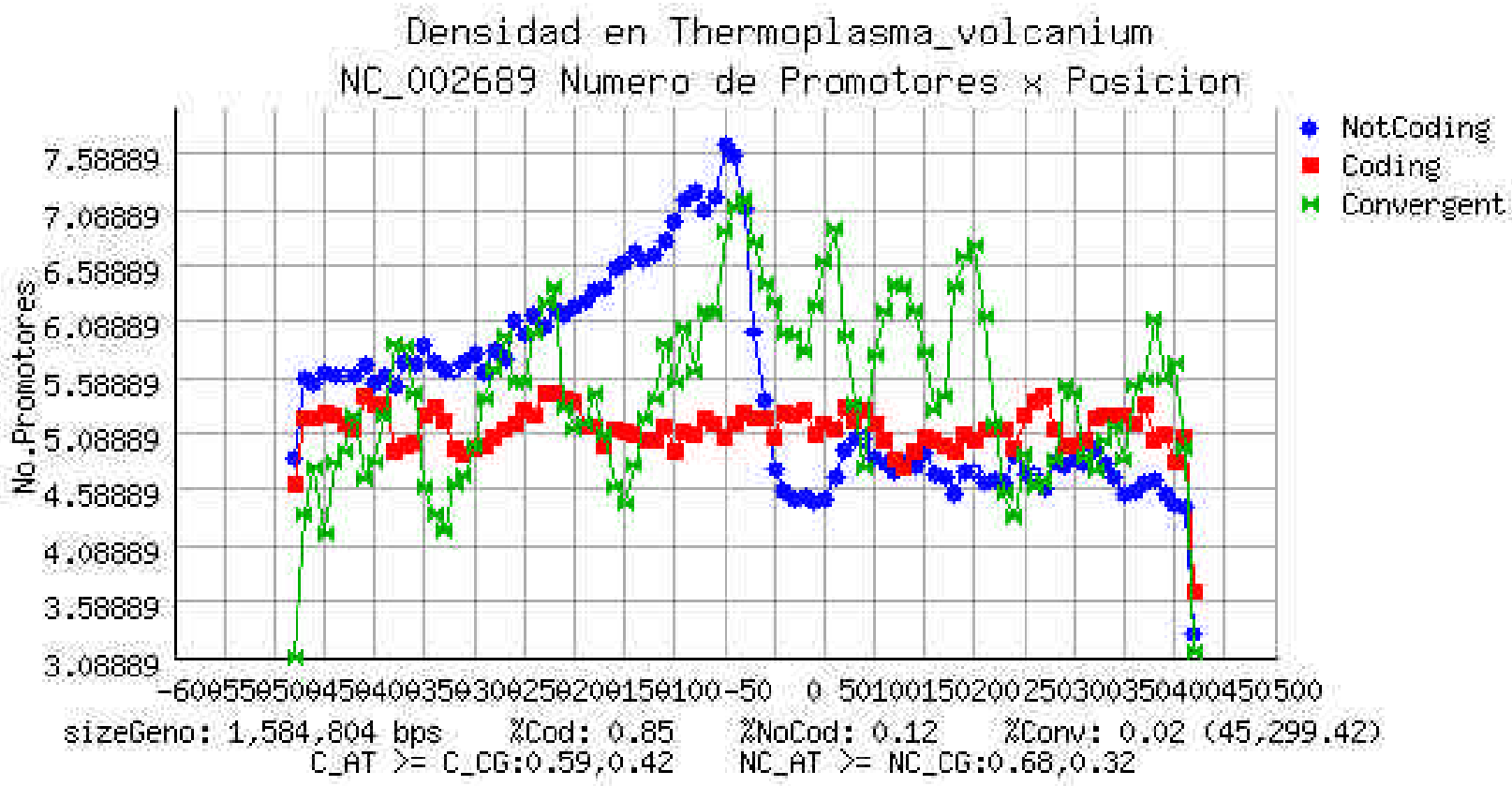
Densidad en Thermoplasma_volcanium
NC_002689 Numero de Promotores x Posicion
NotCoding
Coding
Convergent
No.Promotores
7.58889
7.08889
6.58889
6.08889
5.58889
5.08889
4.58889
4.08889
3.58889
3.08889
-600550500450400350300250200150100-50 0 50100150200250300350400450500
sizeGeno: 1,584,804 bps %Cod: 0.85 %NoCod: 0.12 %Conv: 0.02 (45,299.42)
C_AT >= C_CG:0.59,0.42 NC_AT >= NC_CG:0.68,0.32

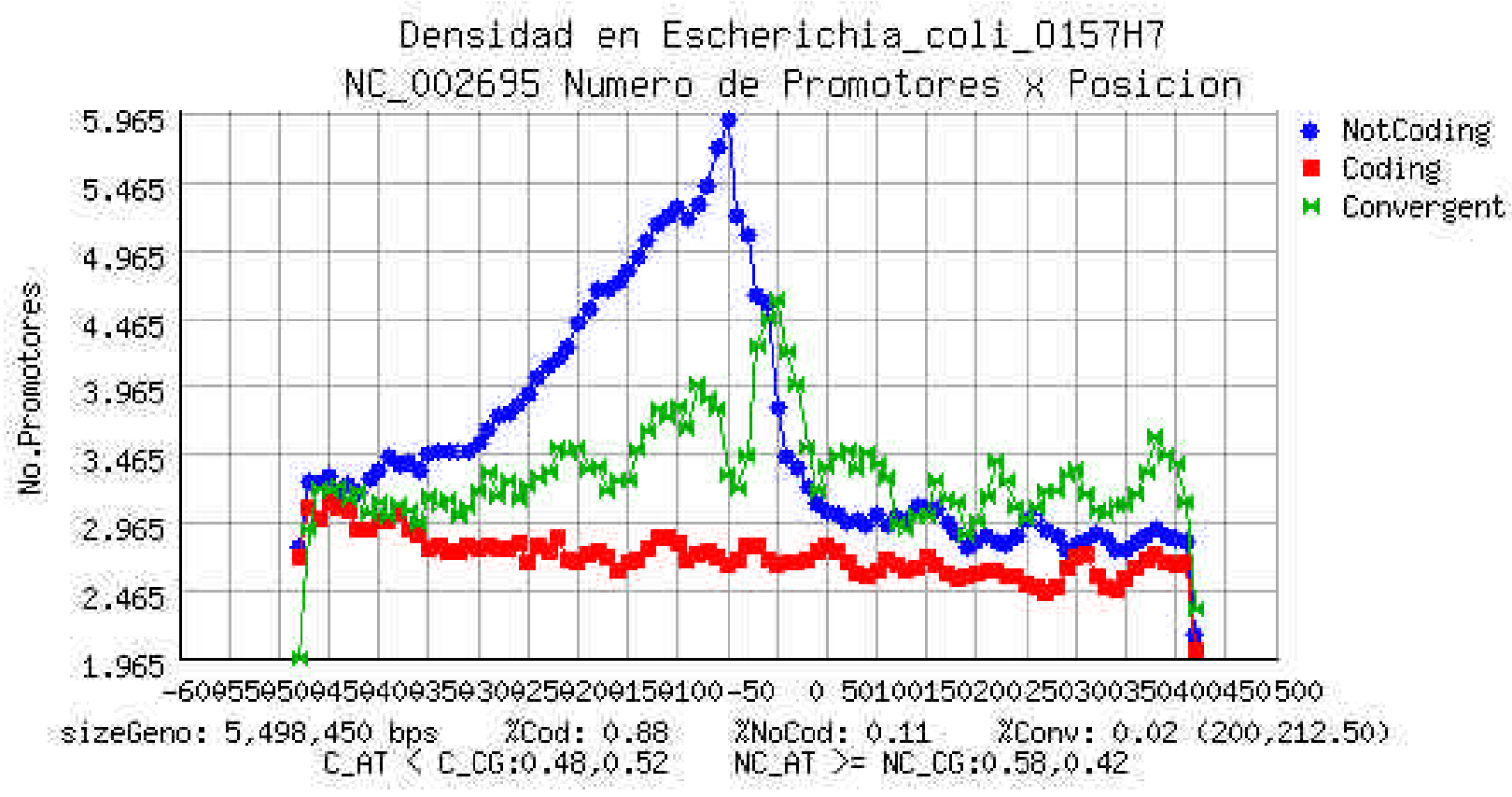
Densidad en Escherichia_coli_O157H7
NC_002695 Numero de Promotores x Posicion
NotCoding
Coding
Convergent
No.Promotores
5.965
5.465
4.965
4.465
3.965
3.465
2.965
2.465
1.965
-600550500450400350300250200150100-50 0 50100150200250300350400450500
sizeGeno: 5,498,450 bps %Cod: 0.88 %NoCod: 0.11 %Conv: 0.02 (200,212.50)
C_AT < C_CG:0.48,0.52 NC_AT >= NC_CG:0.58,0.42

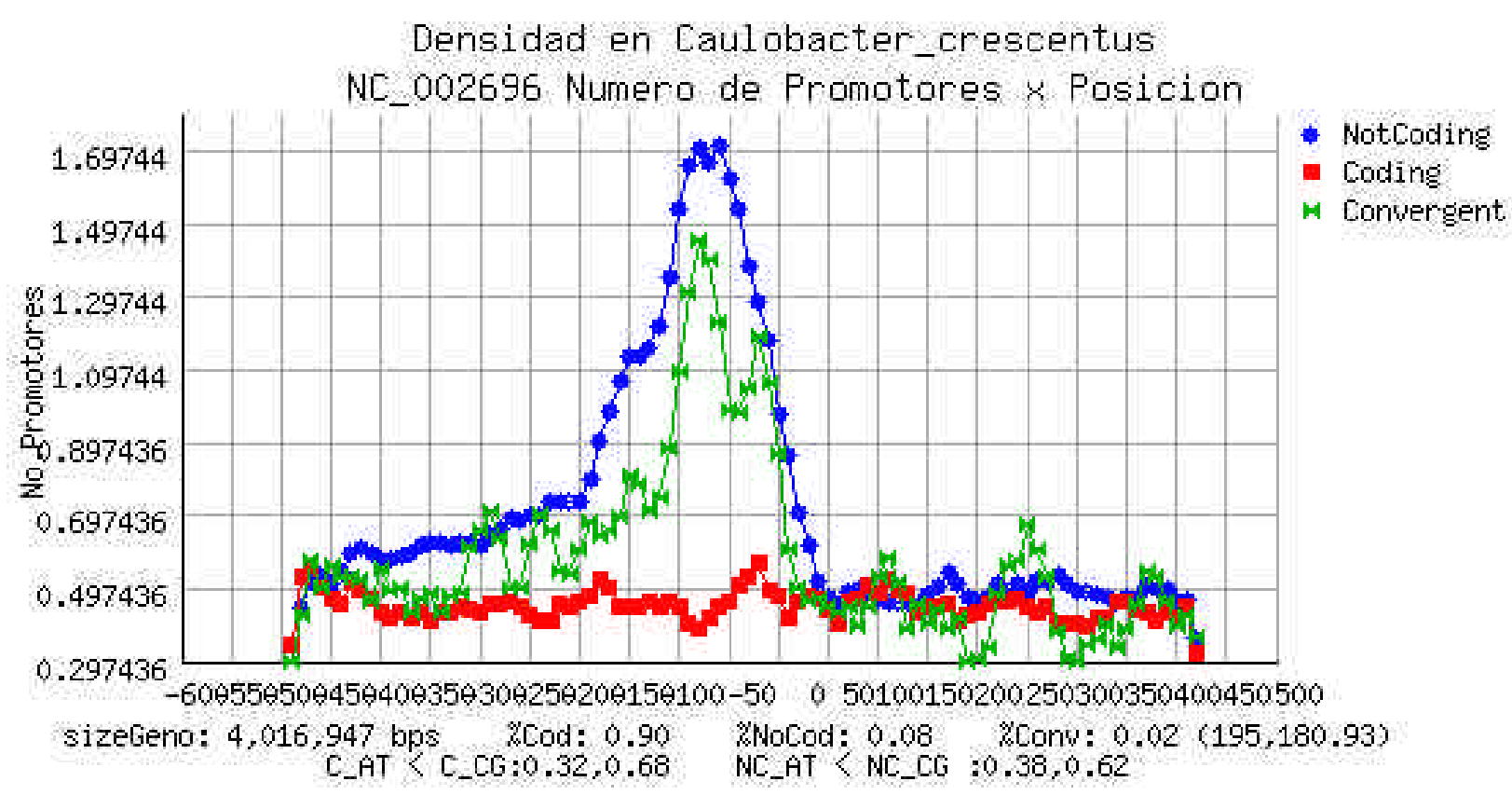
Densidad en Caulobacter_crescentus
NC_002696 Numero de Promotores x Posicion
NotCoding
Coding
Convergent
No.Promotores
1.69744
1.49744
1.29744
1.09744
0.897436
0.697436
0.497436
0.297436
-600550500450400350300250200150100-50 0 50100150200250300350400450500
sizeGeno: 4,016,947 bps %Cod: 0.90 %NoCod: 0.08 %Conv: 0.02 (195,180.93)
C_AT < C_CG:0.32,0.68 NC_AT < NC_CG :0.38,0.62

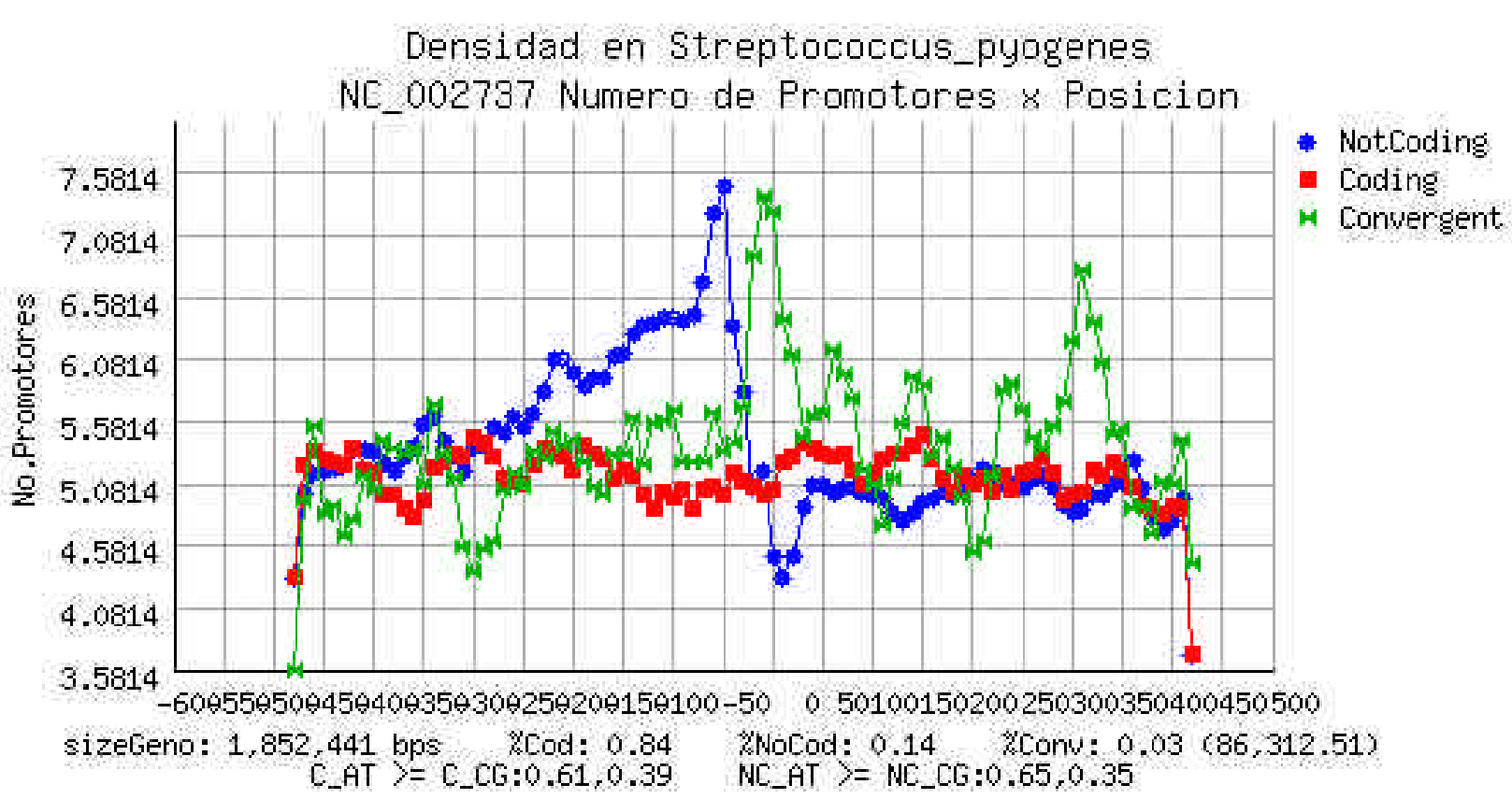
Densidad en Streptococcus_pyogenes
NC_002737 Numero de Promotores x Posicion
NotCoding
Coding
Convergent
No.Promotores
7.5814
7.0814
6.5814
6.0814
5.5814
5.0814
4.5814
4.0814
3.5814
-600550500450400350300250200150100-50 0 50100150200250300350400450500
sizeGeno: 1,852,441 bps %Cod: 0.84 %NoCod: 0.14 %Conv: 0.03 (86,312.51)
C_AT >= C_CG:0.61,0.39 NC_AT >= NC_CG:0.65,0.35

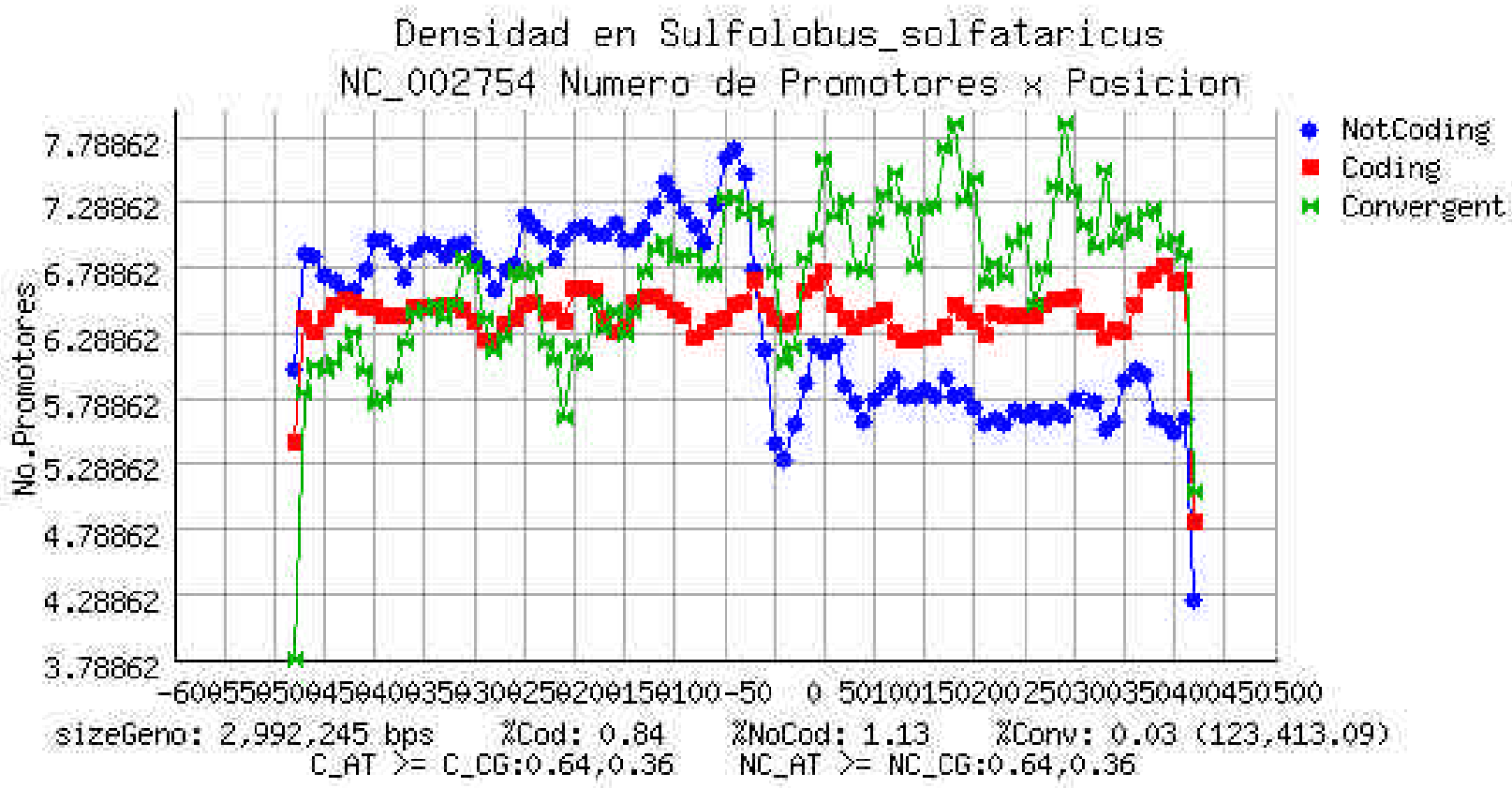
Densidad en Sulfolobus_solfataricus
NC_002754 Numero de Promotores x Posicion
NotCoding
Coding
Convergent
No.Promotores
sizeGeno: 2,992,245 bps %Cod: 0.84 %NoCod: 1.13 %Conv: 0.03 (123,413.09)
C_AT >= C_CG:0.64,0.36 NC_AT >= NC_CG:0.64,0.36

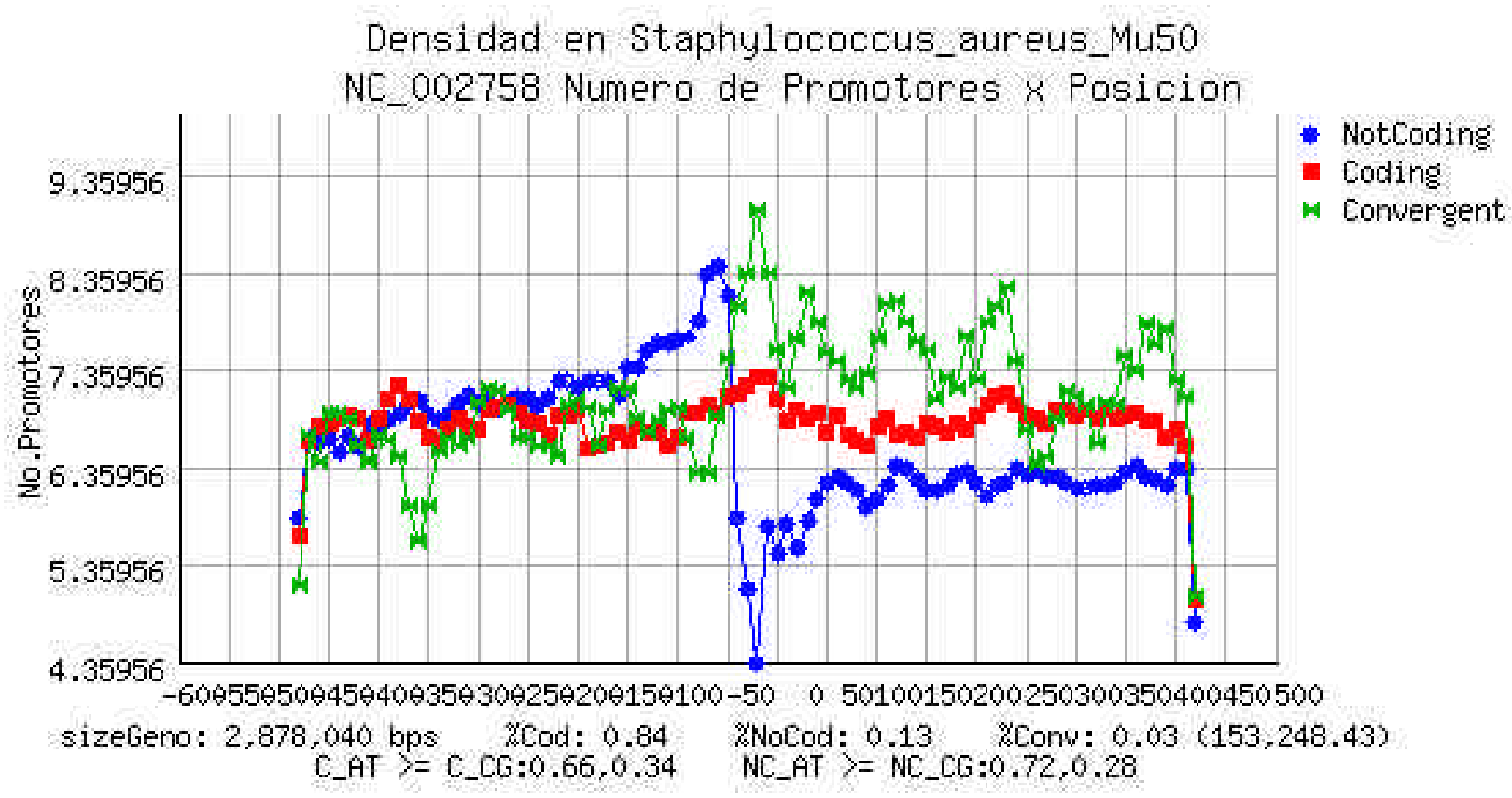
Densidad en Staphylococcus_aureus_Mu50
NC_002758 Numero de Promotores x Posicion
NotCoding
Coding
Convergent
No.Promotores
sizeGeno: 2,878,040 bps %Cod: 0.84 %NoCod: 0.13 %Conv: 0.03 (153,248.43)
C_AT >= C_CG:0.66,0.34 NC_AT >= NC_CG:0.72,0.28

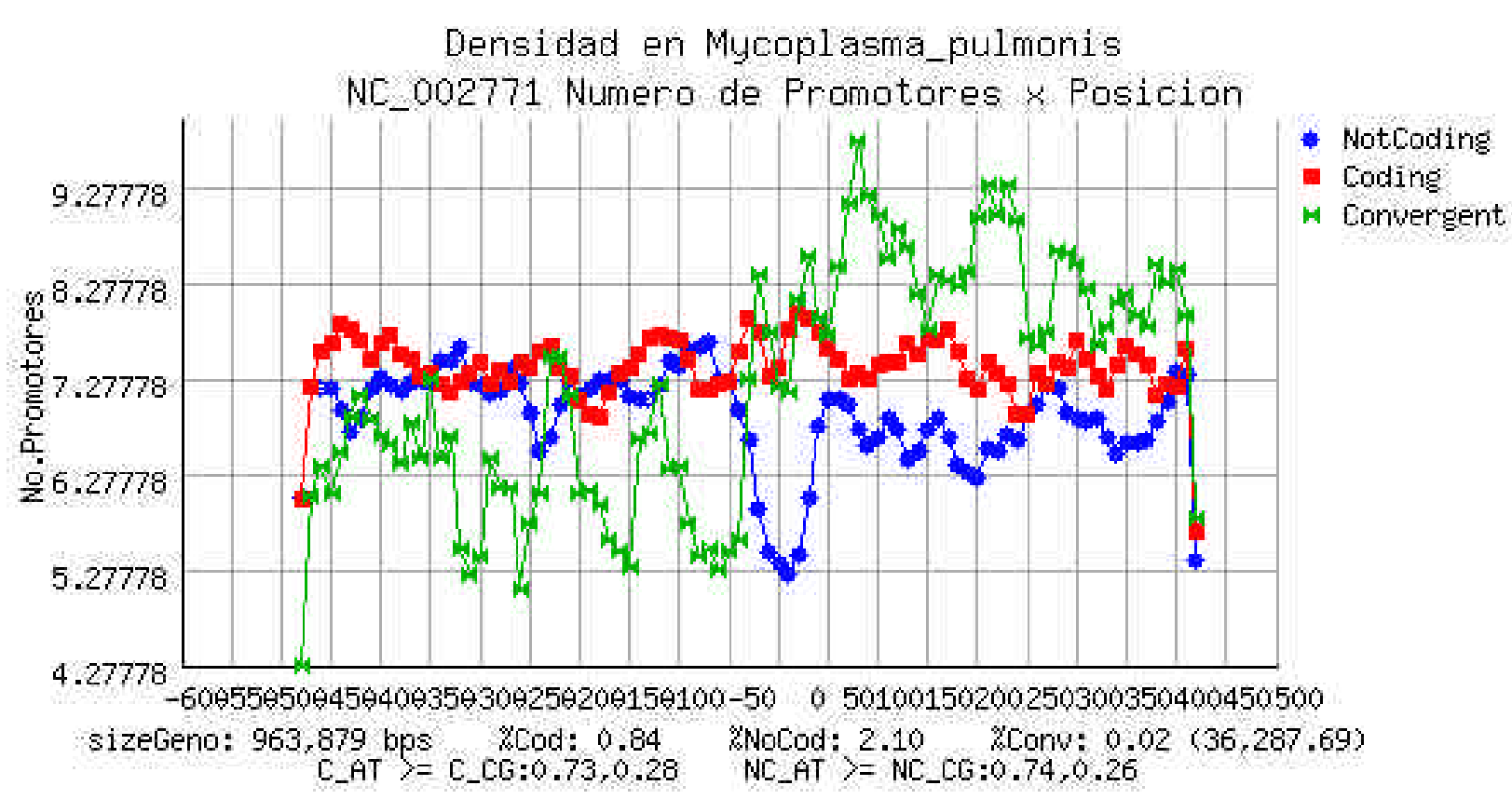
Densidad en Mycoplasma_pulmonis
NC_002771 Numero de Promotores x Posicion
NotCoding
Coding
Convergent
No.Promotores
sizeGeno: 963,879 bps %Cod: 0.84 %NoCod: 2.10 %Conv: 0.02 (36,287.69)
C_AT >= C_CG:0.73,0.28 NC_AT >= NC_CG:0.74,0.26

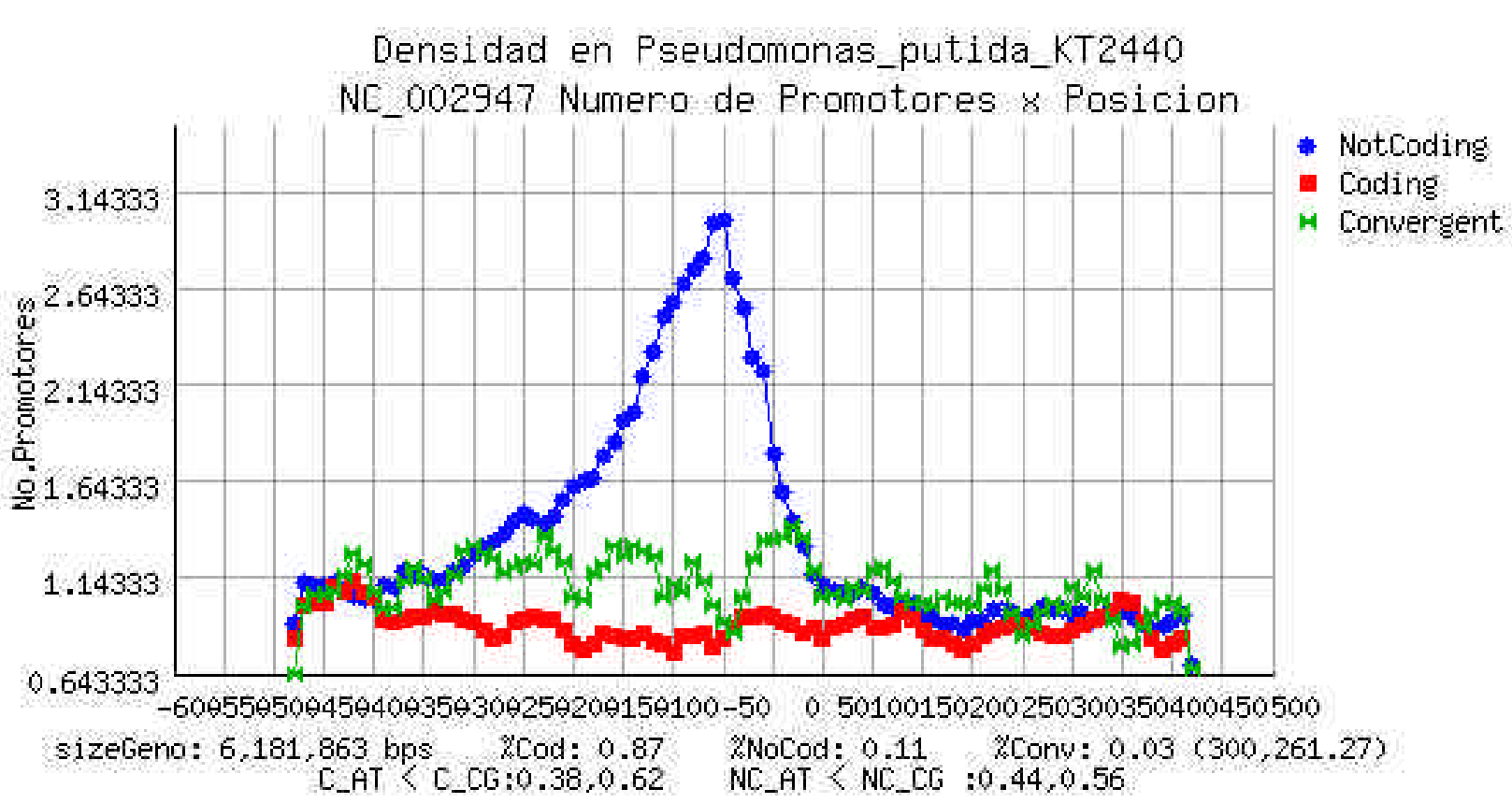
Densidad en Pseudomonas_putida_KT2440
NC_002947 Numero de Promotores x Posicion
NotCoding
Coding
Convergent
No.Promotores
sizeGeno: 6,181,863 bps %Cod: 0.87 %NoCod: 0.11 %Conv: 0.03 (300,261.27)
C_AT < C_CG:0.38,0.62 NC_AT < NC_CG :0.44,0.56

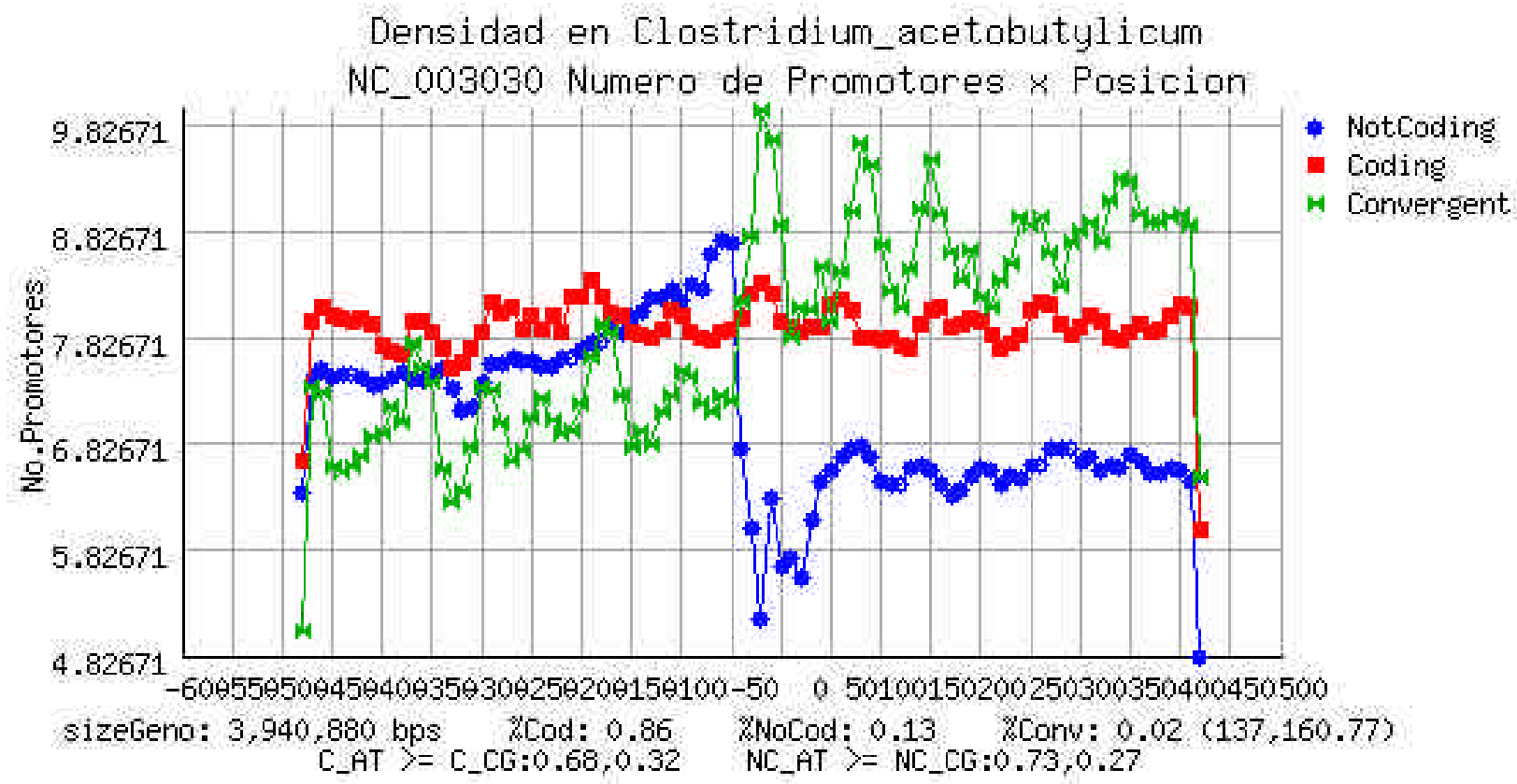
Densidad en Clostridium_acetobutylicum
NC_003030 Numero de Promotores x Posicion
No.Promotores
9.82671
8.82671
7.82671
6.82671
5.82671
4.82671
-600550500450400350300250200150100-50 0 50100150200250300350400450500
NotCoding
Coding
Convergent
sizeGeno: 3,940,880 bps %Cod: 0.86 %NoCod: 0.13 %Conv: 0.02 (137,160.77)
C_AT >= C_CG:0.68,0.32 NC_AT >= NC_CG:0.73,0.27

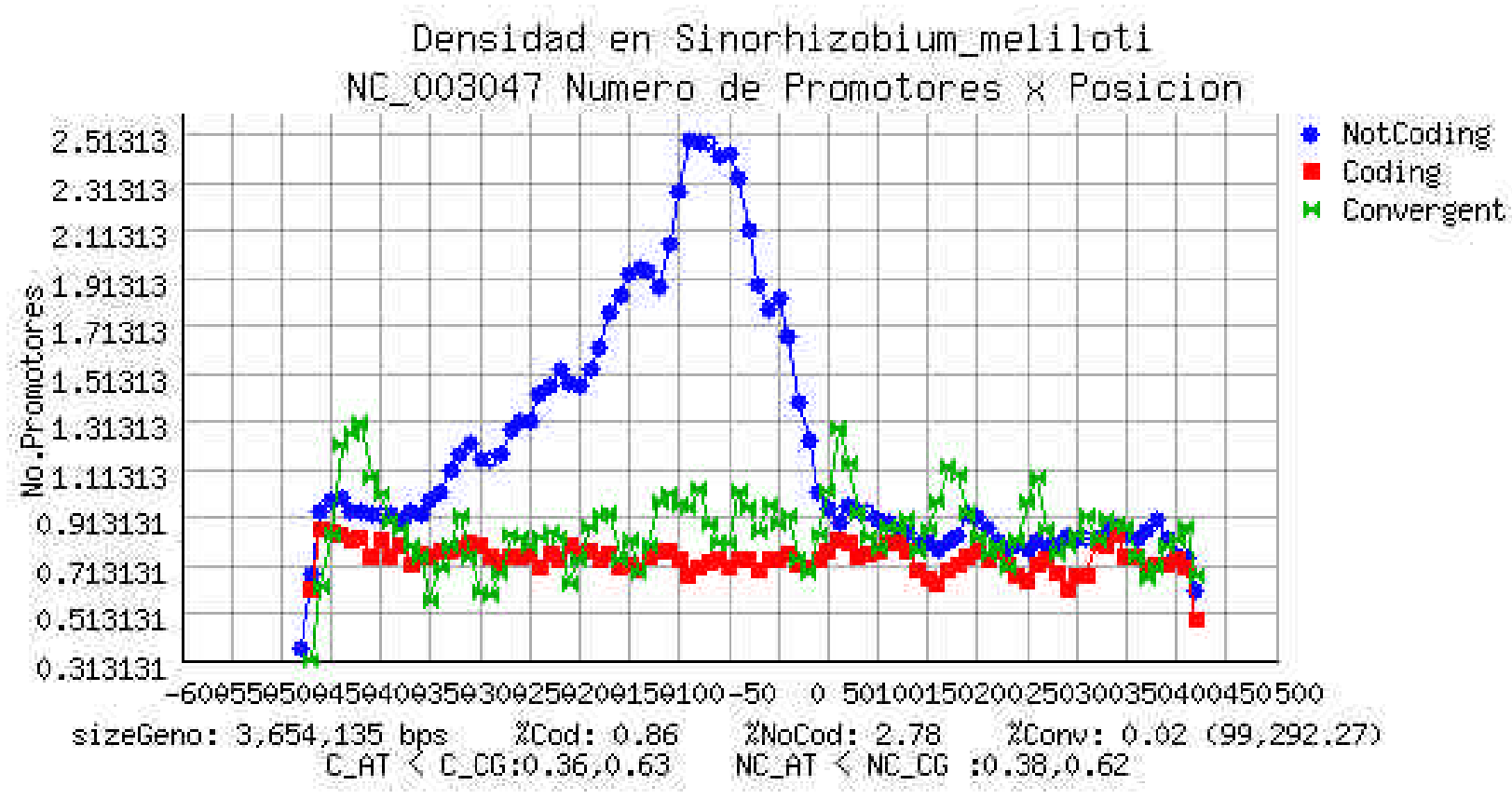
Densidad en Sinorhizobium_meliloti
NC_003047 Numero de Promotores x Posicion
No.Promotores
2.51313
2.31313
2.11313
1.91313
1.71313
1.51313
1.31313
1.11313
0.913131
0.713131
0.513131
0.313131
-600550500450400350300250200150100-50 0 50100150200250300350400450500
NotCoding
Coding
Convergent
sizeGeno: 3,654,135 bps %Cod: 0.86 %NoCod: 2.78 %Conv: 0.02 (99,292.27)
C_AT < C_CG:0.36,0.63 NC_AT < NC_CG :0.38,0.62

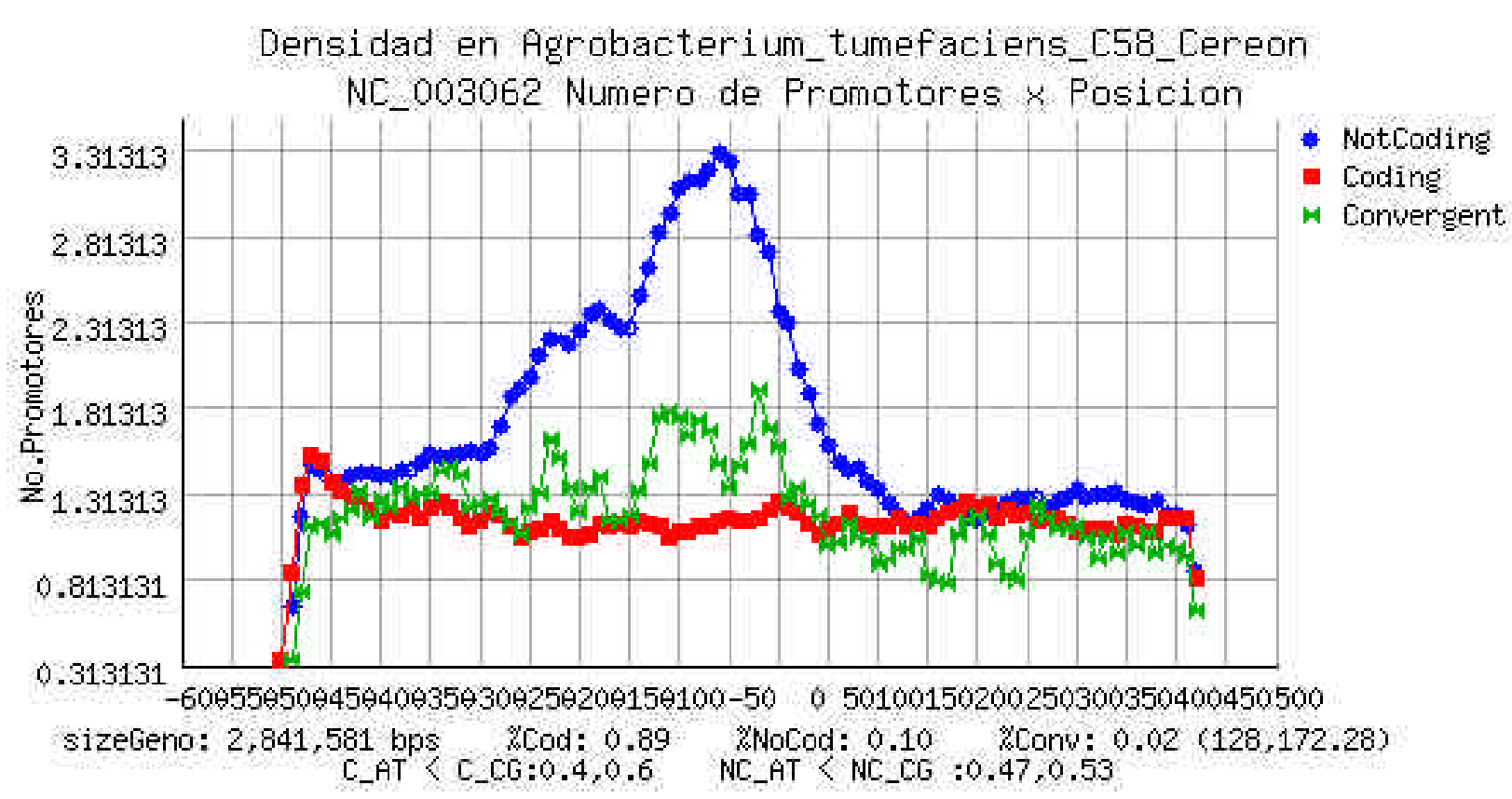
Densidad en Agrobacterium_tumefaciens_C58_Cereon
NC_003062 Numero de Promotores x Posicion
No.Promotores
3.31313
2.81313
2.31313
1.81313
1.31313
0.813131
0.313131
-600550500450400350300250200150100-50 0 50100150200250300350400450500
NotCoding
Coding
Convergent
sizeGeno: 2,841,581 bps %Cod: 0.89 %NoCod: 0.10 %Conv: 0.02 (128,172.28)
C_AT < C_CG:0.4,0.6 NC_AT < NC_CG :0.47,0.53

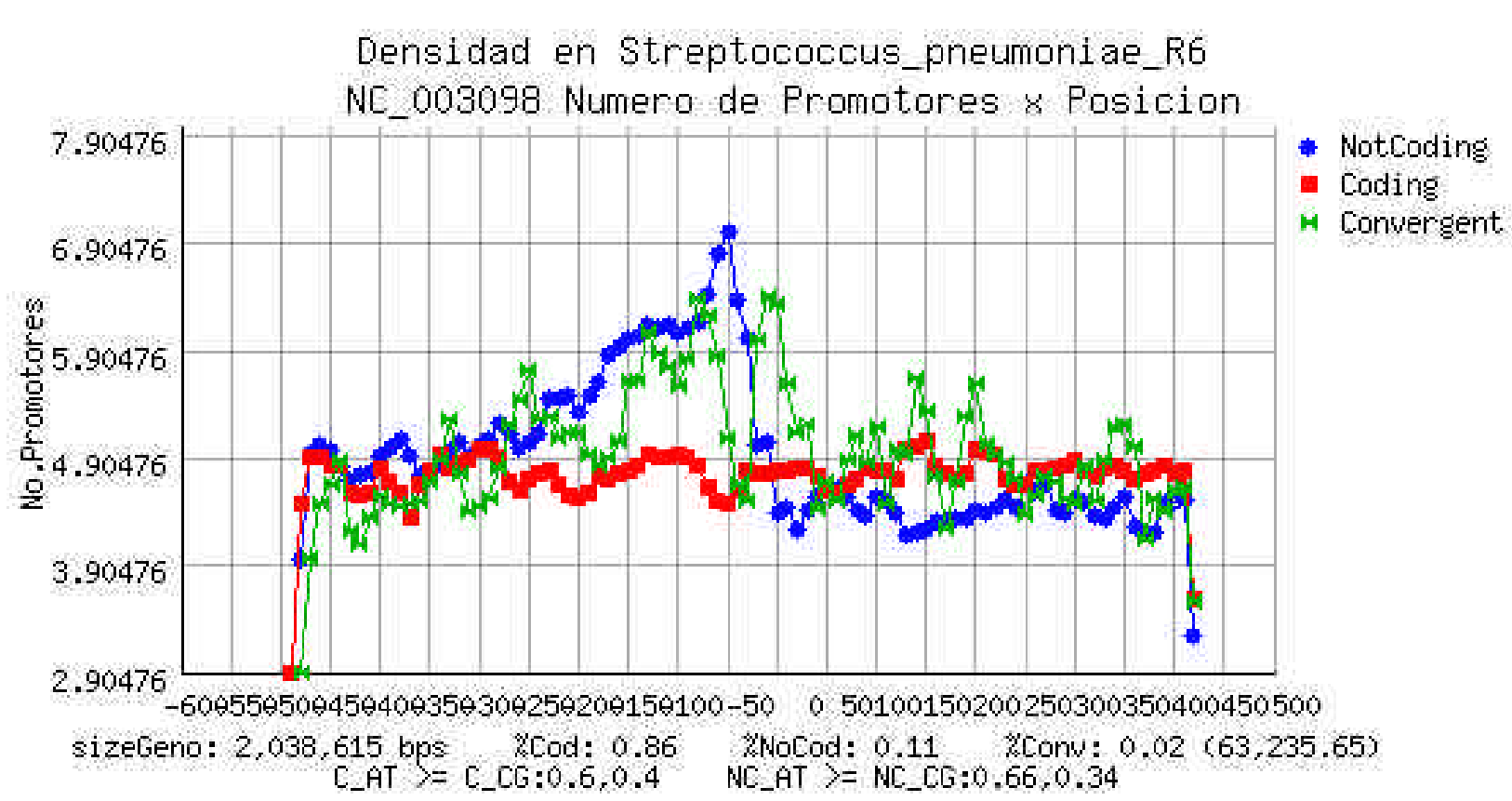
Densidad en Streptococcus_pneumoniae_R6
NC_003098 Numero de Promotores x Posicion
No.Promotores
7.90476
6.90476
5.90476
4.90476
3.90476
2.90476
-600550500450400350300250200150100-50 0 50100150200250300350400450500
NotCoding
Coding
Convergent
sizeGeno: 2,038,615 bps %Cod: 0.86 %NoCod: 0.11 %Conv: 0.02 (63,235.65)
C_AT >= C_CG:0.6,0.4 NC_AT >= NC_CG:0.66,0.34

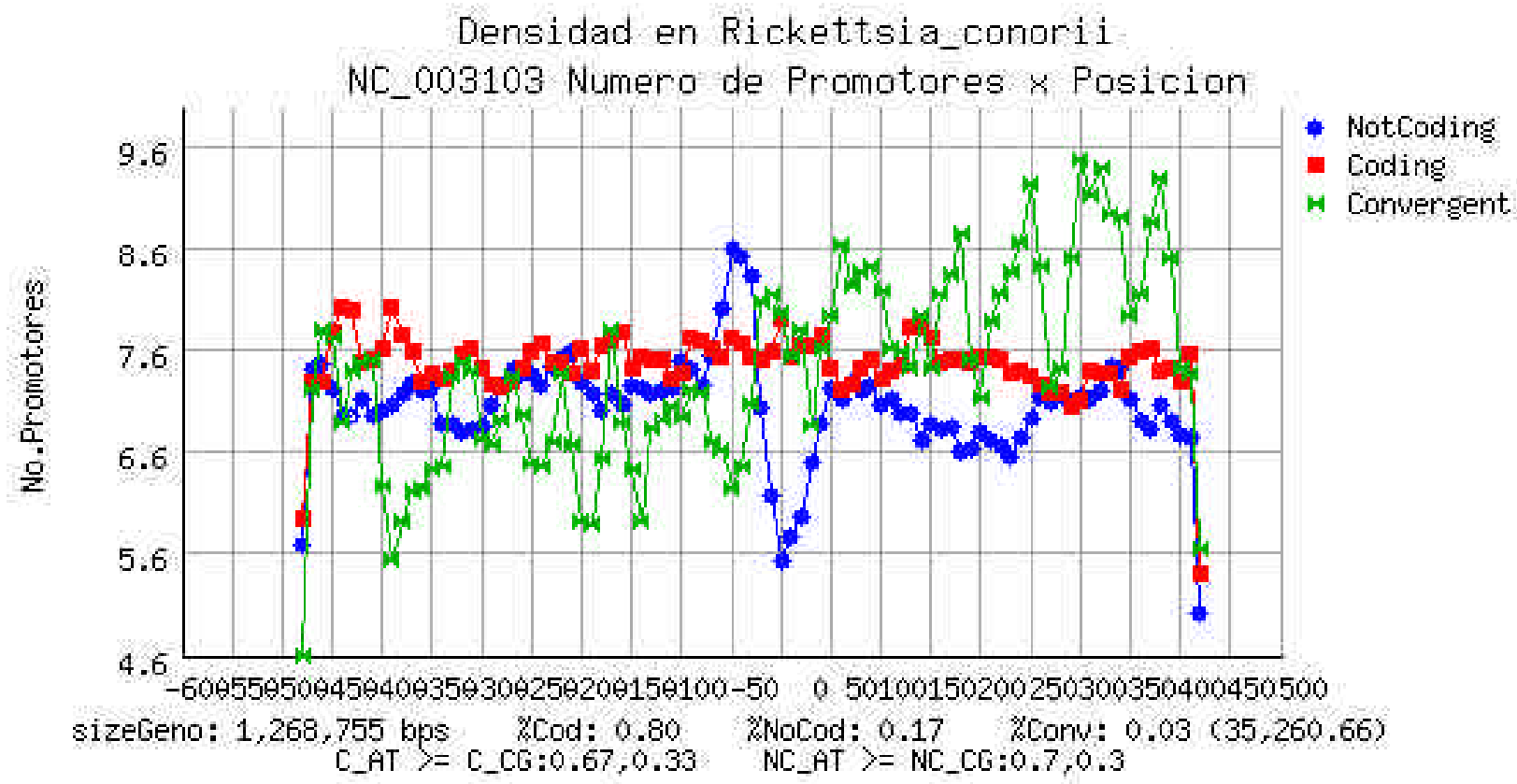
Densidad en Rickettsia_conorii
NC_003103 Numero de Promotores x Posicion
NotCoding
Coding
Convergent
No.Promotores
9.6
8.6
7.6
6.6
5.6
4.6
-600550500450400350300250200150100-50 0 50100150200250300350400450500
sizeGeno: 1,268,755 bps %Cod: 0.80 %NoCod: 0.17 %Conv: 0.03 (35,260.66)
C_AT >= C_CG:0.67,0.33 NC_AT >= NC_CG:0.7,0.3

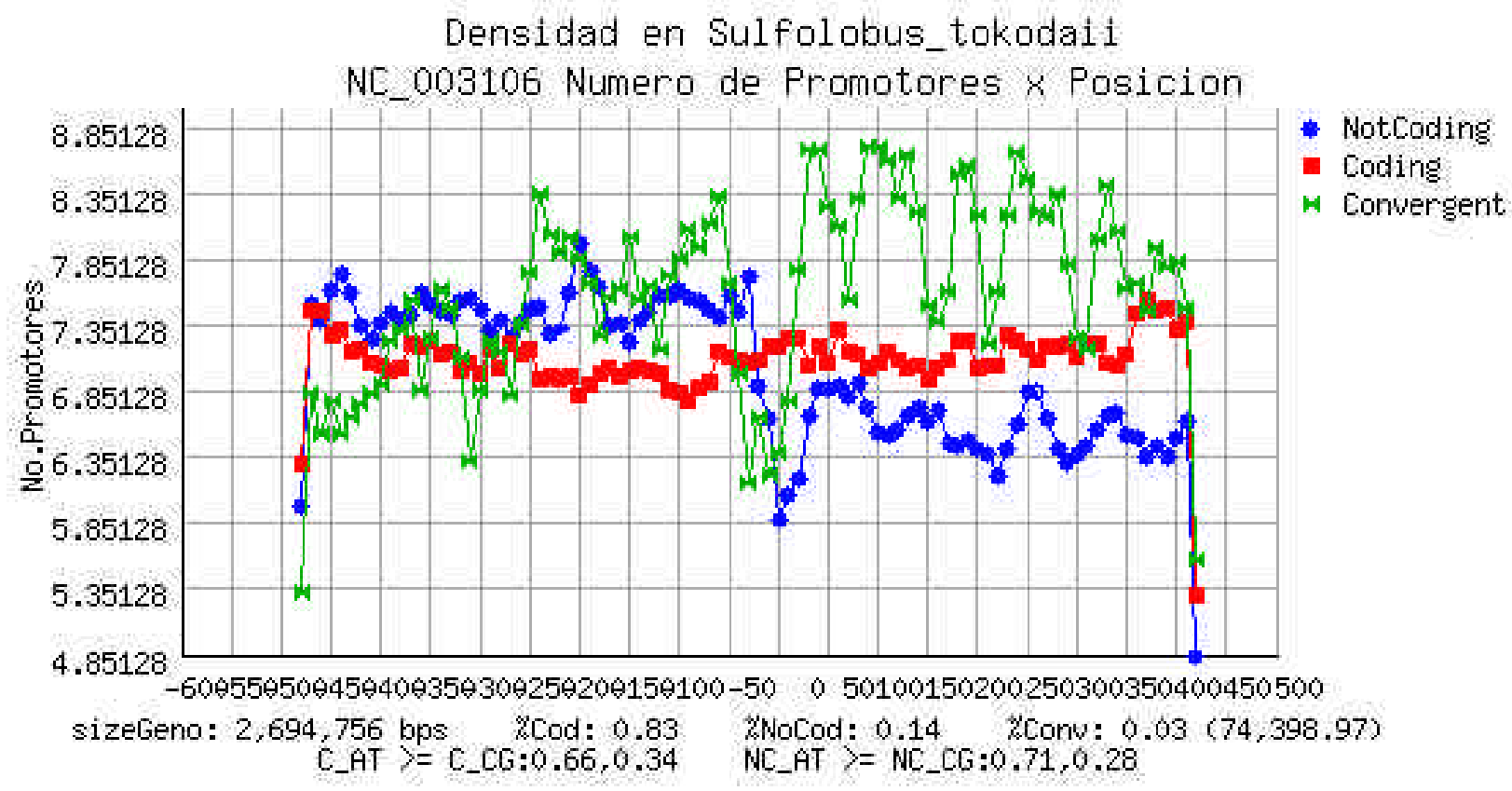
Densidad en Sulfolobus_tokodaii
NC_003106 Numero de Promotores x Posicion
NotCoding
Coding
Convergent
No.Promotores
8.85128
8.35128
7.85128
7.35128
6.85128
6.35128
5.85128
5.35128
4.85128
-600550500450400350300250200150100-50 0 50100150200250300350400450500
sizeGeno: 2,694,756 bps %Cod: 0.83 %NoCod: 0.14 %Conv: 0.03 (74,398.97)
C_AT >= C_CG:0.66,0.34 NC_AT >= NC_CG:0.71,0.28

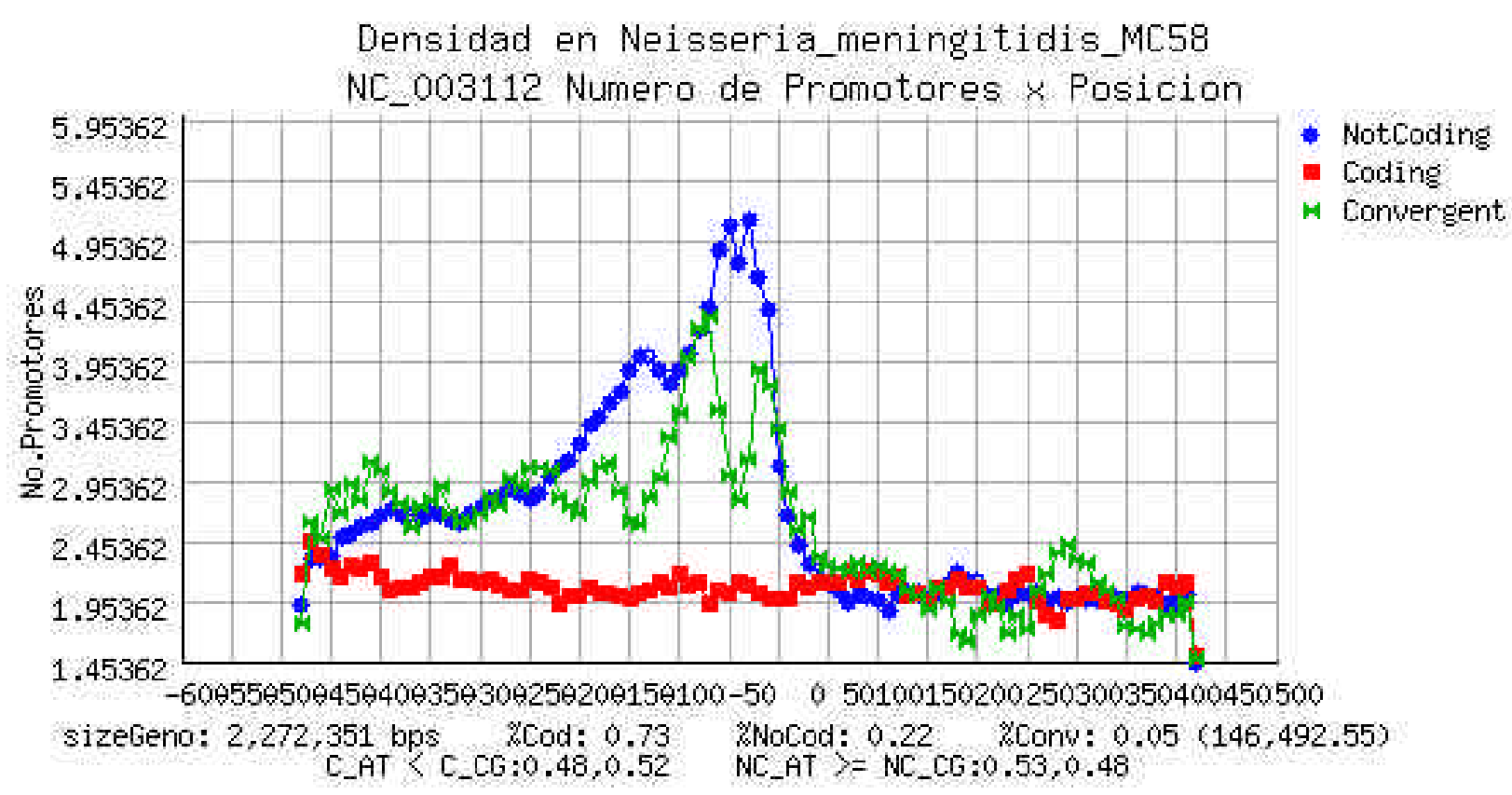
Densidad en Neisseria_meningitidis_MC58
NC_003112 Numero de Promotores x Posicion
NotCoding
Coding
Convergent
No.Promotores
5.95362
5.45362
4.95362
4.45362
3.95362
3.45362
2.95362
2.45362
1.95362
1.45362
-600550500450400350300250200150100-50 0 50100150200250300350400450500
sizeGeno: 2,272,351 bps %Cod: 0.73 %NoCod: 0.22 %Conv: 0.05 (146,492.55)
C_AT < C_CG:0.48,0.52 NC_AT >= NC_CG:0.53,0.48

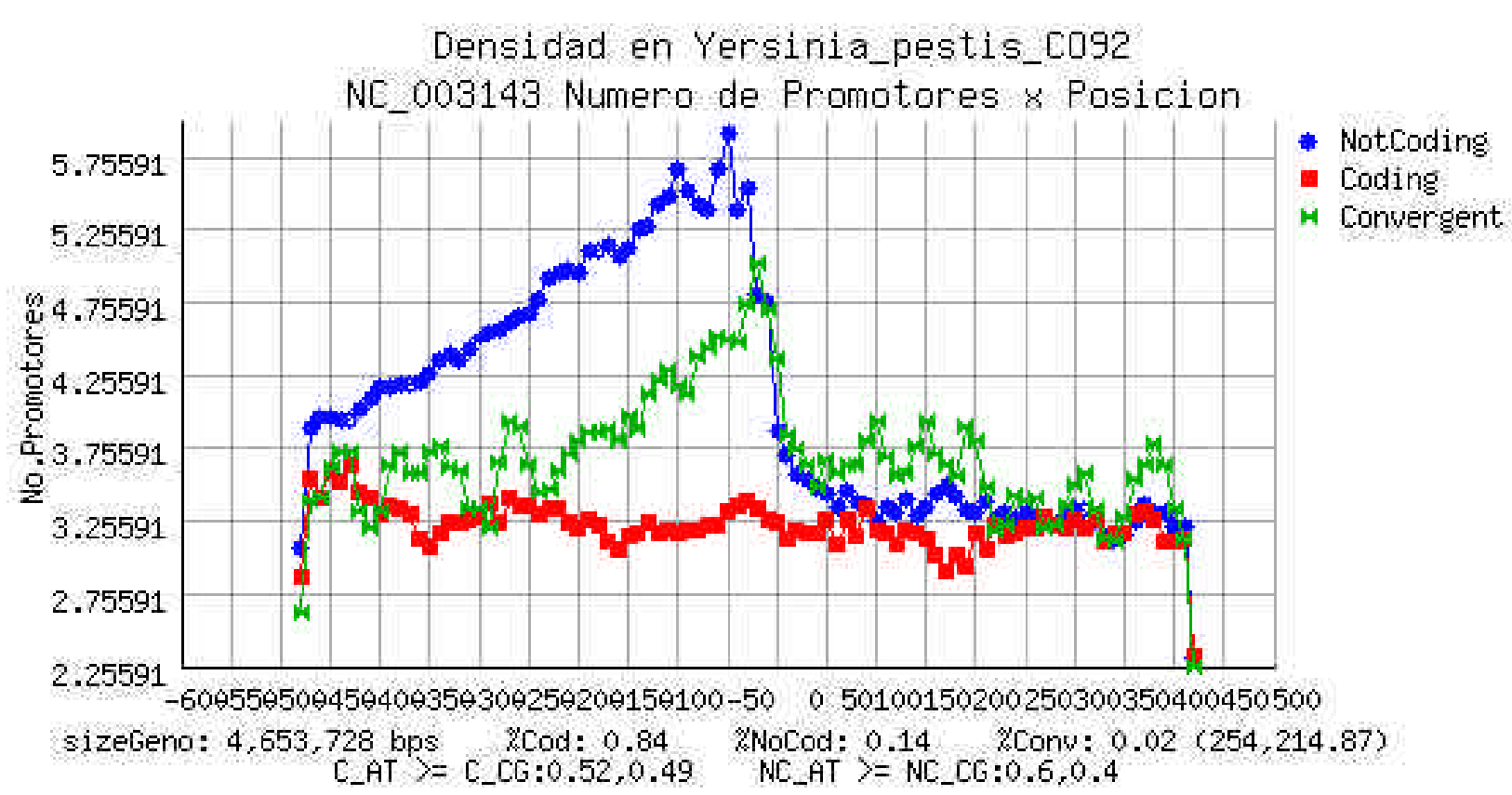
Densidad en Yersinia_pestis_CO92
NC_003143 Numero de Promotores x Posicion
NotCoding
Coding
Convergent
No.Promotores
5.75591
5.25591
4.75591
4.25591
3.75591
3.25591
2.75591
2.25591
-600550500450400350300250200150100-50 0 50100150200250300350400450500
sizeGeno: 4,653,728 bps %Cod: 0.84 %NoCod: 0.14 %Conv: 0.02 (254,214.87)
C_AT >= C_CG:0.52,0.49 NC_AT >= NC_CG:0.6,0.4

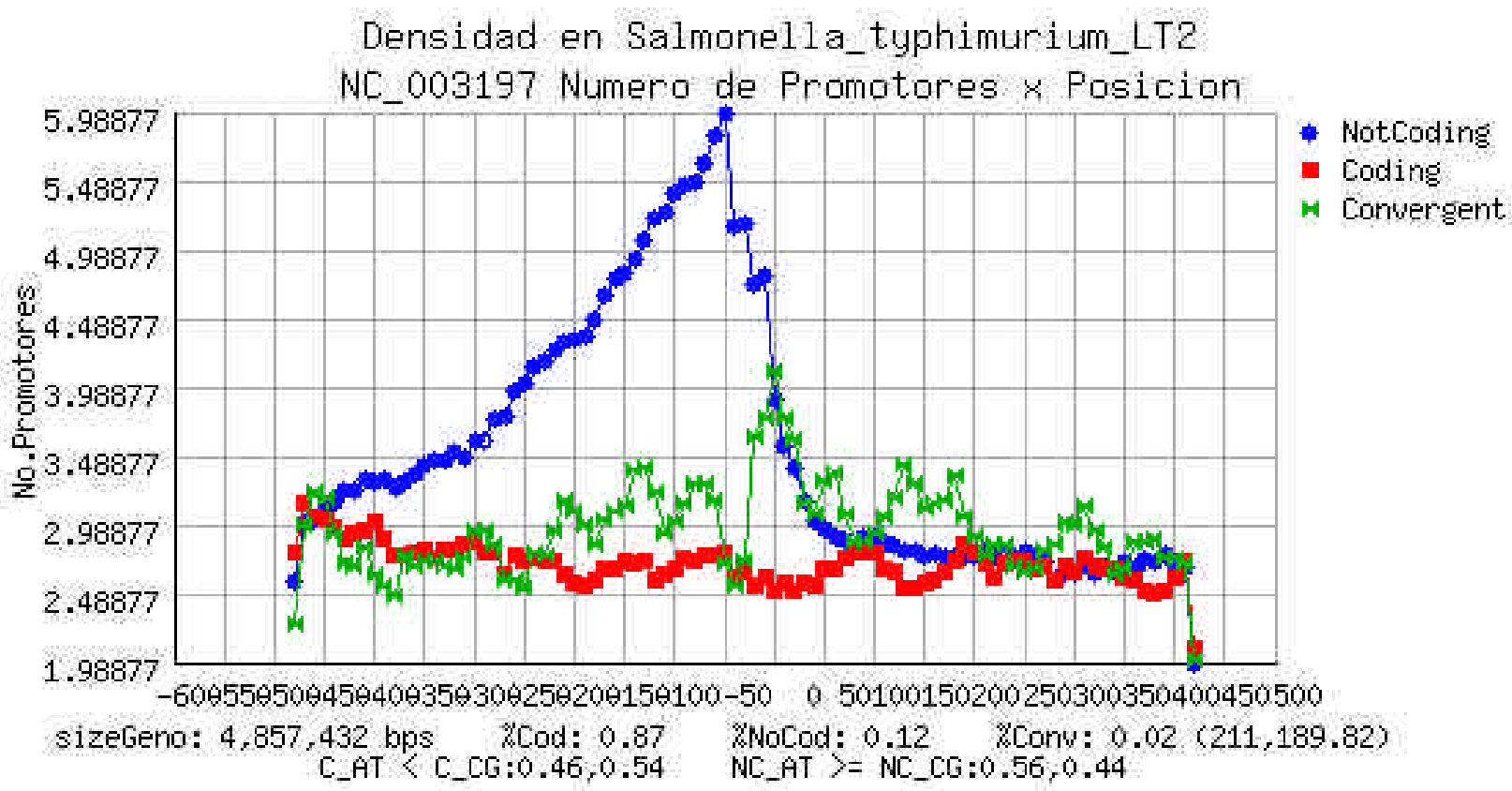
Densidad en Salmonella_typhimurium_LT2
NC_003197 Numero de Promotores x Posicion
No.Promotores
5.98877
5.48877
4.98877
4.48877
3.98877
3.48877
2.98877
2.48877
1.98877
NotCoding
Coding
Convergent
-600550500450400350300250200150100-50 0 50100150200250300350400450500
sizeGeno: 4,857,432 bps %Cod: 0.87 %NoCod: 0.12 %Conv: 0.02 (211,189.82)
C_AT < C_CG:0.46,0.54 NC_AT >= NC_CG:0.56,0.44

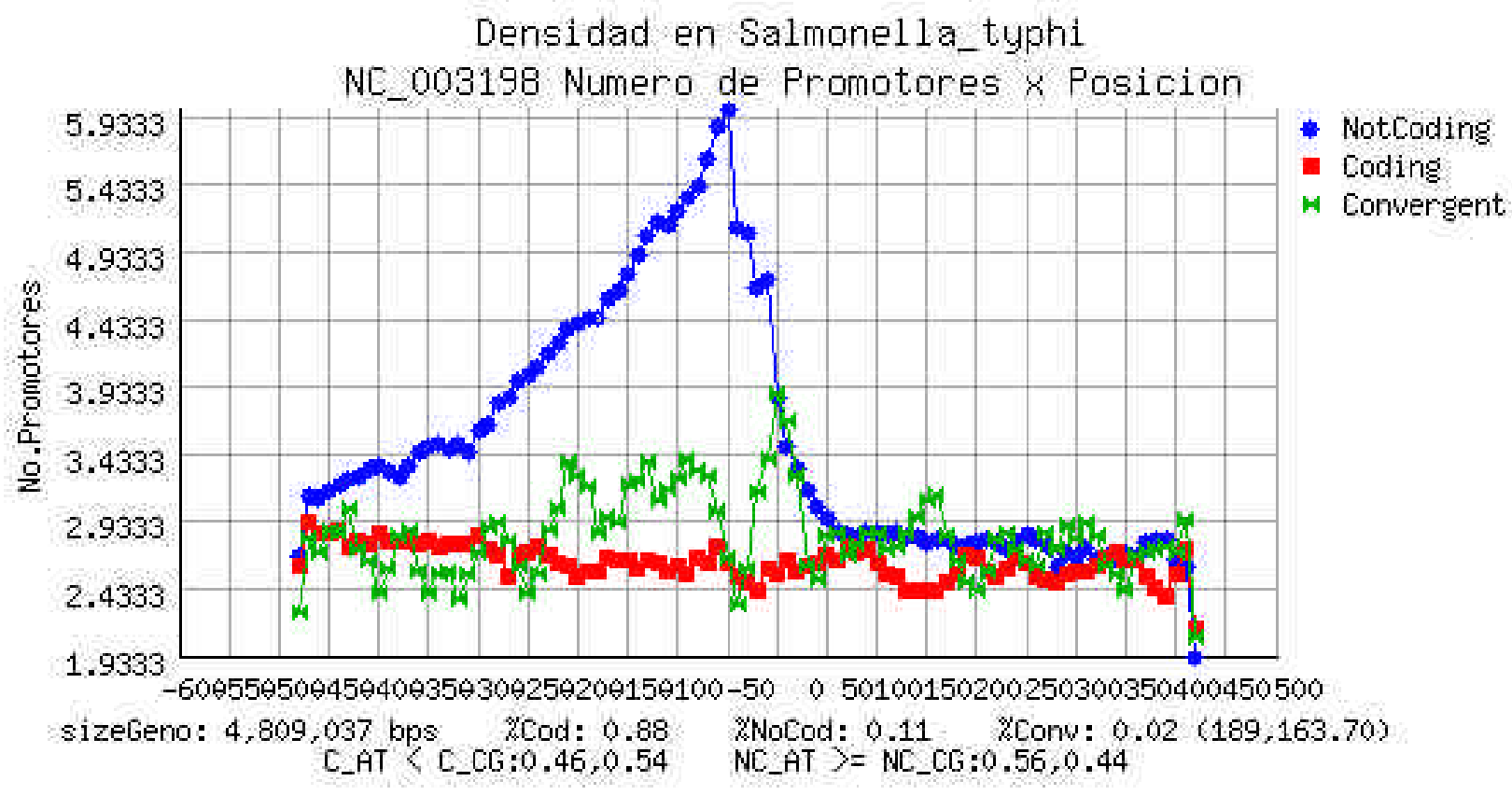
Densidad en Salmonella_typhi
NC_003198 Numero de Promotores x Posicion
No.Promotores
5.9333
5.4333
4.9333
4.4333
3.9333
3.4333
2.9333
2.4333
1.9333
NotCoding
Coding
Convergent
-600550500450400350300250200150100-50 0 50100150200250300350400450500
sizeGeno: 4,809,037 bps %Cod: 0.88 %NoCod: 0.11 %Conv: 0.02 (189,163.70)
C_AT < C_CG:0.46,0.54 NC_AT >= NC_CG:0.56,0.44

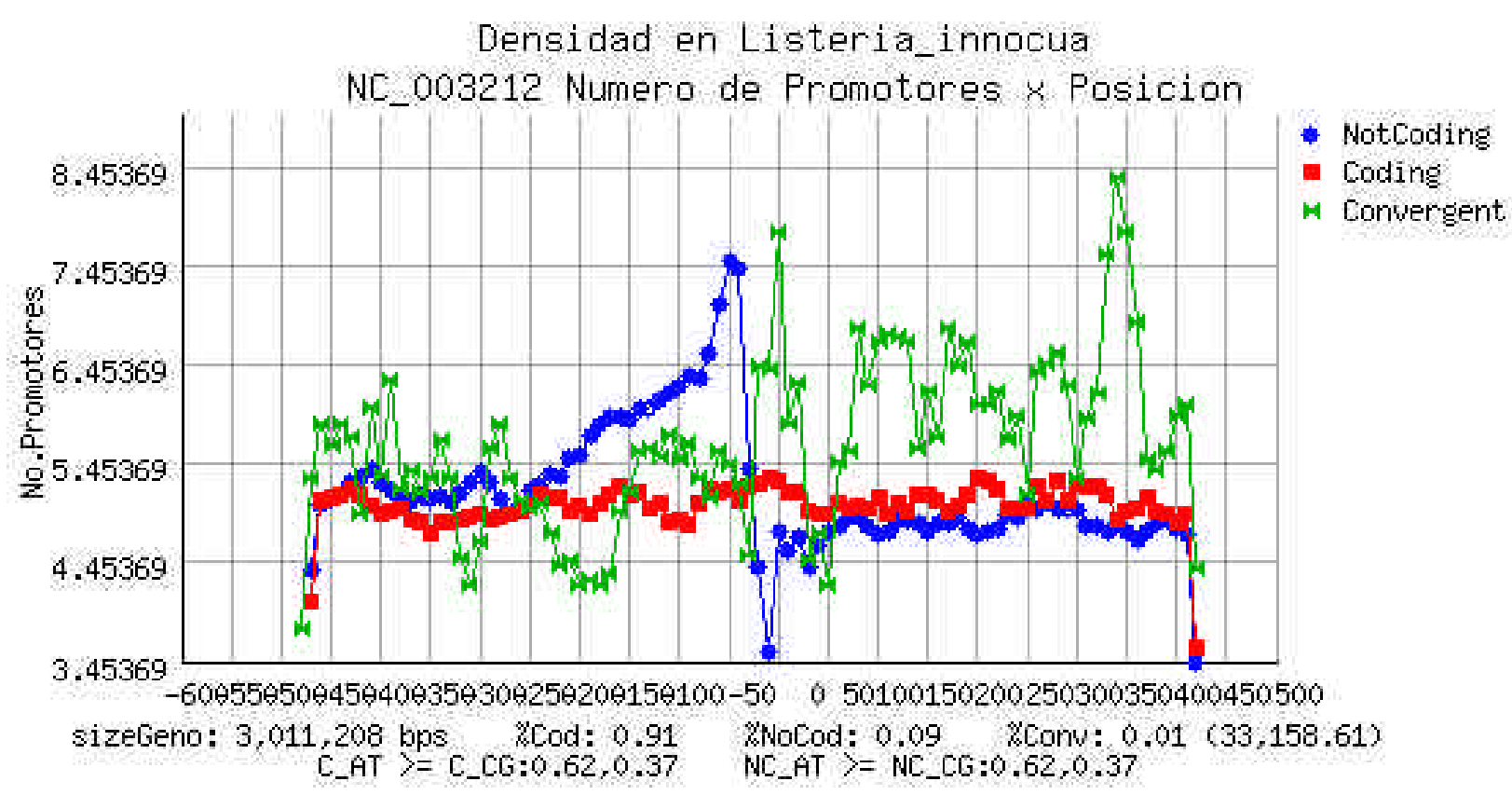
Densidad en Listeria_innocua
NC_003212 Numero de Promotores x Posicion
No.Promotores
8.45369
7.45369
6.45369
5.45369
4.45369
3.45369
NotCoding
Coding
Convergent
-600550500450400350300250200150100-50 0 50100150200250300350400450500
sizeGeno: 3,011,208 bps %Cod: 0.91 %NoCod: 0.09 %Conv: 0.01 (33,158.61)
C_AT >= C_CG:0.62,0.37 NC_AT >= NC_CG:0.62,0.37

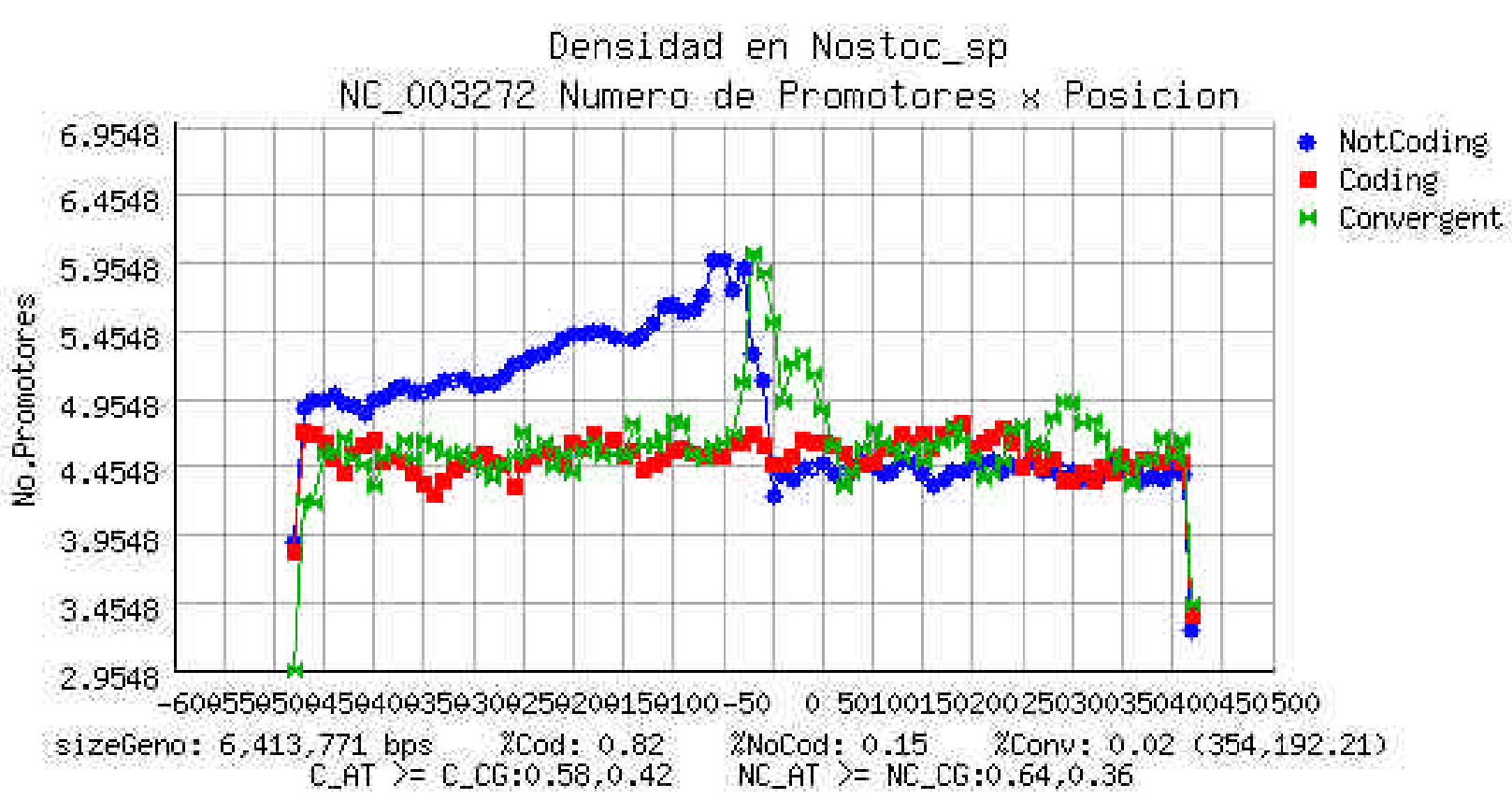
Densidad en Nostoc_sp
NC_003272 Numero de Promotores x Posicion
No.Promotores
6.9548
6.4548
5.9548
5.4548
4.9548
4.4548
3.9548
3.4548
2.9548
NotCoding
Coding
Convergent
-600550500450400350300250200150100-50 0 50100150200250300350400450500
sizeGeno: 6,413,771 bps %Cod: 0.82 %NoCod: 0.15 %Conv: 0.02 (354,192.21)
C_AT >= C_CG:0.58,0.42 NC_AT >= NC_CG:0.64,0.36

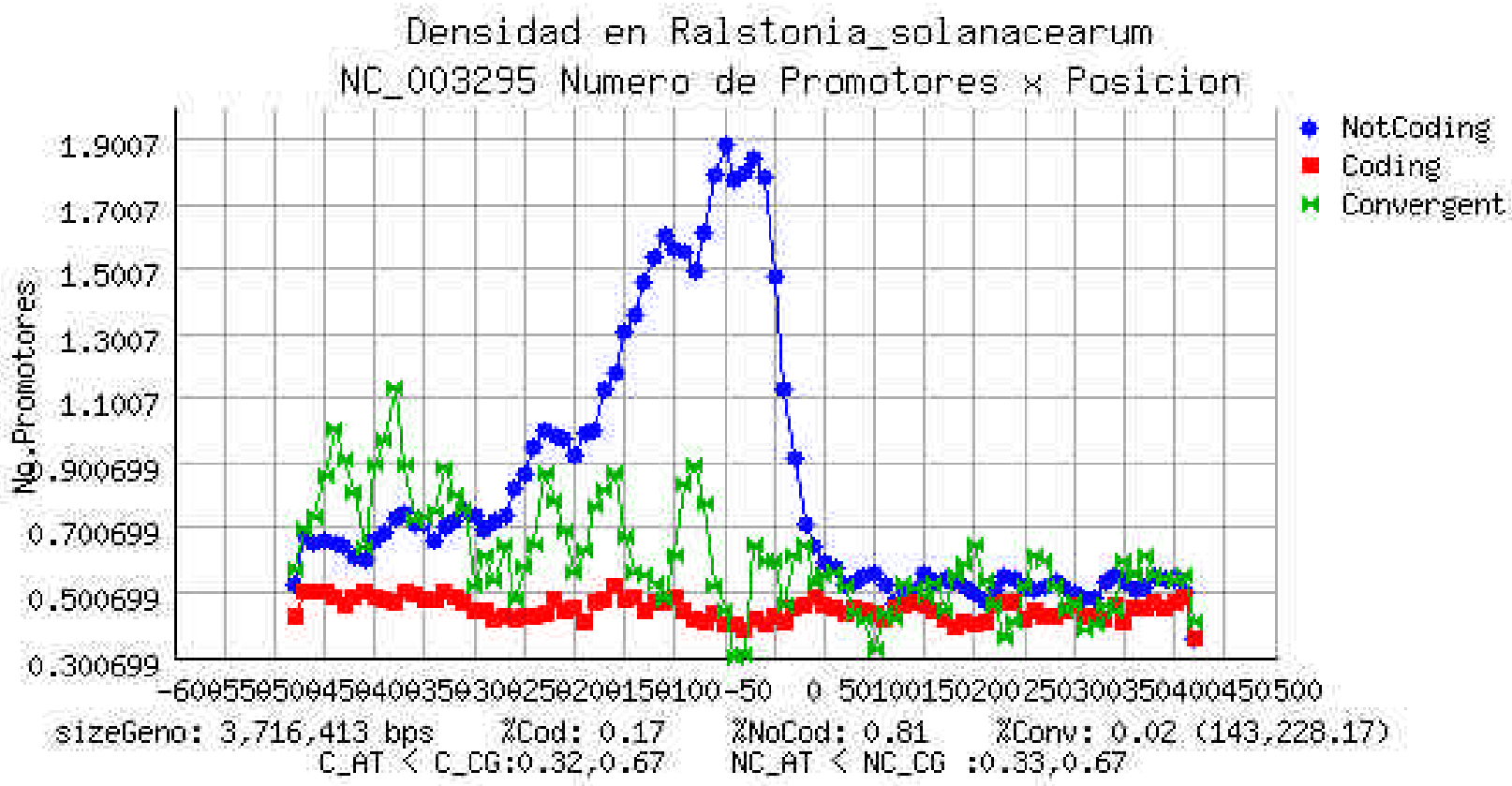
Densidad en Ralstonia_solanacearum
NC_003295 Numero de Promotores x Posicion
No.Promotores
NotCoding
Coding
Convergent
sizeGeno: 3,716,413 bps %Cod: 0.17 %NoCod: 0.81 %Conv: 0.02 (143,228.17)
C_AT < C_CG:0.32,0.67 NC_AT < NC_CG :0.33,0.67

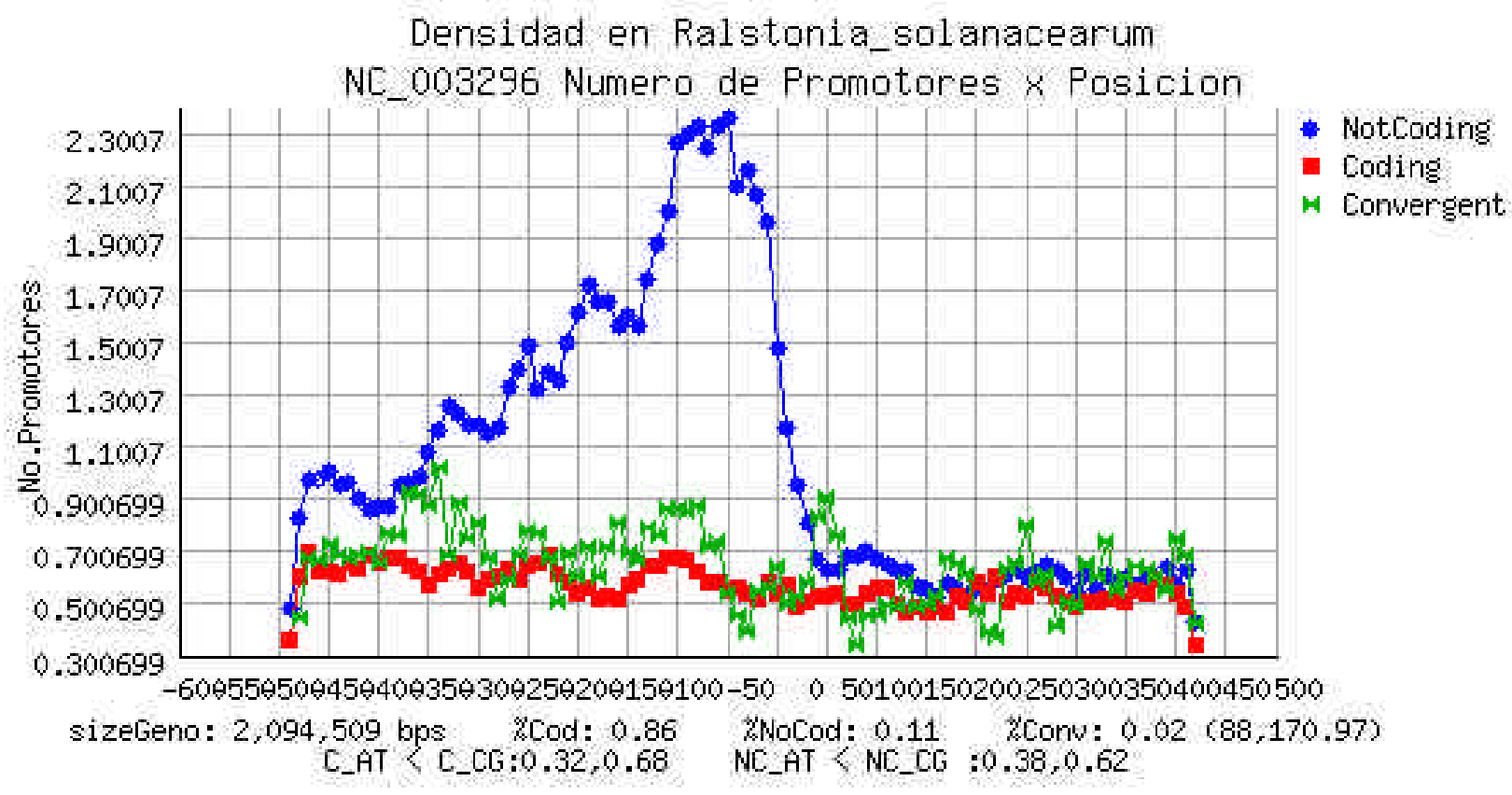
Densidad en Ralstonia_solanacearum
NC_003296 Numero de Promotores x Posicion
No.Promotores
NotCoding
Coding
Convergent
sizeGeno: 2,094,509 bps %Cod: 0.86 %NoCod: 0.11 %Conv: 0.02 (88,170.97)
C_AT < C_CG:0.32,0.68 NC_AT < NC_CG :0.38,0.62

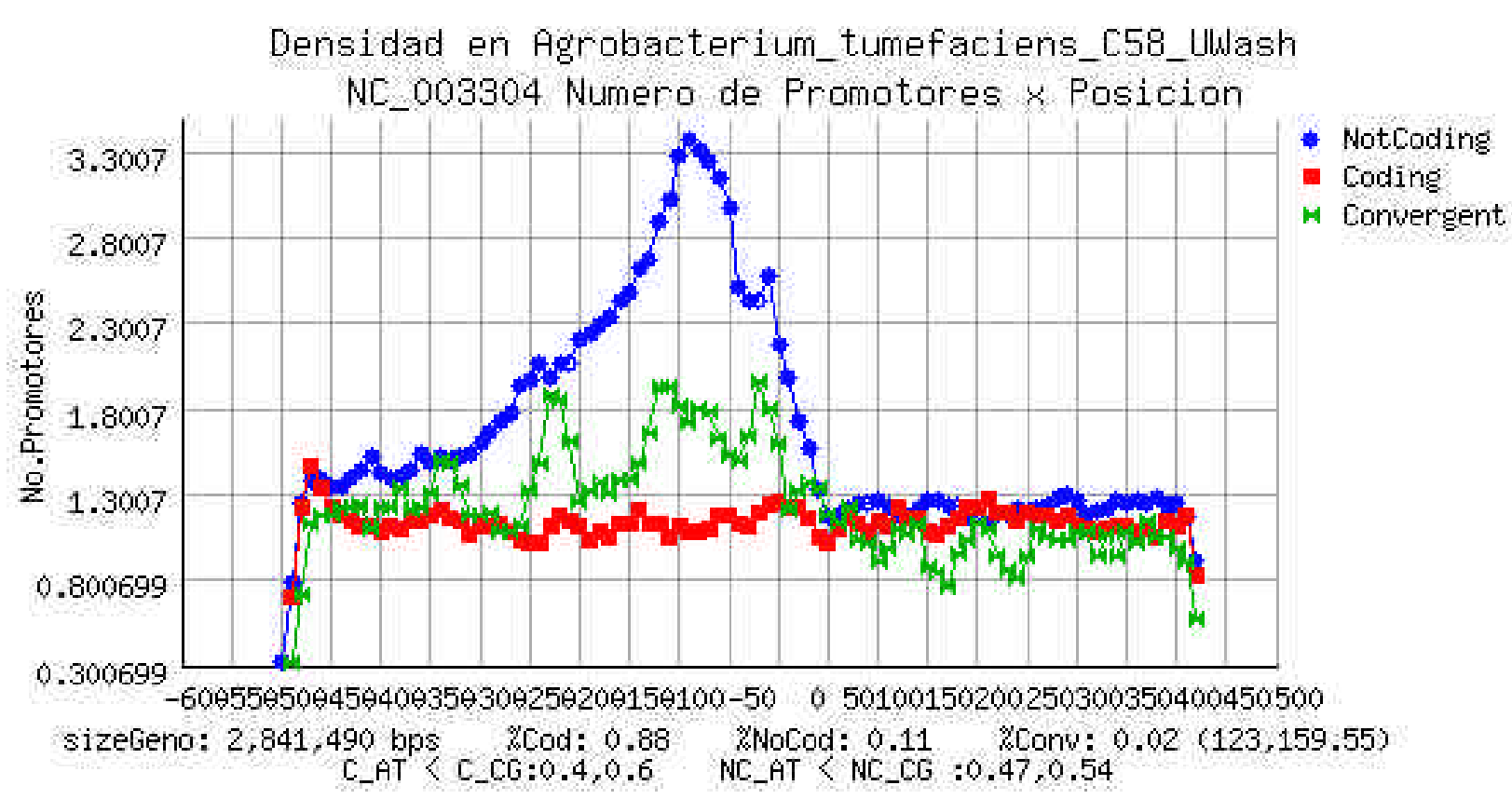
Densidad en Agrobacterium_tumefaciens_C58_UWash
NC_003304 Numero de Promotores x Posicion
No.Promotores
NotCoding
Coding
Convergent
sizeGeno: 2,841,490 bps %Cod: 0.88 %NoCod: 0.11 %Conv: 0.02 (123,159.55)
C_AT < C_CG:0.4,0.6 NC_AT < NC_CG :0.47,0.54

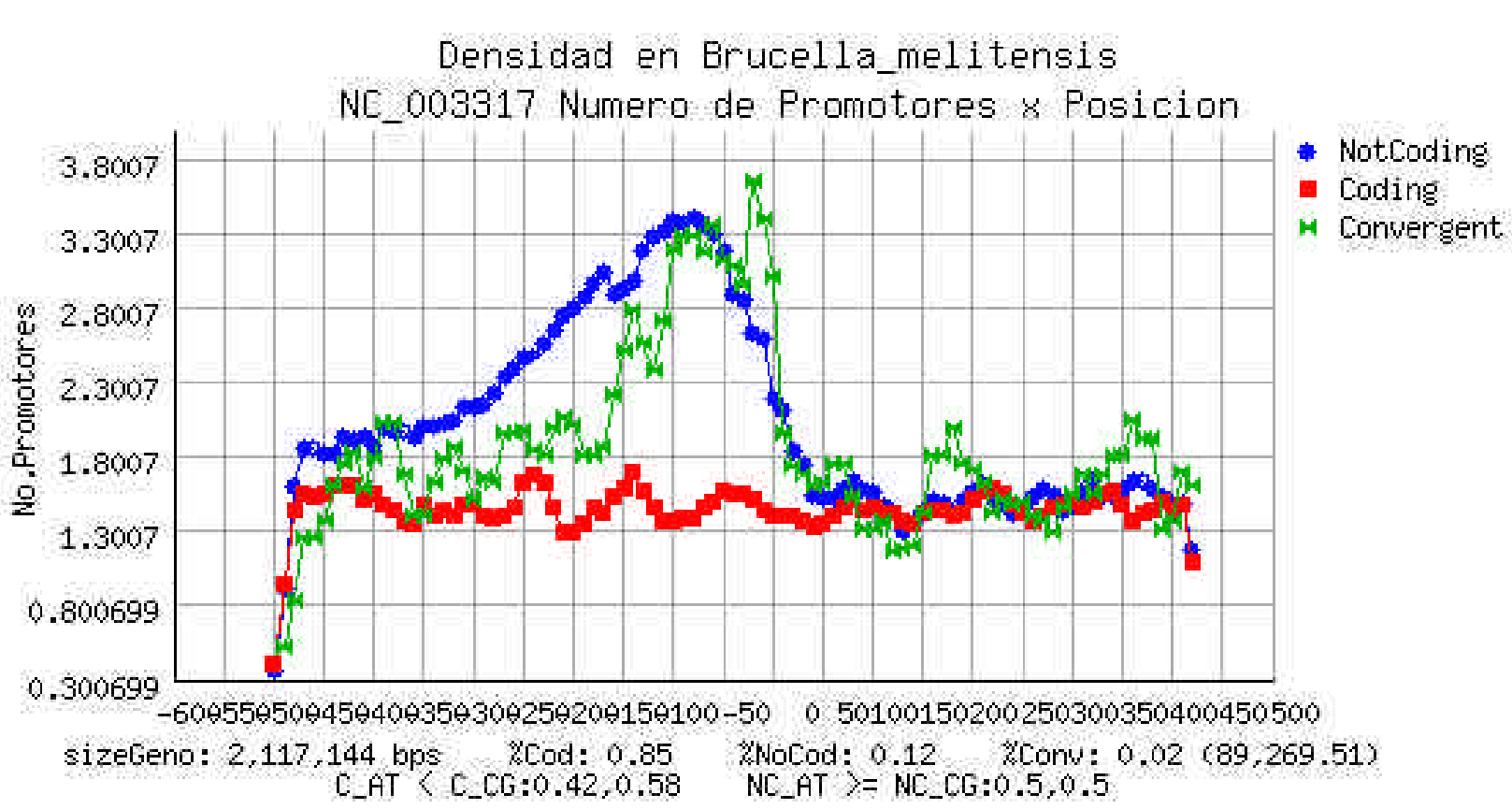
Densidad en Brucella_melitensis
NC_003317 Numero de Promotores x Posicion
No.Promotores
NotCoding
Coding
Convergent
sizeGeno: 2,117,144 bps %Cod: 0.85 %NoCod: 0.12 %Conv: 0.02 (89,269.51)
C_AT < C_CG:0.42,0.58 NC_AT >= NC_CG:0.5,0.5

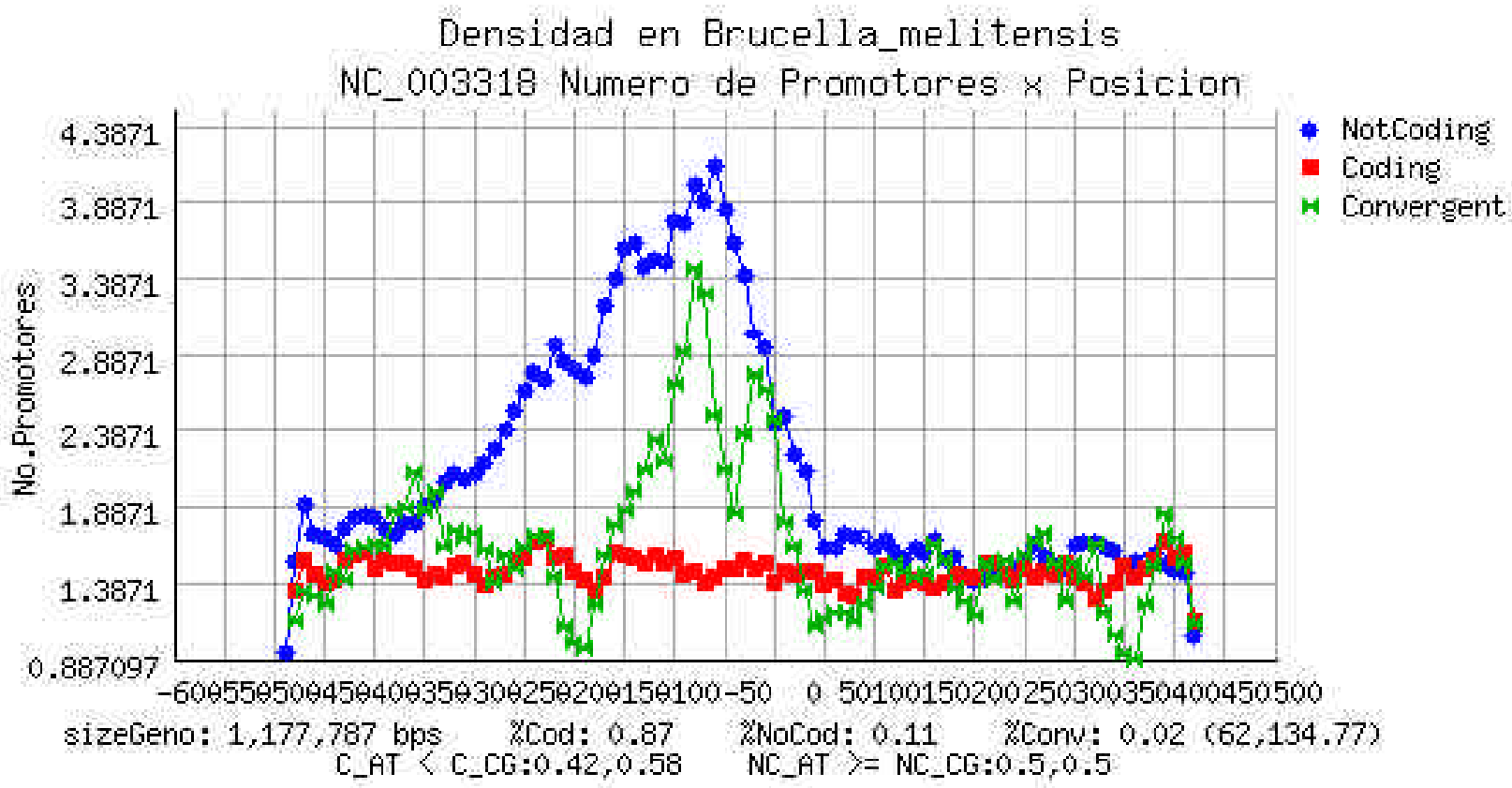
Densidad en Brucella_melitensis
NC_003318 Numero de Promotores x Posicion
No.Promotores
4.3871
3.8871
3.3871
2.8871
2.3871
1.8871
1.3871
0.887097
-600550500450400350300250200150100-50 0 50100150200250300350400450500
NotCoding
Coding
Convergent
sizeGeno: 1,177,787 bps %Cod: 0.87 %NoCod: 0.11 %Conv: 0.02 (62,134.77)
C_AT < C_CG:0.42,0.58 NC_AT >= NC_CG:0.5,0.5

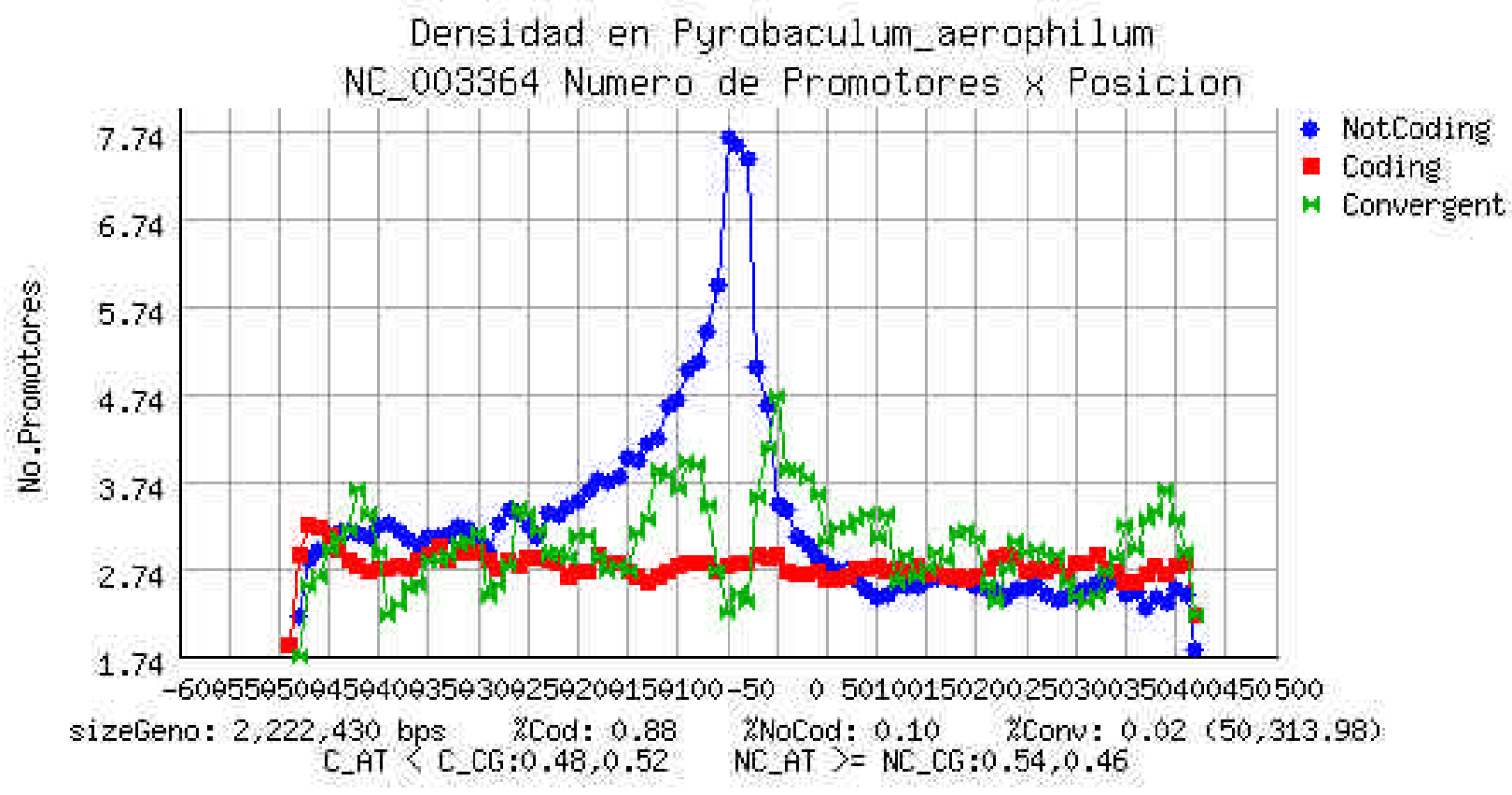
Densidad en Pyrobaculum_aerophilum
NC_003364 Numero de Promotores x Posicion
No.Promotores
7.74
6.74
5.74
4.74
3.74
2.74
1.74
-600550500450400350300250200150100-50 0 50100150200250300350400450500
NotCoding
Coding
Convergent
sizeGeno: 2,222,430 bps %Cod: 0.88 %NoCod: 0.10 %Conv: 0.02 (50,313.98)
C_AT < C_CG:0.48,0.52 NC_AT >= NC_CG:0.54,0.46

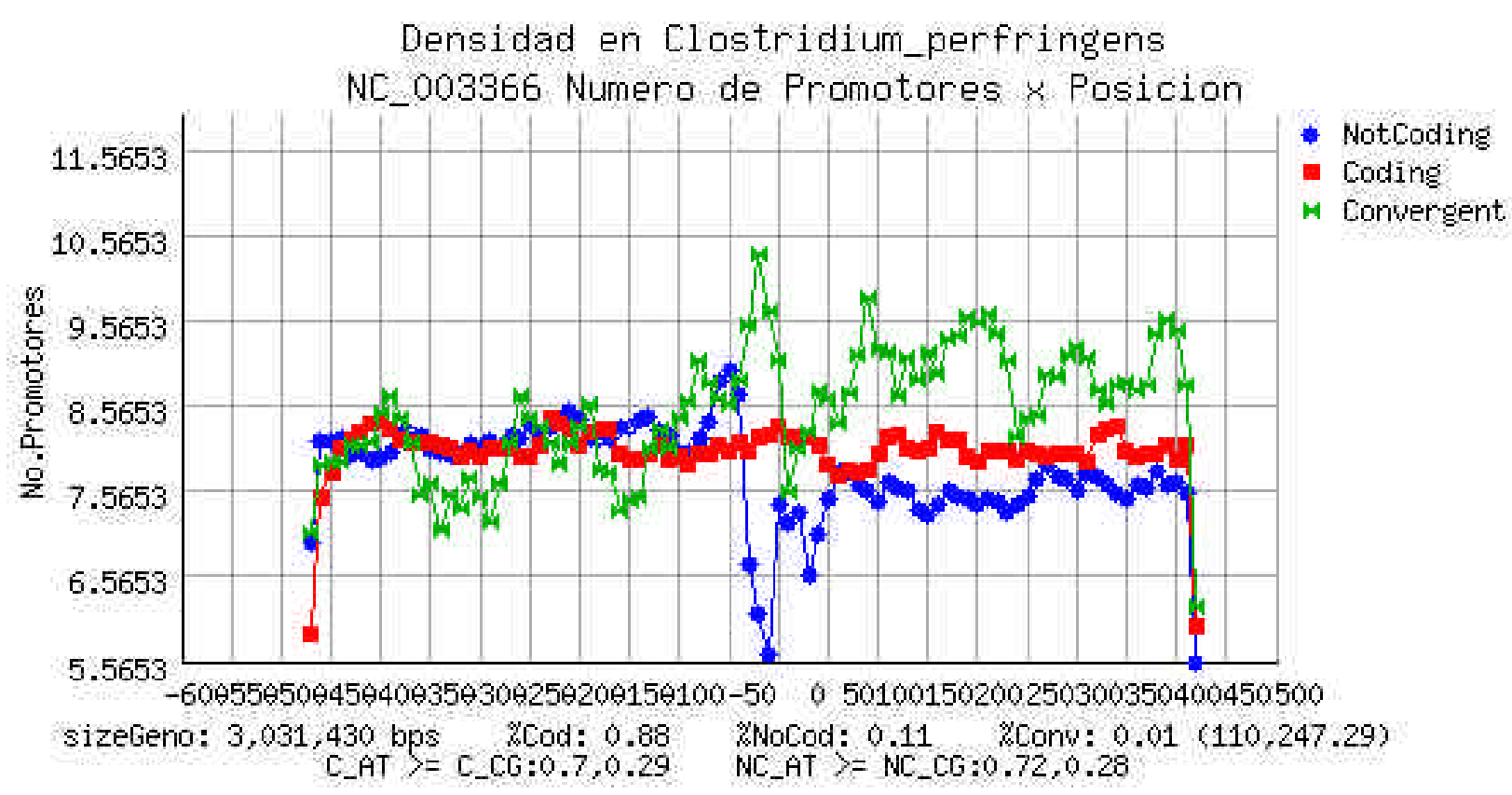
Densidad en Clostridium_perfringens
NC_003366 Numero de Promotores x Posicion
No.Promotores
11.5653
10.5653
9.5653
8.5653
7.5653
6.5653
5.5653
-600550500450400350300250200150100-50 0 50100150200250300350400450500
NotCoding
Coding
Convergent
sizeGeno: 3,031,430 bps %Cod: 0.88 %NoCod: 0.11 %Conv: 0.01 (110,247.29)
C_AT >= C_CG:0.7,0.29 NC_AT >= NC_CG:0.72,0.28

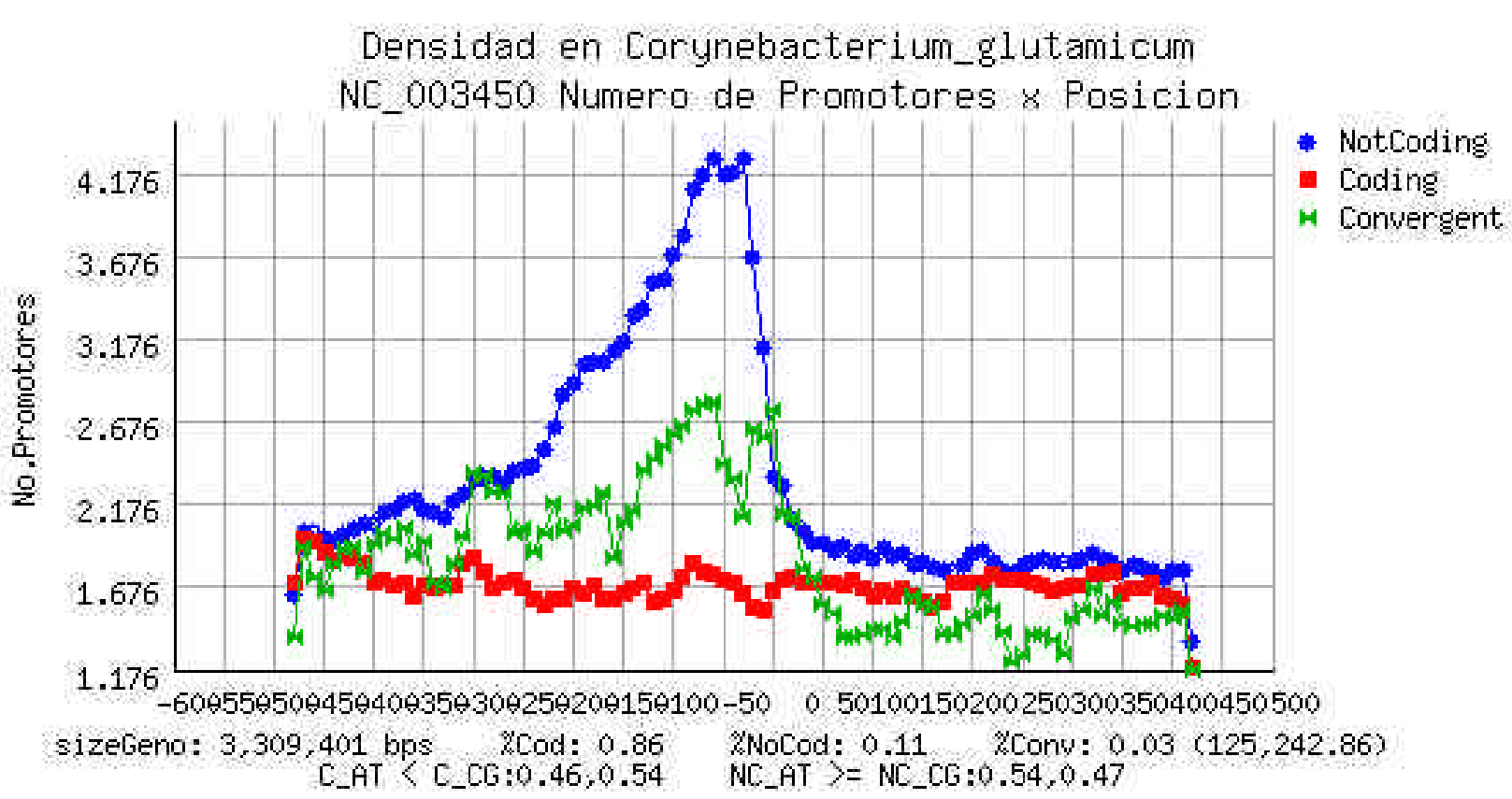
Densidad en Corynebacterium_glutamicum
NC_003450 Numero de Promotores x Posicion
No.Promotores
4.176
3.676
3.176
2.676
2.176
1.676
1.176
-600550500450400350300250200150100-50 0 50100150200250300350400450500
NotCoding
Coding
Convergent
sizeGeno: 3,309,401 bps %Cod: 0.86 %NoCod: 0.11 %Conv: 0.03 (125,242.86)
C_AT < C_CG:0.46,0.54 NC_AT >= NC_CG:0.54,0.47

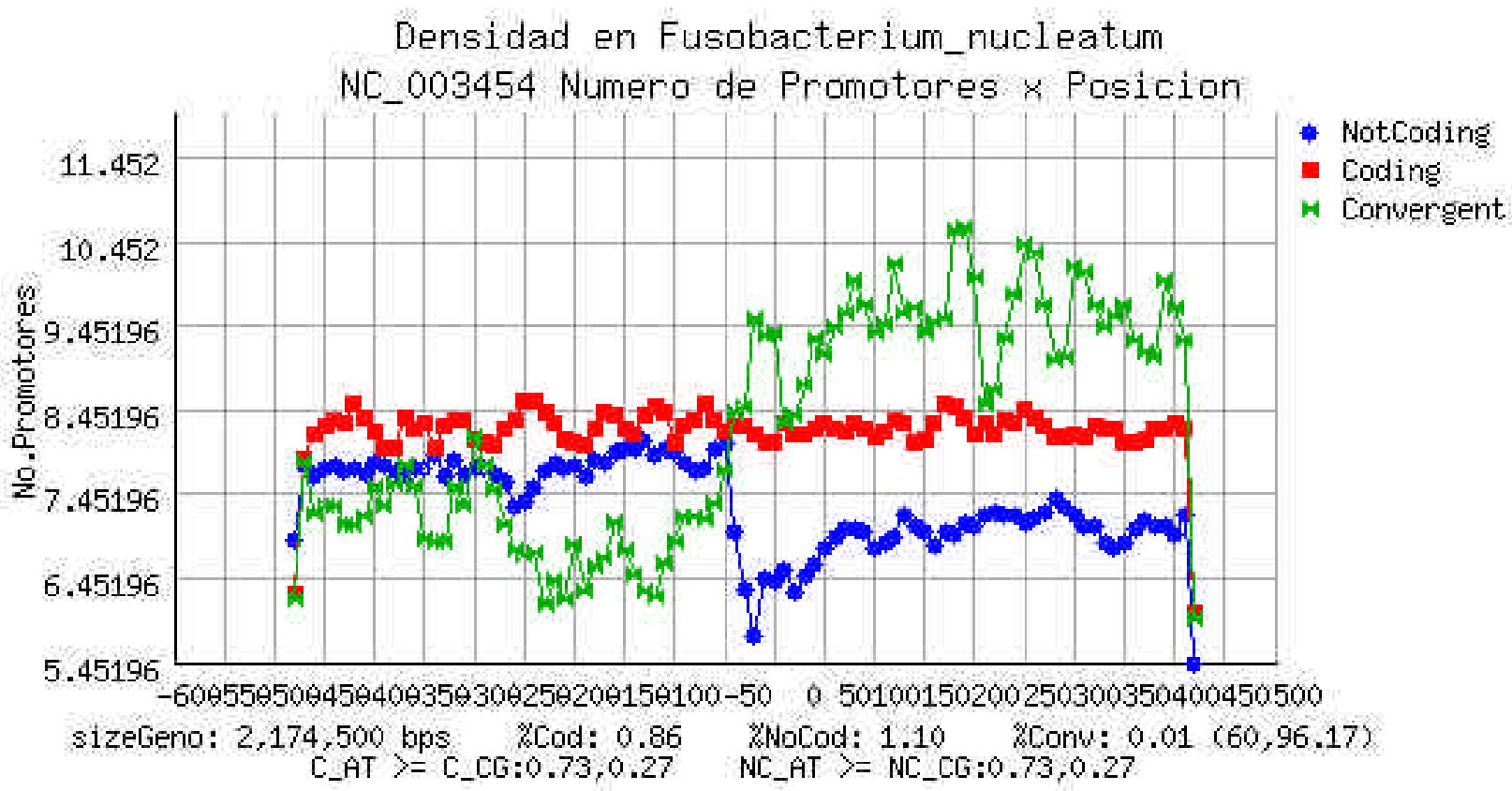
Densidad en Fusobacterium_nucleatum
NC_003454 Numero de Promotores x Posicion
NotCoding
Coding
Convergent
No.Promotores
11.452
10.452
9.45196
8.45196
7.45196
6.45196
5.45196
-600550500450400350300250200150100-50 0 50100150200250300350400450500
sizeGeno: 2,174,500 bps %Cod: 0.86 %NoCod: 1.10 %Conv: 0.01 (60,96.17)
C_AT >= C_CG:0.73,0.27 NC_AT >= NC_CG:0.73,0.27

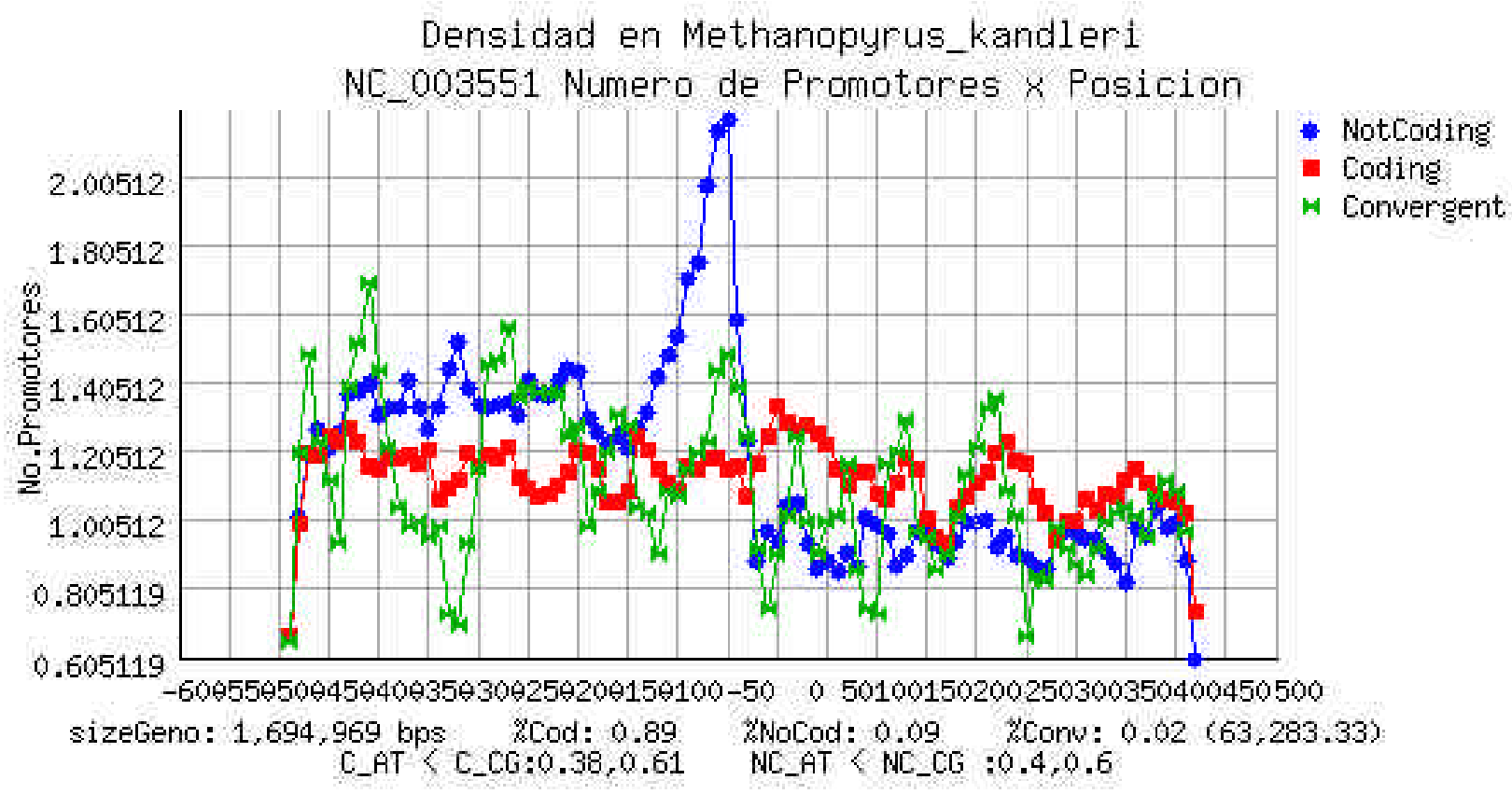
Densidad en Methanopyrus_kandleri
NC_003551 Numero de Promotores x Posicion
NotCoding
Coding
Convergent
No.Promotores
2.00512
1.80512
1.60512
1.40512
1.20512
1.00512
0.805119
0.605119
-600550500450400350300250200150100-50 0 50100150200250300350400450500
sizeGeno: 1,694,969 bps %Cod: 0.89 %NoCod: 0.09 %Conv: 0.02 (63,283.33)
C_AT < C_CG:0.38,0.61 NC_AT < NC_CG :0.4,0.6

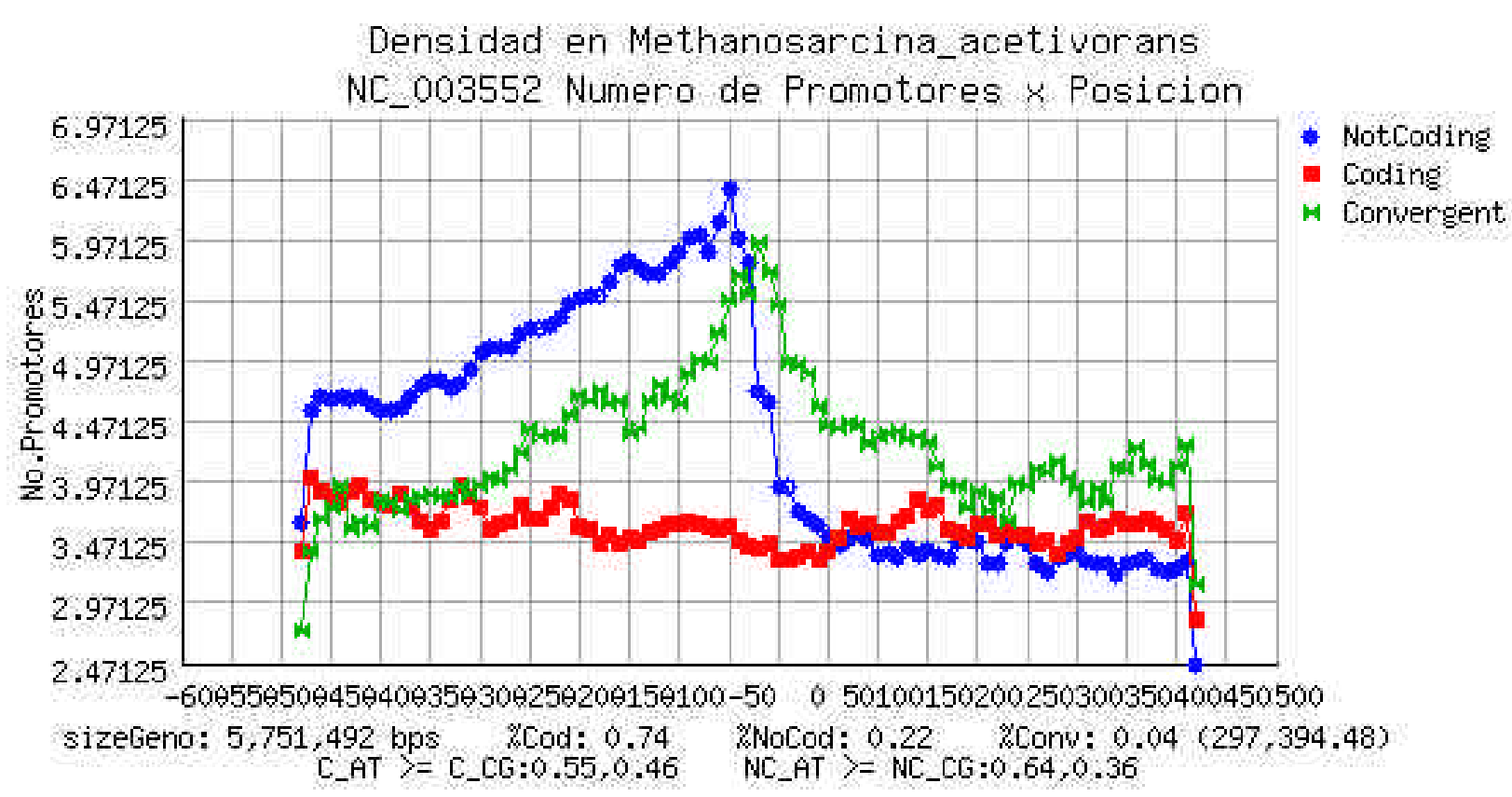
Densidad en Methanosarcina_acetivorans
NC_003552 Numero de Promotores x Posicion
NotCoding
Coding
Convergent
No.Promotores
6.97125
6.47125
5.97125
5.47125
4.97125
4.47125
3.97125
3.47125
2.97125
2.47125
-600550500450400350300250200150100-50 0 50100150200250300350400450500
sizeGeno: 5,751,492 bps %Cod: 0.74 %NoCod: 0.22 %Conv: 0.04 (297,394.48)
C_AT >= C_CG:0.55,0.46 NC_AT >= NC_CG:0.64,0.36

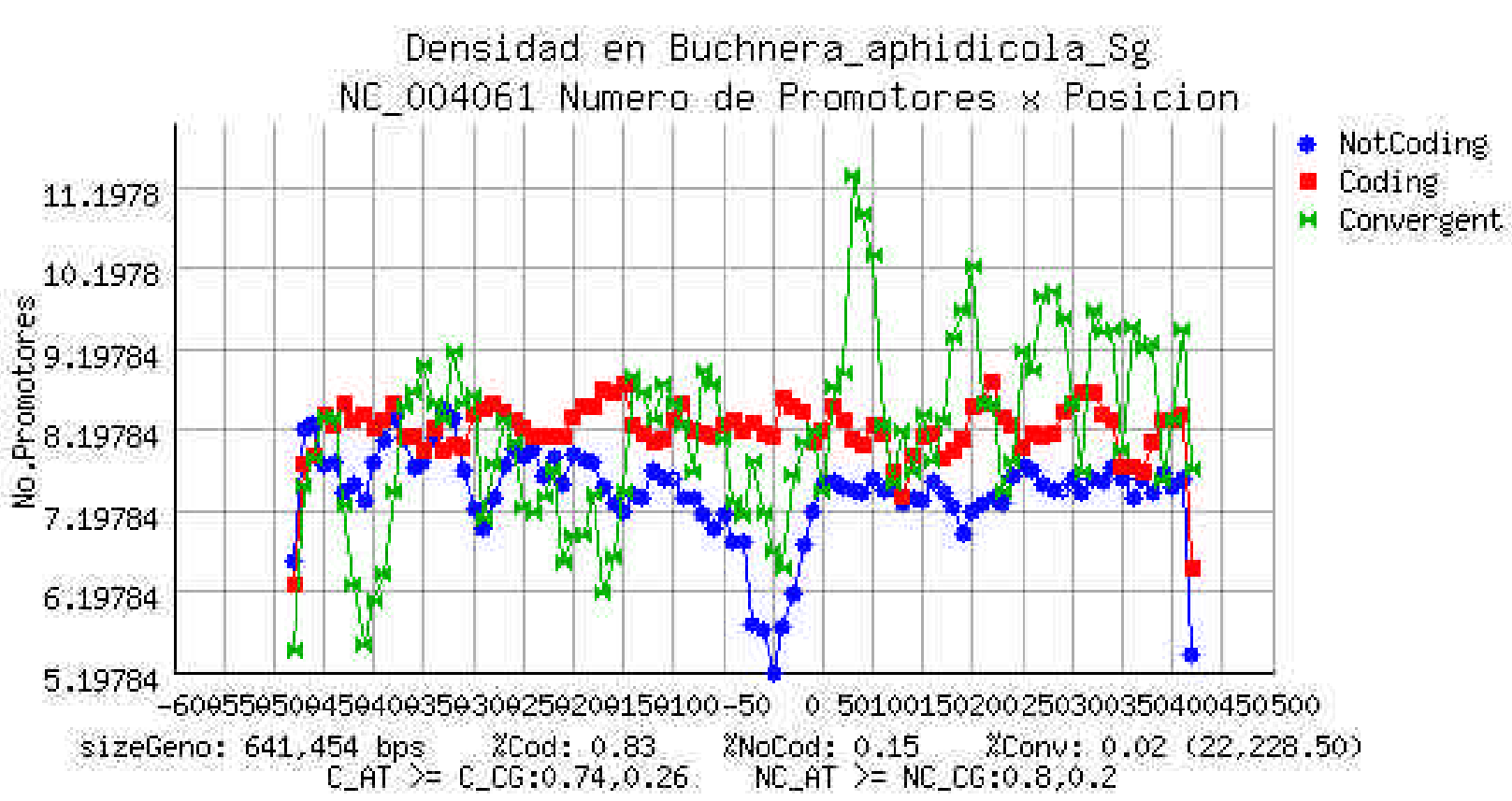
Densidad en Buchnera_aphidicola_Sg
NC_004061 Numero de Promotores x Posicion
NotCoding
Coding
Convergent
No.Promotores
11.1978
10.1978
9.19784
8.19784
7.19784
6.19784
5.19784
-600550500450400350300250200150100-50 0 50100150200250300350400450500
sizeGeno: 641,454 bps %Cod: 0.83 %NoCod: 0.15 %Conv: 0.02 (22,228.50)
C_AT >= C_CG:0.74,0.26 NC_AT >= NC_CG:0.8,0.2

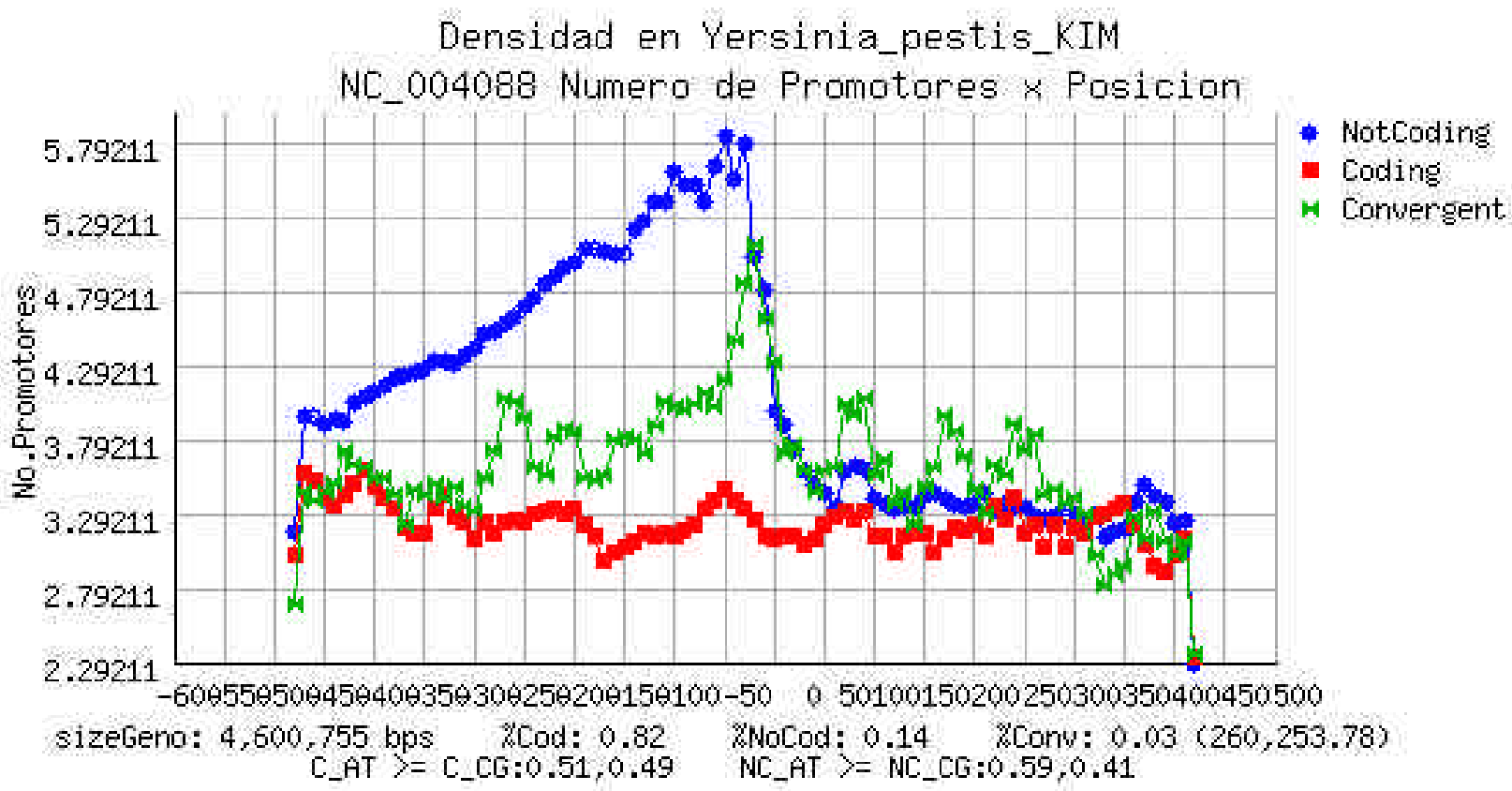
Densidad en Yersinia_pestis_KIM
NC_004088 Numero de Promotores x Posicion
No.Promotores
NotCoding
Coding
Convergent
sizeGeno: 4,600,755 bps %Cod: 0.82 %NoCod: 0.14 %Conv: 0.03 (260,253.78)
C_AT >= C_CG:0.51,0.49 NC_AT >= NC_CG:0.59,0.41

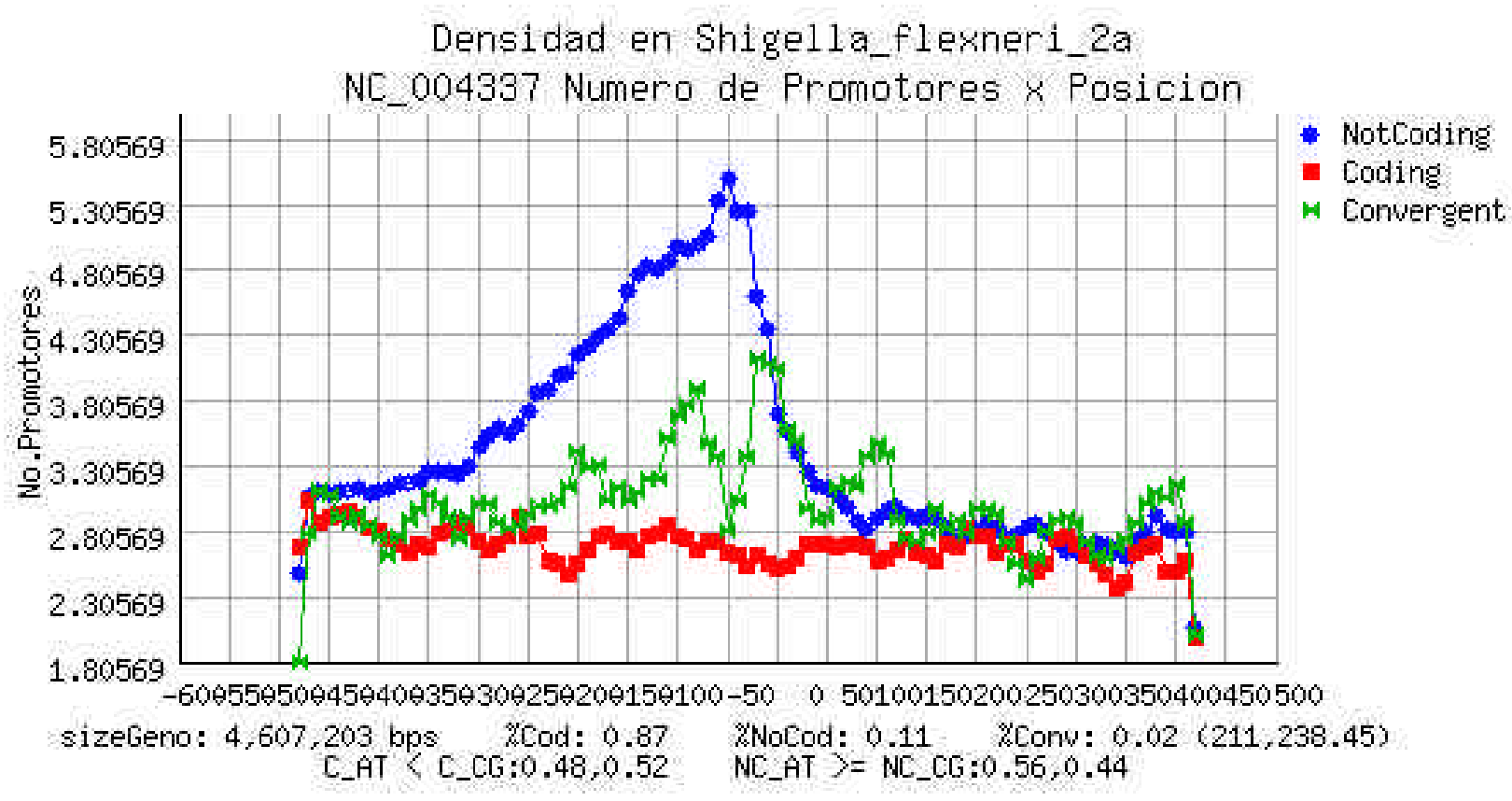
Densidad en Shigella_flexneri_2a
NC_004337 Numero de Promotores x Posicion
No.Promotores
NotCoding
Coding
Convergent
sizeGeno: 4,607,203 bps %Cod: 0.87 %NoCod: 0.11 %Conv: 0.02 (211,238.45)
C_AT < C_CG:0.48,0.52 NC_AT >= NC_CG:0.56,0.44

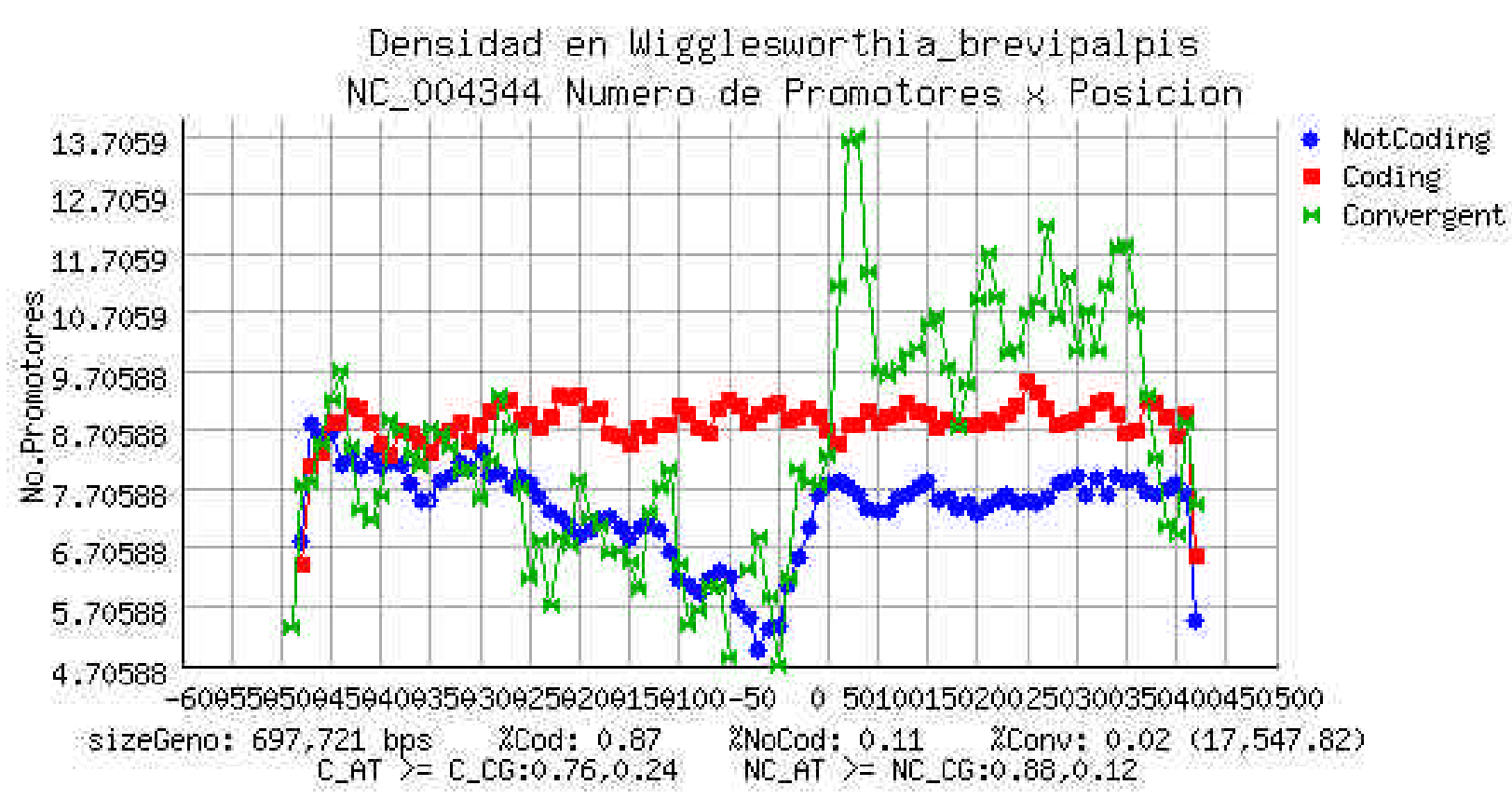
Densidad en Wigglesworthia_brevipalpis
NC_004344 Numero de Promotores x Posicion
No.Promotores
NotCoding
Coding
Convergent
sizeGeno: 697,721 bps %Cod: 0.87 %NoCod: 0.11 %Conv: 0.02 (17,547.82)
C_AT >= C_CG:0.76,0.24 NC_AT >= NC_CG:0.88,0.12

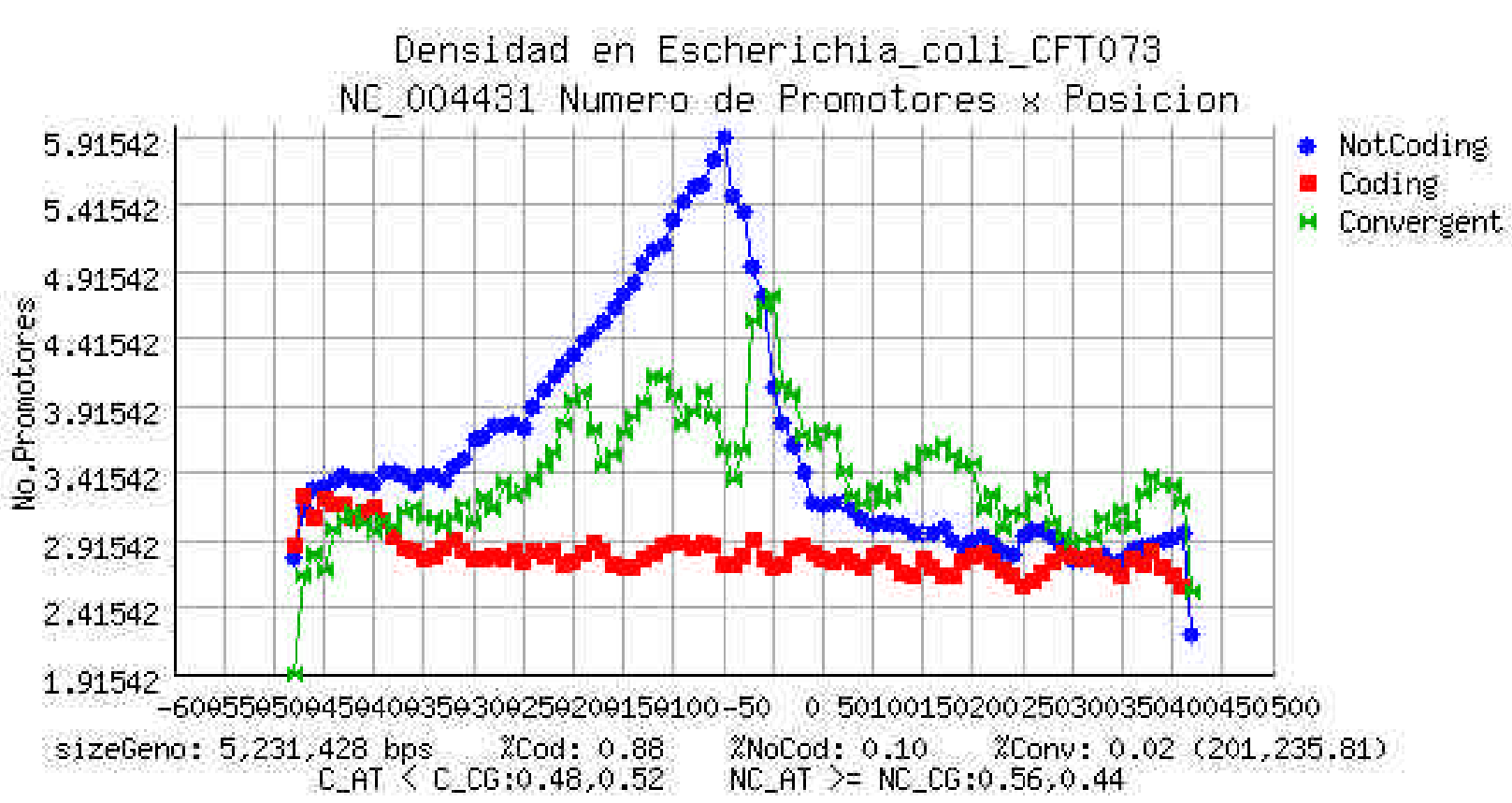
Densidad en Escherichia_coli_CFT073
NC_004431 Numero de Promotores x Posicion
No.Promotores
NotCoding
Coding
Convergent
sizeGeno: 5,231,428 bps %Cod: 0.88 %NoCod: 0.10 %Conv: 0.02 (201,235.81)
C_AT < C_CG:0.48,0.52 NC_AT >= NC_CG:0.56,0.44

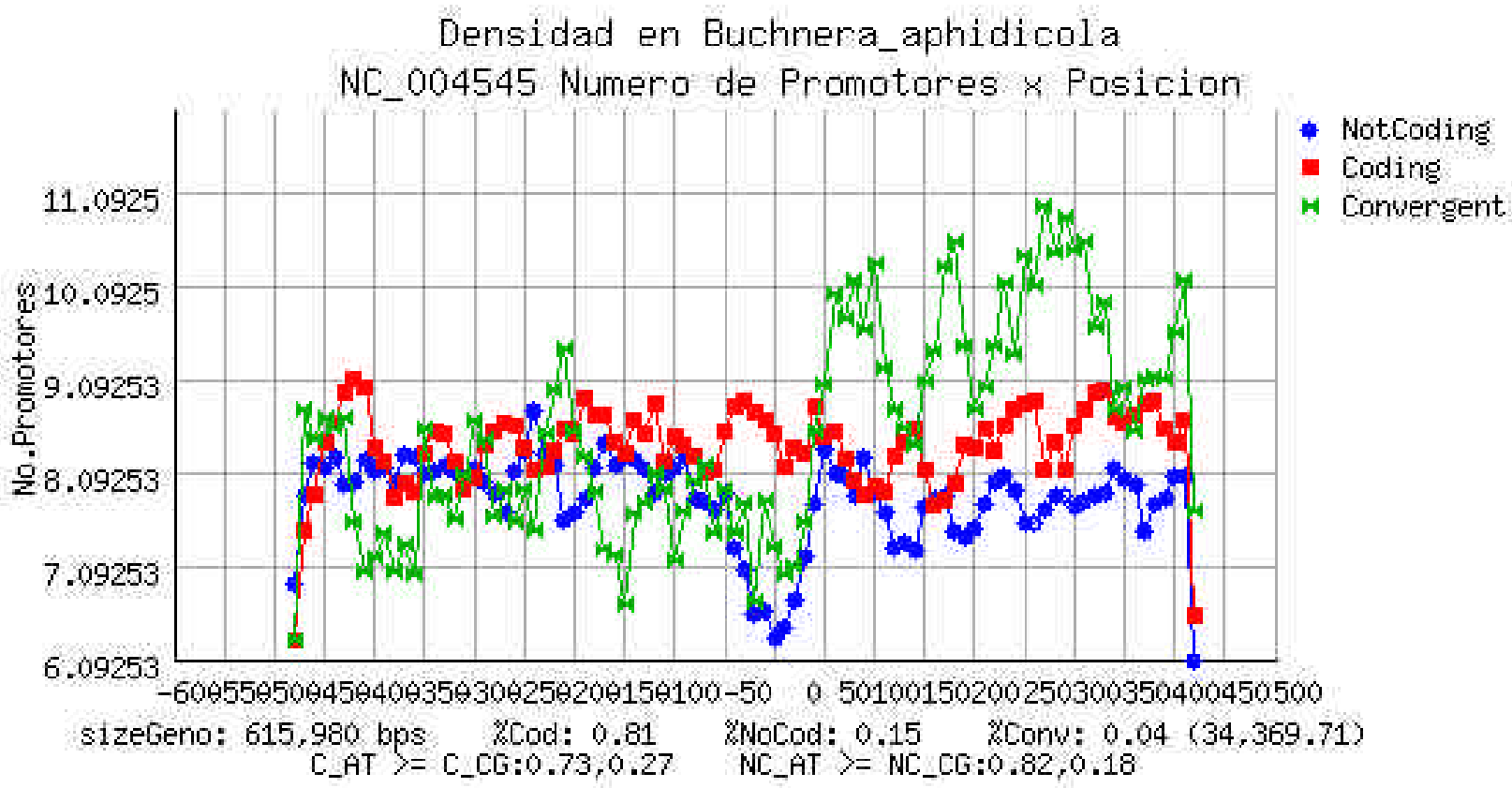
Densidad en Buchnera_aphidicola
NC_004545 Numero de Promotores x Posicion
NotCoding
Coding
Convergent
No.Promotores
sizeGeno: 615,980 bps %Cod: 0.81 %NoCod: 0.15 %Conv: 0.04 (34,369.71)
C_AT >= C_CG:0.73,0.27 NC_AT >= NC_CG:0.82,0.18

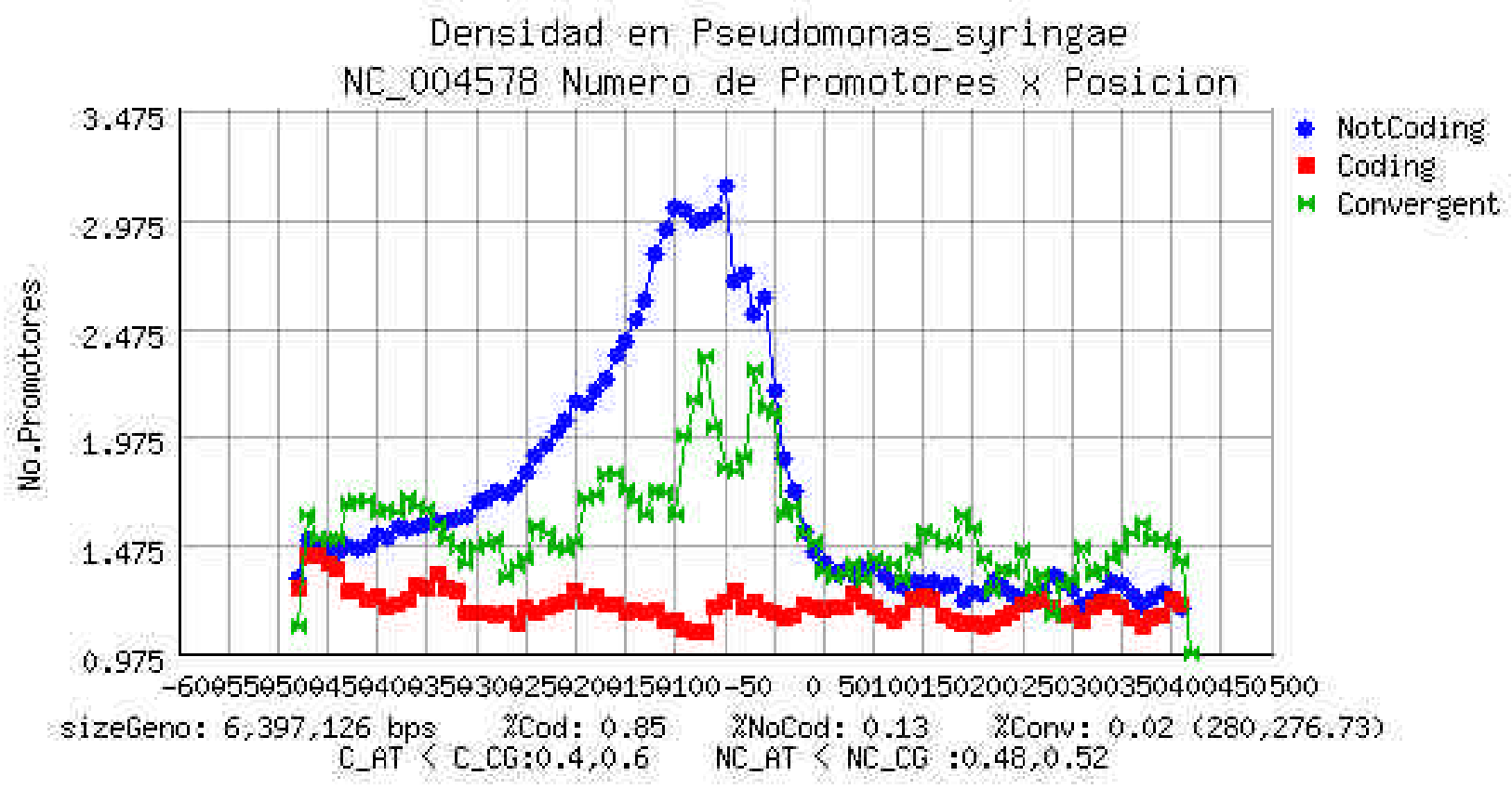
Densidad en Pseudomonas_syringae
NC_004578 Numero de Promotores x Posicion
NotCoding
Coding
Convergent
No.Promotores
sizeGeno: 6,397,126 bps %Cod: 0.85 %NoCod: 0.13 %Conv: 0.02 (280,276.73)
C_AT < C_CG:0.4,0.6 NC_AT < NC_CG :0.48,0.52

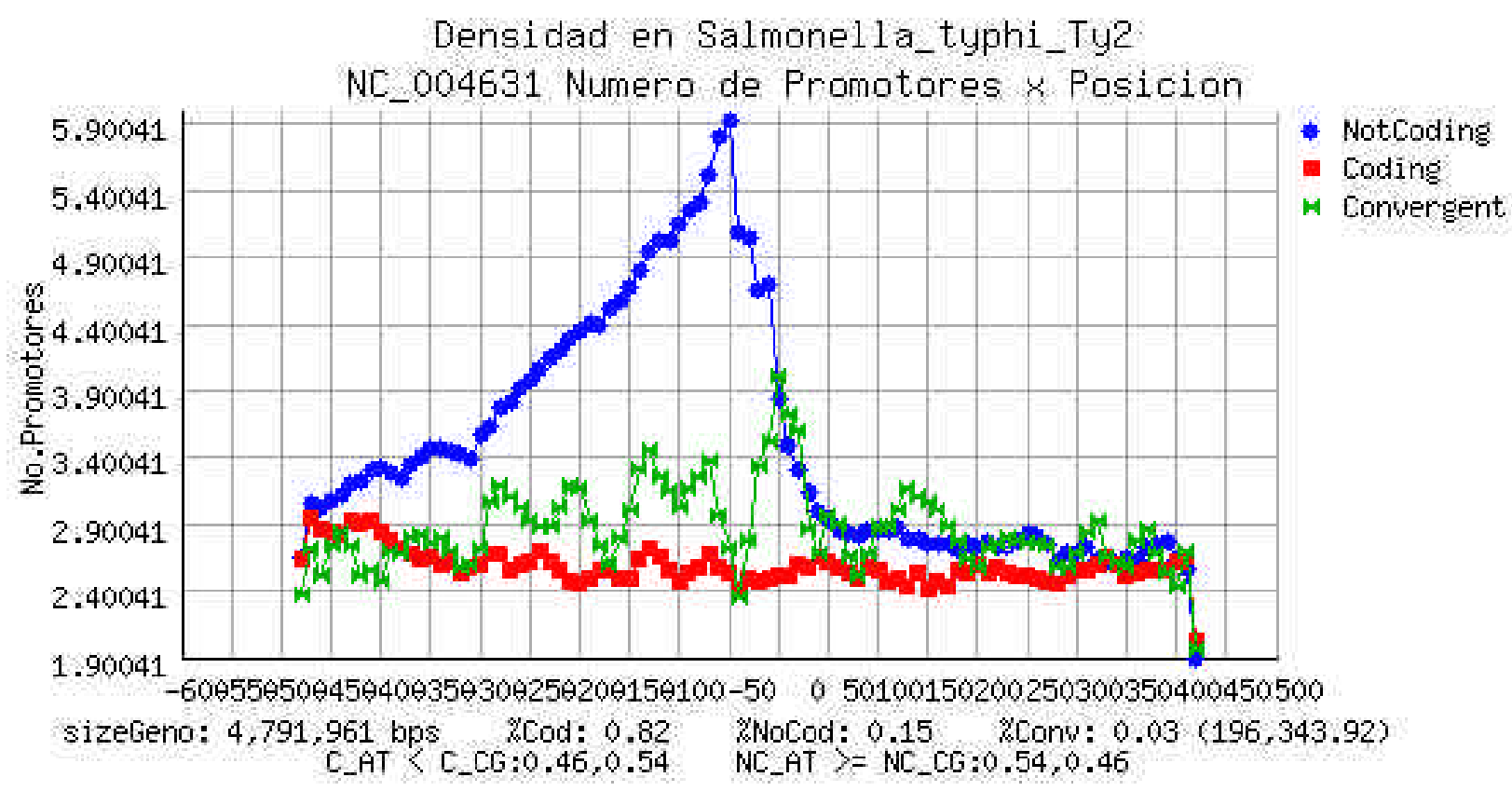
Densidad en Salmonella_typhi_Ty2
NC_004631 Numero de Promotores x Posicion
NotCoding
Coding
Convergent
No.Promotores
sizeGeno: 4,791,961 bps %Cod: 0.82 %NoCod: 0.15 %Conv: 0.03 (196,343.92)
C_AT < C_CG:0.46,0.54 NC_AT >= NC_CG:0.54,0.46

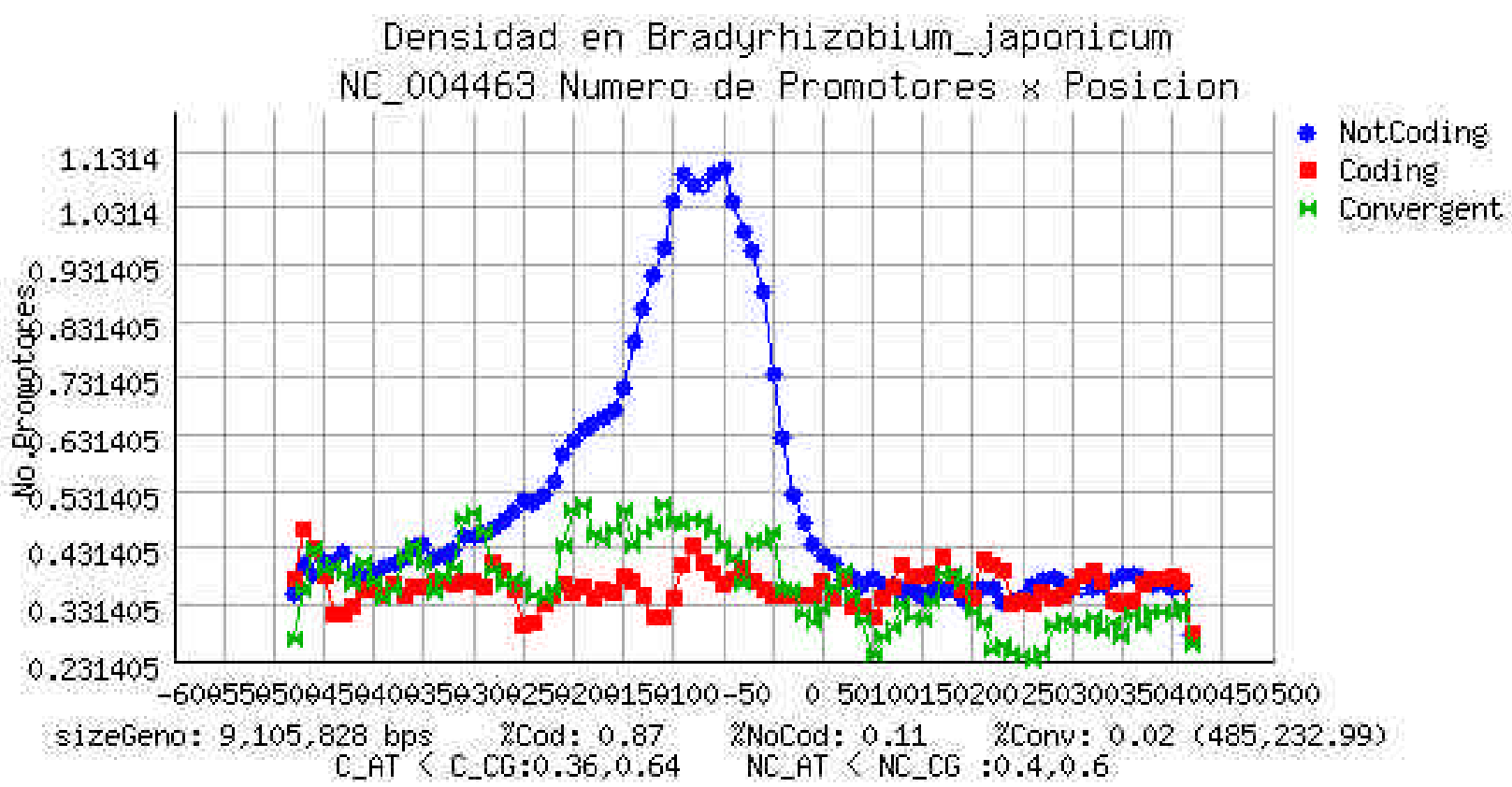
Densidad en Bradyrhizobium_japonicum
NC_004463 Numero de Promotores x Posicion
NotCoding
Coding
Convergent
No.Promotores
sizeGeno: 9,105,828 bps %Cod: 0.87 %NoCod: 0.11 %Conv: 0.02 (485,232.99)
C_AT < C_CG:0.36,0.64 NC_AT < NC_CG :0.4,0.6
