# Supplementary material for: Selection for Unequal Densities of σ70 Promoter-Like Signals in Different Regions of Large Bacterial Genomes
Source: PLoS Genet. 2006 Nov 10;2(11):e185. doi: 10.1371/journal.pgen.0020185 (PMC1635534; doi:10.1371/journal.pgen.0020185)
Supplement: Supplementary file 2 [file pgen.0020185.sg002.pdf]

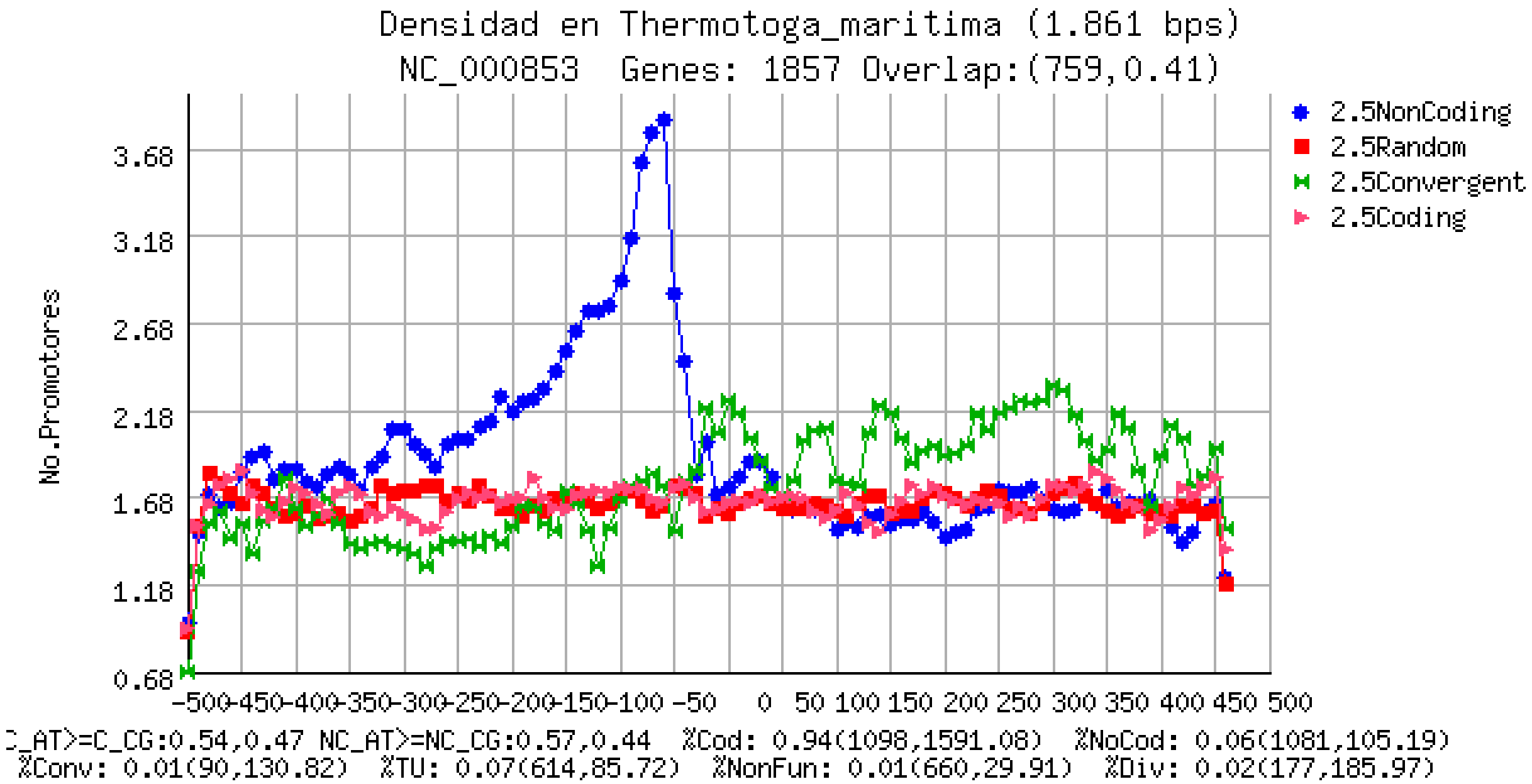
Densidad en Thermotoga_maritima (1.861 bps)
NC_000853 Genes: 1857 Overlap:(759,0.41)
2.5NonCoding
2.5Random
2.5Convergent
2.5Coding
No.Promotores
3.68
3.18
2.68
2.18
1.68
1.18
0.68
-500-450-400-350-300-250-200-150-100 -50 0 50 100 150 200 250 300 350 400 450 500
C_AT>=C_CG:0.54,0.47 NC_AT>=NC_CG:0.57,0.44 %Cod: 0.94(1098,1591.08) %NoCod: 0.06(1081,105.19)
%Conv: 0.01(90,130.82) %TU: 0.07(614,85.72) %NonFun: 0.01(660,29.91) %Div: 0.02(177,185.97)

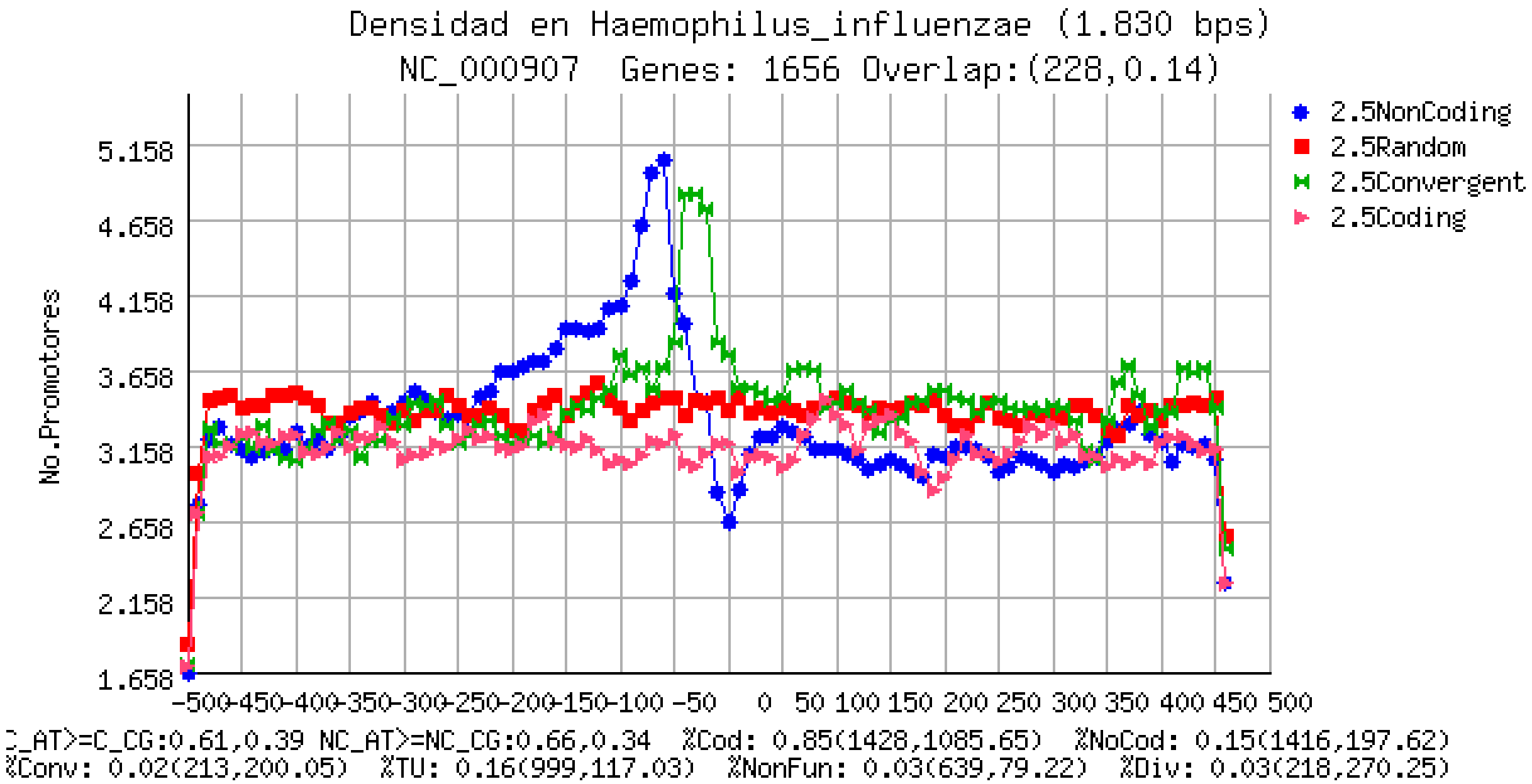
Densidad en Haemophilus_influenzae (1.830 bps)
NC_000907 Genes: 1656 Overlap:(228,0.14)
No.Promotores
5.158
4.658
4.158
3.658
3.158
2.658
2.158
1.658
-500 -450 -400 -350 -300 -250 -200 -150 -100 -50 0 50 100 150 200 250 300 350 400 450 500
2.5NonCoding
2.5Random
2.5Convergent
2.5Coding
C_AT>=C_CG:0.61,0.39 NC_AT>=NC_CG:0.66,0.34 %Cod: 0.85(1428,1085.65) %NoCod: 0.15(1416,197.62)
%Conv: 0.02(213,200.05) %TU: 0.16(999,117.03) %NonFun: 0.03(639,79.22) %Div: 0.03(218,270.25)

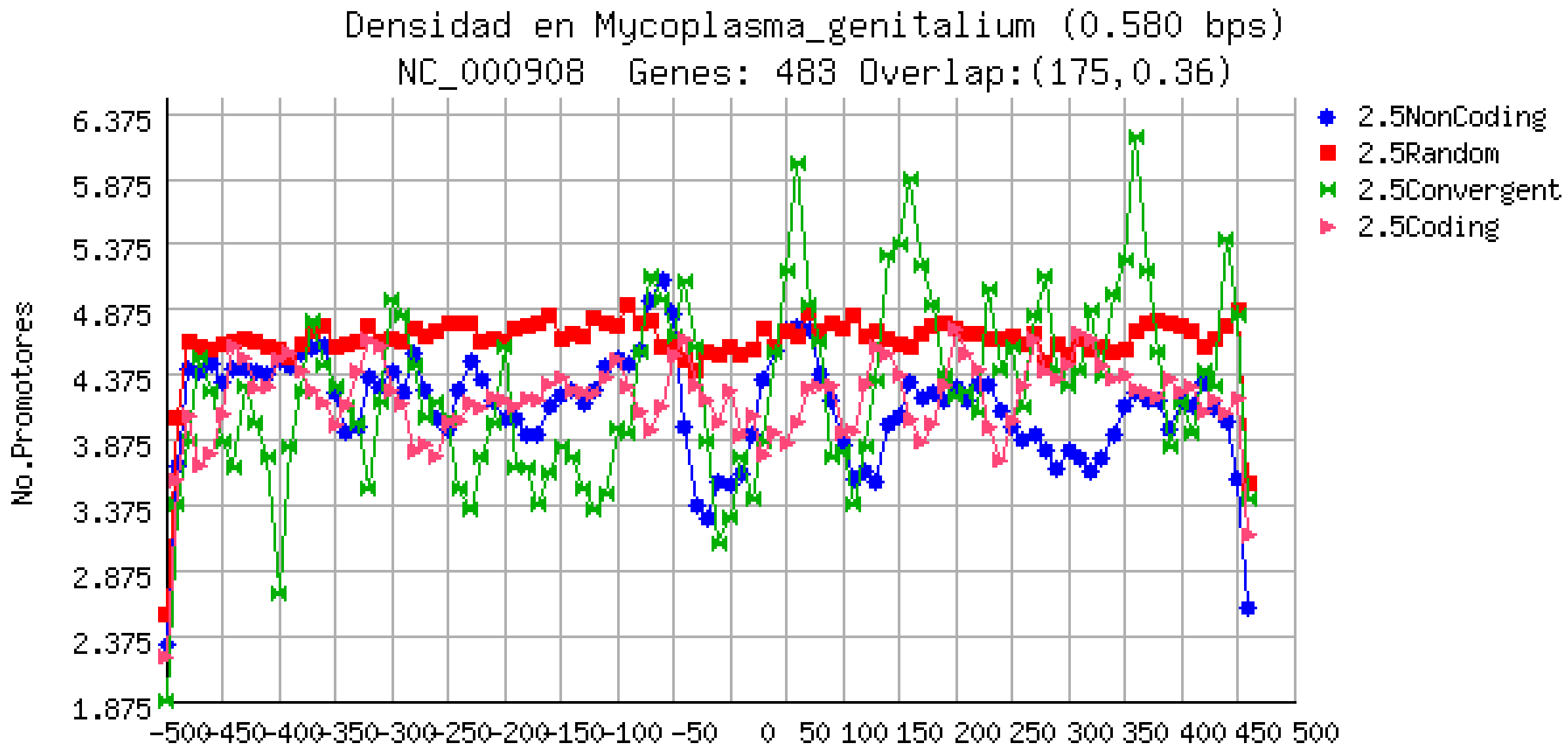

C_AT>=C_CG:0.69,0.32 NC_AT>=NC_CG:0.67,0.33 %Cod: 0.91(308,1707.22) %NoCod: 0.09(300,180.83)
%Conv: 0.01(12,408.17) %TU: 0.09(167,116.43) %NonFun: 0.01(174,39.83) %Div: 0.01(35,179.11)

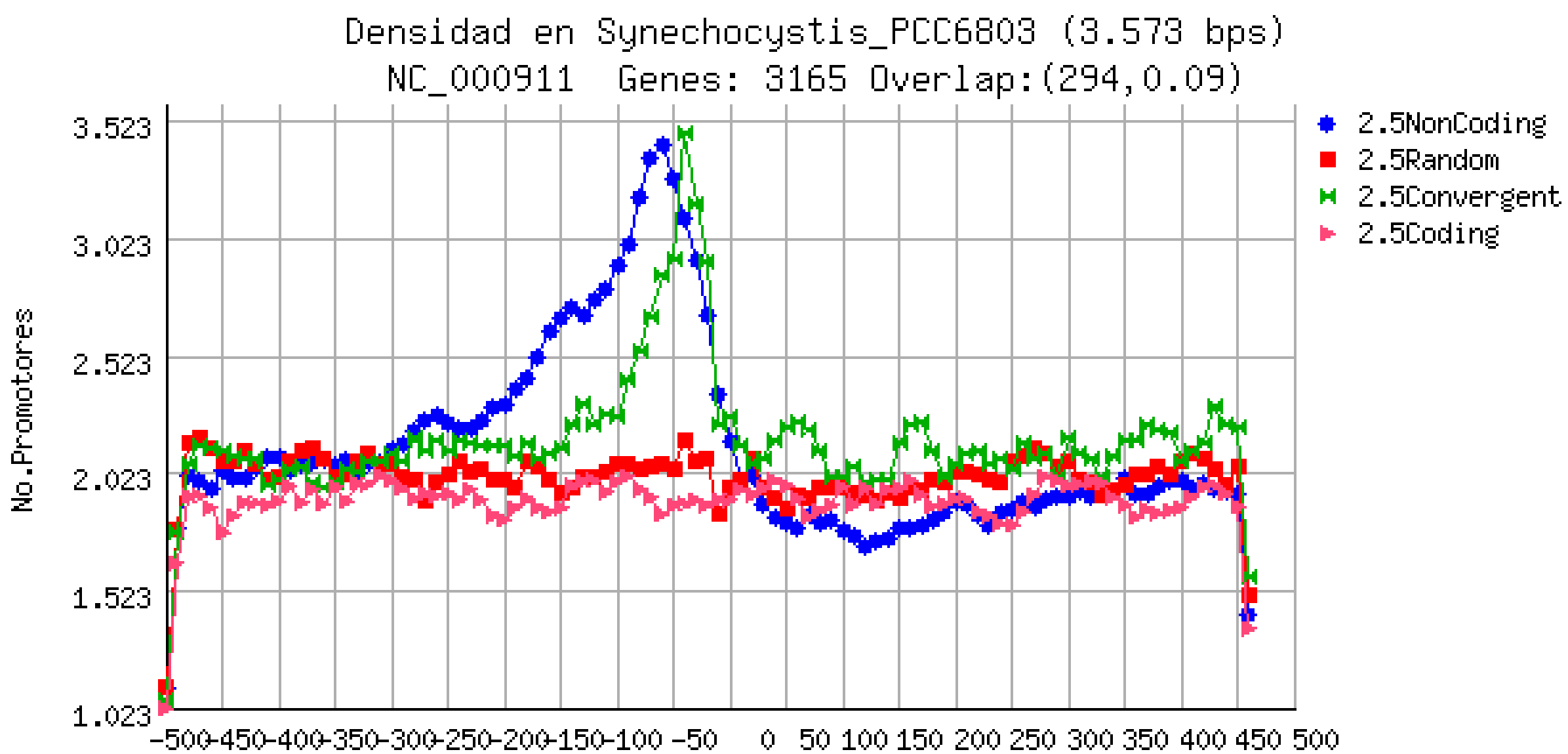

C_AT>=C_CG:0.52,0.48 NC_AT>=NC_CG:0.58,0.42 %Cod: 0.87(2871,1080.08) %NoCod: 0.13(2862,165.12)
%Conv: 0.02(458,128.41) %TU: 0.15(2486,80.63) %NonFun: 0.02(922,77.75) %Div: 0.04(527,267.01)

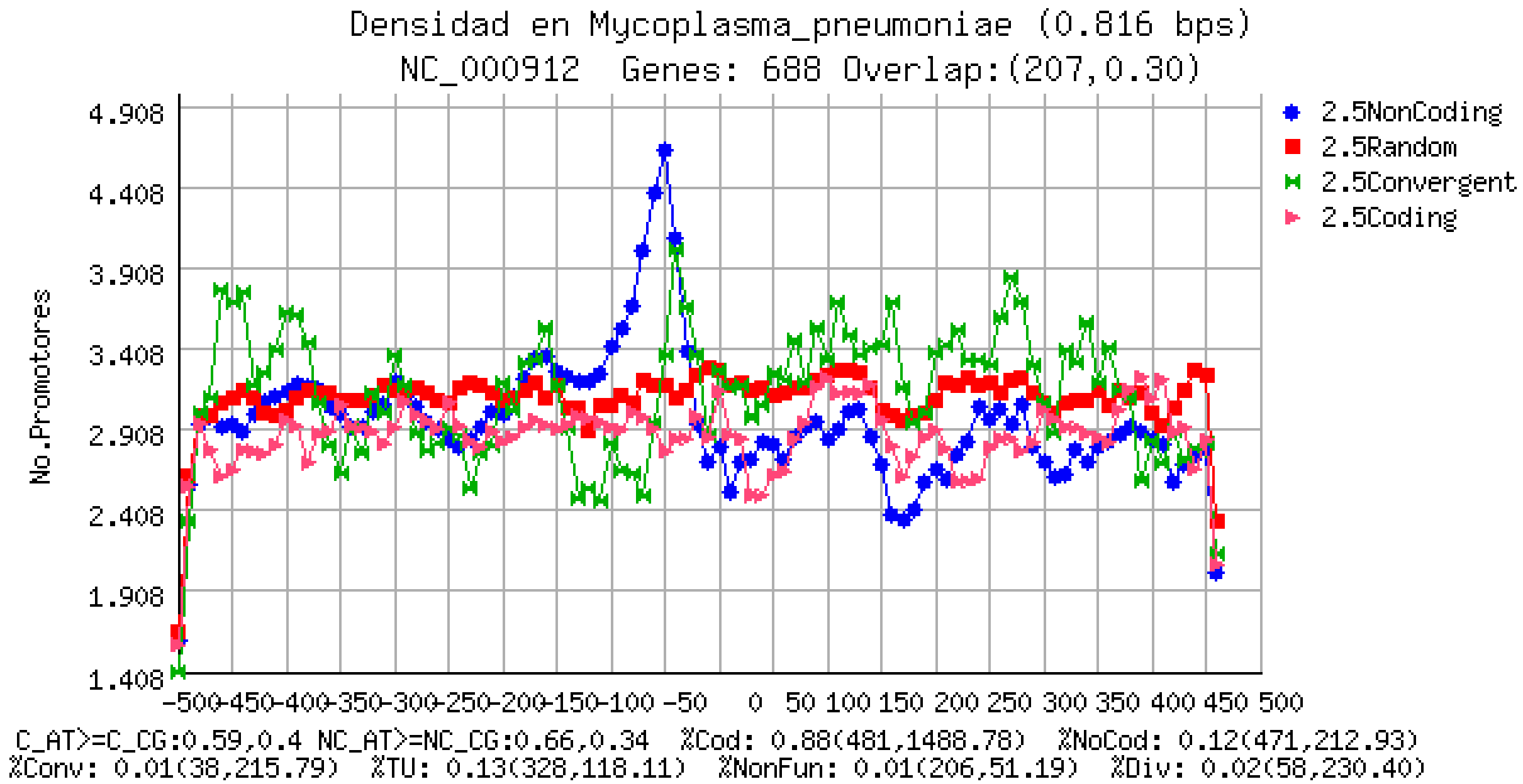
Densidad en Mycoplasma_pneumoniae (0.816 bps)
NC_000912 Genes: 688 Overlap:(207,0.30)
No.Promotores
4.908
4.408
3.908
3.408
2.908
2.408
1.908
1.408
-500 -450 -400 -350 -300 -250 -200 -150 -100 -50 0 50 100 150 200 250 300 350 400 450 500
2.5NonCoding
2.5Random
2.5Convergent
2.5Coding
C_AT>=C_CG:0.59,0.4 NC_AT>=NC_CG:0.66,0.34 %Cod: 0.88(481,1488.78) %NoCod: 0.12(471,212.93)
%Conv: 0.01(38,215.79) %TU: 0.13(328,118.11) %NonFun: 0.01(206,51.19) %Div: 0.02(58,230.40)

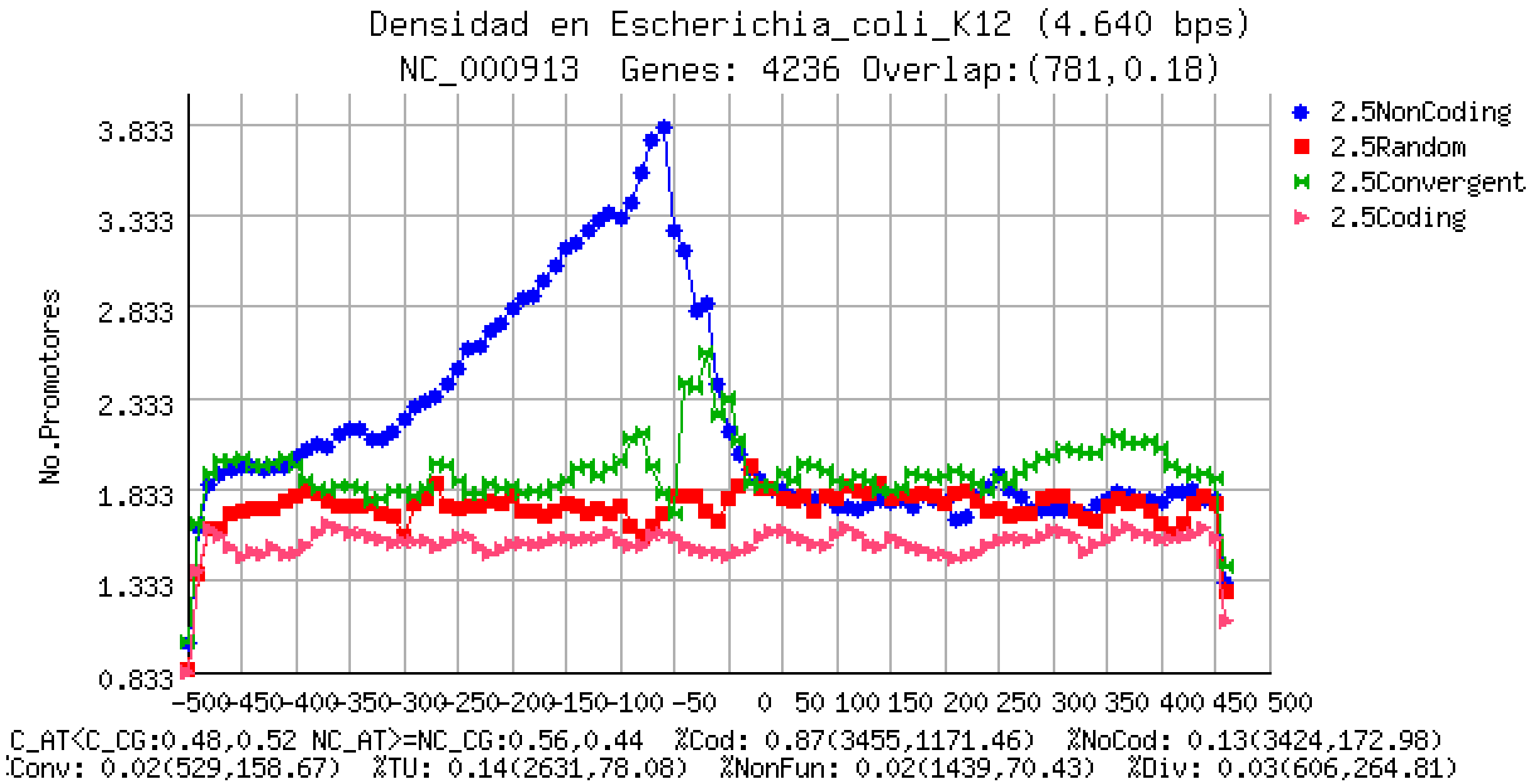
Densidad en Escherichia_coli_K12 (4.640 bps)
NC_000913 Genes: 4236 Overlap:(781,0.18)
No.Promotores
3.833
3.333
2.833
2.333
1.833
1.333
0.833
-500 -450 -400 -350 -300 -250 -200 -150 -100 -50 0 50 100 150 200 250 300 350 400 450 500
2.5NonCoding
2.5Random
2.5Convergent
2.5Coding
C_AT<C_CG:0.48,0.52 NC_AT>=NC_CG:0.56,0.44 %Cod: 0.87(3455,1171.46) %NoCod: 0.13(3424,172.98)
Conv: 0.02(529,158.67) %TU: 0.14(2631,78.08) %NonFun: 0.02(1439,70.43) %Div: 0.03(606,264.81)

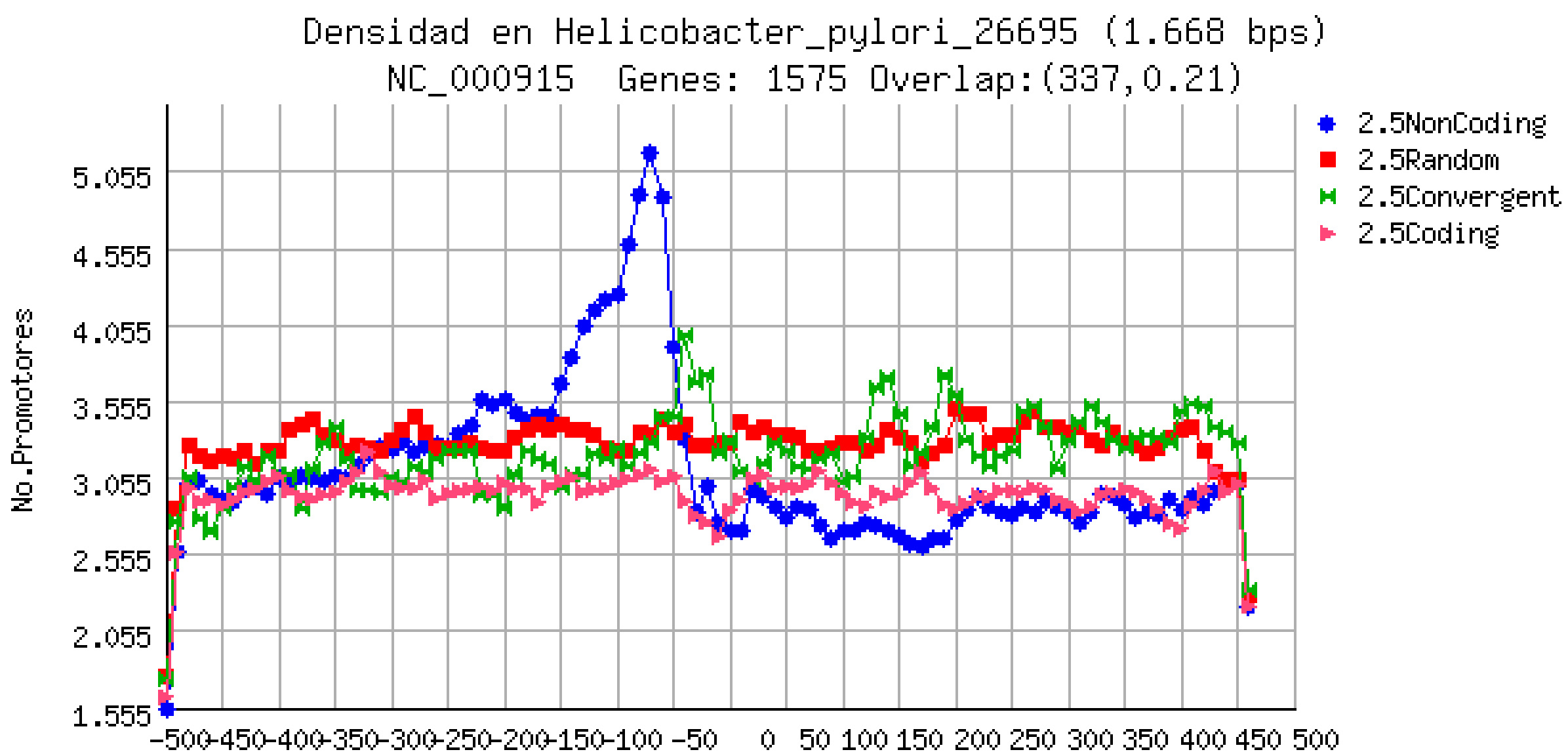

C_AT>=C_CG:0.61,0.4 NC_AT>=NC_CG:0.68,0.32 %Cod: 0.90(1238,1215.04) %NoCod: 0.10(1208,135.47)
%Conv: 0.02(158,196.65) %TU: 0.10(753,94.96) %NonFun: 0.02(633,60.79) %Div: 0.03(170,248.23)

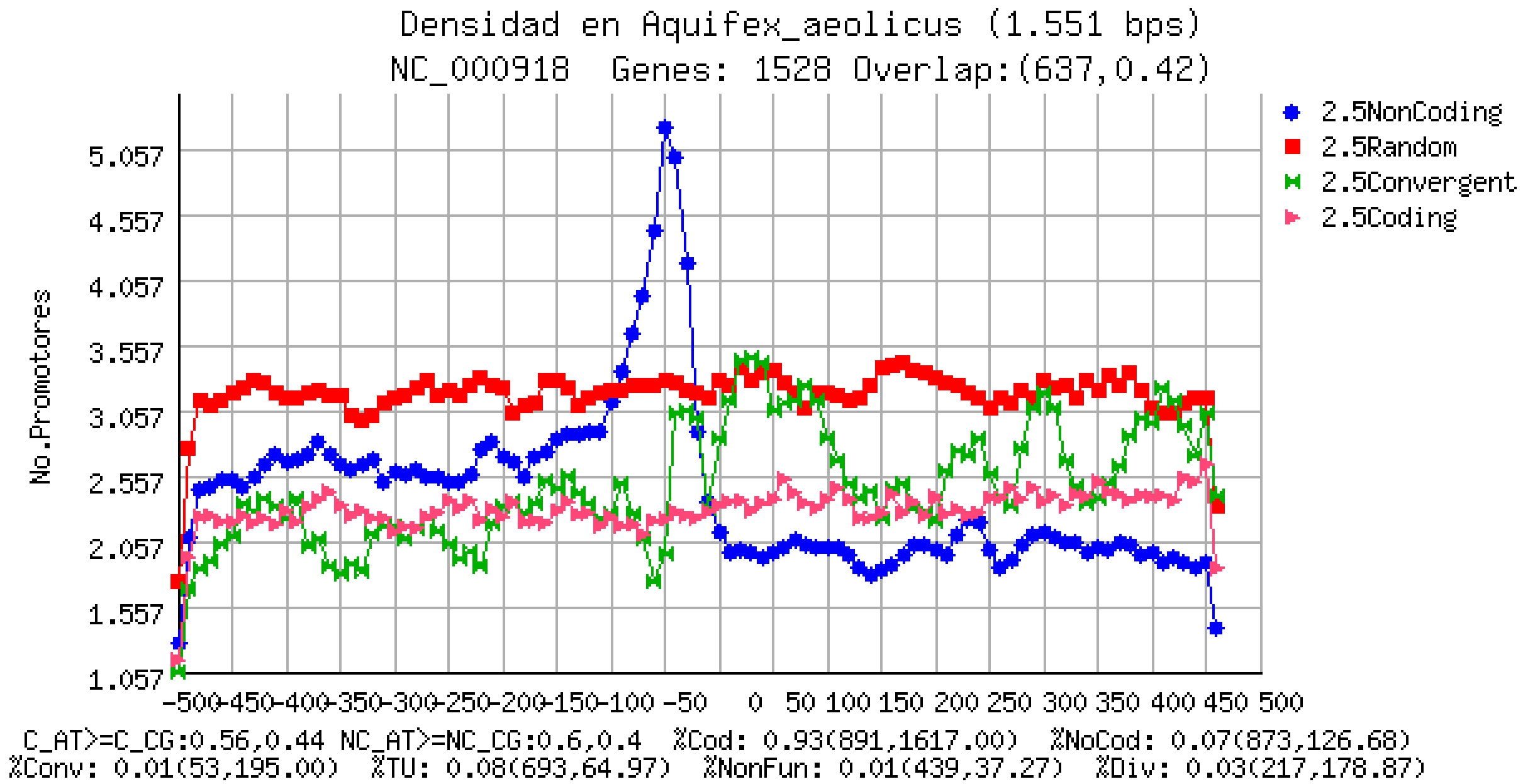
Densidad en Aquifex_aeolicus (1.551 bps)
NC_000918 Genes: 1528 Overlap:(637,0.42)
No.Promotores
5.057
4.557
4.057
3.557
3.057
2.557
2.057
1.557
1.057
-500-450-400-350-300-250-200-150-100 -50 0 50 100 150 200 250 300 350 400 450 500
2.5NonCoding
2.5Random
2.5Convergent
2.5Coding
C_AT>=C_CG:0.56,0.44 NC_AT>=NC_CG:0.6,0.4 %Cod: 0.93(891,1617.00) %NoCod: 0.07(873,126.68)
%Conv: 0.01(53,195.00) %TU: 0.08(693,64.97) %NonFun: 0.01(439,37.27) %Div: 0.03(217,178.87)

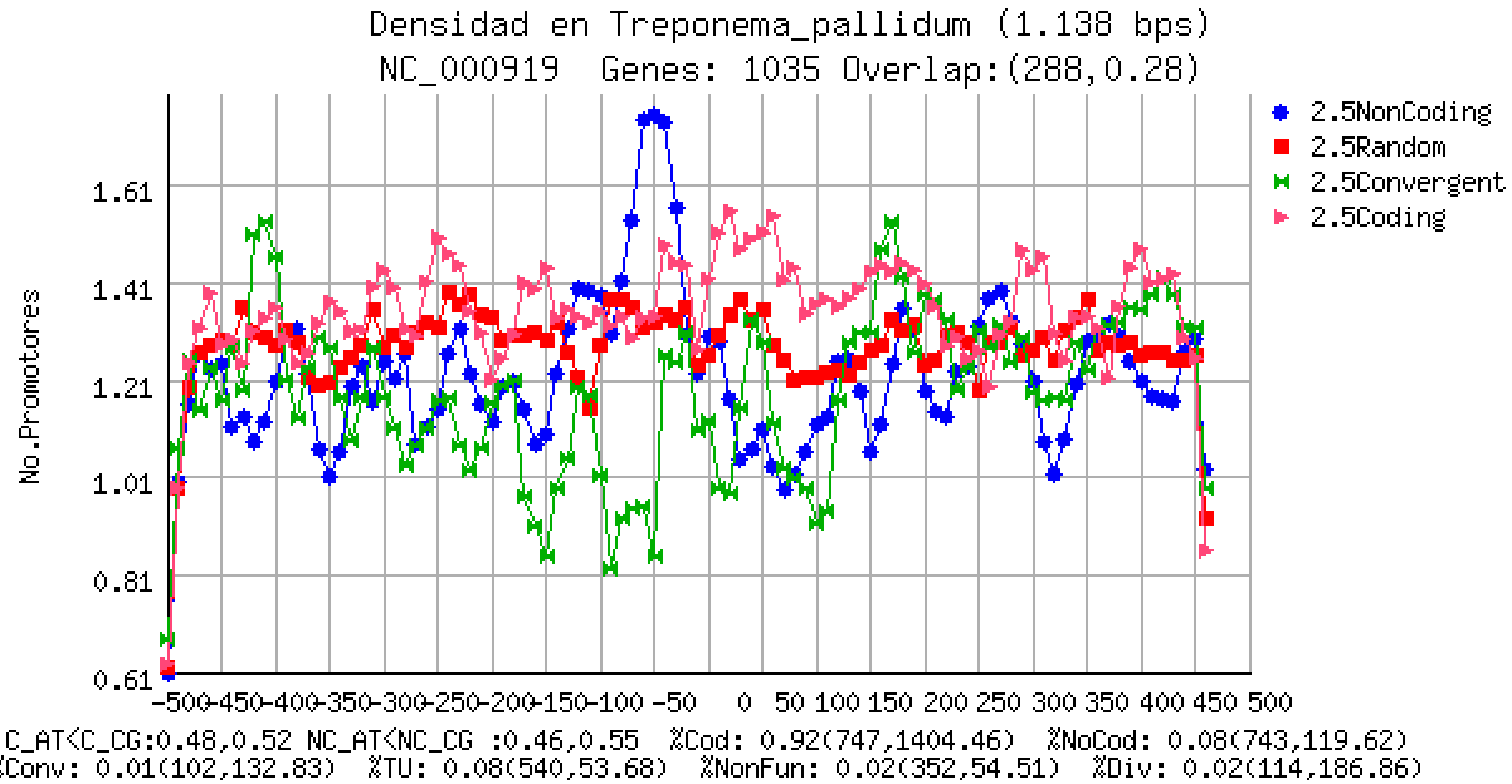
Densidad en Treponema_pallidum (1.138 bps)
NC_000919 Genes: 1035 Overlap:(288,0.28)
2.5NonCoding
2.5Random
2.5Convergent
2.5Coding
No.Promotores
1.61
1.41
1.21
1.01
0.81
0.61
-500-450-400-350-300-250-200-150-100 -50 0 50 100 150 200 250 300 350 400 450 500
C_AT<C_CG:0.48,0.52 NC_AT<NC_CG :0.46,0.55 %Cod: 0.92(747,1404.46) %NoCod: 0.08(743,119.62)
%Conv: 0.01(102,132.83) %TU: 0.08(540,53.68) %NonFun: 0.02(352,54.51) %Div: 0.02(114,186.86)

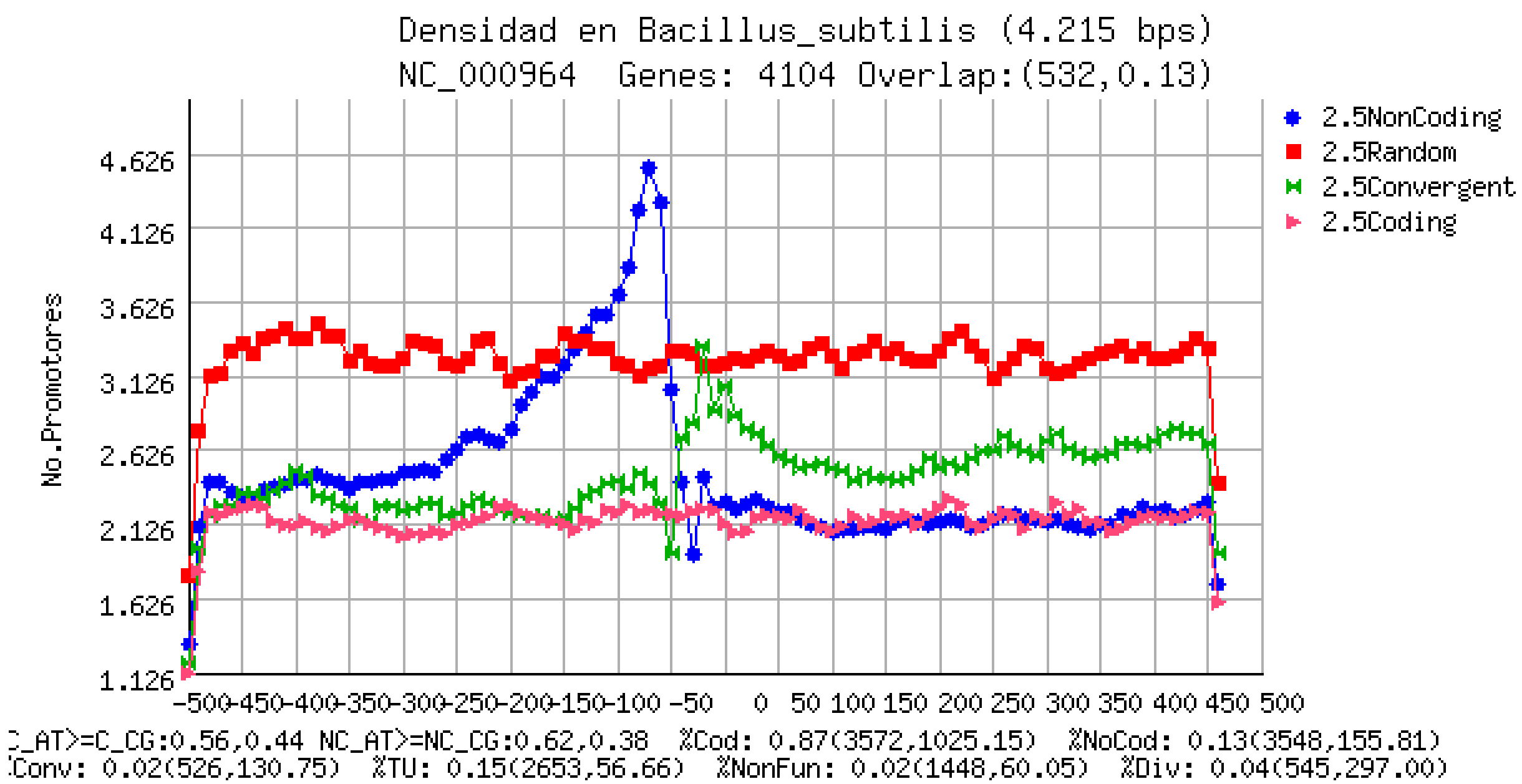
Densidad en Bacillus_subtilis (4.215 bps)
NC_000964 Genes: 4104 Overlap:(532,0.13)
2.5NonCoding
2.5Random
2.5Convergent
2.5Coding
No.Promotores
4.626
4.126
3.626
3.126
2.626
2.126
1.626
1.126
-500-450-400-350-300-250-200-150-100 -50 0 50 100 150 200 250 300 350 400 450 500
C_AT>=C_CG:0.56,0.44 NC_AT>=NC_CG:0.62,0.38 %Cod: 0.87(3572,1025.15) %NoCod: 0.13(3548,155.81)
Conv: 0.02(526,130.75) %TU: 0.15(2653,56.66) %NonFun: 0.02(1448,60.05) %Div: 0.04(545,297.00)

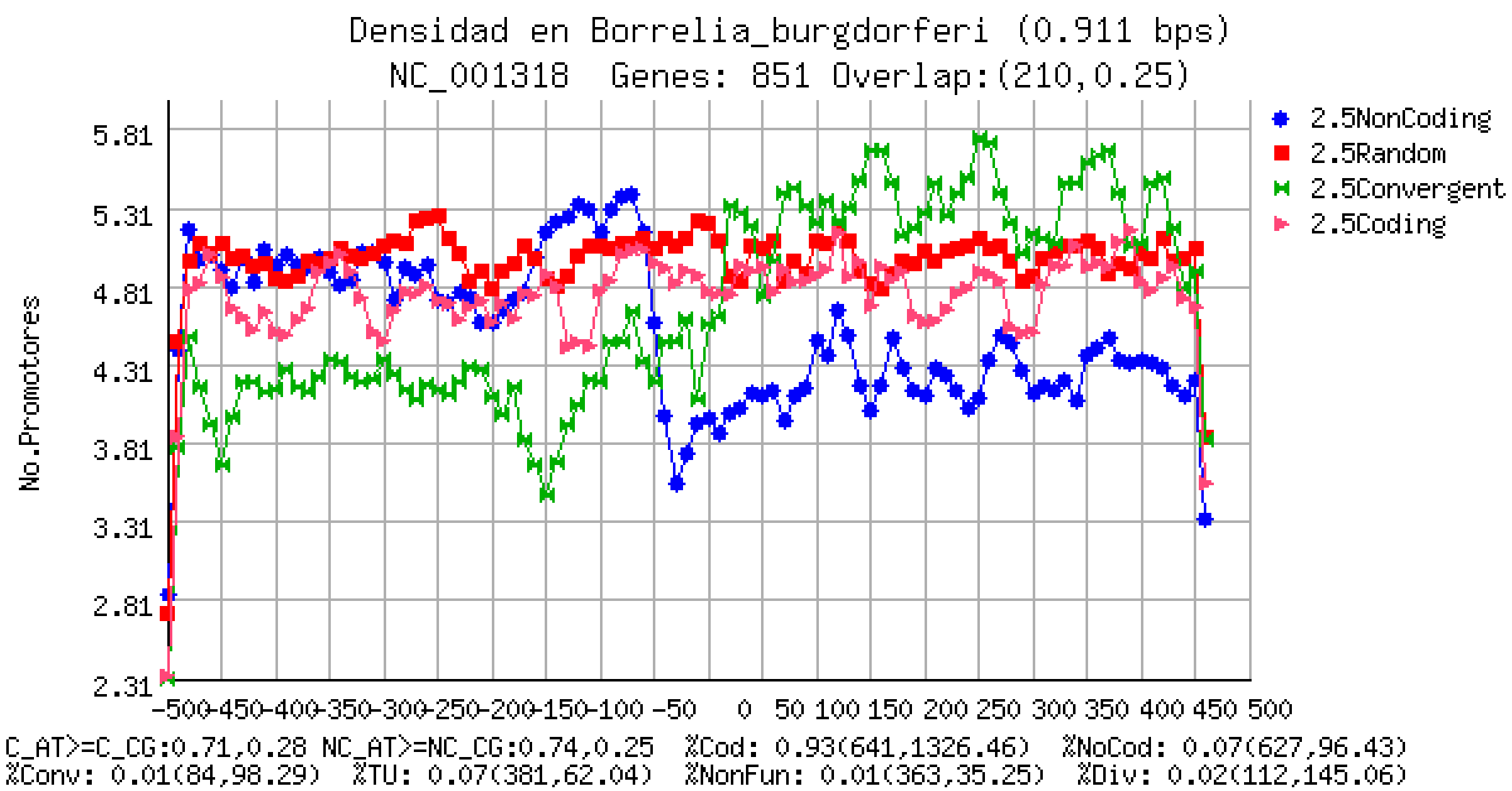
Densidad en Borrelia_burgdorferi (0.911 bps)
NC_001318 Genes: 851 Overlap:(210,0.25)
No.Promotores
5.81
5.31
4.81
4.31
3.81
3.31
2.81
2.31
-500-450-400-350-300-250-200-150-100 -50 0 50 100 150 200 250 300 350 400 450 500
2.5NonCoding
2.5Random
2.5Convergent
2.5Coding
C_AT>=C_CG:0.71,0.28 NC_AT>=NC_CG:0.74,0.25 %Cod: 0.93(641,1326.46) %NoCod: 0.07(627,96.43)
%Conv: 0.01(84,98.29) %TU: 0.07(381,62.04) %NonFun: 0.01(363,35.25) %Div: 0.02(112,145.06)

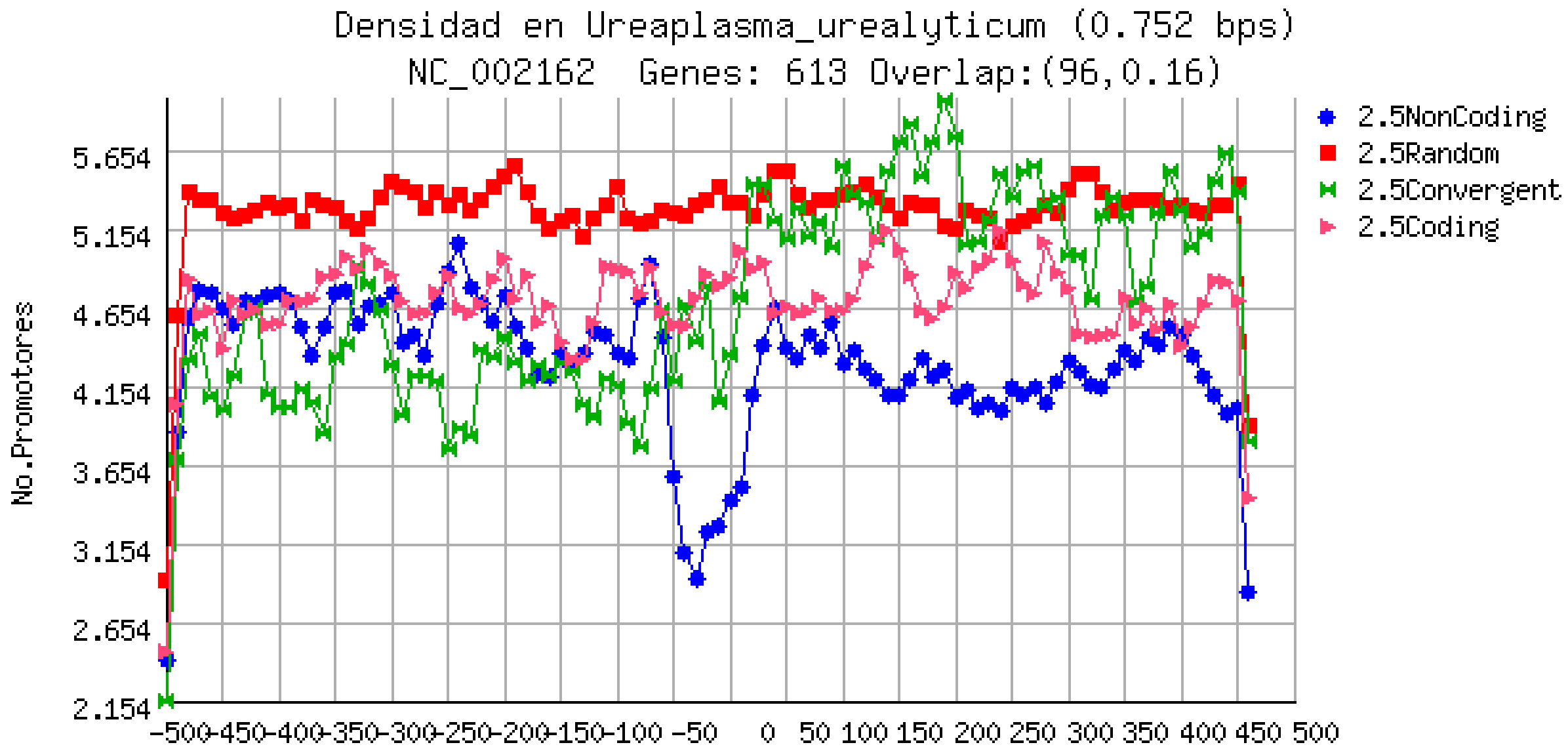

C_AT>=C_CG:0.74,0.26 NC_AT>=NC_CG:0.77,0.23 %Cod: 0.91(517,1324.60) %NoCod: 0.09(509,131.44)
%Conv: 0.02(52,230.98) %TU: 0.08(290,103.11) %NonFun: 0.02(276,56.99) %Div: 0.02(53,235.51)

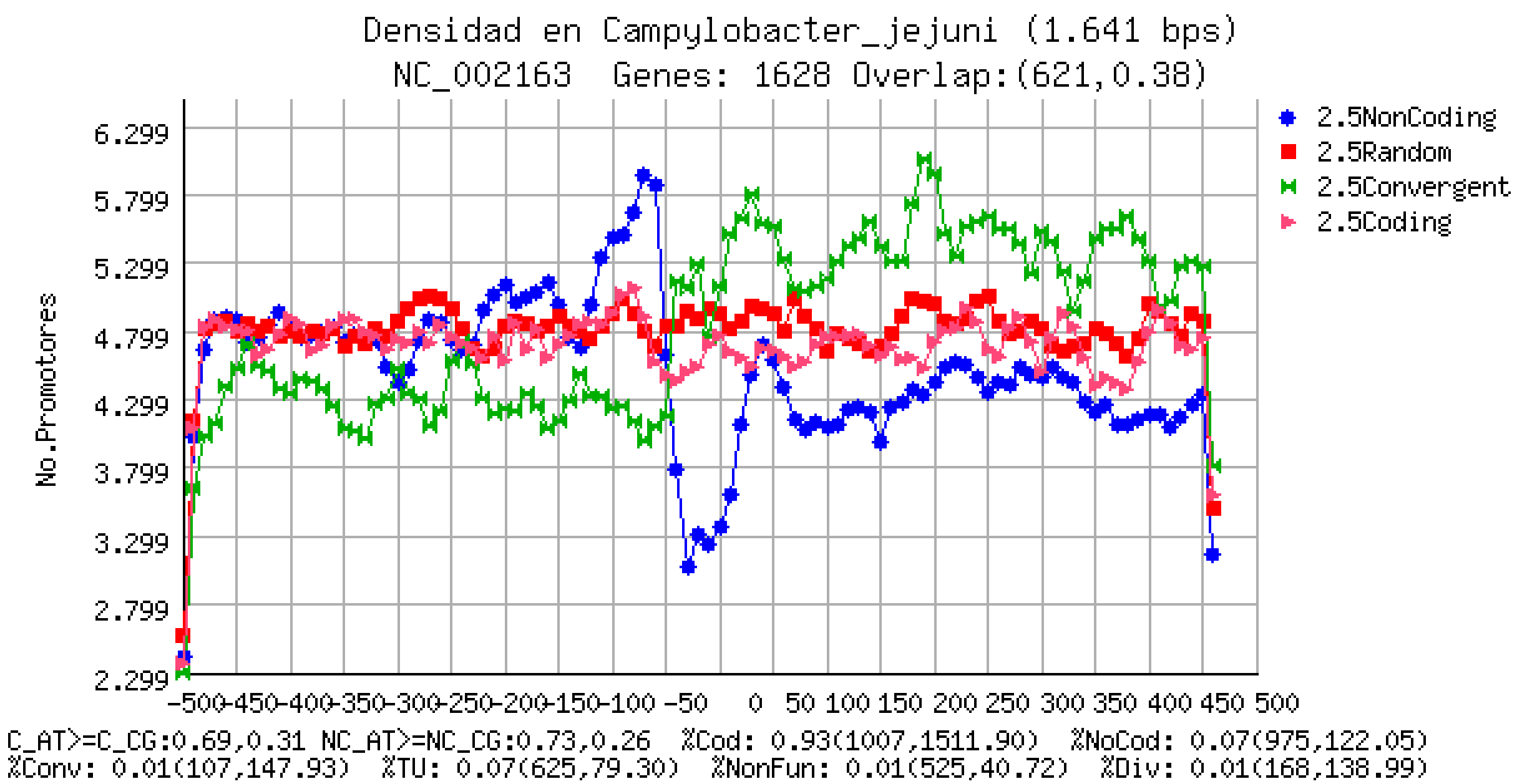
Densidad en Campylobacter_jejuni (1.641 bps)
NC_002163 Genes: 1628 Overlap:(621,0.38)
No.Promotores
6.299
5.799
5.299
4.799
4.299
3.799
3.299
2.799
2.299
-500-450-400-350-300-250-200-150-100 -50 0 50 100 150 200 250 300 350 400 450 500
2.5NonCoding
2.5Random
2.5Convergent
2.5Coding
C_AT>=C_CG:0.69,0.31 NC_AT>=NC_CG:0.73,0.26 %Cod: 0.93(1007,1511.90) %NoCod: 0.07(975,122.05)
%Conv: 0.01(107,147.93) %TU: 0.07(625,79.30) %NonFun: 0.01(525,40.72) %Div: 0.01(168,138.99)

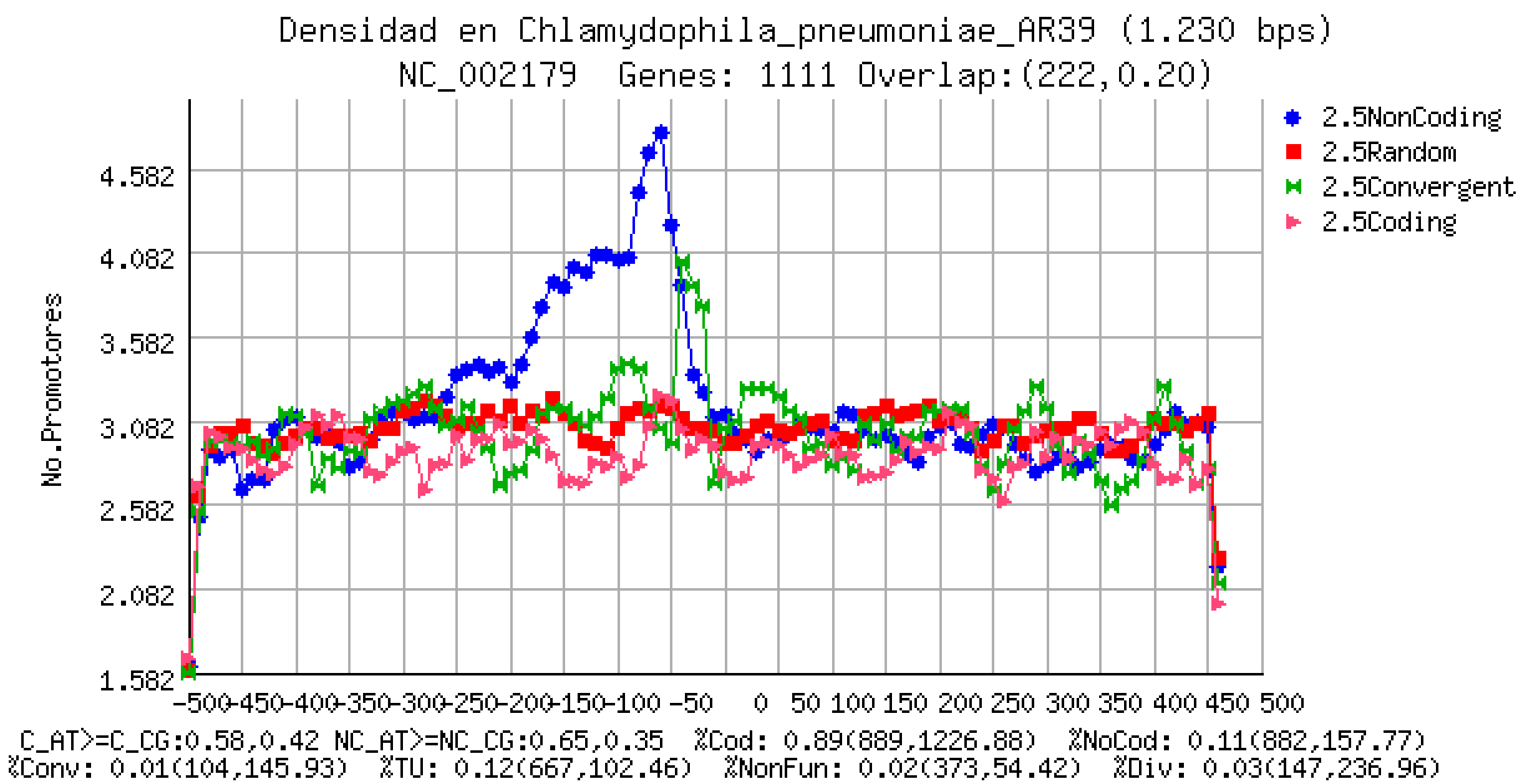
Densidad en Chlamydophila_pneumoniae_AR39 (1.230 bps)
NC_002179 Genes: 1111 Overlap:(222,0.20)
2.5NonCoding
2.5Random
2.5Convergent
2.5Coding
No.Promotores
4.582
4.082
3.582
3.082
2.582
2.082
1.582
-500-450-400-350-300-250-200-150-100 -50 0 50 100 150 200 250 300 350 400 450 500
C_AT>=C_CG:0.58,0.42 NC_AT>=NC_CG:0.65,0.35 %Cod: 0.89(889,1226.88) %NoCod: 0.11(882,157.77)
%Conv: 0.01(104,145.93) %TU: 0.12(667,102.46) %NonFun: 0.02(373,54.42) %Div: 0.03(147,236.96)

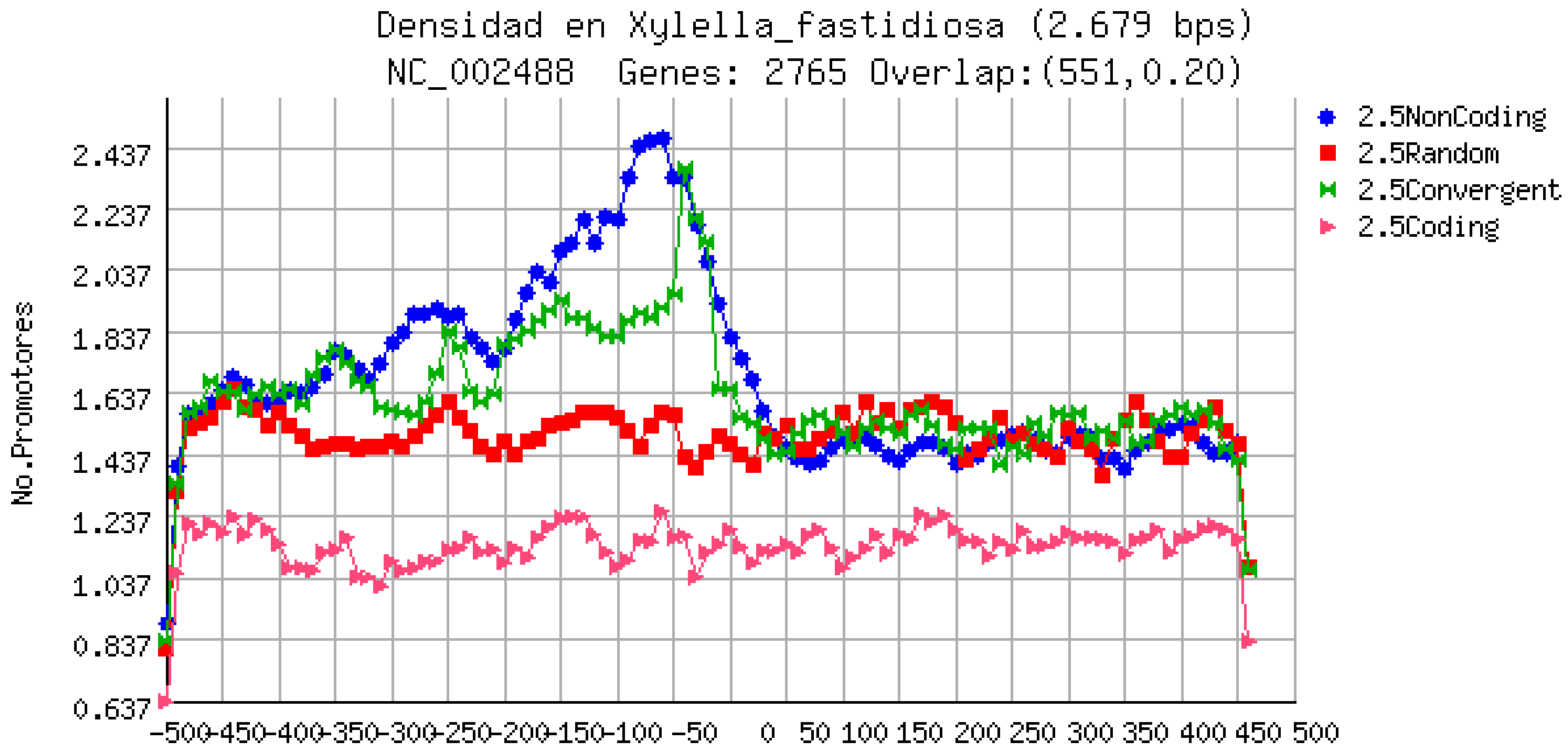

C_AT<C_CG:0.46,0.54 NC_AT>=NC_CG:0.53,0.47 %Cod: 0.83(2214,1007.32) %NoCod: 0.17(2192,204.88)
Conv: 0.03(406,225.40) %TU: 0.17(1826,113.46) %NonFun: 0.04(844,119.27) %Div: 0.04(397,274.32)

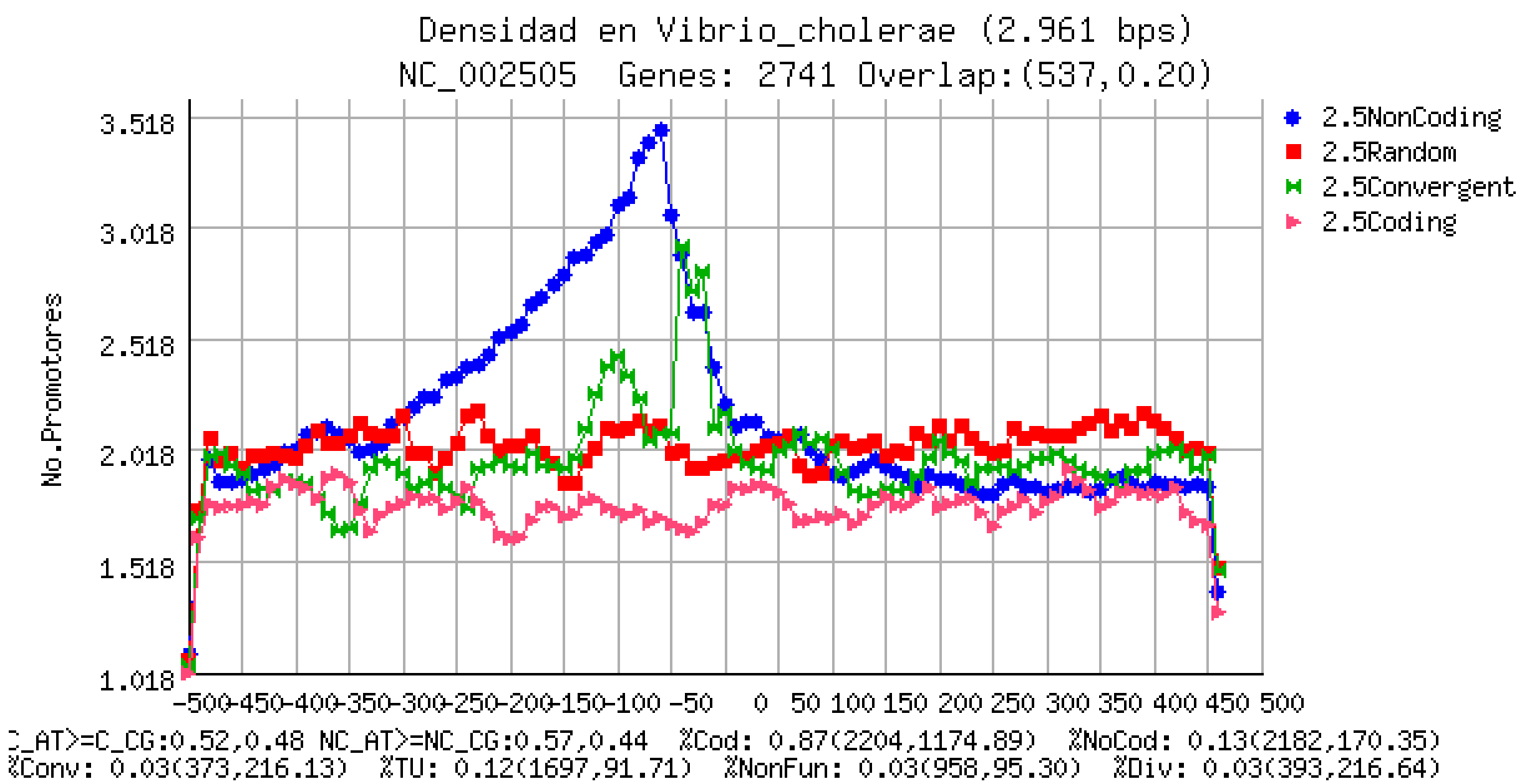
Densidad en Vibrio_cholerae (2.961 bps)
NC_002505 Genes: 2741 Overlap:(537,0.20)
No.Promotores
3.518
3.018
2.518
2.018
1.518
1.018
-500 -450 -400 -350 -300 -250 -200 -150 -100 -50 0 50 100 150 200 250 300 350 400 450 500
2.5NonCoding
2.5Random
2.5Convergent
2.5Coding
C_AT>=C_CG:0.52,0.48 NC_AT>=NC_CG:0.57,0.44 %Cod: 0.87(2204,1174.89) %NoCod: 0.13(2182,170.35)
%Conv: 0.03(373,216.13) %TU: 0.12(1697,91.71) %NonFun: 0.03(958,95.30) %Div: 0.03(393,216.64)

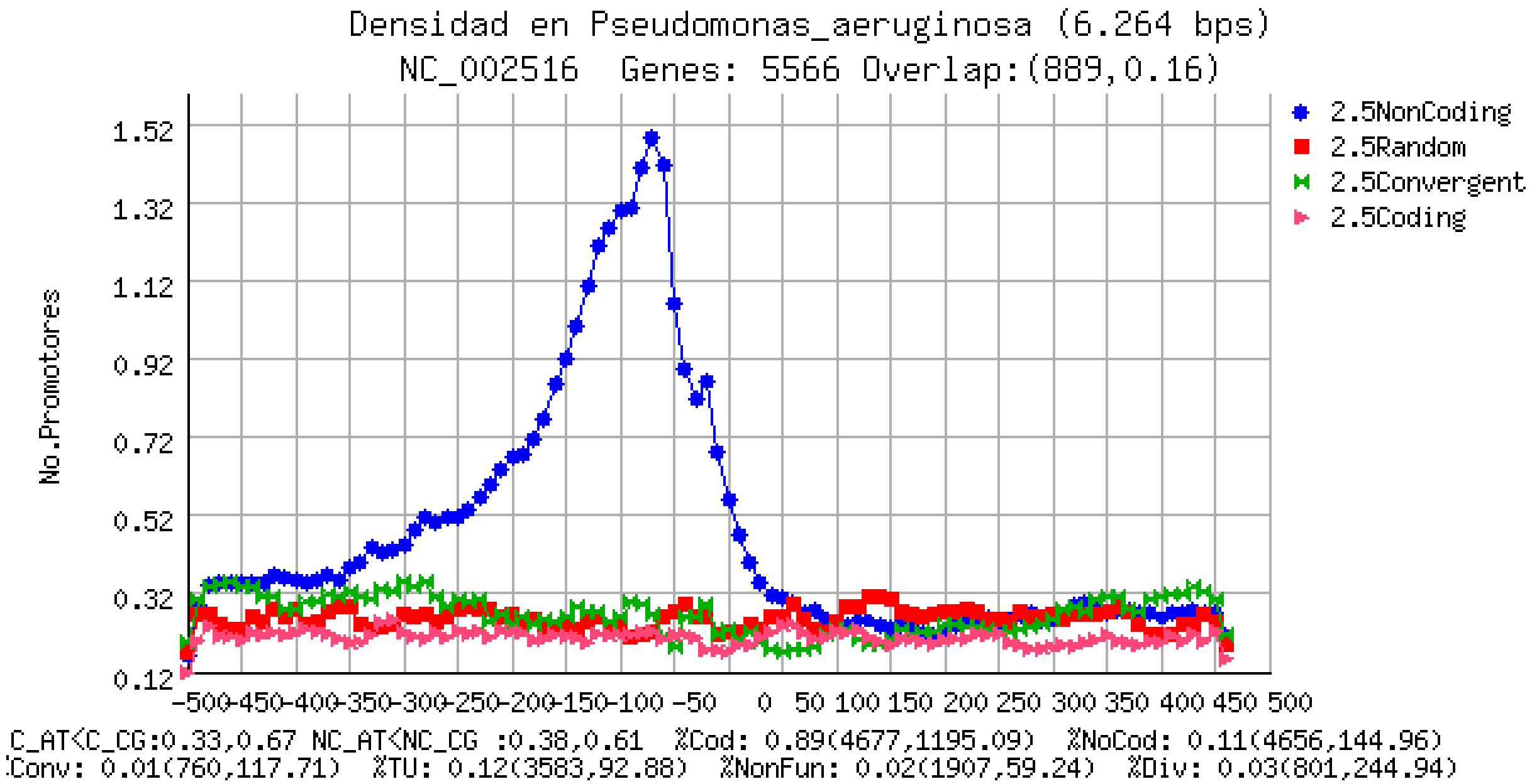
Densidad en Pseudomonas_aeruginosa (6.264 bps)
NC_002516 Genes: 5566 Overlap:(889,0.16)
No.Promotores
1.52
1.32
1.12
0.92
0.72
0.52
0.32
0.12
-500-450-400-350-300-250-200-150-100 -50 0 50 100 150 200 250 300 350 400 450 500
2.5NonCoding
2.5Random
2.5Convergent
2.5Coding
C_AT<C_CG:0.33,0.67 NC_AT<NC_CG :0.38,0.61 %Cod: 0.89(4677,1195.09) %NoCod: 0.11(4656,144.96)
Conv: 0.01(760,117.71) %TU: 0.12(3583,92.88) %NonFun: 0.02(1907,59.24) %Div: 0.03(801,244.94)

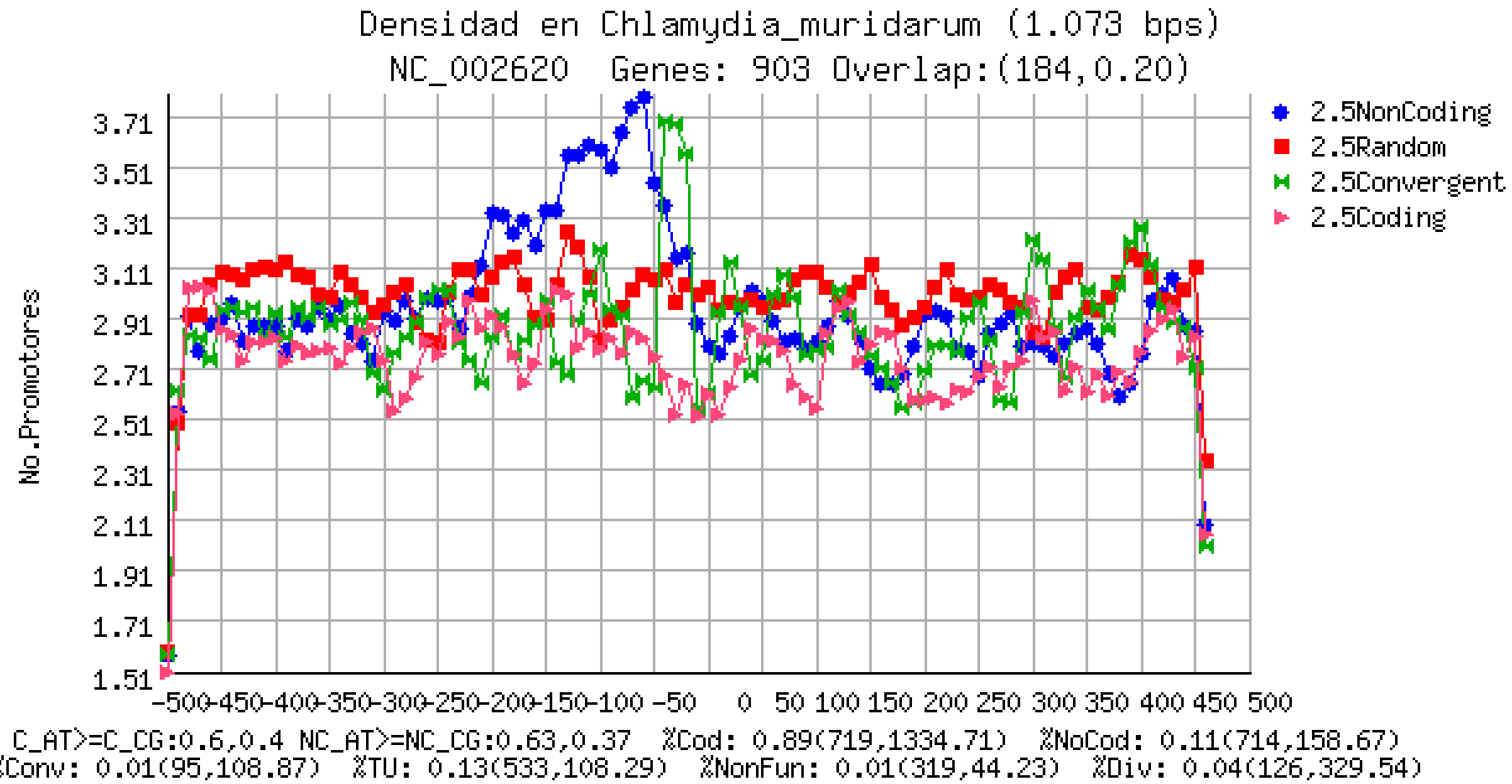
Densidad en Chlamydia_muridarum (1.073 bps)
NC_002620 Genes: 903 Overlap:(184,0.20)
No.Promotores
3.71
3.51
3.31
3.11
2.91
2.71
2.51
2.31
2.11
1.91
1.71
1.51
-500-450-400-350-300-250-200-150-100 -50 0 50 100 150 200 250 300 350 400 450 500
2.5NonCoding
2.5Random
2.5Convergent
2.5Coding
C_AT>=C_CG:0.6,0.4 NC_AT>=NC_CG:0.63,0.37 %Cod: 0.89(719,1334.71) %NoCod: 0.11(714,158.67)
%Conv: 0.01(95,108.87) %TU: 0.13(533,108.29) %NonFun: 0.01(319,44.23) %Div: 0.04(126,329.54)

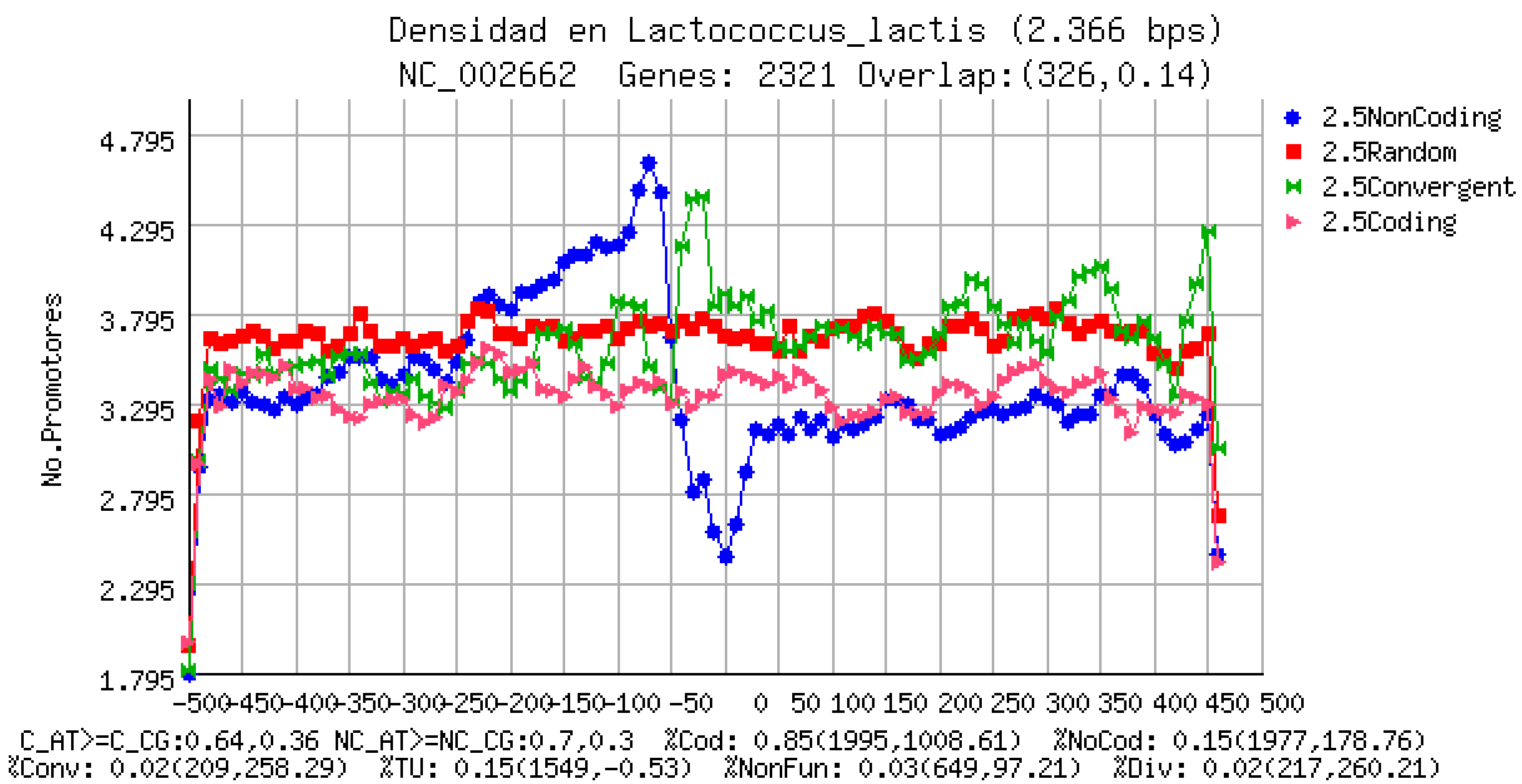
Densidad en Lactococcus_lactis (2.366 bps)
NC_002662 Genes: 2321 Overlap:(326,0.14)
No.Promotores
4.795
4.295
3.795
3.295
2.795
2.295
1.795
-500-450-400-350-300-250-200-150-100 -50 0 50 100 150 200 250 300 350 400 450 500
2.5NonCoding
2.5Random
2.5Convergent
2.5Coding
C_AT>=C_CG:0.64,0.36 NC_AT>=NC_CG:0.7,0.3 %Cod: 0.85(1995,1008.61) %NoCod: 0.15(1977,178.76)
%Conv: 0.02(209,258.29) %TU: 0.15(1549,-0.53) %NonFun: 0.03(649,97.21) %Div: 0.02(217,260.21)

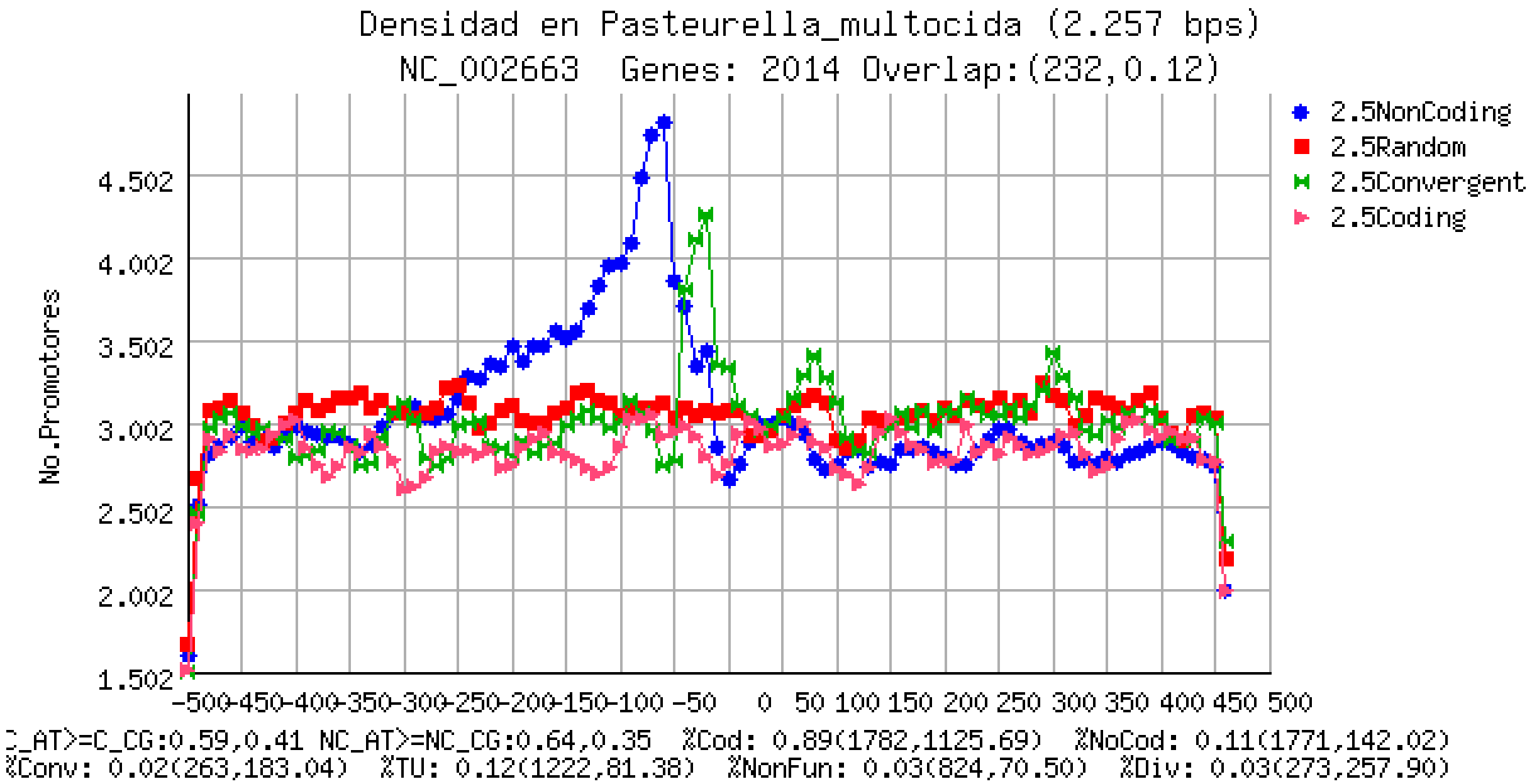
Densidad en Pasteurella_multocida (2.257 bps)
NC_002663 Genes: 2014 Overlap:(232,0.12)
No.Promotores
4.502
4.002
3.502
3.002
2.502
2.002
1.502
-500 -450 -400 -350 -300 -250 -200 -150 -100 -50 0 50 100 150 200 250 300 350 400 450 500
2.5NonCoding
2.5Random
2.5Convergent
2.5Coding
C_AT>=C_CG:0.59,0.41 NC_AT>=NC_CG:0.64,0.35 %Cod: 0.89(1782,1125.69) %NoCod: 0.11(1771,142.02)
%Conv: 0.02(263,183.04) %TU: 0.12(1222,81.38) %NonFun: 0.03(824,70.50) %Div: 0.03(273,257.90)

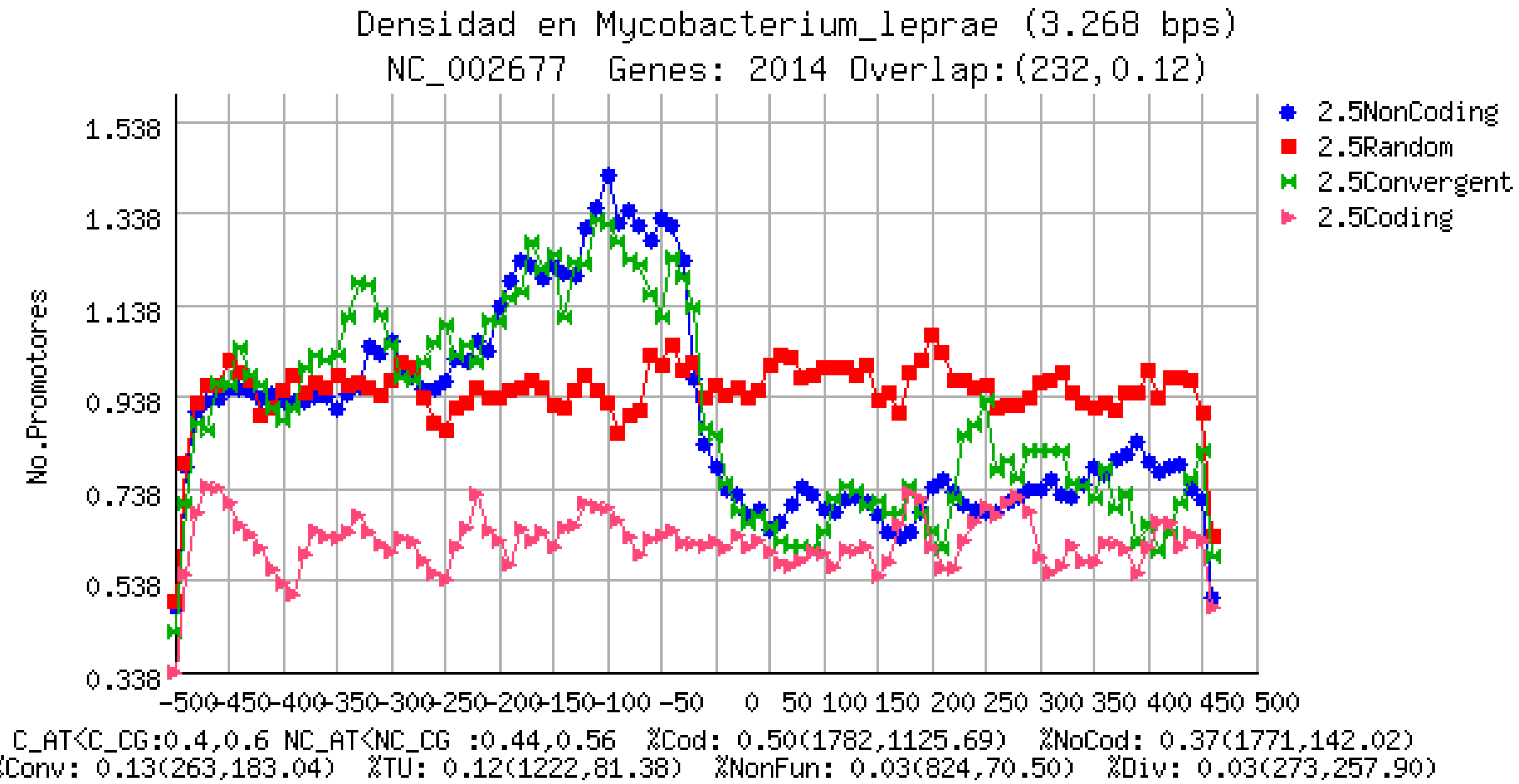
Densidad en Mycobacterium_leprae (3.268 bps)
NC_002677 Genes: 2014 Overlap:(232,0.12)
2.5NonCoding
2.5Random
2.5Convergent
2.5Coding
No.Promotores
1.538
1.338
1.138
0.938
0.738
0.538
0.338
-500 -450 -400 -350 -300 -250 -200 -150 -100 -50 0 50 100 150 200 250 300 350 400 450 500
C_AT<C_CG:0.4,0.6 NC_AT<NC_CG :0.44,0.56 %Cod: 0.50(1782,1125.69) %NoCod: 0.37(1771,142.02)
%Conv: 0.13(263,183.04) %TU: 0.12(1222,81.38) %NonFun: 0.03(824,70.50) %Div: 0.03(273,257.90)

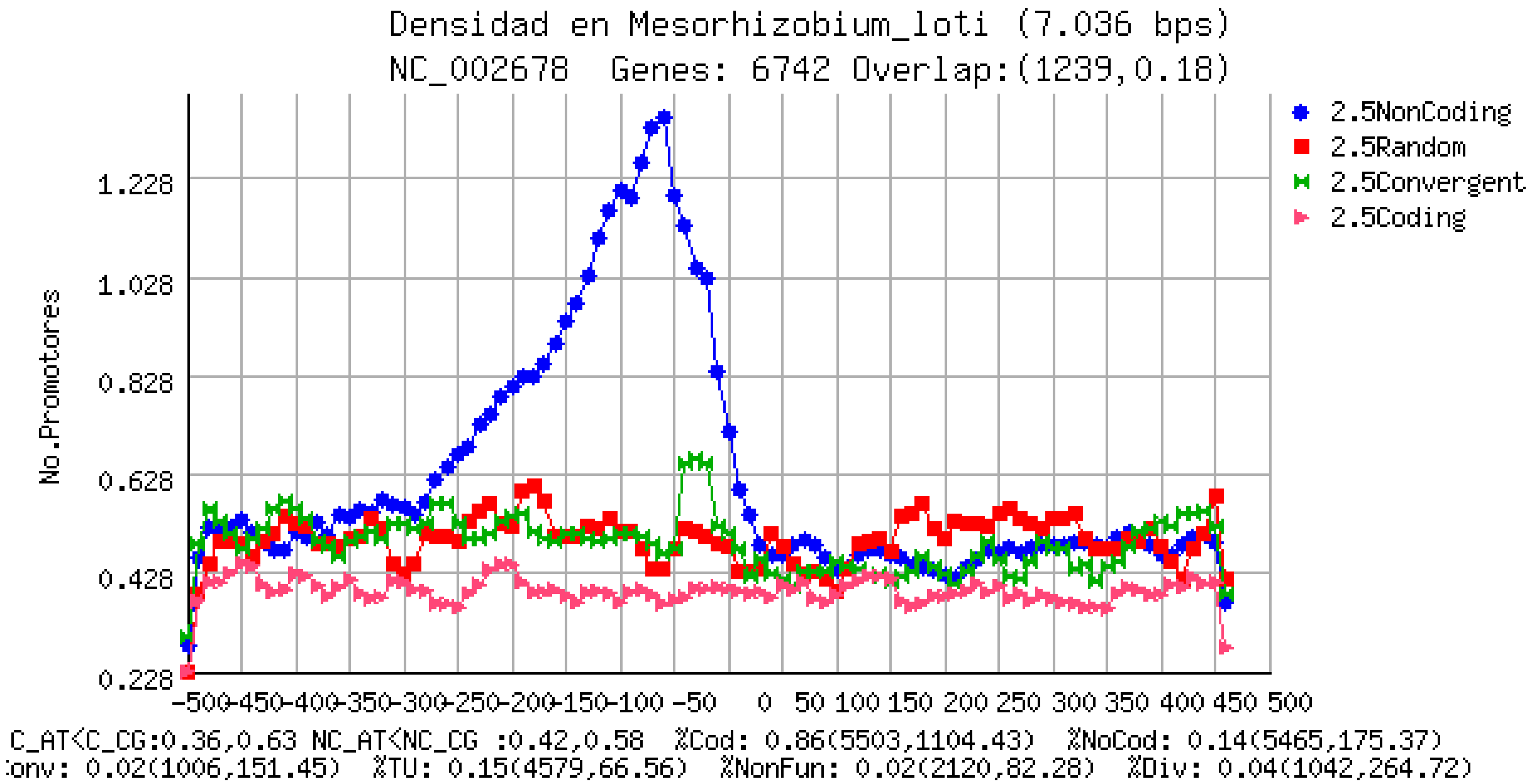
Densidad en Mesorhizobium_loti (7.036 bps)
NC_002678 Genes: 6742 Overlap:(1239,0.18)
2.5NonCoding
2.5Random
2.5Convergent
2.5Coding
No.Promotores
1.228
1.028
0.828
0.628
0.428
0.228
-500-450-400-350-300-250-200-150-100 -50 0 50 100 150 200 250 300 350 400 450 500
C_AT<C_CG:0.36,0.63 NC_AT<NC_CG :0.42,0.58 %Cod: 0.86(5503,1104.43) %NoCod: 0.14(5465,175.37)
onv: 0.02(1006,151.45) %TU: 0.15(4579,66.56) %NonFun: 0.02(2120,82.28) %Div: 0.04(1042,264.72)

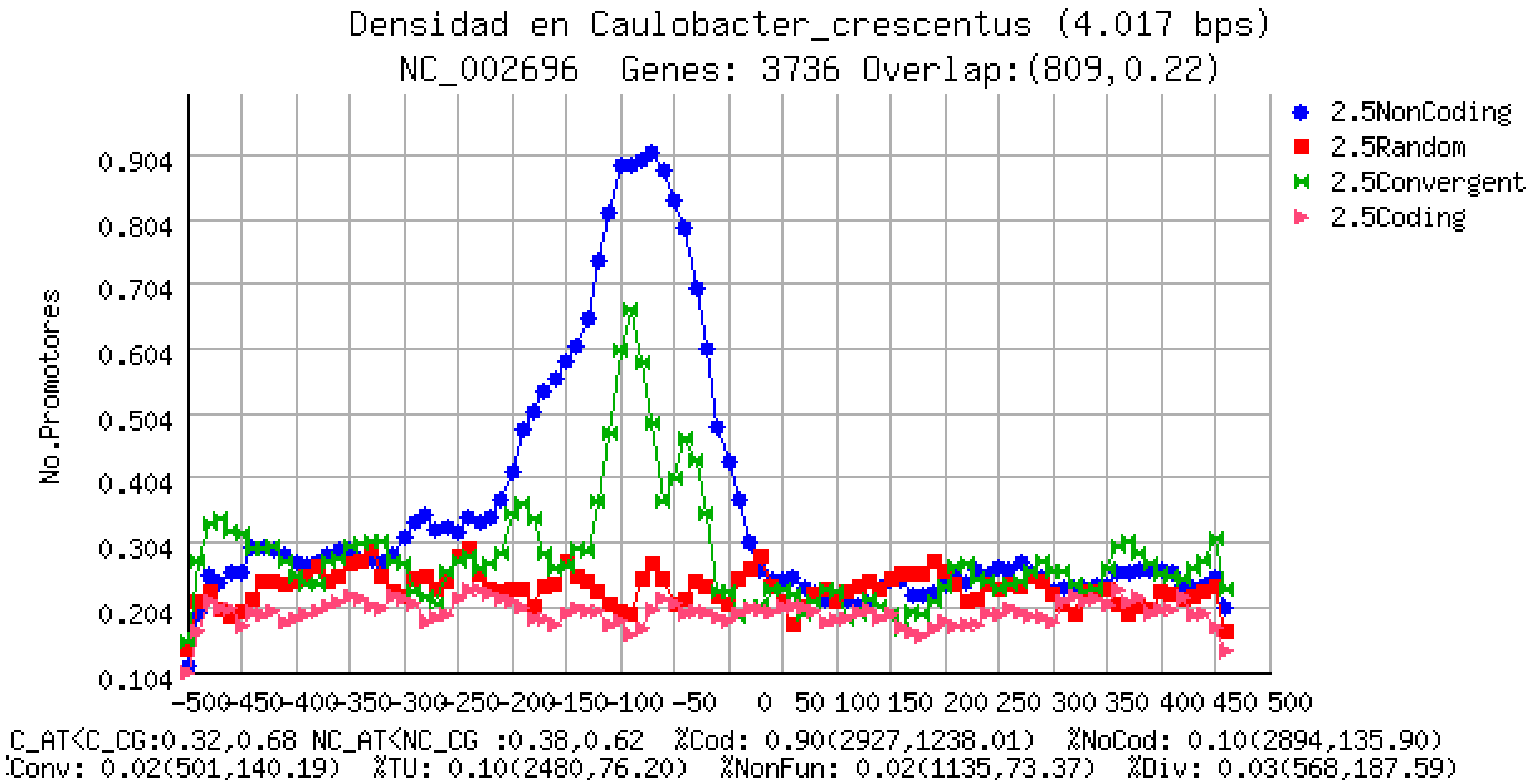
Densidad en Caulobacter_crescentus (4.017 bps)
NC_002696 Genes: 3736 Overlap:(809,0.22)
No.Promotores
0.904
0.804
0.704
0.604
0.504
0.404
0.304
0.204
0.104
-500-450-400-350-300-250-200-150-100 -50 0 50 100 150 200 250 300 350 400 450 500
2.5NonCoding
2.5Random
2.5Convergent
2.5Coding
C_AT<C_CG:0.32,0.68 NC_AT<NC_CG :0.38,0.62 %Cod: 0.90(2927,1238.01) %NoCod: 0.10(2894,135.90)
%Conv: 0.02(501,140.19) %TU: 0.10(2480,76.20) %NonFun: 0.02(1135,73.37) %Div: 0.03(568,187.59)

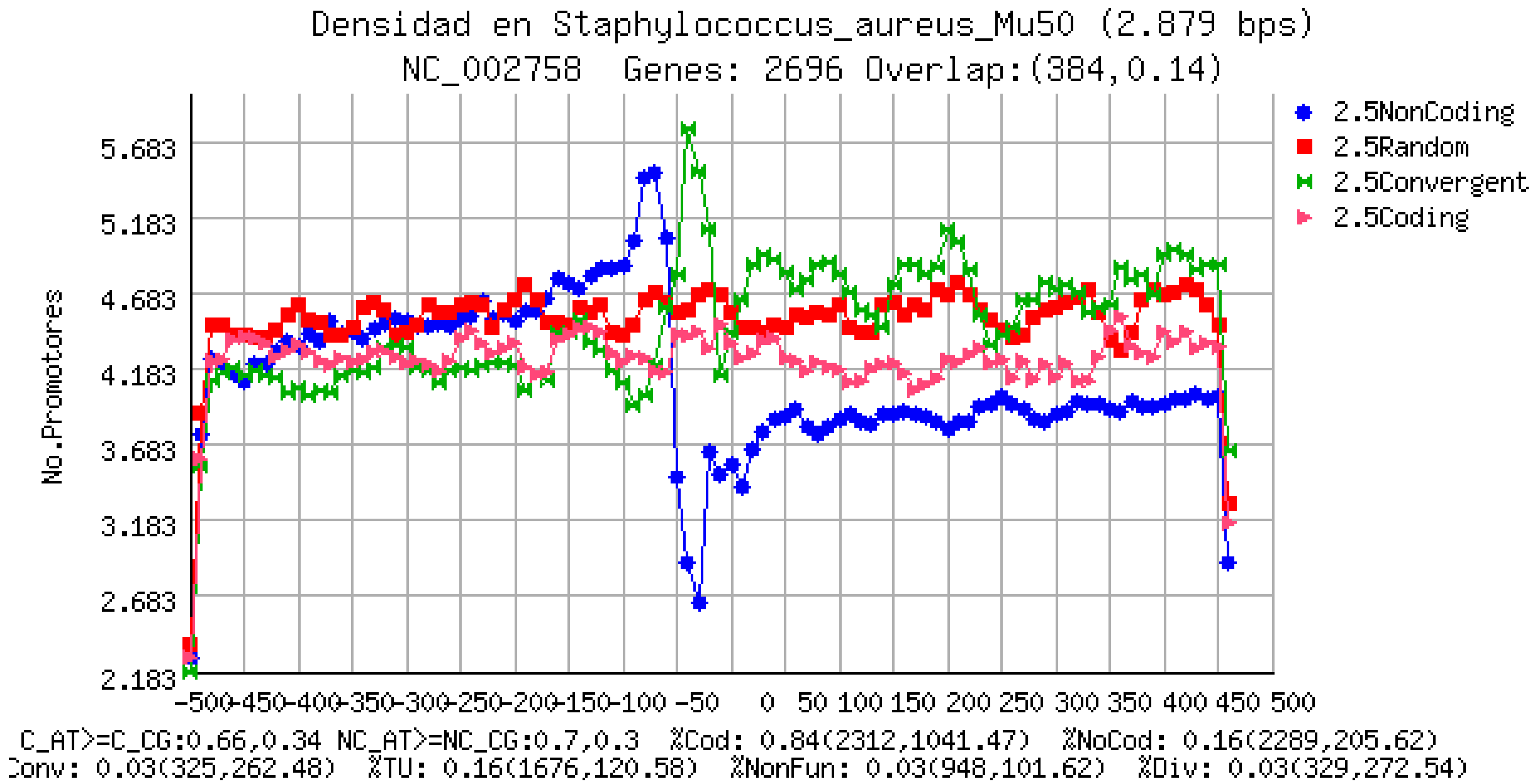
Densidad en Staphylococcus_aureus_Mu50 (2.879 bps)
NC_002758 Genes: 2696 Overlap:(384,0.14)
No.Promotores
5.683
5.183
4.683
4.183
3.683
3.183
2.683
2.183
-500 -450 -400 -350 -300 -250 -200 -150 -100 -50 0 50 100 150 200 250 300 350 400 450 500
2.5NonCoding
2.5Random
2.5Convergent
2.5Coding
C_AT>=C_CG:0.66,0.34 NC_AT>=NC_CG:0.7,0.3 %Cod: 0.84(2312,1041.47) %NoCod: 0.16(2289,205.62)
Conv: 0.03(325,262.48) %TU: 0.16(1676,120.58) %NonFun: 0.03(948,101.62) %Div: 0.03(329,272.54)

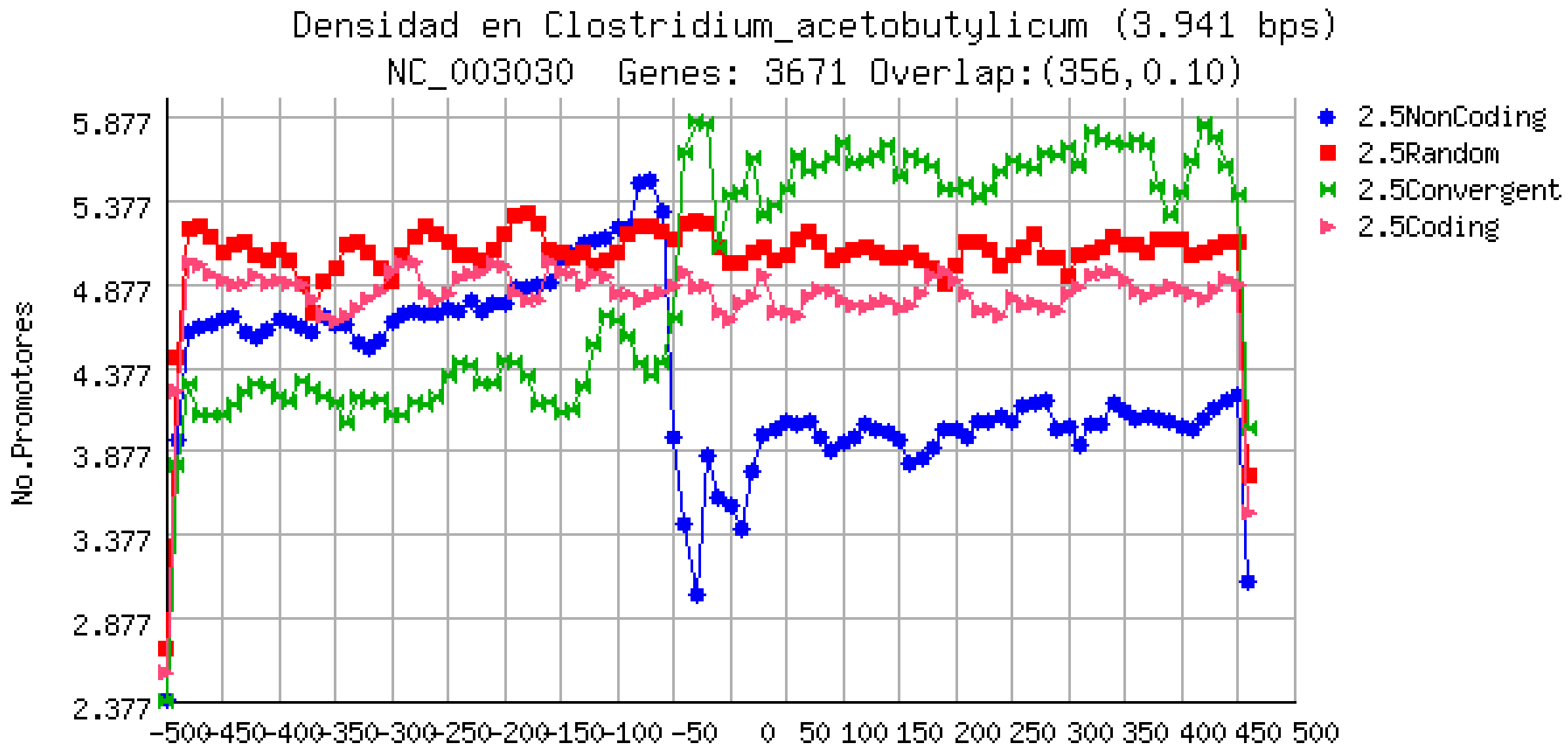

C_AT>=C_CG:0.68,0.32 NC_AT>=NC_CG:0.73,0.27 %Cod: 0.86(3315,1018.56) %NoCod: 0.14(3278,172.17)
Conv: 0.02(391,154.03) %TU: 0.16(2309,128.89) %NonFun: 0.02(1384,57.21) %Div: 0.03(413,326.60)

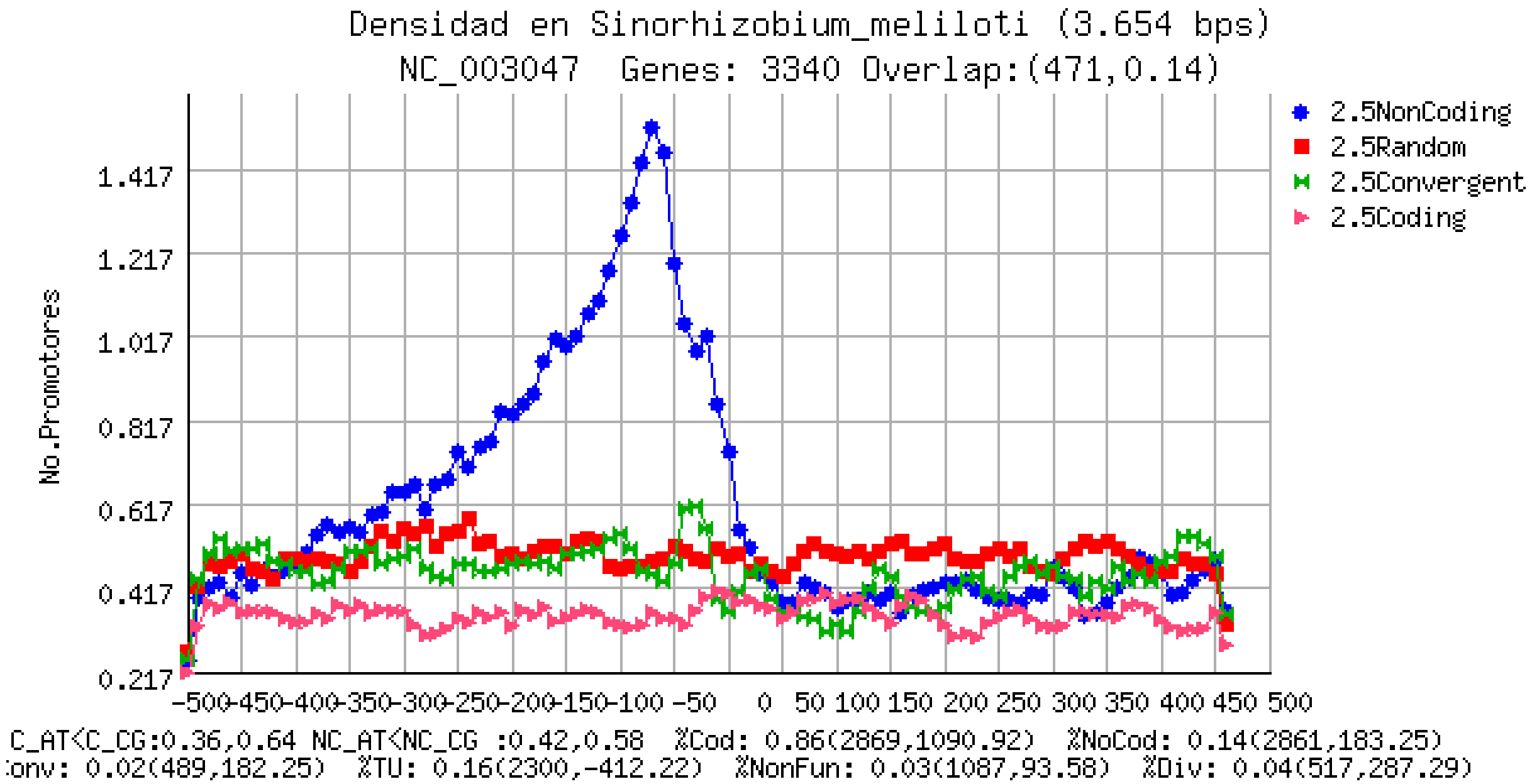
Densidad en Sinorhizobium_meliloti (3.654 bps)
NC_003047 Genes: 3340 Overlap:(471,0.14)
No.Promotores
1.417
1.217
1.017
0.817
0.617
0.417
0.217
-500-450-400-350-300-250-200-150-100 -50 0 50 100 150 200 250 300 350 400 450 500
2.5NonCoding
2.5Random
2.5Convergent
2.5Coding
C_AT<C_CG:0.36,0.64 NC_AT<NC_CG :0.42,0.58 %Cod: 0.86(2869,1090.92) %NoCod: 0.14(2861,183.25)
:onv: 0.02(489,182.25) %TU: 0.16(2300,-412.22) %NonFun: 0.03(1087,93.58) %Div: 0.04(517,287.29)

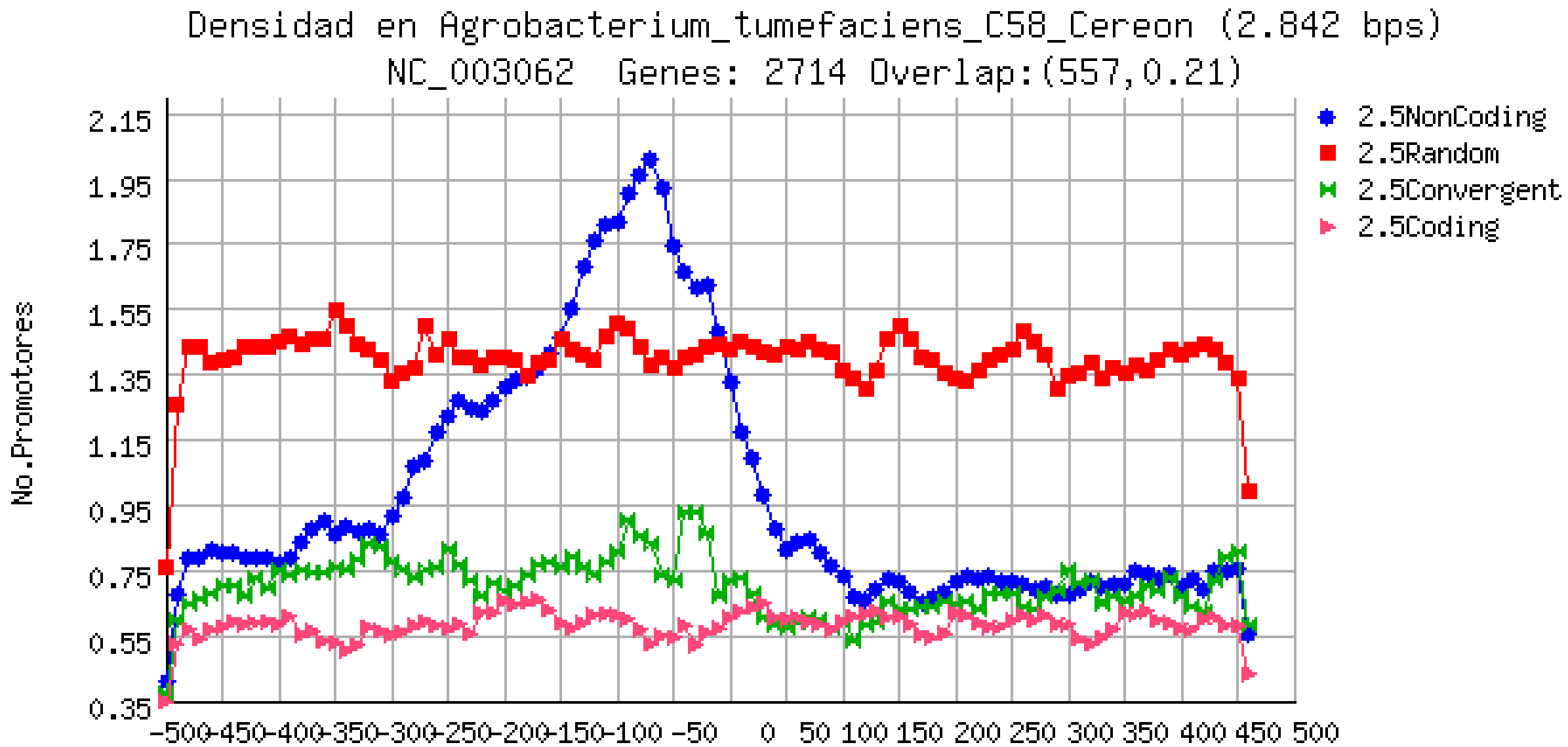

C_AT<C_CG:0.4,0.6 NC_AT<NC_CG :0.47,0.53 %Cod: 0.89(2157,1170.55) %NoCod: 0.11(2144,147.72)
%Conv: 0.01(384,108.93) %TU: 0.12(1795,87.17) %NonFun: 0.02(822,61.50) %Div: 0.03(385,209.28)

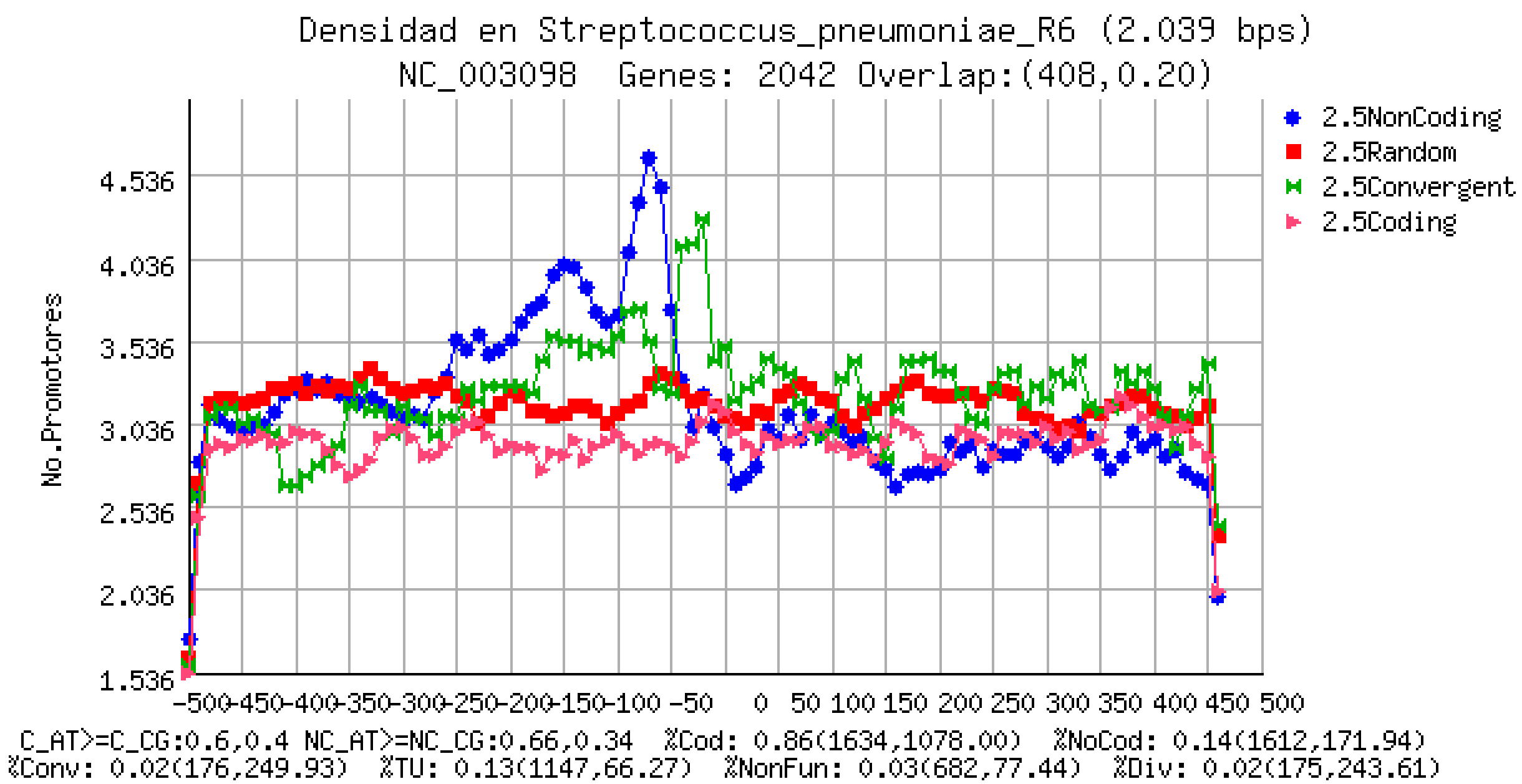
Densidad en Streptococcus_pneumoniae_R6 (2.039 bps)
NC_003098 Genes: 2042 Overlap:(408,0.20)
2.5NonCoding
2.5Random
2.5Convergent
2.5Coding
No.Promotores
4.536
4.036
3.536
3.036
2.536
2.036
1.536
-500 -450 -400 -350 -300 -250 -200 -150 -100 -50 0 50 100 150 200 250 300 350 400 450 500
C_AT>=C_CG:0.6,0.4 NC_AT>=NC_CG:0.66,0.34 %Cod: 0.86(1634,1078.00) %NoCod: 0.14(1612,171.94)
%Conv: 0.02(176,249.93) %TU: 0.13(1147,66.27) %NonFun: 0.03(682,77.44) %Div: 0.02(175,243.61)

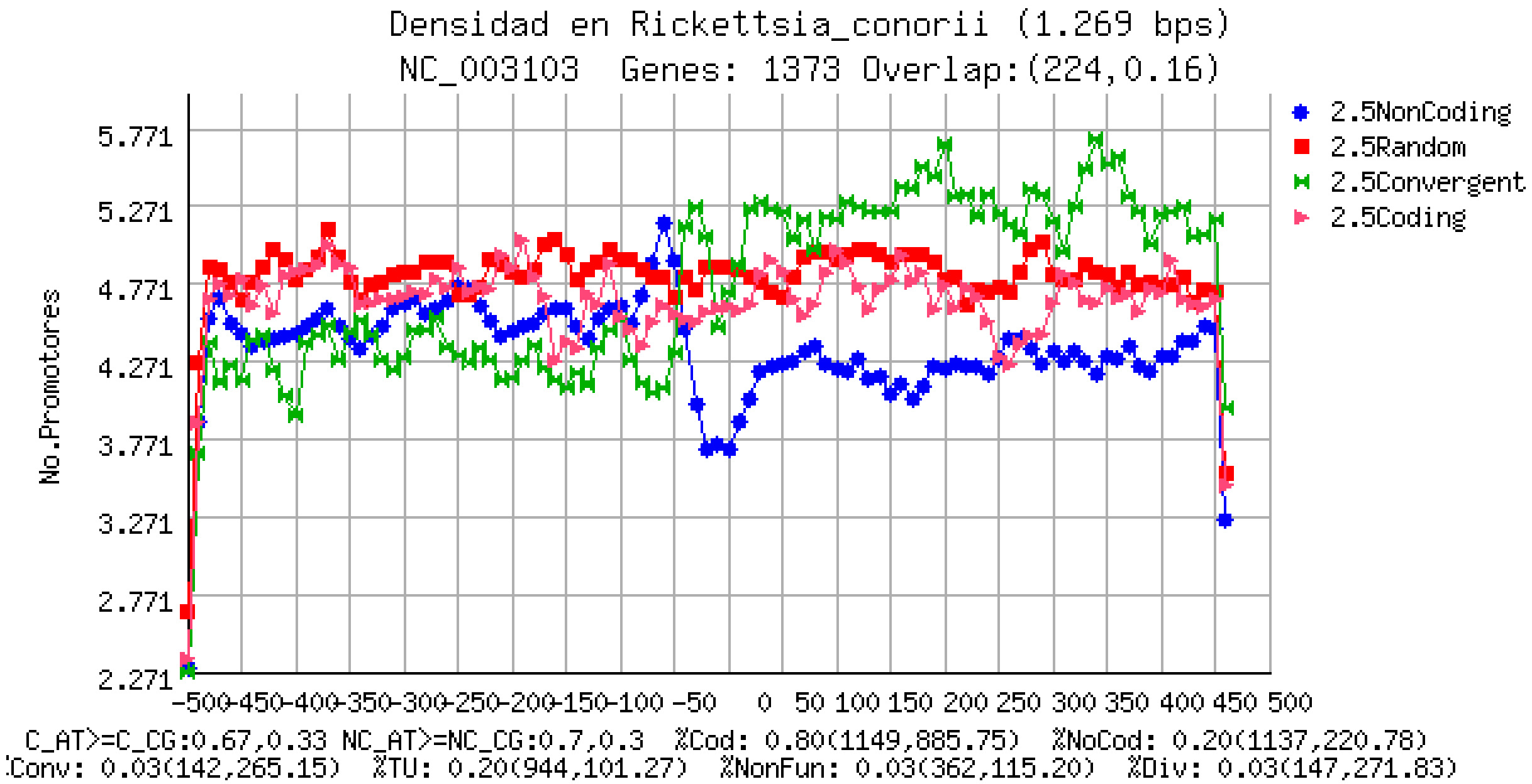
Densidad en Rickettsia_conorii (1.269 bps)
NC_003103 Genes: 1373 Overlap:(224,0.16)
2.5NonCoding
2.5Random
2.5Convergent
2.5Coding
No.Promotores
5.771
5.271
4.771
4.271
3.771
3.271
2.771
2.271
-500 -450 -400 -350 -300 -250 -200 -150 -100 -50 0 50 100 150 200 250 300 350 400 450 500
C_AT>=C_CG:0.67,0.33 NC_AT>=NC_CG:0.7,0.3 %Cod: 0.80(1149,885.75) %NoCod: 0.20(1137,220.78)
%Conv: 0.03(142,265.15) %TU: 0.20(944,101.27) %NonFun: 0.03(362,115.20) %Div: 0.03(147,271.83)

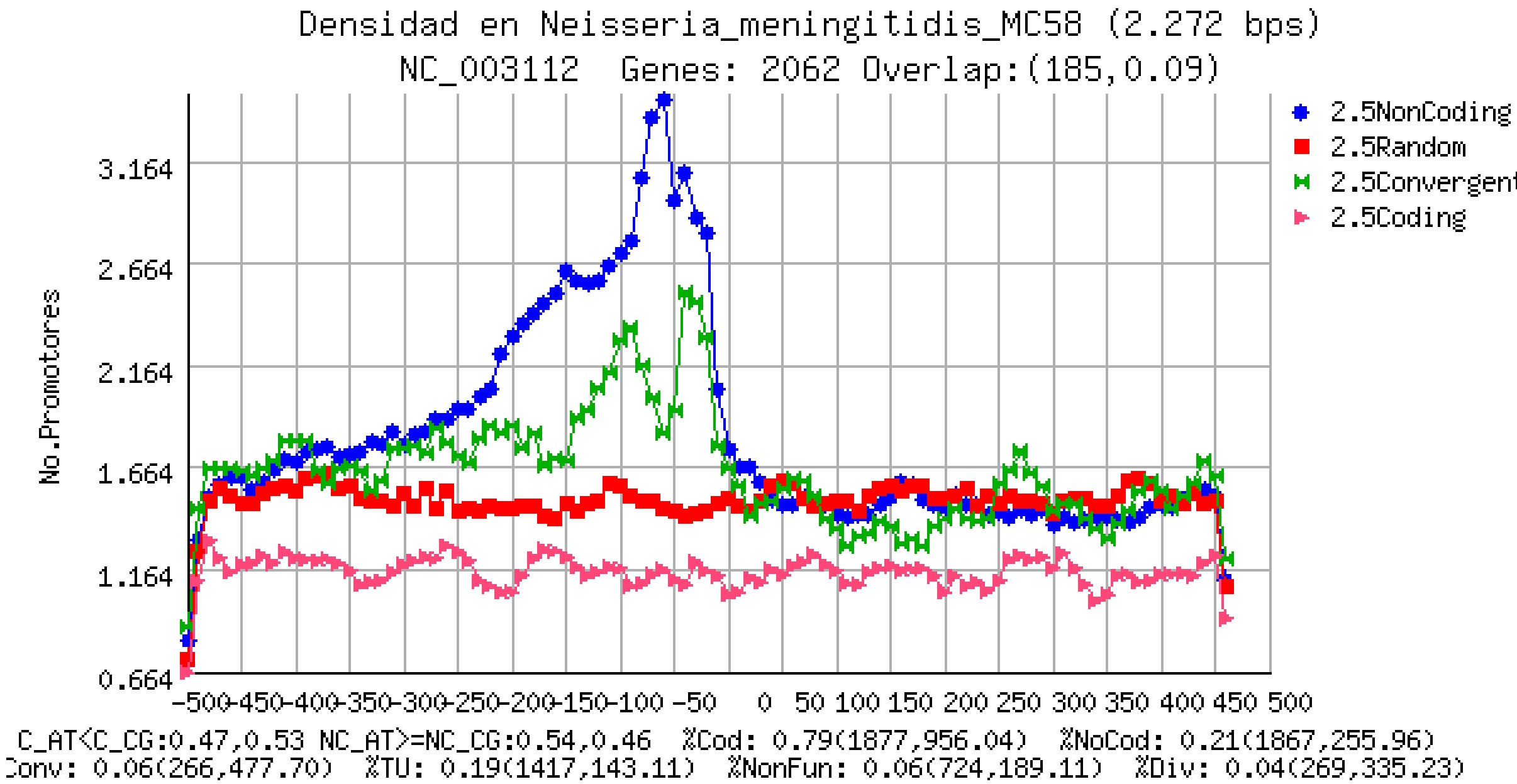
Densidad en Neisseria_meningitidis_MC58 (2.272 bps)
NC_003112 Genes: 2062 Overlap:(185,0.09)
2.5NonCoding
2.5Random
2.5Convergent
2.5Coding
No.Promotores
3.164
2.664
2.164
1.664
1.164
0.664
-500-450-400-350-300-250-200-150-100 -50 0 50 100 150 200 250 300 350 400 450 500
C_AT<C_CG:0.47,0.53 NC_AT>=NC_CG:0.54,0.46 %Cod: 0.79(1877,956.04) %NoCod: 0.21(1867,255.96)
Conv: 0.06(266,477.70) %TU: 0.19(1417,143.11) %NonFun: 0.06(724,189.11) %Div: 0.04(269,335.23)

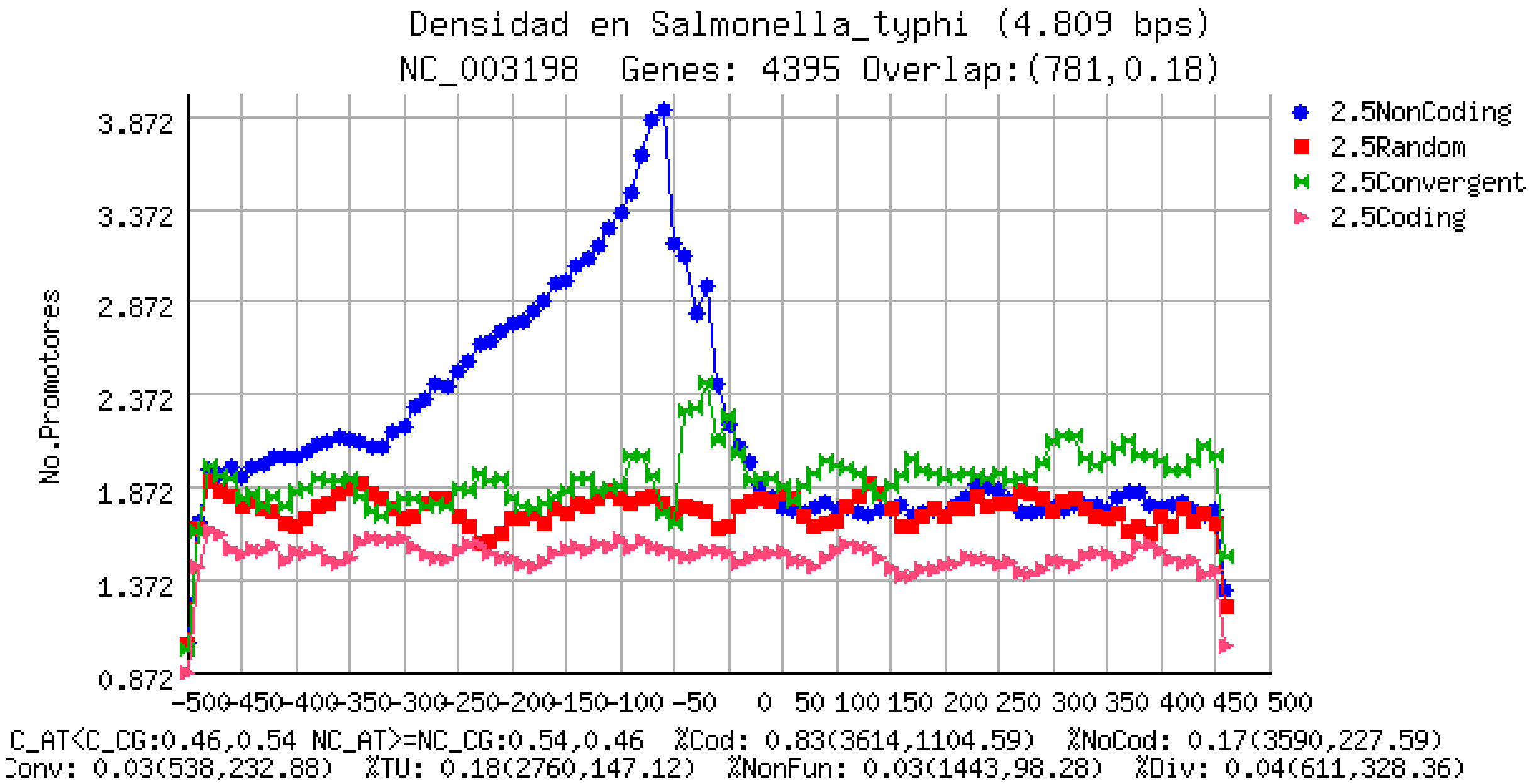
Densidad en Salmonella_typhi (4.809 bps)
NC_003198 Genes: 4395 Overlap:(781,0.18)
No.Promotores
3.872
3.372
2.872
2.372
1.872
1.372
0.872
-500 -450 -400 -350 -300 -250 -200 -150 -100 -50 0 50 100 150 200 250 300 350 400 450 500
2.5NonCoding
2.5Random
2.5Convergent
2.5Coding
C_AT<C_CG:0.46,0.54 NC_AT>=NC_CG:0.54,0.46 %Cod: 0.83(3614,1104.59) %NoCod: 0.17(3590,227.59)
Conv: 0.03(538,232.88) %TU: 0.18(2760,147.12) %NonFun: 0.03(1443,98.28) %Div: 0.04(611,328.36)

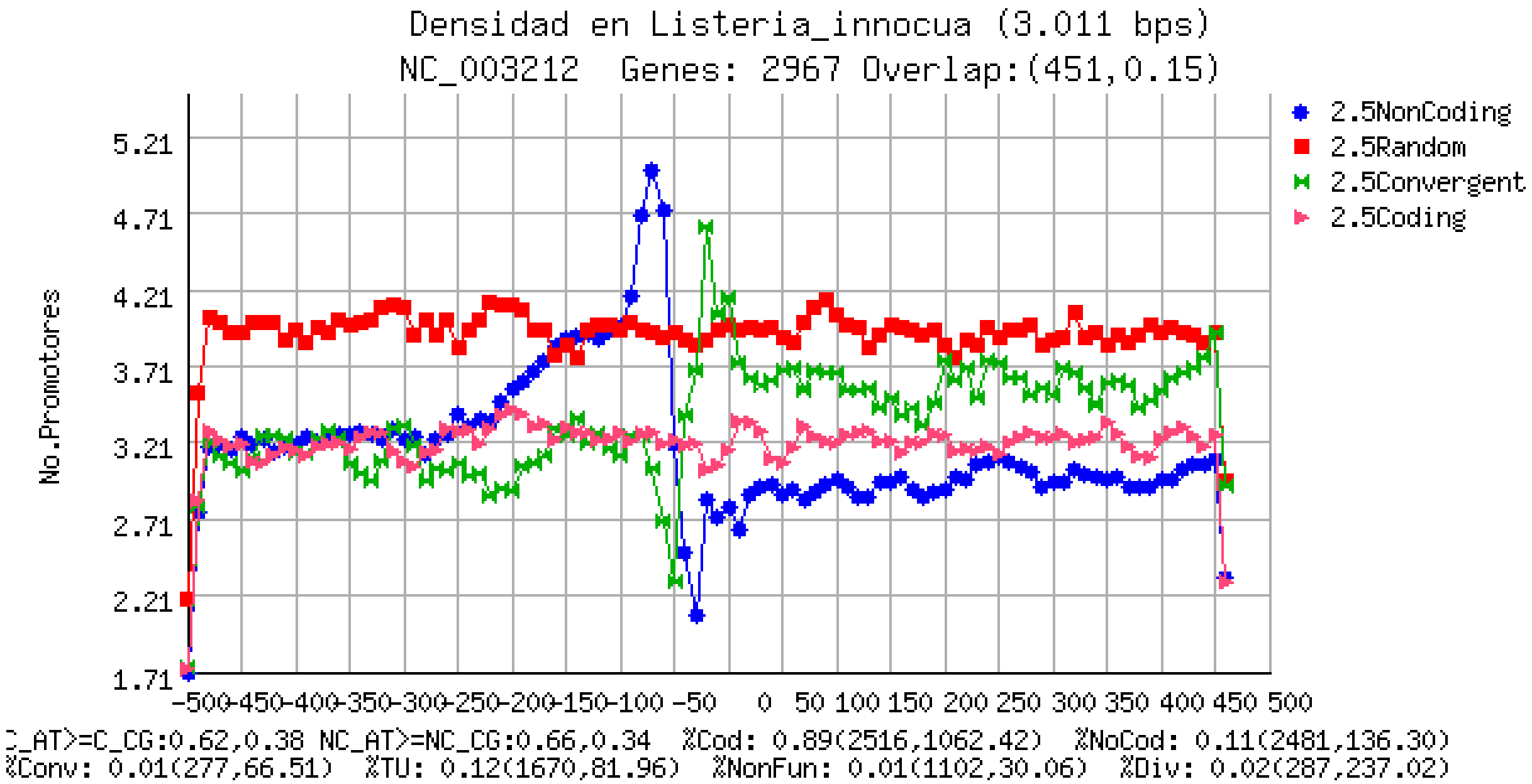
Densidad en Listeria_innocua (3.011 bps)
NC_003212 Genes: 2967 Overlap:(451,0.15)
No.Promotores
5.21
4.71
4.21
3.71
3.21
2.71
2.21
1.71
-500-450-400-350-300-250-200-150-100 -50 0 50 100 150 200 250 300 350 400 450 500
2.5NonCoding
2.5Random
2.5Convergent
2.5Coding
C_AT>=C_CG:0.62,0.38 NC_AT>=NC_CG:0.66,0.34 %Cod: 0.89(2516,1062.42) %NoCod: 0.11(2481,136.30)
%Conv: 0.01(277,66.51) %TU: 0.12(1670,81.96) %NonFun: 0.01(1102,30.06) %Div: 0.02(287,237.02)

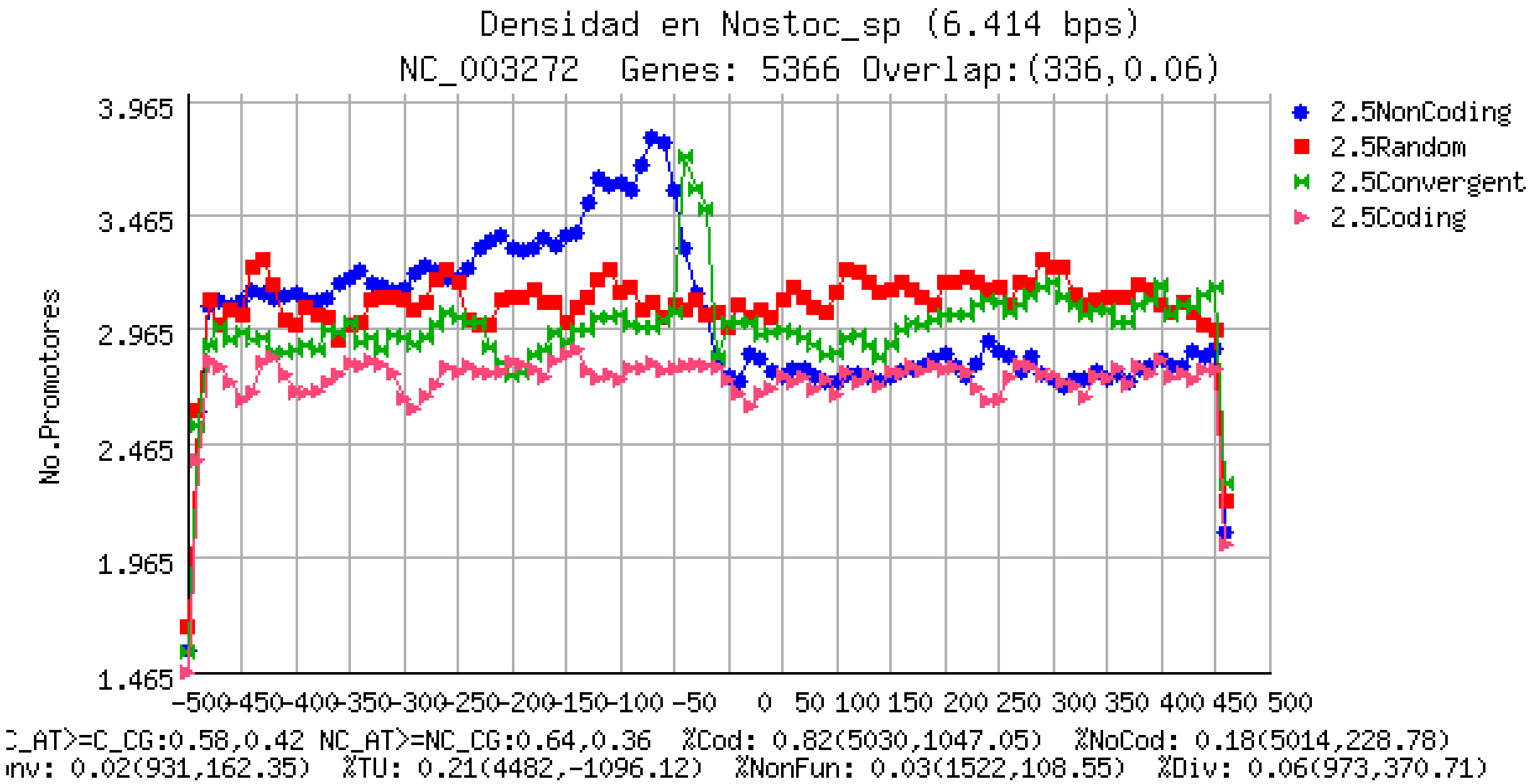
Densidad en Nostoc_sp (6.414 bps)
NC_003272 Genes: 5366 Overlap:(336,0.06)
3.965
3.465
2.965
2.465
1.965
1.465
No.Promotores
-500-450-400-350-300-250-200-150-100 -50 0 50 100 150 200 250 300 350 400 450 500
2.5NonCoding
2.5Random
2.5Convergent
2.5Coding
C_AT>=C_CG:0.58,0.42 NC_AT>=NC_CG:0.64,0.36 %Cod: 0.82(5030,1047.05) %NoCod: 0.18(5014,228.78)
nv: 0.02(931,162.35) %TU: 0.21(4482,-1096.12) %NonFun: 0.03(1522,108.55) %Div: 0.06(973,370.71)

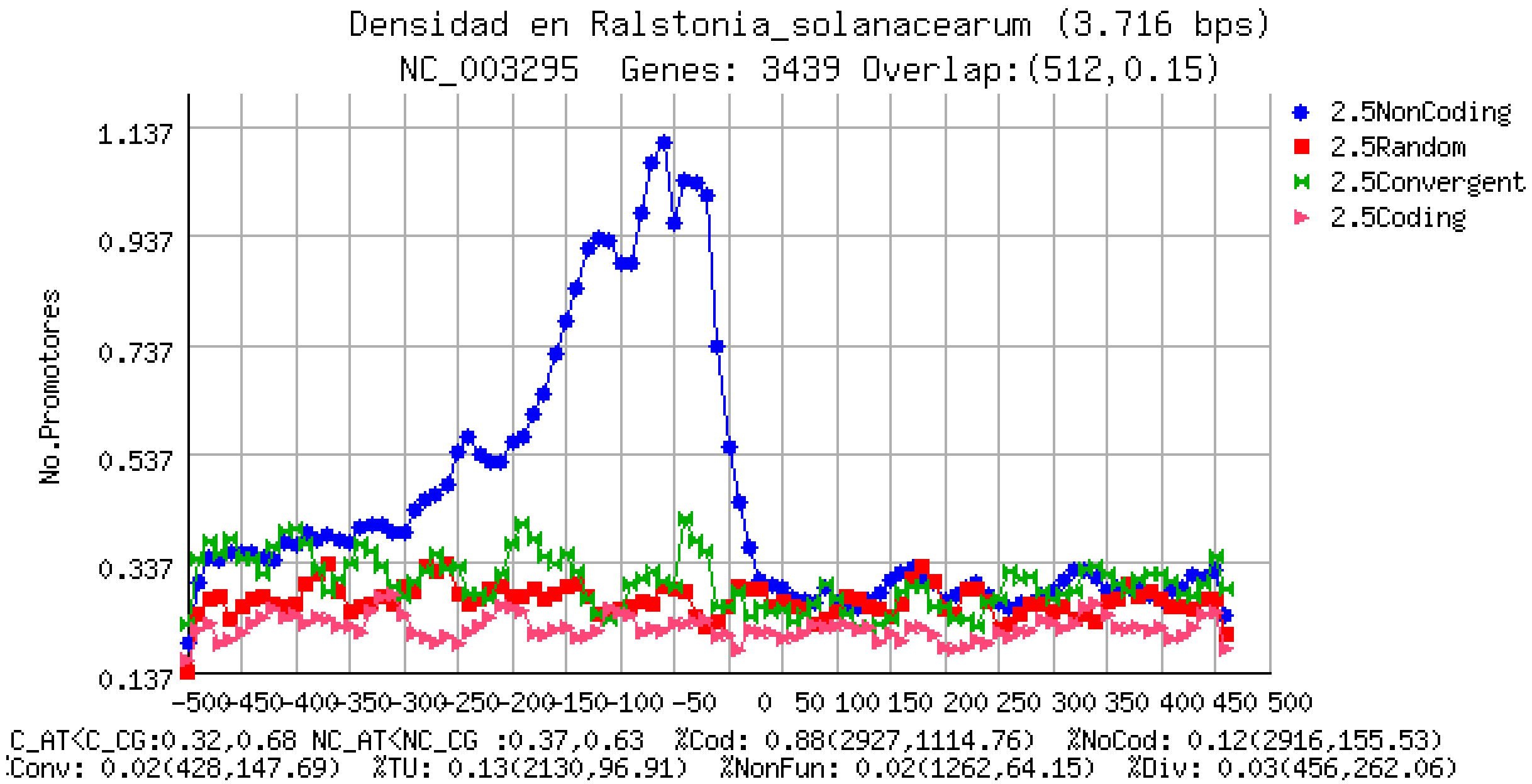
Densidad en Ralstonia_solanacearum (3.716 bps)
NC_003295 Genes: 3439 Overlap:(512,0.15)
No.Promotores
1.137
0.937
0.737
0.537
0.337
0.137
-500 -450 -400 -350 -300 -250 -200 -150 -100 -50 0 50 100 150 200 250 300 350 400 450 500
2.5NonCoding
2.5Random
2.5Convergent
2.5Coding
C_AT<C_CG:0.32,0.68 NC_AT<NC_CG :0.37,0.63 %Cod: 0.88(2927,1114.76) %NoCod: 0.12(2916,155.53)
Conv: 0.02(428,147.69) %TU: 0.13(2130,96.91) %NonFun: 0.02(1262,64.15) %Div: 0.03(456,262.06)

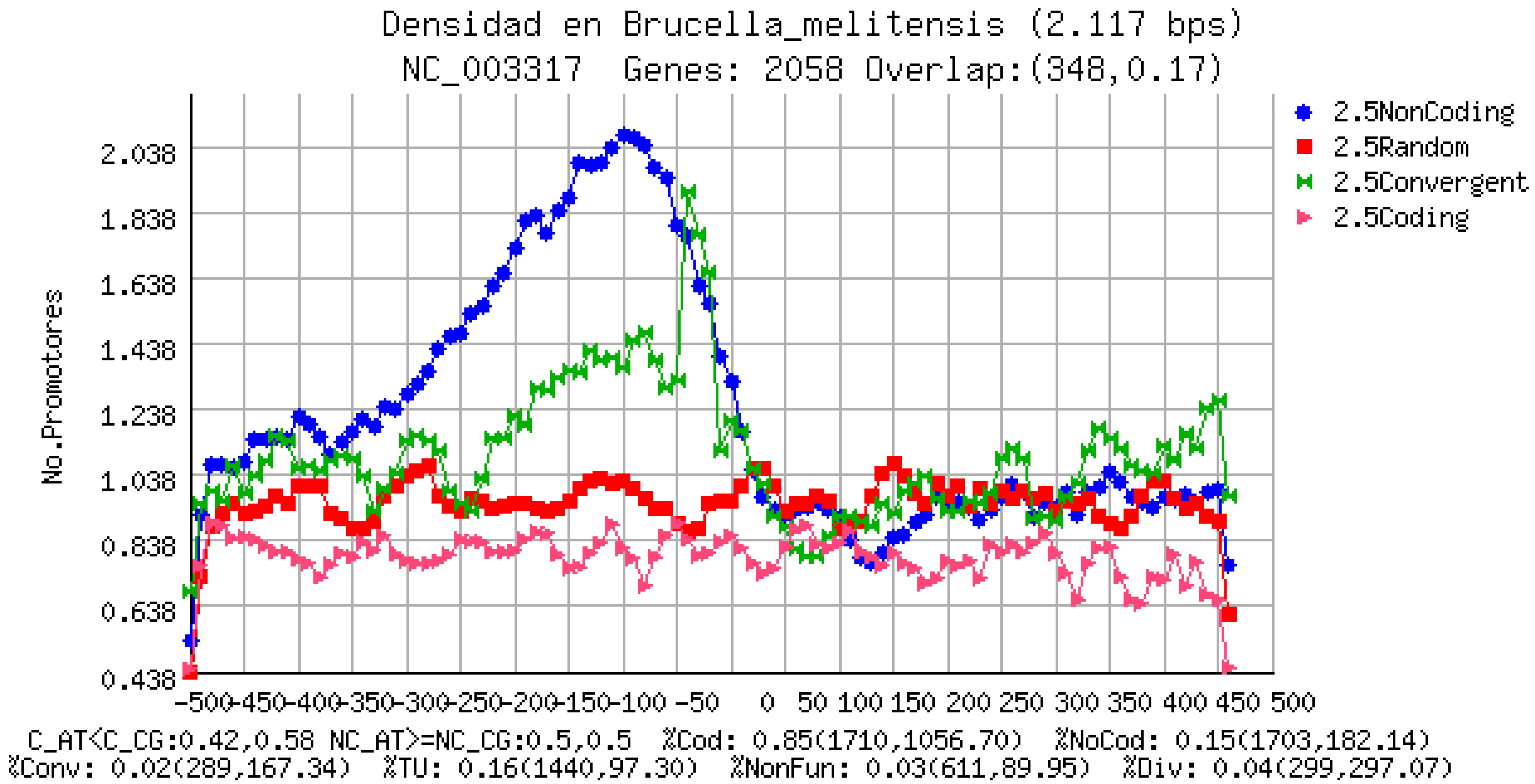
Densidad en Brucella_melitensis (2.117 bps)
NC_003317 Genes: 2058 Overlap:(348,0.17)
No.Promotores
2.038
1.838
1.638
1.438
1.238
1.038
0.838
0.638
0.438
-500-450-400-350-300-250-200-150-100 -50 0 50 100 150 200 250 300 350 400 450 500
2.5NonCoding
2.5Random
2.5Convergent
2.5Coding
C_AT<C_CG:0.42,0.58 NC_AT>=NC_CG:0.5,0.5 %Cod: 0.85(1710,1056.70) %NoCod: 0.15(1703,182.14)
%Conv: 0.02(289,167.34) %TU: 0.16(1440,97.30) %NonFun: 0.03(611,89.95) %Div: 0.04(299,297.07)

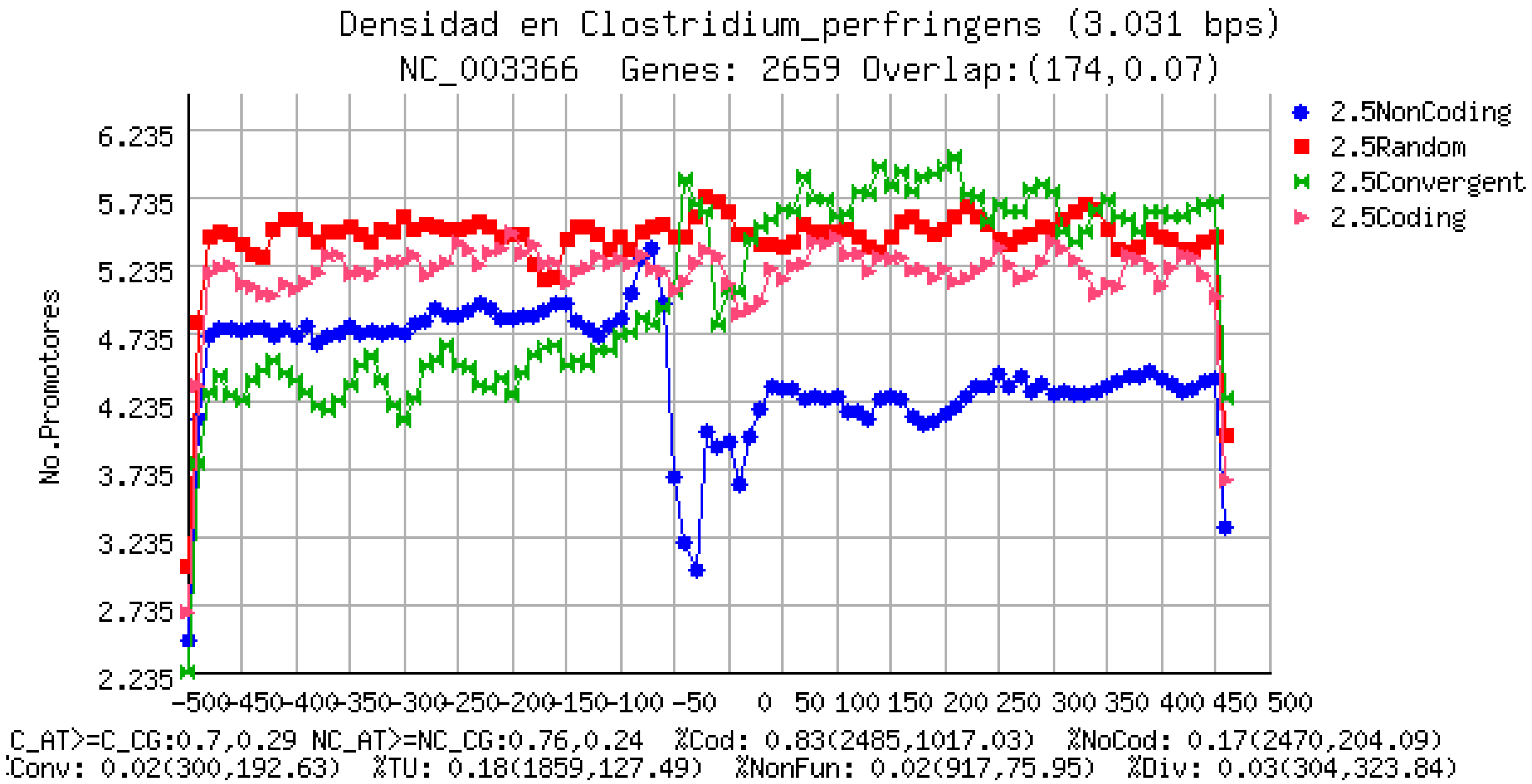
Densidad en Clostridium_perfringens (3.031 bps)
NC_003366 Genes: 2659 Overlap:(174,0.07)
No.Promotores
6.235
5.735
5.235
4.735
4.235
3.735
3.235
2.735
2.235
-500-450-400-350-300-250-200-150-100 -50 0 50 100 150 200 250 300 350 400 450 500
2.5NonCoding
2.5Random
2.5Convergent
2.5Coding
C_AT>=C_CG:0.7,0.29 NC_AT>=NC_CG:0.76,0.24 %Cod: 0.83(2485,1017.03) %NoCod: 0.17(2470,204.09)
Conv: 0.02(300,192.63) %TU: 0.18(1859,127.49) %NonFun: 0.02(917,75.95) %Div: 0.03(304,323.84)

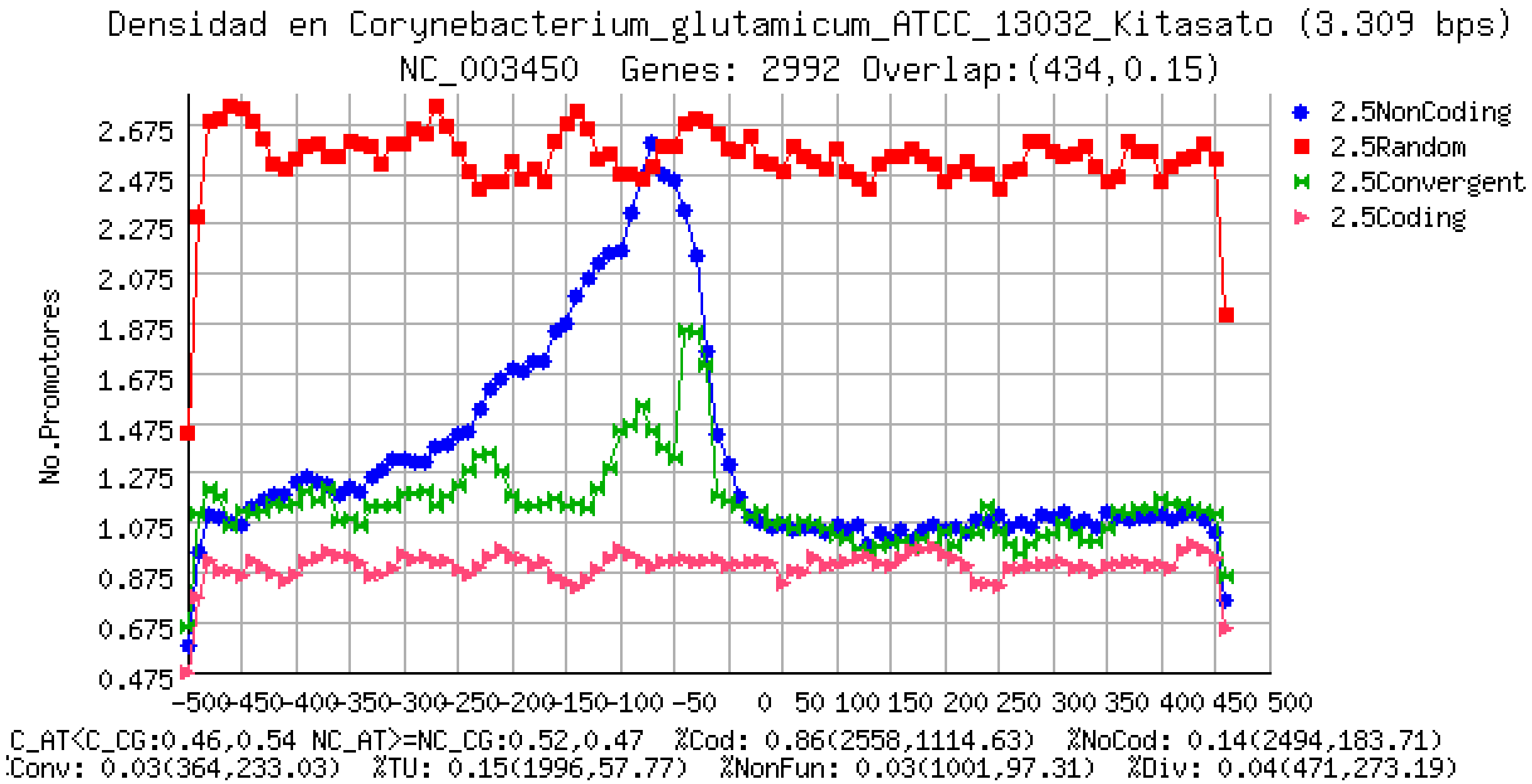
Densidad en Corynebacterium_glutamicum_ATCC_13032_Kitasato (3.309 bps)
NC_003450 Genes: 2992 Overlap:(434,0.15)
No.Promotores
2.5NonCoding
2.5Random
2.5Convergent
2.5Coding
C_AT<C_CG:0.46,0.54 NC_AT>=NC_CG:0.52,0.47 %Cod: 0.86(2558,1114.63) %NoCod: 0.14(2494,183.71)
%Conv: 0.03(364,233.03) %TU: 0.15(1996,57.77) %NonFun: 0.03(1001,97.31) %Div: 0.04(471,273.19)

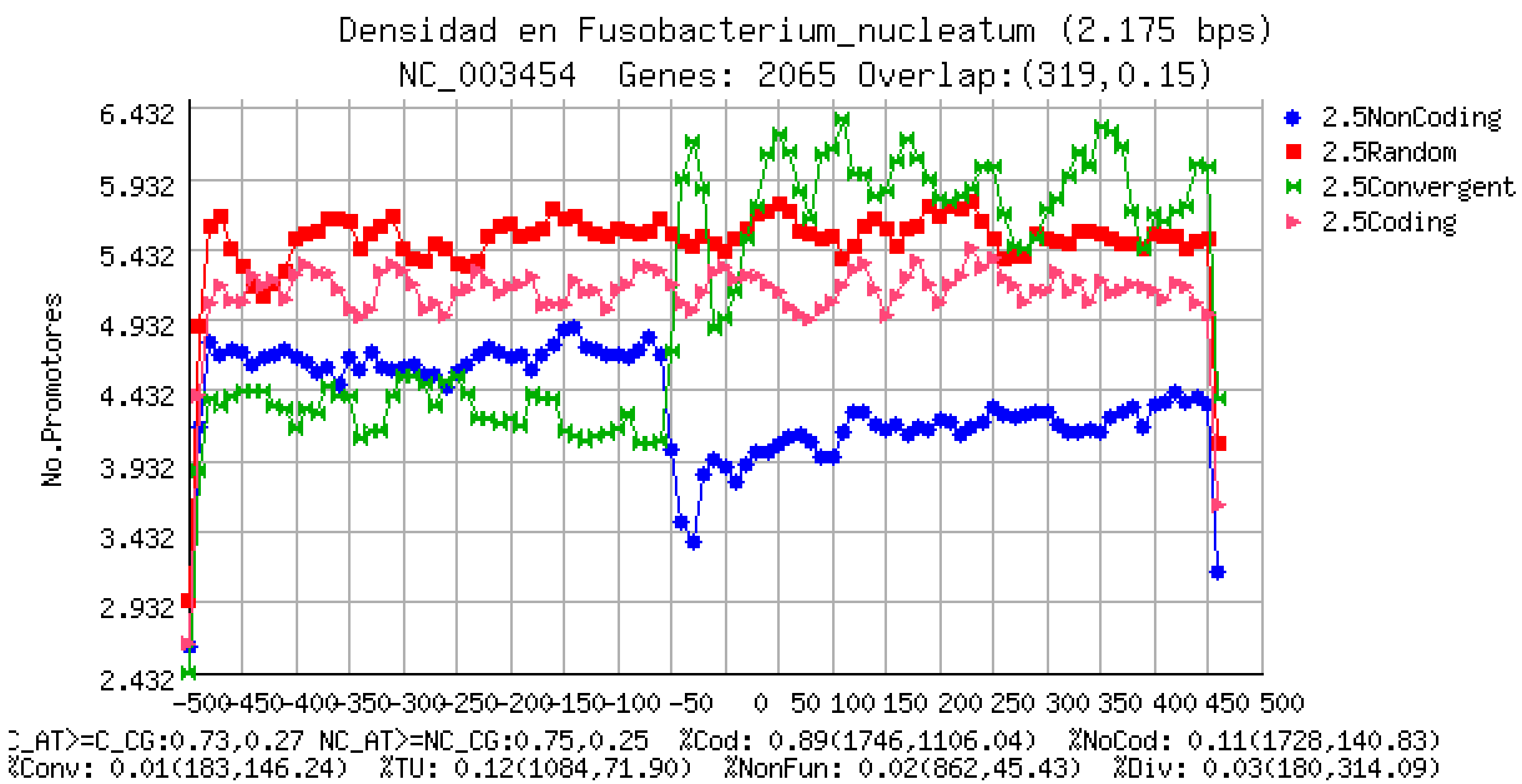
Densidad en Fusobacterium_nucleatum (2.175 bps)
NC_003454 Genes: 2065 Overlap:(319,0.15)
No.Promotores
6.432
5.932
5.432
4.932
4.432
3.932
3.432
2.932
2.432
-500 -450 -400 -350 -300 -250 -200 -150 -100 -50 0 50 100 150 200 250 300 350 400 450 500
2.5NonCoding
2.5Random
2.5Convergent
2.5Coding
C_AT>=C_CG:0.73,0.27 NC_AT>=NC_CG:0.75,0.25 %Cod: 0.89(1746,1106.04) %NoCod: 0.11(1728,140.83)
%Conv: 0.01(183,146.24) %TU: 0.12(1084,71.90) %NonFun: 0.02(862,45.43) %Div: 0.03(180,314.09)

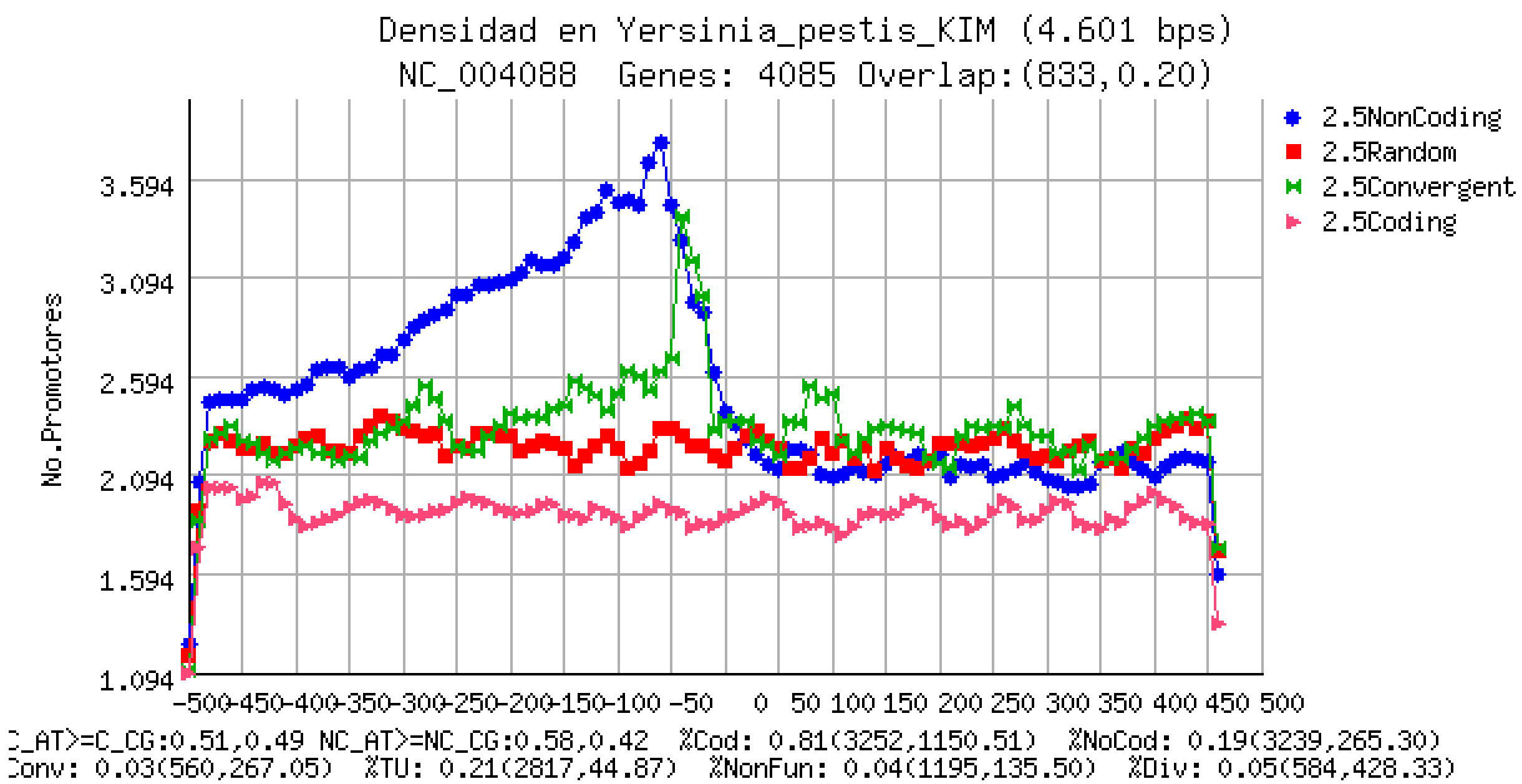
Densidad en Yersinia_pestis_KIM (4.601 bps)
NC_004088 Genes: 4085 Overlap:(833,0.20)
2.5NonCoding
2.5Random
2.5Convergent
2.5Coding
No.Promotores
3.594
3.094
2.594
2.094
1.594
1.094
-500-450-400-350-300-250-200-150-100 -50 0 50 100 150 200 250 300 350 400 450 500
C_AT>=C_CG:0.51,0.49 NC_AT>=NC_CG:0.58,0.42 %Cod: 0.81(3252,1150.51) %NoCod: 0.19(3239,265.30)
Conv: 0.03(560,267.05) %TU: 0.21(2817,44.87) %NonFun: 0.04(1195,135.50) %Div: 0.05(584,428.33)

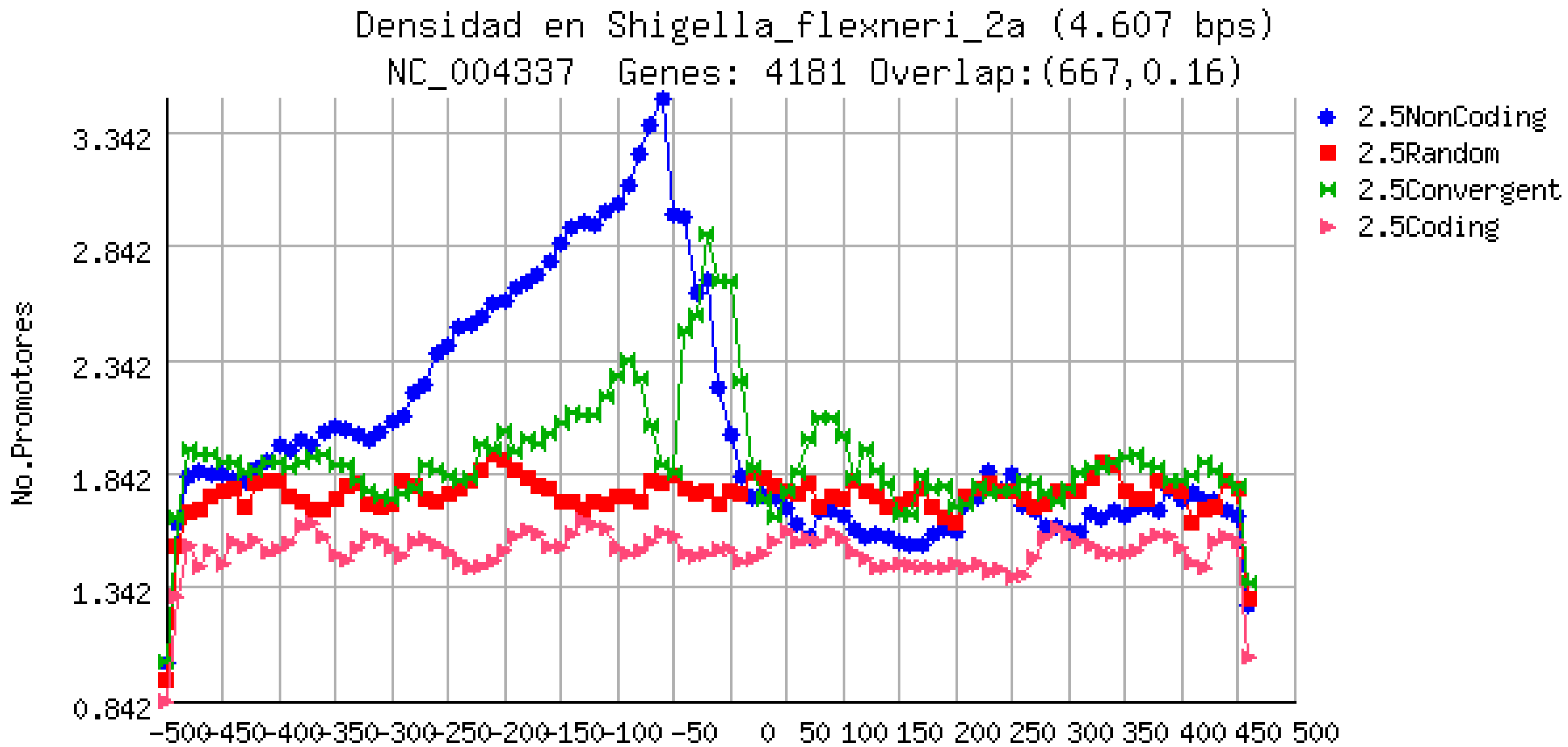

C_AT<C_CG:0.48,0.52 NC_AT>=NC_CG:0.54,0.46 %Cod: 0.80(3514,1048.04) %NoCod: 0.20(3489,264.95)
onv: 0.04(561,316.75) %TU: 0.21(2709,129.24) %NonFun: 0.04(1447,135.83) %Div: 0.05(625,362.46)

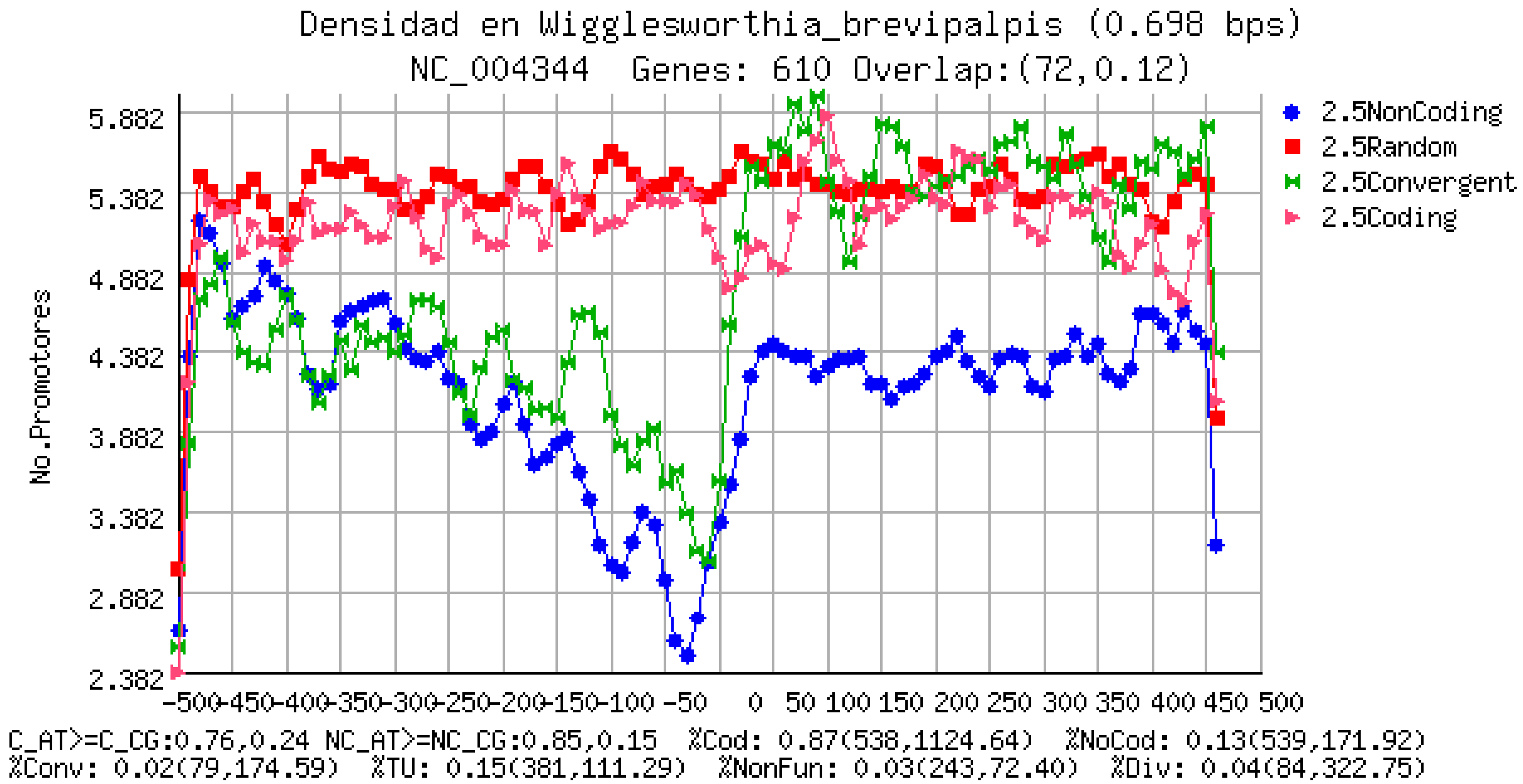
Densidad en Wigglesworthia_brevipalpis (0.698 bps)
NC_004344 Genes: 610 Overlap:(72,0.12)
No.Promotores
5.882
5.382
4.882
4.382
3.882
3.382
2.882
2.382
-500 -450 -400 -350 -300 -250 -200 -150 -100 -50 0 50 100 150 200 250 300 350 400 450 500
2.5NonCoding
2.5Random
2.5Convergent
2.5Coding
C_AT>=C_CG:0.76,0.24 NC_AT>=NC_CG:0.85,0.15 %Cod: 0.87(538,1124.64) %NoCod: 0.13(539,171.92)
%Conv: 0.02(79,174.59) %TU: 0.15(381,111.29) %NonFun: 0.03(243,72.40) %Div: 0.04(84,322.75)

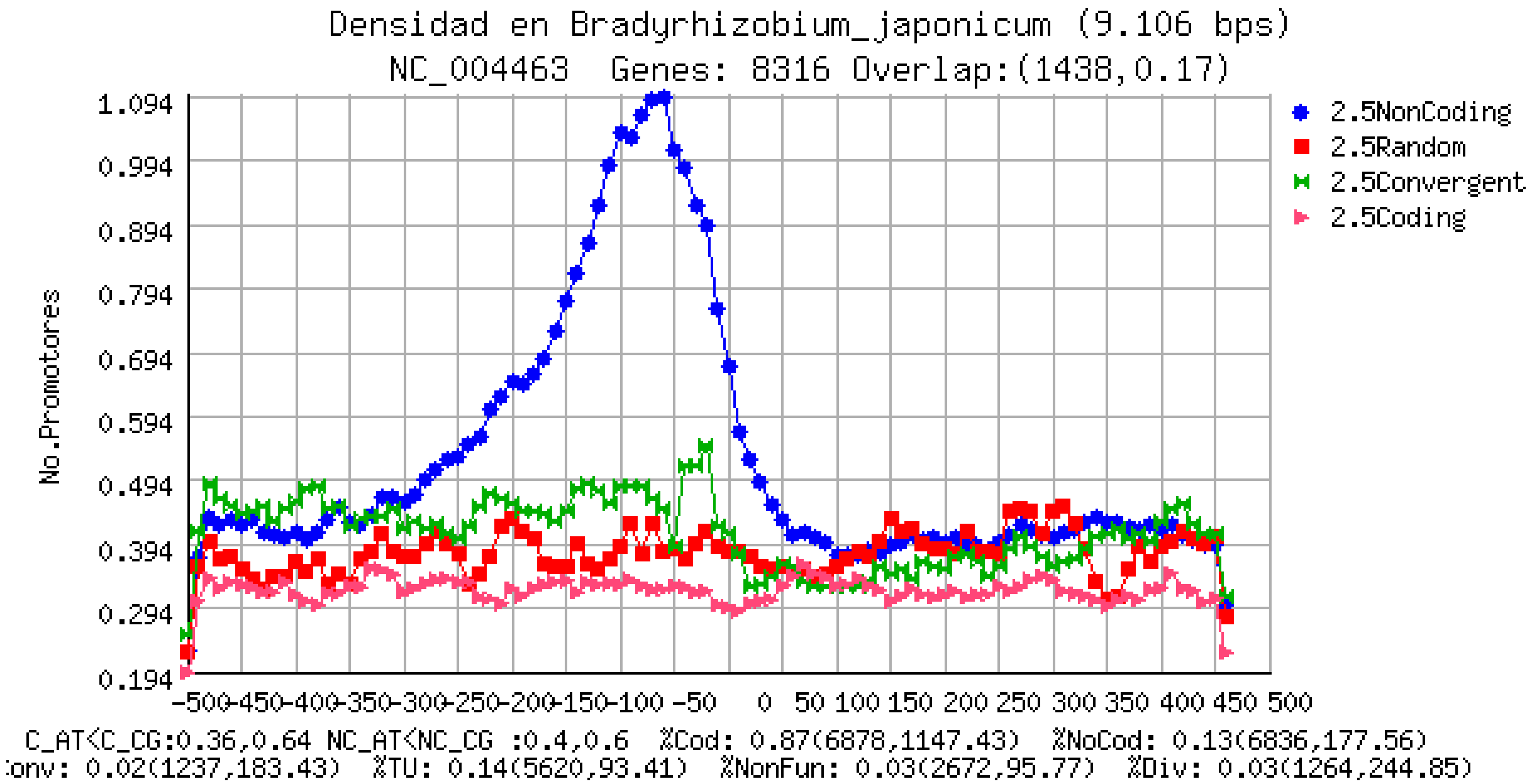
Densidad en Bradyrhizobium_japonicum (9.106 bps)
NC_004463 Genes: 8316 Overlap:(1438,0.17)
No.Promotores
1.094
0.994
0.894
0.794
0.694
0.594
0.494
0.394
0.294
0.194
-500-450-400-350-300-250-200-150-100 -50 0 50 100 150 200 250 300 350 400 450 500
2.5NonCoding
2.5Random
2.5Convergent
2.5Coding
C_AT<C_CG:0.36,0.64 NC_AT<NC_CG :0.4,0.6 %Cod: 0.87(6878,1147.43) %NoCod: 0.13(6836,177.56)
onv: 0.02(1237,183.43) %TU: 0.14(5620,93.41) %NonFun: 0.03(2672,95.77) %Div: 0.03(1264,244.85)

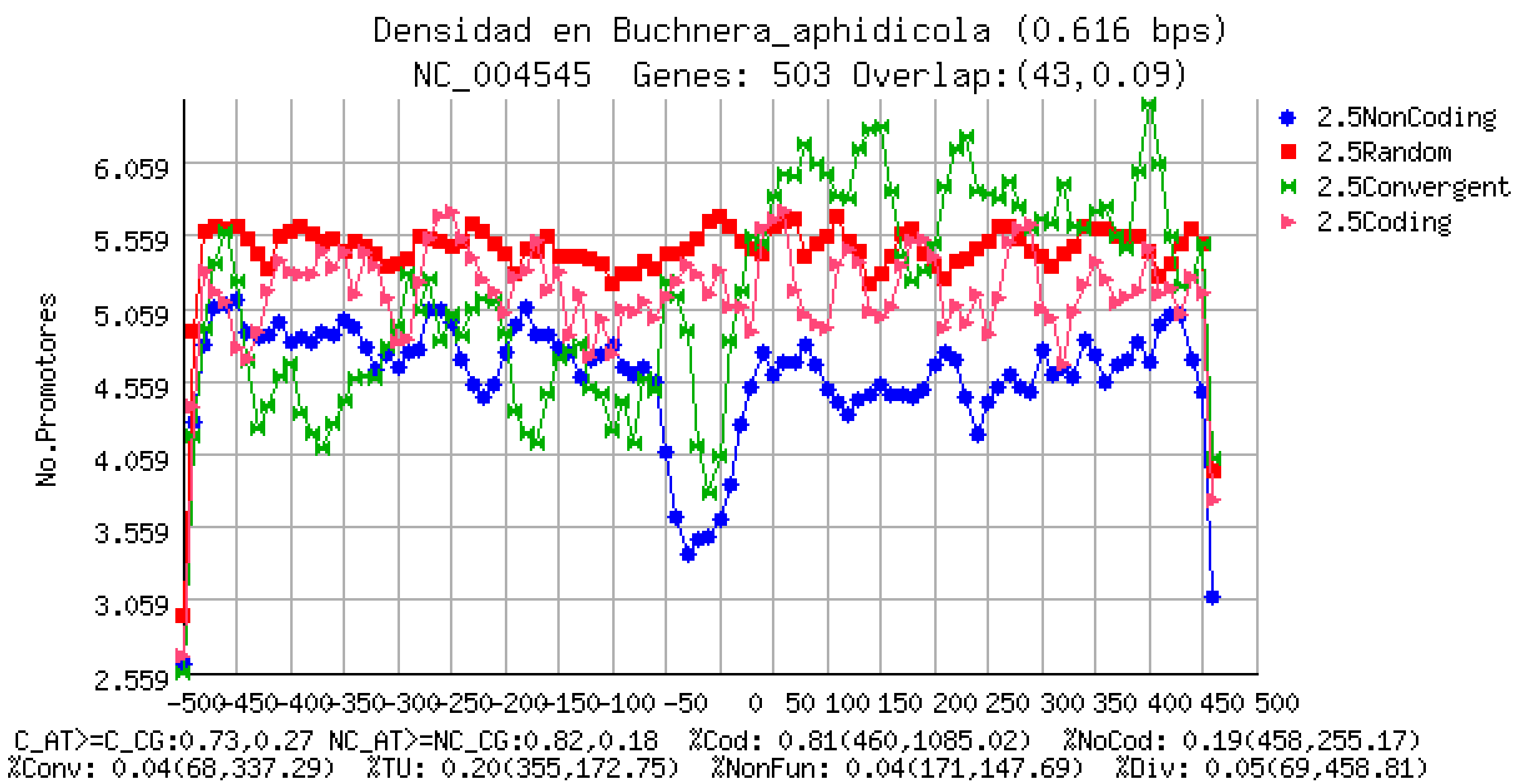
Densidad en Buchnera_aphidicola (0.616 bps)
NC_004545 Genes: 503 Overlap:(43,0.09)
No.Promotores
6.059
5.559
5.059
4.559
4.059
3.559
3.059
2.559
-500 -450 -400 -350 -300 -250 -200 -150 -100 -50 0 50 100 150 200 250 300 350 400 450 500
2.5NonCoding
2.5Random
2.5Convergent
2.5Coding
C_AT>=C_CG:0.73,0.27 NC_AT>=NC_CG:0.82,0.18 %Cod: 0.81(460,1085.02) %NoCod: 0.19(458,255.17)
%Conv: 0.04(68,337.29) %TU: 0.20(355,172.75) %NonFun: 0.04(171,147.69) %Div: 0.05(69,458.81)
