# Supplementary material for: Selection for Unequal Densities of σ70 Promoter-Like Signals in Different Regions of Large Bacterial Genomes
Source: PLoS Genet. 2006 Nov 10;2(11):e185. doi: 10.1371/journal.pgen.0020185 (PMC1635534; doi:10.1371/journal.pgen.0020185)
Supplement: Supplementary file 3 [file pgen.0020185.st001.pdf]

Table S1. Detailed examples of *M. leprae* pseudogenes with ortholog genes in *M. tuberculosis*

Pseudogenes*	Promoter-like Signals		GC Content		Average Score	
	M_tuberculosis	*M_leprae*	*M_tuberculosis*	*M_leprae*	*M_tuberculosis*	*M_leprae*
BfrB	13	62	0.51	0.31	6.08	5.77
cmaA1	1	26	0.65	0.48	4.41	4.77
Icl	12	11	0.53	0.49	4.24	4.11
lldD1	2	11	0.72	0.52	3.24	5.13
UmaA	6	13	0.56	0.55	4.91	3.94
gnd2	1	6	0.65	0.56	6.04	4.68
glnA3	2	13	0.62	0.58	3.75	3.52
MetC	1	4	0.68	0.58	6.15	4.1
PdhB	1	1	0.63	0.58	3.71	4.28
CitA	1	6	0.62	0.59	2.86	4.22
LigB	2	4	0.62	0.6	4.07	3.6
icd1	4	6	0.67	0.6	4.29	4.63
glnA4	8	12	0.62	0.6	3.54	3.38
PdhA	4	3	0.63	0.61	4.18	2.87
PdhC	2	4	0.63	0.62	3.78	3.69
LpdA	3	7	0.68	0.63	3.66	3.05

* *M. leprae* pseudogenes whose orthologues in *M. tuberculosis* are functional genes. Information about of the metabolic pathways where these genes are involved is described in [Cole ST et al. 2001. Nature 409:1007-1011]. Data are ordered by the GC content of the upstream regions of the *M. leprae* pseudogenes. Except for *icl*, *pdhA* and *pdhB*, there is a trend to get more promoter-like signals in the upstream regions of pseudogenes. This increase of signals is related with the increase of mutations from GC to AT bases. 69% of *M. leprae* pseudogenes have a average promoter score lower than their ortholog gene in *M. tuberculosis* suggesting an effect of the AT-enrichment in the degradation of the promoter signal.
